# Genetic Variation in the Domain II, 3′ Untranslated Region of Human and Mosquito Derived Dengue Virus Strains in Sri Lanka

**DOI:** 10.3390/v13030421

**Published:** 2021-03-05

**Authors:** P. D. Dayananda, Harendra de Silva, LakKumar Fernando, B. G. D. N. K. de Silva

**Affiliations:** 1 Center for Biotechnology, Department of Zoology, Faculty of Applied Sciences, University of Sri Jayewardenepura, Nugegoda 10250, Sri Lanka; dilakshini@sci.sjp.ac.lk; 2 Department of Pediatrics, Faculty of Medicine, University of Colombo, Colombo 00700, Sri Lanka; harendra51@gmail.com; 3 Centre for Clinical Management of Dengue & Dengue Hemorrhagic Fever, Negombo 11500, Sri Lanka; lakkumar@gmail.com

**Keywords:** dengue virus, domain II, 3′ untranslated region, genetic variations, Sri Lanka

## Abstract

Genetic variations in dengue virus (DENV) play a distinct role in epidemic emergence. The DENV 3′ UTR has become a recent interest in research. The objective of the study was to examine the genetic variation in the domain II, 3′ UTR region of human and mosquito-derived DENV. DENV-infected human sera were orally infected to laboratory reared *Aedes aegypti* mosquitoes. The domain II, 3′ UTR of each human- and mosquito-derived sample was amplified. The nucleotide sequence variation, phylogenetic and secondary structure analysis was carried out incorporating respective regions of so far recorded Sri Lankan and the reference genotype strains of the DENV3 and DENV1 serotypes. The human- and mosquito-derived domain II, 3′ UTR were identical in nucleotide sequences within the serotypes isolated, indicating the conserved nature of the region during host switch. The sequence analysis revealed distinct variations in study isolates compared to so far recorded Sri Lankan isolates. However, despite single nucleotide variations, the maintenance of structural integrity was evident in related strains within the serotypes in the secondary structure analysis. The phylogenetic analysis revealed distinct clade segregation of the study sequences from so far reported Sri Lankan isolates and illustrated the phylogenetic relations of the study sequences to the available global isolates of respective serotypes.

## 1. Introduction

Dengue, the most common vector-borne viral disease of humans, has been distributed throughout the tropics and is now invading the subtropical regions of the world, causing frequent and recurrent epidemics [1,2,3]. Although, most of these epidemics are known to be caused by fluctuations of the prevalence of DENV serotypes in a background of low herd serotype specific immunity, genetic variations in DENV appear to play a distinct role in epidemic emergence [4]. The factors that lead to DENV genetic variability and viral replacements in nature are not well understood. However, a complex network of host–virus interactions, together with environmental factors, may account for the transmission of certain viral variants over others [5].

Genetic variations between serotypes, genotypes and lineages are important determinants for differential viral fitness, virulence and epidemic potential [6,7]. Global genotype replacement events have been observed in different regions [8,9,10,11] including Sri Lanka, coinciding with severe dengue prevalence [12,13,14,15]. Sri Lanka experienced a major epidemic in 2017 (186,101 cases) in the recent past, a significant reduction of dengue cases was observed in 2018 (51,659 cases), while a two-fold increase in dengue cases was observed by the end of 2019 (105,049 cases) [16] suggesting a possible strain/genotype shift or a genotype/clade replacement event.

DENV genotype classification has been continuously changed, depending on the area of concern in the genome. Different areas of the DENV genome evolve at different rates and sometimes exhibit areas of higher mutation rates within a region, as evident in the E gene or 3′ UTR [17]. So far, genotype classification within each serotype is carried out mainly based on the E gene sequences. Interestingly, the nucleotide composition in the 3′ UTRs is known to differentiate dominant strains from weaker DENV strains, as observed in many dengue endemic countries [14,18,19]. However, the 3′ UTR has been yet less utilized in sequence characterization and phylogenetic assessments [14,17] while the structural consequences and impact of the nucleotide variations on viral fitness have yet to be experimentally defined [20].

DENV 3′ UTR is made of stem loops (SLs), two dumbbell (DB) structures and conserved sequences (CSs) and repeat conserved sequences (RCSs). The length of the 3′ UTR varies depending on the serotype/strain of the virus, but it is mainly divided into three domains. Domain I is situated immediately downstream the stop codon, consisting of a region with a variable nucleotide sequences and two SL structures, domain II is moderately conserved with two DB structures incorporating conserved regions; repeated conserved sequence2 (RCS2) and conserved sequence2 (CS2) regions and domain III is highly conserved with one SL structure [21]. The biological significance of maintaining two almost identical RNA structures in the 3′ UTR of flaviviruses is not yet fully resolved. Redundant functions such as replication enhancers have been proposed for the domain II 3′ UTR which composes two DB elements [22,23]. Further, DB structures are proposed as complex RNA elements that accommodate multiple signals that modulate viral process [24]. However, predictions of the distinct folding intermediates of DB1 and DB2 have suggested possible distinct functions [23].

A fascinating feature of the 3′ UTR of flavivirus genomes is the evolutionary conservation of sequence repeats and duplicated RNA structures [25]. The 3′ UTR of DENV contains two almost identical SL structures (SLI and SLII) and two similar DB elements (DB1 and DB2). These two pairs of duplicated RNA elements acquire stable secondary structures, which can stall the genome degradation [26] during the process of generating subgenomic flavivirus RNA (sfRNA). Depending on which secondary structure the degradation stalls at, four different species of sfRNA (sfRNA1-4) have been identified [5]. The sfRNA help the virus evade the host immune response [21] leading to epidemiological fitness [27]. Evidence prevails for lineage replacement events that has correlated with sfRNA production-related virulence [28,29]. The domain II and domain III regions are the mostly represented regions in the sfRNAs. With the domain III region being highly conserved, the domain II region, with considerable variability, is worth investigating for the genetic variations in order to have an insight of the DENV circulation.

A major source of DENV genetic diversity is the natural alternation among vertebrate and invertebrate hosts, which imposes different selective pressures on the viral population [5,30,31]. The source of the selective pressure and the mechanisms that explain the positive and negative selection of viral variants during host adaptation is still largely unknown. It has been revealed that sequence variations in the 3′ UTR, acquired during host adaptation, are associated with the generation of sfRNAs [32].

The most recent findings on RNA structure duplications are reported by de Borba et al. [24], which imply that each of the duplicated DB structures in the DENV3′ UTR is under different selective pressure in adult mosquitoes. It was proposed that the maintenance of double copies of RNA structures is a viral strategy to ensure the functionality of one conserved element while the other is under a different selective pressure within the two hosts [24]. These observations have raised concerns regarding the mechanisms by which the viral RNA structures act in mosquitoes and humans, and the implications of the genetic variations of the 3′ UTR in host adaptation, transmission, and pathogenesis.

To expand the scientific knowledge in this area of interest, the study examined the genetic variation of the domain II, 3′ UTR of the DENV sequences derived from dengue-confirmed patient serum and the respective laboratory-infected *Aedes aegypti* mosquito vectors and analyzed the genetic variations with the corresponding regions of the so far reported Sri Lankan strains and the reference global genotypes for respective serotypes.

## 2. Materials and Methods

### 2.1. Ethical Approval

The study was approved by the Ethics Review Committee, Faculty of Medicine, University of Sri Jayewardenepura, Sri Lanka on 24 June 2014 (Application number 785/13). All patients were provided with detailed information about the study and written consent was obtained before the patient was recruited for the study.

### 2.2. Sample Collection

From the dengue-confirmed patients admitted to the Centre for Clinical Management of Dengue and Dengue Hemorrhagic Fever (CCMDDHF), Negombo, Sri Lanka, in January 2019, six patients within the first 5 days of onset of fever were selected. One drop of whole blood from each patient was used for a rapid immunochromatographic test (ICT) to confirm the viremic state. The presence of the dengue NS1 antigen was determined using NS1Ag strips (Standard Diagnostics, Korea) according to the manufacturer’s recommendations. The test results were confirmed by experienced healthcare professionals attached to CCMDDHF. The patients who produced positive dengue NS1Ag results were proceeded (n = 5).

Blood was collected (3 mL) via venipuncture by a phlebotomist. The blood (1 mL) was separated and mixed with an anticoagulant (Sodium citrate 3.8%) for mosquito inoculation experiments. Serum was separated from the remaining whole blood by centrifugation (Centurion Scientific C2 centrifuge, Chichester, UK), labelled and preserved at −60 °C for molecular diagnosis.

### 2.3. Laboratory Rearing of Ae. aegypti Mosquitoes

Mosquito rearing procedures were followed according to standard protocols [33,34]. The colony was maintained at the insectary, Department of Zoology, Faculty of Applied Sciences, University of Sri Jayewardenepura, Sri Lanka. F1–F5 progenies were obtained accordingly and progenies after F5 were used for experiments to reduce the chance of any possible transovarian transmission of DENV. The subsamples of P, F1 and F2 progenies were tested for the DENV infection using NS1Ag strips as specified above and the un-infectivity was confirmed and maintained throughout the experiment.

### 2.4. Mosquito Inoculation: Oral Infection of Laboratory-Infected Mosquitoes

Laboratory-reared, five day old female *Ae. aegypti* mosquitoes were selected for the study. Mosquitoes were starved for 24 h overnight [35] prior to the experiment. Anticoagulated whole blood (1 mL) from each dengue NS1-positive patient (D1H_2019SL-D5H 2019SL) was orally fed to thirty *Ae. aegypti* mosquitoes within 10 min of blood drawing [36] via a membrane feeding apparatus [34,37]. Mosquitoes were blood fed for 25 min [38]. The mosquitoes for negative control were fed with a non-infectious blood meal using the same procedure and the apparatus. Immediately after the feeding trials, mosquitoes were cold anesthetized and only engorged females were transferred to a separate cage for extrinsic incubation of the DENV. They were maintained in sealed containers with double barriers in the insectary (28 °C, 70–80% relative humidity) during the extrinsic incubation period (EIP) of 14 days. The mosquitoes who survived the complete incubation of 14 days were (12, 10, 11, and 9 mosquitoes, respectively, from D1H_2019SL-D5H_2019SL infectious blood fed cages) euthanized by freezing and stored individually in TRIzole reagent (Invitrogen Corp., Carlsbad, CA, USA) at −80 °C for further analysis.

### 2.5. Molecular Diagnosis of Human Serum and Mosquito Samples

The viral RNA was extracted from serum samples using a QIAmp Viral RNA Mini kit (Qiagen, Valencia, CA, USA) following the manufacturer’s recommendations. The extracted RNA was stored in −80 °C until reverse transcriptase polymerase chain reaction (RT-PCR).

Each individual mosquito stored in −80 °C was homogenized in triazole reagent on an ice platform and total RNA was extracted from 200 μL of each specimen by using the TRIzole reagent (Invitrogen Corp., Carlsbad, CA, USA), according to the manufacturer’s recommendations. The extracted RNA was stored in −80 °C until RT-PCR.

All RT-PCR reactions were performed in a Master Cycler personnel(Eppendorf, Hamburg, Germany).A single step RT-PCR system (Qiagen one step RT-PCR kit, Hilden, Germany) was used to amplify the domain II, 3′ UTR region of the DENV genome in a total volume of 25 μL according to the manufacturer’s recommendations using two forward primers (ALD-1 and ALD-1b) and a reverse primer (ALD-2) [39]. The RT-PCR reaction mixture (25 µL) contained 2 µg of extracted RNA and 20 μL of RT-PCR master mix. The thermal cycling conditions were: reverse transcription at 50 °C for 30 min, initial PCR activation at 95 °C for 10 min, followed by 35 cycles of 94 °C for 30 s, 55 °C for 1 min and 72 °C for 30 s, followed by a final extension at 72 °C for 10 min.

The PCR amplified fragments were subjected to gel electrophoresis (Electrophoresis apparatus (Labnet International Inc. USA) in Ethidium bromide stained 1.5% agarose (Promega, Madison, WI, USA) gels. The DNA bands were visualized and imaged under ultraviolet (UV) transillumination using a gel documentation system (AlphaImager MINI, France). The positive PCR products were sent to Macrogen Inc. Korea for sequencing.

### 2.6. Nucleotide Sequence Analysis

The forward and reverse sequences were assembled and consensus contig assembly was performed using a DNA baser sequence assembler V.4 (2013) (Heracle BioSoft, www.DnaBaser.com accessed on 7 July 2019). The contig sequences were edited using Bio Edit sequence alignment editor [40] and Mega X software [41].

The assembled sequences were subjected to BLAST (Basic Local Alignment Search Tool) on the NCBI database, to carry out the sequence similarity search. Sequence homology was used to identify the respective DENV serotype.

Sequences identified in the study with the respective DENV3 and DENV1 reference genotype sequences [17,42,43,44] and the respective regions of the DENV3 and DENV1 serotypes recorded from Sri Lanka [13,14,45,46] (Tables S1 and S2) were aligned using CLUSTALW program (Bioinformatics Center, Institute for Chemical Research, Kyoto University, Uji, Kyoto 611-0011, Japan) and visually inspected to ensure correct alignment. Sequences were further aligned with the respective prototypes for each serotype [47] (M93130 for DENV3 and EU848545 for DENV1) using the ClustalW program in MEGA X software [41]. Nucleotide sequence variations were identified with respect to the prototype reference sequence for each serotype. The single-nucleotide variation (SNV) frequency with regard to the genome position of each data set was analyzed using Microsoft Excel (2016).

A sequence similarity search was carried out using BLAST on the NCBI database for the respective domain II, 3′ UTR sequences, in order to identify the homologues sequences to the sequences isolated in the study.

### 2.7. Phylogenetic Analysis

DENV 3′ UTR, domain II region sequences identified in the study, the DENV3 and DENV1 serotypes so far recorded from Sri Lanka (isolates which contained the sequences for respective domain II, 3′ UTR sequences) [13,14,45,46] and reference strains for DENV3 genotypes (isolates which contained the sequences for the respective domain II, 3′ UTR sequences) (Table S1) and reference strains for DENV1 genotypes (isolates which contained the sequences for respective domain II, 3′ UTR sequences) (Table S2) [17,42,43,44] available in GenBank were used for the phylogenetic analysis. Moreover, DENV2 reference strains (AF038403, KM204118) and DENV4 reference strains (AY947539, KR011349) [47] were used as outgroups. Furthermore, to examine the relationship between the so far reported global strains, respective DENV3 and DENV1 sequences identified in the study were analyzed with the so far reported Sri Lankan isolates and the homologues DENV3 (Table S3) and DENV1 (Table S4) isolates resulted from the sequence similarity search.

The aligned sequences were analyzed in the model test program partition finder [48] to identify the best fit model of nucleotide substitution for phylogenetic reconstruction; in all the analyses, the general time reversible model of nucleotide substitution with gamma-distribution rates among invariant sites (termed as the GTR+G model) was found to be the best fit model.

Bayesian phylogenetic analysis was carried out employing Bayesian Markov Chain Monte Carlo method of Mr. Bayes version 4.1.2 [49]. Posterior probabilities (PP) of phylogenetic trees were estimated using 6 million generations (sampled every 1000 generations) and two Metropolis-coupled Markov chain Monte Carlo (MCMC) to allow adequate time and mixing for convergence. The first 25% of sampled trees were considered as burn in. The consensus tree was visualized using Figtree v.1.3.2.

### 2.8. Secondary Structure Analysis

For RNA structure analysis, the domain II region of the 3′ UTR of the study-identified sequences, corresponding regions of the DENV3 and DENV1 isolates representative of different genotypes, along with the corresponding regions of the so far reported DENV1 and DENV3 Sri Lankan isolates were used.

The consensus structures for the prediction for RNA alignments of DENV3 and DENV1 study-identified sequences were performed by RNAalifold program implemented by Vienna RNA package [50], accessed through the Jalview desktop graphical user interface (GUI) (version 2) [51] to understand the biological significance of DB structure duplication.

The prediction of secondary structures of the DB1 and DB2 of the 3′ UTR domain II region was performed using the webservers MFold [52] and RNAfold [53]. They were used with default folding parameters and folding predictions at 37 °C.

## 3. Results

### 3.1. Results of RT-PCR and Sequence-Based Serotype Analysis of the Human-Derived and Mosquito-Derived DENV Samples to Examine the Domain II, 3′ UTR

The five serum samples that were positive for NS1 were subjected to RT-PCR (Figure S3) and the nucleotide sequences of the amplified products were analyzed with the 3′ UTR sequences in the NCBI database. Based on the similarity BLAST search, the sequences revealed that the five serum samples belonged to DENV3 and DENV1 serotypes (Table 1). Moreover, the amplified 3′ UTR regions of each DENV1 (D1H_2019SL and D3H_2019SL) and DENV3 (D4H_2019SL and D5H_2019SL) were 100% identical. Table S5 shows the GenBank accession numbers obtained for the study isolates of human-derived samples.

The oral mosquito feeding experiment revealed successful mosquito infections in four out of five (4/5) infectious blood samples, according to the results of the RT-PCR in mosquito tissues (Table 1, Figure S4). The DENV nucleotide sequence obtained for each infected individual mosquito was identical to that of the respective human host (as in mosquito-derived DENV3 sequences to human-derived DENV3 sequences and mosquito-derived DENV1 sequences to human-derived DENV1 sequences). Consequently, the mosquito-derived DENV3 sequences (D4M1_2019SL-D4M7_2019SL and D5M1_2019SL-D5M4_2019SL) and DENV1 sequences (D1M1_2019SL-D1M5_2019SL and D3M1_2019SL-D3M6_2019SL) were revealed to be belonging to the respective DENV serotypes 3 and 1 (percentage identity 100%) (Table 1). Table S5 shows the GenBank accession numbers obtained for study-identified mosquito-derived samples.

### 3.2. Sequence Analysis for Nucleotide Variation

**Figure 1:**
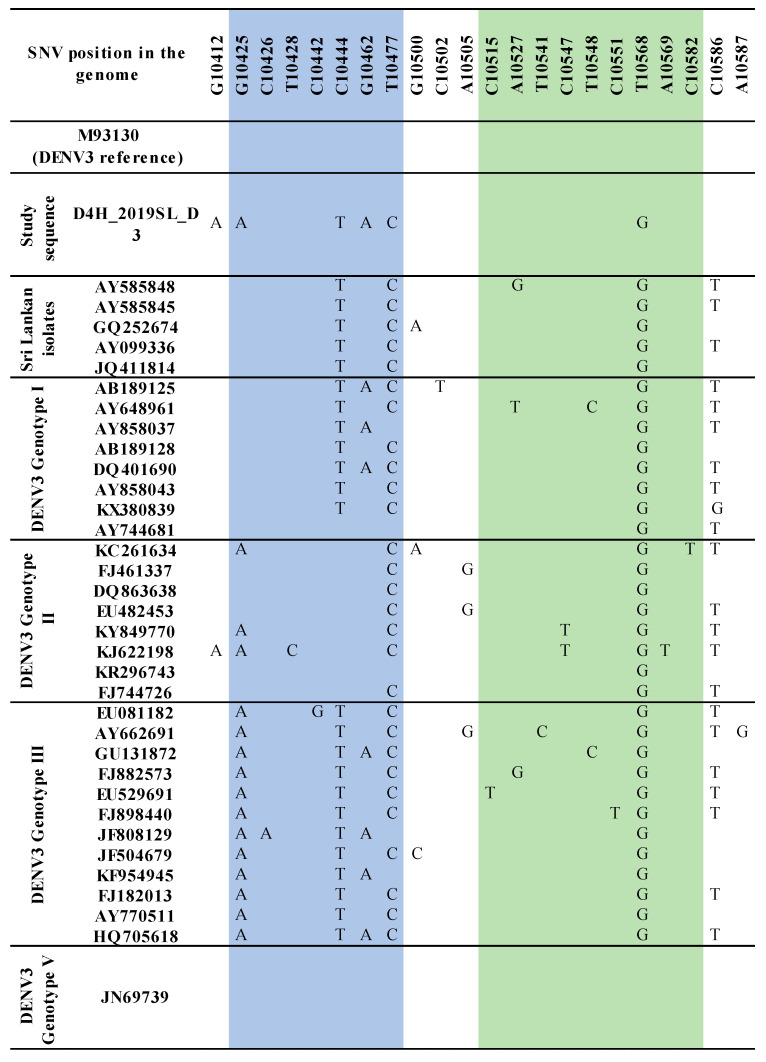
Summary of the single-nucleotide variation (SNV) in DENV3 domain II, 3′ UTR region. A DENV3 sequence identified in the study, DENV3 isolates of Sri Lanka and DENV3 genotypes I–V. SNVs are mapped according to the DENV3 reference strain (GenBank accession number M93130). The DB1 region is highlighted in blue and the DB2 region is highlighted in green.

The sequence analysis of the domain II, 3′ UTR of DENV3 samples identified from the study revealed sequence variations compared to that of the so far reported Sri Lankan isolates (Figure S1 and Figure 1). Distinct SNV was observed with respect to each DENV3 genotype studied. In DENV3 genotypes except for genotype V (genotype I–IV), the mutations T10477C and T10568G were observed in the RCS2 and CS2 regions, respectively (Figure S1).

**Figure 2:**
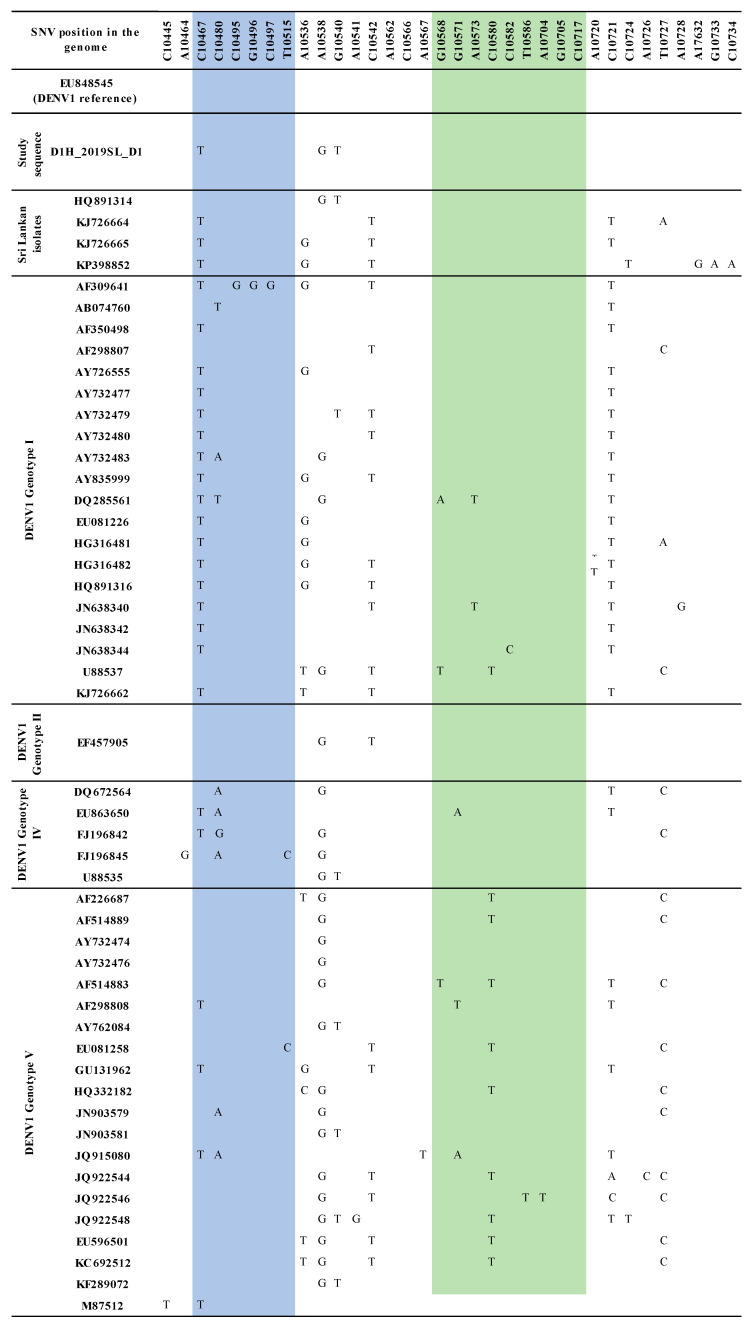
Summary of the SNV in DENV1 domain II, 3′ UTR region. A DENV1 sequence identified in the study, DENV1 isolates of Sri Lanka and DENV1 genotypes I–V. SNVs are mapped according to the DENV1 reference strain (GenBank accession number EU8485). The DB1 region is highlighted in blue and the DB2 region is highlighted in green.

Similarly, sequence variations were observed in domain II, 3′ UTR of DENV1 sequences identified from the study compared to that of the so far reported Sri Lankan DENV1 isolates (Figure S2 and Figure 2). Distinct SNV was observed with respect to each genotype of DENV1 studied. No mutations were observed in the RCS2 region (except for two isolates; one in DENV1 genotype IV and one in DENV1 genotype V) and in the CS2 region (except for one DENV1 genotype V isolate) (Figure S2).

Six mutations were observed among the DENV3 isolates used in the study (G10,412A, G10,425A, C10,444T, G10,462A, T10.462A, T10,477C, T10,568C). Mutations, G10,412A (in the spacer region), G10,425A and G10,462A (in the DB1 region) were not shared by other reported Sri Lankan isolates (Figure 1).

Three mutations were observed in the DENV1 sequences used in the study (C10,467T, A10,538G, C10,540T). Mutation C10,467T, which was positioned in the DB1 region, was found commonly shared among all the genotypes as well as among the other Sri Lankan isolates except in HQ891314. Interestingly, A10,538G and C10,540T mutations in the spacer region following the DB1 were shared only with HQ891314 among the other Sri Lankan isolates (Figure 2).

**Figure 3:**
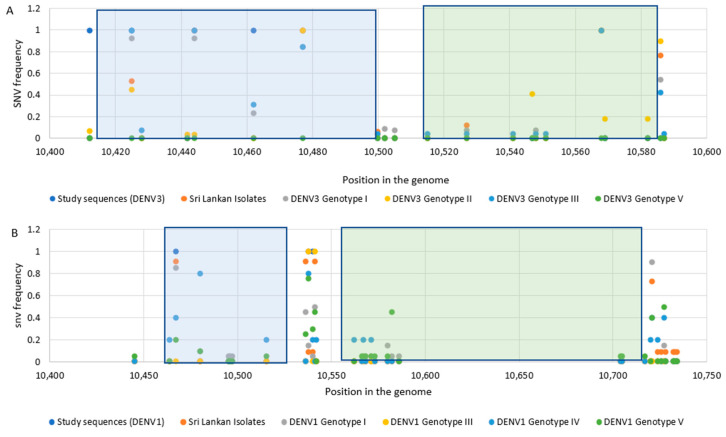
SNV frequency in 3′ UTR domain II of DENV3 and DENV1 isolates. SNV frequency in 3′ UTR domain II of (**A**) DENV3 sequences (DENV3 sequences identified in the study, DENV3 Sri Lankan isolates, DENV3 genotype I isolates, DENV3 genotype II isolates, DENV3 genotype III isolates and DENV3 genotype V isolates, (**B**) DENV1 sequences (DENV1 sequences identified in the study, DENV1 Sri Lankan isolates, DENV1 genotype I isolates, DENV1 genotype III isolates, DENV1 genotype IV isolates and DENV1 genotype V isolates. The DB1 region is highlighted in blue and the DB2 region is highlighted in green.

Distinct patterns of SNV were observed among the genotypes within each serotype. Mutations observed in the RCS2 region were contained in the DB1 region while mutations in the CS2 region contained in the DB2 region. According to the SNV frequency illustrated in Figure 3, it was shown that not only the SNV in DB regions but also the SNV in spacer regions were frequent among the isolates.

### 3.3. Phylogenetic Analysis

**Figure 4:**
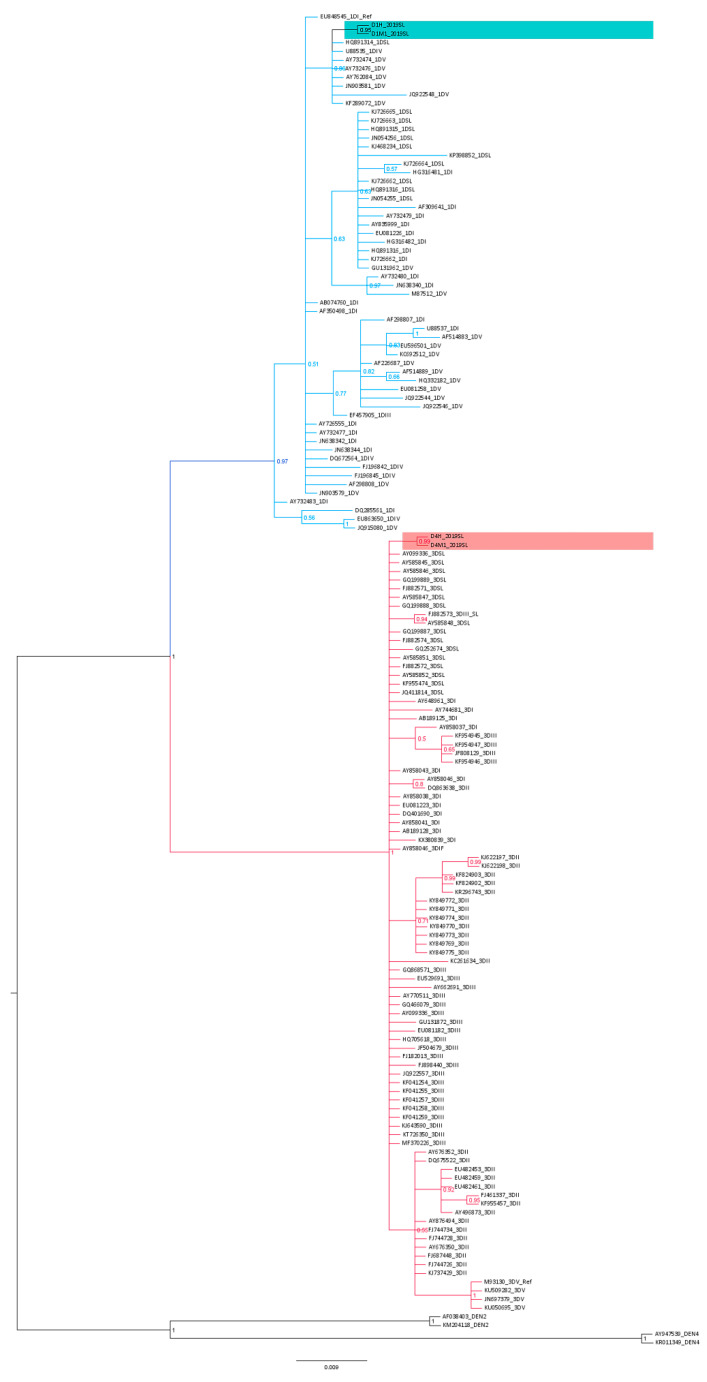
Calibrated maximum-clade-credibility tree for DENV3 and DENV1. DENV3 and DENV1 study-identified sequences, so far reported Sri Lankan isolates and reference genotype strains, based on domain II, 3′ UTR. The general time reversible (GTR) model was used for a 154 base-pair dataset of the 3′ UTR fragment. The numbers above each branch represent posterior probability (PP) obtained in the Bayesian Index (1.00). DENV2 (AF038403, KM204118) and DENV4 (AY947539, KR011349) reference strains were used as outgroups. Branches in pink represents DENV3 sequences and DENV3 study-identified sequences are highlighted in pink. Branches in blue represents DENV1 sequences and DENV1 study-identified sequences are highlighted in blue.

The phylogenetic analysis with the Bayesian tree revealed clustering of all the domain II, 3′ UTR DENV3 isolates and DENV1 isolates into two separate basal clades (Figure 4 and Figure S5). Within the DENV3 basal clade, the study-identified DENV3 sequences (only one human-derived and one mosquito-derived isolate are represented) segregated into a distinct clade (posterior probability (PP) 0.99 and distinct mean branch length 0.0043). Furthermore, considering the so far isolated DENV3 Sri Lankan isolates, a distinct segregation of the Sri Lankan isolates, AY585848 and FJ88273 (both isolated in 1993) was evident by a separate clade (PP 0.94 and distinct mean branch length of 0.0039). A similar deviation of the Sri Lankan isolate GQ252674 (isolated in 1997) was also observed (distinct mean branch length 0.0037).

The phylogenetic analysis revealed the segregation of study-identified DENV1 (only one human-derived and one mosquito-derived isolate are represented) sequences of domain II, 3′ UTR with DENV1 genotype V strains (JN903581, JQ922548 isolated from 2009–2011 in India, AY732474, AY732474, AY62084 isolated before 1993 in Thailand, Singapore), IV (U88535 year of isolation unknown) and Sri Lankan isolate HQ891314 (isolated in 2009) (PP 0.87) and then further clustering into a distinct clade (PP 0.95 and branch length 0.0034) (Figure 4 and Figure S3).

The clustering of all the other Sri Lankan isolates (from 2009–2014) with DENV1 genotype I and genotype V isolates with Asian origin (isolated from 1990–2010 from Thailand, Bangladesh, China) was also observed (PP 0.63). However, no clear clustering of genotypes I–V was observed among the domain II, 3′ UTR-based classification.

**Figure 5:**
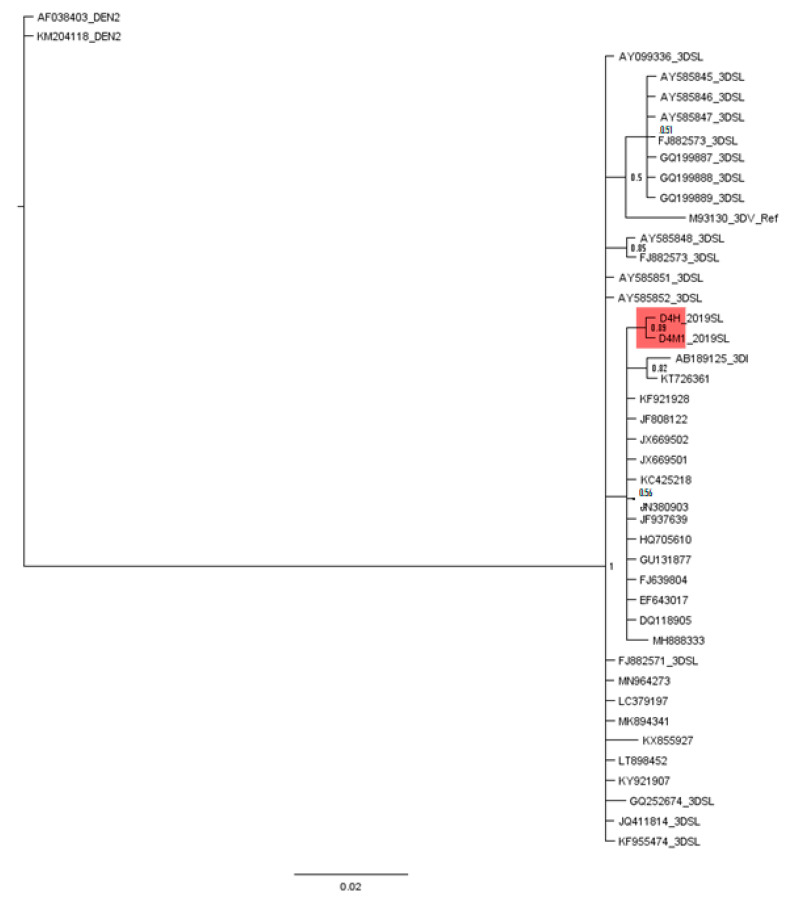
Calibrated maximum-clade-credibility tree for DENV3. DENV3 study-identified sequences, Sri Lankan isolates and similar sequences revealed from BLAST, based on 3′ UTR domain II fragment. The GTR model was used for a 45 base-pair dataset of the 3′ UTR fragment. The numbers above each branch represent the PP obtained in the Bayesian Index (1.00). DENV2 reference strains (AF038403, KM204118) were used as outgroups. DENV3 study-identified sequences are highlighted in pink.

The DENV3 3′ UTR domain II region from this study found homology with a set of sequences shown in Table S3 (Query cover-100%, E value 2 × 10^−91^, percentage identity 99.47%) which resulted from the BLAST, which were isolated from Latin American countries including Brazil, Nicaragua, Venezuela and Paraguay. The phylogenetic analysis using the Bayesian tree (Figure 5) revealed that all so far reported Sri Lankan DENV3 isolates and study-identified DENV3 sequences clustered in the same basal cluster with the Latin American DENV3 sequences resulted from the BLAST.

**Figure 6:**
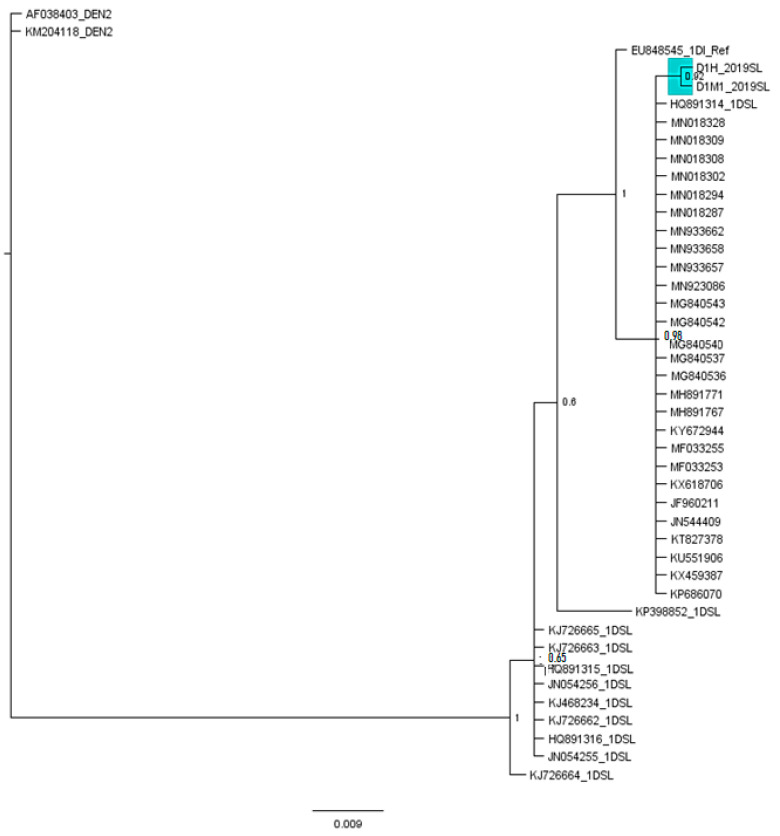
Calibrated maximum-clade-credibility tree for DENV1. DENV1 study-identified sequences, Sri Lankan isolates and similar sequences revealed from BLAST, based on 3′ UTR, domain II fragment. The GTR model was used for a 59 base-pair dataset of the 3′ UTR fragment. The numbers above each branch represent the PP obtained in the Bayesian Index (1.00). DENV2 reference strains (AF038403, KM204118) were used as outgroups. DENV1 study-identified sequences are highlighted in blue.

The DENV1 3′ UTR domain II region from this study found homology with a set of sequences shown in Table S4 (Query cover-100%, E value 5 × 10^−93^, percentage identity 99.48%) as per the BLAST result. These strains had an Asian origin and were identical in the selected nucleotide region. However, the phylogenetic analysis with the similar sequences and Sri Lankan isolates revealed (Figure 6) that, the 2019 DENV1 sequences identified in the study were more related to the 2009 Sri Lankan isolate HQ891314, than the Sri Lankan isolates recorded earlier. Furthermore, the 2019 DENV1 sequence was more related to the other homologues Asian isolates than the Sri Lankan isolates.

### 3.4. Secondary Structure Analysis

**Figure 7:**
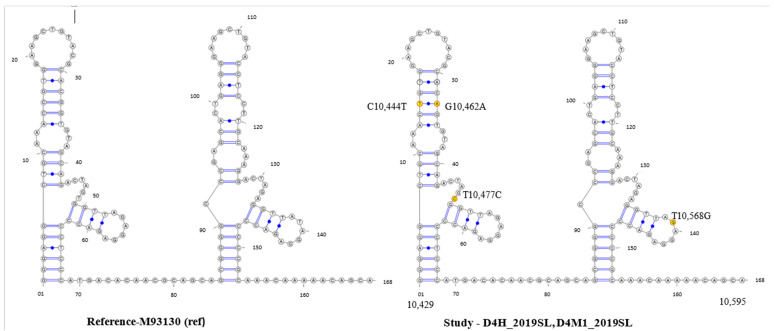
RNAalifold-predicted consensus secondary structures of RNA alignments of DENV3. DENV3 DB1 and DB2 of the 3′ UTR region, reference strain (M93130) and DENV3 sequences derived from human serum (D4H_2019SL, D5H_2019SL) and mosquito tissues (D1M1_2019SL-D1M5_2019SL and D3M1_2019SL-D3M6_2019SL) in this study, implemented by the Vienna RNA package. Mutations C10444T, G10462A and T10477C were observed on the DB1 structure while the mutation T10568C was observed on the DB2 structure. Nucleotide variations are indicated in yellow.

**Figure 8:**
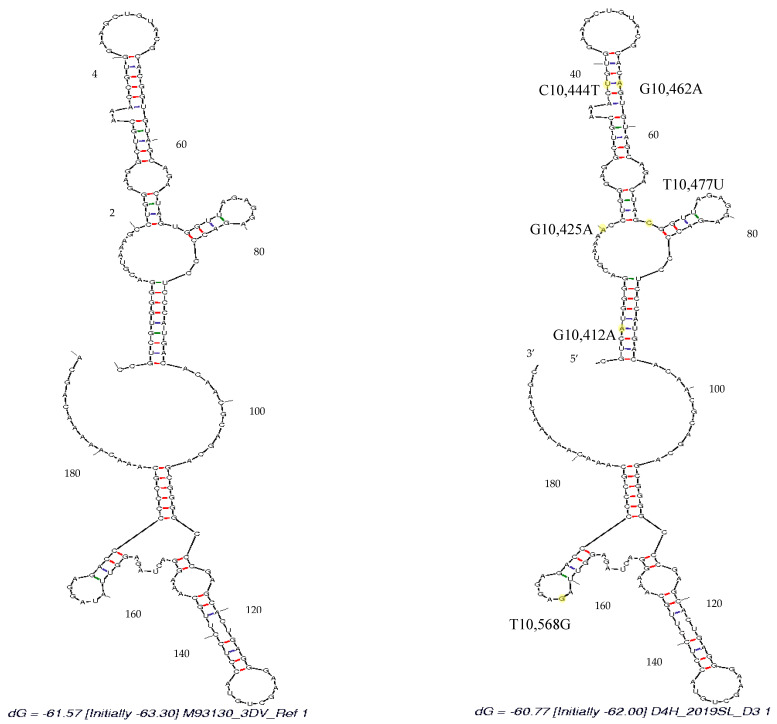
Mfold-predicted minimum free energy (MFE) secondary structures for RNA alignments of DENV3. DENV3, domain II of 3′ UTR region, reference strain (M93130) and DENV3 sequences identified from the study. Nucleotide variations are indicated in yellow.

DENV3 sequences derived in the study revealed mutations on both DB1 and DB2 structures respective to the DENV3 reference sequence (Figure 1, Table S6). According to the Mfold- and RNAfold-predicted secondary structures, both the reference- and study-identified DENV3 sequences assumed similar secondary structures (Figure 7 and Figure 8). Table S6 shows that the secondary structure predictions using the Mfold and RNAfold programs revealed distinct DB1 and DB2 secondary structures for the DENV3 sequences of the study sequences compared to other Sri Lankan isolates. The similar DB1 and DB2 structures were observed in the study-identified sequences of DENV3 and reference DENV3 genotype II and genotype V isolates. Despite the nucleotide variation, all the Sri Lankan DENV3 isolates shared similar DB1 and DB2 secondary structures (Table S6). Lesser variation was observed among the DB2 secondary structures and two predominant structures for domain II, 3′ UTR were observed among all the DENV3 isolates considered.

**Figure 9:**
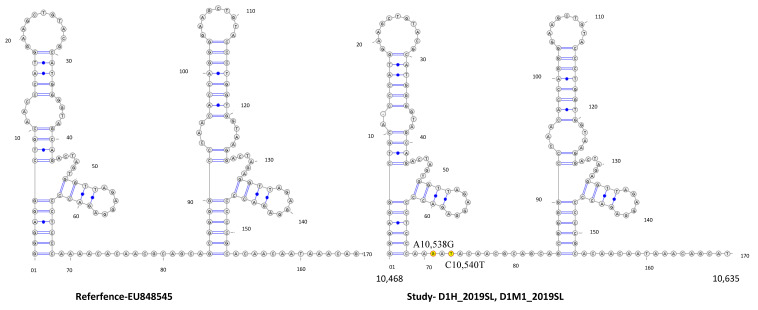
RNAalifold-predicted consensus structures for RNA alignments of DENV1. DENV1, DB1 and DB2 of 3′ UTR region, reference strain (EU848545) and DENV1 sequences derived from human serum (D1H_2019SL, D3H_2019SL) and mosquito tissues (D4M1_2019SL-D4M7_2019SL, D5M1_2019SL-D5M4_2019SL), implemented by the Vienna RNA package. Mutations C10538G and C1540T were observed on the DB1 structure while no mutations were observed on the DB2 structure. Nucleotide variations are indicated in yellow.

**Figure 10:**
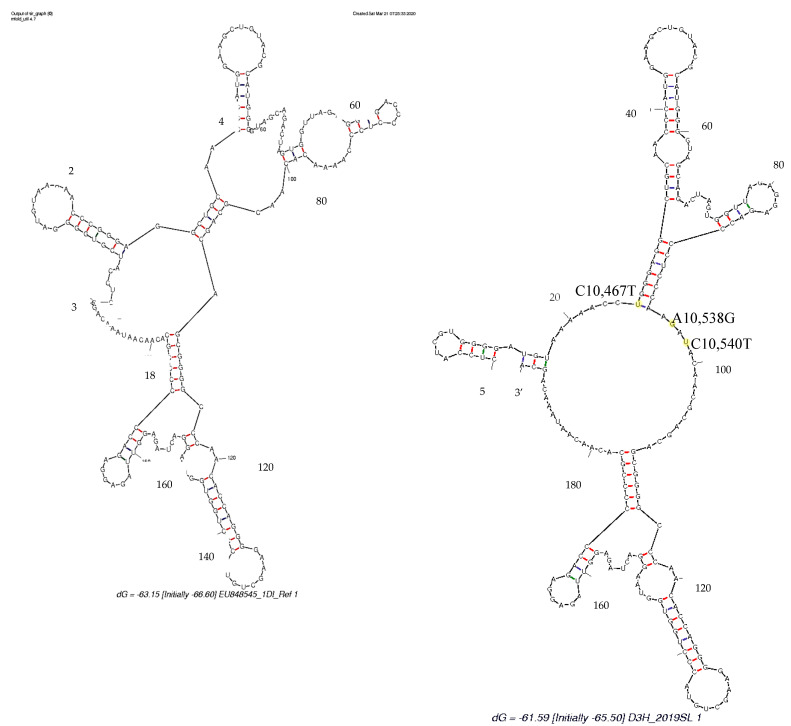
Mfold-predicted MFE secondary structures for RNA alignments of DENV1. DENV1, DB1 and DBII of 3′ UTR region, reference strain (EU848545) and DENV1 sequences identified from the study. Nucleotide variations are indicated in yellow.

Study-identified DENV1 sequences revealed mutations on DB1 structure respective to the DENV1 reference sequence (Figure 2, Table S7). According to the Mfold- and RNAfold-predicted secondary structures, the reference- and study-isolated DENV1 sequences assumed similar DB2 structures but different DB1 structures (Figure 9 and Figure 10). Table S7 shows that the secondary structure predictions using the Mfold and RNAfold programs revealed similar DB1 and DB2 secondary structures for all the Sri Lankan isolates including the study-isolated DENV1 sequences. Lesser variation was observed among the DB2 sequences of all DENV1 isolates. Although a low level of structural variation was observed according to Mfold predictions, more structural conservation was observed in DENV1 isolates according to the RNAfold predictions. One predominant structure for domain II, 3′ UTR with lesser or no variation was observed among all DENV1 isolates according to both Mfold and RNAfold predictions.

## 4. Discussion

The DENV genotypes show a characteristic geographical distribution, implying competitive advantage for individual genotypes in different environments [1]. The utilization of 3′ UTR in genetic characterization is less common among the DENV research. [14,17]. Therefore, to have an insight on the genetic variation of the DENV genotypes, this study focused on the moderately conserved domain II, 3′ UTR, which also houses the conserved RCS2 and CS2 regions and two DB structures.

DENV, being an RNA virus, exhibits exceptional genetic variability, mainly due to the intrinsically high rate of mutation associated with RNA-dependent RNA polymerase [54]. The sequence variation of the DENV genome during the human to mosquito host switch has been investigated in many studies [30,55,56,57,58,59]. In the study of de Borba et al. [24], the significance of the variability of host adaptation has been studied. Their study found mutations in both DB regions of DENV populations with increased frequency from the first to second generation (F1 to F2) of infected *Ae. aegypti* vector mosquitoes. It has been concluded that the mutations are positively selected in adult mosquitoes that affect DB2 structures resulting in enhanced RNA replication in mosquito cells. One of the objectives of the current study was to examine the nucleotide variation of the domain II, 3′ UTR during the host switch in human- and mosquito-derived sequences respective to the infected serotype. No such sequence variations were observed in the domain II, 3′ UTR of DENV3 and DENV1 sequences identified in the current study and the human- and mosquito-derived sequences in the domain II, 3′ UTR were identical in their respective serotypes (Table 1). However, adaptive mutations within the minor DENV populations within each host are known to be obscured by wild-type sequences and only revealed once they become dominant in the virus population and deep sequencing experiments are warranted to inference further in this regard [60]. The present study confirmed the reportedly high conservation of the RCS2 and CS2 regions in domain II, 3′ UTR as shown by de Castro et al. [55] in is his study of human- and mosquito-derived samples [55]. Furthermore, one of the Brazilian mosquito-derived isolates from his study JN38344 showed homology with the study isolates of DENV3 (Table S3). Similarly, Sessions et al. [31] did not find any mutations in the 5′ or 3′ UTR that has been identified in recent studies, except for a single position in the 3′ UTR in two isolates out of the twelve DENV1 isolates. The consensus changes detected in that study were positioned within the UTR between DB1 and DB2 in the two isolates. As discussed by Sessions et al. [31], the reason for the observed stability in this region is unclear. The studies Liu et al. [56], de Borba et al. [24] and Villordo et al. [30] were primarily conducted in cell lines with DENV2 and to a lesser extent with DENV3 [55]. As most of the host adaptation studies have been carried out with low sample numbers, further inferences in this regard are warranted with extensive studies of mosquito adaptation.

The sequence analysis of the DENV3 domain II, 3′ UTR sequences derived from the human and respective laboratory-infected mosquitoes revealed nucleotide differences compared to that of the so far reported Sri Lankan isolates (Figure 1 and Figure S1). The phylogenetic analysis with the Bayesian tree revealed clustering of the domain II, 3′ UTR DENV3 sequences identified in the study into a distinct clade (PP 0.99 and distinct mean branch length 0.0043) (Figure 4 and Figure S2). Discrete SNVs were observed among the study-identified sequences of DENV3 and the respective regions of so far reported Sri Lankan isolates (Figure 1). The mutations C10,444T, T10,477C (DB1 region) and T10,568G (DB2 region) were commonly shared between Sri Lankan isolates and DENV3 sequences identified in this study, while mutation G10,425 and G10,462A (DB1 region) was seen only with study-identified sequences. The mutations G10,412A which was observed in the spacer region just before the DB1 were not shared by the other reported Sri Lankan isolates. Furthermore, except in few DENV3 genotype II isolates, the mutation G10,412A was rarely observed among the DENV3 isolates (Figure 1).

With respect to the sequence variations, DB1 and DB2 structural variations were observed among all the DENV3 sequences considered (Table S6). The secondary structure predictions using the Mfold and RNAfold programs revealed distinct DB1 and DB2 secondary structures for the DENV3 sequences identified in the study compared to other Sri Lankan isolates investigated earlier. Despite the nucleotide variation observed, all the Sri Lankan isolates shared a similar DB1 and DB2 secondary structures. Compared to the SNV observed in Sri Lankan isolates, the discrete SNV observed in DENV sequences from the study (Figure 1 and Figure S1), (both within DB regions and in-between spacer regions) may have attributed to the structural differences observed in the secondary structures.

DENV3 viruses isolated before and after the emergence of dengue hemorrhagic fever (DHF) in 1989 in Sri Lanka belonged to two distinct clades (DENV3 genotype IIIA and IIIB, respectively) [12,13]. The stepwise increase in cases after 2000 has been accompanied by the appearance of another clade of the DENV3 genotype III strain that has replaced the clade IIIB [15]. The DENV3 serotype was known to be responsible for the 2002 and 2004 epidemics [61]. In 2008, Silva et al. [14] studied the 5′ and 3′ UTRs of 15 serum-derived DENV-3 genotype III isolates from Sri Lanka, Nicaragua and Martinique and found sequence variability in the 3′ UTRs. Silva et al. [14] were able to reproduce the clustering of Messer et al. [13] in the phylogenetic analysis based on whole genome sequences. However, in his phylogenetic analysis based on 3′ UTR, the isolate AY585848 (isolated in 1993) which fell outside of the group B clade based on structural gene analysis of Messer et al. [13], segregated with the group B isolates. In the current study, the phylogenetic analysis revealed the segregation of all Sri Lankan isolates of DENV3 in the same basal clade, except for the study sequences of DENV3 (PP 0.99 and distinct mean branch length 0.0043) and the 1993 isolates, AY585848, FJ88273 (PP of 0.94 and mean branch length of 0.0039) which showed distinct segregations in separate clades (Figure 4 and Figure S3). Furthermore, the Sri Lankan isolate GQ252674 (isolated in 1997) displayed a distinct branching (distinct mean branch length 0.0037) (Figure 4 and Figure S5). The previously recorded DENV3 Sri Lankan strains isolated after 2000 were not considered in the phylogenetic study due to the unavailability of whole or corresponding 3′ UTRs. However, the DENV3, genotype III, group A segregation observed in Messer et al. [12] and Silva et al. [14] was not observed in the phylogenetic classification of the current study based on the domain II, 3′ UTR (Figure 4 and Figure S5). All genotype III viruses are known to be closely related, with distinct phylogenetic groups associated with moderate or severe disease [13]. Due to various limitations, the phenotypical presentation of the study isolates was not considered. However, phylogenetic studies with the phenotypic appearances for each subtype are warranted and would bridge the knowledge gaps in future studies.

DENV3 was known to have caused unexpected epidemics of DHF in Sri Lanka, East Africa, and Latin America over the past two decades. The virus evolution using a phylogenetic approach has been investigated and found that isolates from these geographically distinct epidemics were closely related and belonged to DENV3, subtype III, which originated in the Indian subcontinent [13]. The emergence of DHF in Sri Lanka in 1989 has correlated with the appearance of a new DENV3, subtype III variant, which likely has spread from the Indian subcontinent into Africa in the 1980s and from Africa into Latin America in the mid-1990s [13]. Silva et al. [14] in his study with 3′ UTR, suggested that the Martinique isolates described by Peyrefitte et al. [62] and the Nicaraguan DENV3 samples were related to the Sri Lankan genotype IIIB DENV3. Furthermore, the clustering of the Latin American/Caribbean isolates with the Sri Lankan genotype IIIB DENV3 in his phylogenetic analysis supported the proposed common East African origin for all these strains confirming the use of the 3′ UTR for molecular epidemiologic studies of DENV3 [14]. The DENV3 3′ UTR domain II region sequences from this study was found to be homologous to a set of sequences shown in Table S3 which resulted from the BLAST. Interestingly, these homologous sequences were isolated from Latin American countries including Brazil, Nicaragua, Venezuela and Paraguay. The phylogenetic analysis using the Bayesian tree (Figure 5) revealed that all so far reported Sri Lankan DENV3 isolates and study-identified DENV3 sequences clustered in the same basal cluster with the Latin American DENV3 sequences resulted from the BLAST. As reported by Messer et al. [13], a single introduction and eventual diversification of the virus population from the founding strain is indicated by the clustering of Latin America/Caribbean isolates. The most recent isolate has been reported from Nicaragua in 2010 (Table S3). The exact mode of persistent transportation of DENV3 strains from Latin America to Sri Lanka cannot be exactly speculated. However, the environmental and socioeconomic factors responsible for the transport and persistence in Sri Lankan context should be further examined.

The DENV1 3′ UTR domain II sequences derived from the human and respective laboratory-infected mosquito vectors revealed nucleotide differences compared to that of the so far reported Sri Lankan sequences according to the sequence analysis (Figure S2 and Figure 2). The phylogenetic analysis with the Bayesian tree (Figure 1 and Figure 2) revealed the segregation of study-identified DENV1 sequences with DENV1 genotype IV, V and Sri Lankan isolate HQ891314 (PP 0.86) and then further clustering into a distinct clade (PP 0.95 and distinct mean branch length 0.0034). The clustering of all the other Sri Lankan isolates with DENV1 genotype I and V isolates (PP 0.63) was also observed. Discrete SNV was observed (Figure 2) in the DENV1 sequences identified in the study and the so far reported Sri Lankan isolates except in HQ891314 (isolated in 2009) which shared two of the three mutations. As in all the other reported Sri Lankan isolates, no mutations were observed in the DB2 regions of the study-identified sequences of DENV1. The C10,467T mutation which was observed in the DB1 region was commonly shared by all Sri Lankan isolates except for the HQ891314 and most of the other DENV1 isolates of genotype I–V. The DENV1 sequences identified in the study and the Sri Lankan isolate HQ891314 shared the mutations A10,538G and C10,540T in the spacer region following the DB1. Although these mutations were commonly observed among all the genotypes of DENV1, co-occurrence of both mutations were rarely observed except in the genotype IV and V. However, despite the SNV in DB regions and in between spacer regions, all the Sri Lankan DENV1 isolates and study-identified sequences of DENV1 shared the same DB1 and DB2 secondary structures according to RNAfold predictions (Table S7). However, differences were observed in the DB1 structure of the HQ891314 isolate according to Mfold predictions (Table S7).

The earliest isolates of DENV1 from Sri Lanka isolated in 1983 and 1984 belonged to the South Pacific genotype III [15]. More recent isolates obtained during 1997–2004 were belonged to the Africa/America genotype IV, indicating that at some point between the early 1980s and the mid-1990s, there was a DENV1 genotype shift. The DENV1 genotype with an Asian origin introduced before the 2009 epidemic which appeared to be responsible for the 2009 epidemic of Dengue and Dengue hemorrhagic fever has been reported [45], and since then DENV1 has remained as the predominant serotype in the country [46]. According to the prevailing phylogenetic evidence, the DENV1 strain from the 2009 epidemic has continued to circulate within the population and caused severe disease in the epidemic of 2012 [46]. The DENV1 3′ UTR domain II region identified from this study was found to be homologous to a set of sequences shown in Table S4 as per the BLAST results. All these homologous sequences had an Asian origin and were identical in the selected region to DENV1 study sequences. The phylogenetic analysis with these homologous sequences and Sri Lankan isolates revealed that the 2019, study-identified DENV1 sequences were more related to these homologous sets of sequences with the Asian origin than to the so far reported DENV1 Sri Lankan sequences, except for the 2009 Sri Lankan isolate HQ891314, which also segregated into the same clade (Figure 6). The most recently reported isolate out of these homologous Asian sequences were from Singapore (2016) and India (2017). It has been shown that the virus corresponding to the 3′ UTR domain II has circulated and persisted in Asian countries such as China, Singapore and India since 2000 (Table S4). Since continuous transportations to the above countries occur for commercial, cultural and religious requirements, it is unclear why this isolate was not reported earlier. However, the phenotypical presentation of the 2019 DENV1 strain together with other strains should be further studied with its whole genome composition to make further inferences. de Borba et al. [24] examined the evolutionary relationships between the duplicated RNA structures in different DENV serotypes and found that DB1 and DB2 elements from different DENV serotypes were more alike between them than DB1 and DB2 from the same serotype. The same observation was made during the secondary structure predictions for DENV3 and DENV1 sequences (Tables S6 and S7). As suggested by de Borba et al. [24], this fact supported a divergent evolutionary path and specialization of each structure after the duplication.

It was observed that DB2 displays more sequence variation than DB1 within the same serotype in both DENV3 and DENV1 sequences. de Borba et al. [24] examined the sequence conservation of the two DB structures among genotypes of DENV2 serotype and observed a higher sequence variability of DB2 among its genotypes. As elaborated by de Borba et al. [24], the observations suggest that the two paralogues RNA elements are under different selective pressures in nature, which could be associated with the selection observed in mosquitoes [24].

The fact that mutations in the flaviviral 3′ UTR could affect sfRNA production and its affinity to host proteins, which are necessary for successful viral replication, is well established [20]. In the case of DENV, the two SLs in domain II are found to be functionally coupled for sfRNA generation and the sequence variation acquired in mosquito adaptation was found to be detrimental for sfRNA1 formation [55]. de Borba et al. [24] identified sequence variations in one of the two DB structures in DENVs that were isolated after passage in mosquitoes, which resembled earlier experimental results in SLs. Furthermore, the deletion of both DB elements was near lethal for viral replication in both hosts, and the deletion of DB2 was found to be advantageous for mosquito infection [24]. It is known that shorter variants, sfRNA3 and sfRNA4, were positively selected in mosquito cell lines [5]. It has been demonstrated that shorter sfRNAs are produced as the result of stalling degradation just upstream of two DB structures [63]. It can be hypothesized that the point mutations on DB or resulting secondary structures of DBs could also play a role in shorter sfRNA formation. Furthermore, except the highly conserved domain III region, the DB region (majorly the DB2 region) is the most represented region among all sfRNA species. However, the structural consequences and impact of the substitutions in this region on viral fitness have yet to be experimentally defined.

It is known that a high similarity between centroid and MFE structures indicate a reliable prediction [64]. The MFE and centroid predictions for the study isolates of both DENV3 and DENV1 resulted in similar structures (Tables S6 and S7). However, variations were also observed to a lesser extent in DB2 structures. The predominant types of DB1 and DB2 structures were clearly observed among both DENV3 and DENV1 isolates and further studies are warranted to interpret the relationship with viral features.

Maintaining structural integrity in DB structures contributes to sfRNA production and the epidemiological fitness of the virus [32]. The predominant secondary structures of DBs revealed in the study clearly demonstrated the structural integrity among the DENV3 and DENV1 strains studied (Tables S6 and S7). Amidst the number of SNVs revealed, DB structures were often accompanied by compensatory mutations to maintain structural integrity [20]. However, it is also known that these functionally important RNA structures can be disrupted by SNVs [65]. Recently, single-nucleotide-induced changes of RNA conformations has been studied and it was recommended that SNVs could be used as a powerful tool to study the impact on structural changes of RNAs [66]. RNA secondary structure is a result of a complex network of base-pairing and stacking interactions [67]. To observe a large conformational change in RNA, the mutation must not only disrupt an existing base-pair, but also favor a completely alternative base-pairing network. The functional consequences of structure disruption depend on whether the affected region is involved in important regulatory interactions. In certain cases, small local changes in RNA structures may have functional consequences [65,68]. The two RNA structure prediction approaches have revealed certain SNVs that have disrupted the structure, in both the DB and the spacer regions adjoining the DB structures (Figure 1, Figure 2 and Figure 3, Tables S6 and S7). However, predicting exactly which SNV will alter the secondary structure remains challenging and the fact should be further examined. Phenotypic presentations respective to the secondary structures should be further investigated throughout all the genotypes and the SNVs responsible should be identified in future studies of the genetic manipulation of DENV.

## 5. Conclusions

This study examined the genetic variations of the domain II, 3′ UTR of DENV3 and DENV1 circulated during the 2019 dengue outbreak in Sri Lanka. The study elaborated on the stability and the conserved nature of the domain II, 3′ UTR region during the host switch. Distinct nucleotide sequence variations in the domain II, 3′ UTR were observed between the study-identified sequences and the so far recorded Sri Lankan isolates. DENV genotype classifications with respect to domain II, 3′ UTR revealed distinct segregations of the study isolates. Unique SNVs in 3′ UTR have been revealed in the study and the phenotypical presentation corresponding to the variations should be further examined to correlate with the disease presentation. The structure and the role of DBs have been a novel area of interest and the contribution to sfRNA production must be further examined. Furthermore, the study recommends future research in examining and analyzing the genetic variation of Sri Lankan isolates along with the intra-host genetic variation among native vector mosquitoes. 

## Figures and Tables

**Table 1:** The RT-PCR and sequence-based serotype results for human- and mosquito-derived samples. DENV: dengue virus.

RT-PCR Results on Human Serum (*n* = 5)	DENV Serotype According to Sequence Analysis	Human-Derived DENV Samples	RT-PCR Results on Mosquito Tissues	Mosquito-Derived DENV Samples
+	DENV 1	D1H_2019SL	+(5/12)	D1M1_2019SL-D1M5-2019SL
+	DENV 1	D3H_2019SL	+(6/10)	D3M1_2019SL-D3M6_2019SL
+	DENV 3	D4H_2019SL	+(7/11)	D4M1_2019SL-D4M7_2019SL
+	DENV 3	D5H_2019SL	+(4/9)	D5M1_2019SL-D5M4_2019SL
+	DENV 3	D6H_2019SL	-	-

## Data Availability

Data is contained within the article or supplementary material. The data presented in this study are available in Table S3.

## Supplementary Materials

The following are available online at https://www.mdpi.com/1999-4915/13/3/421/s1. Figure S1: Nucleotide variation analysis of the domain II, 3′ UTR of DENV3. Figure S2: Nucleotide variation analysis of the domain II, 3′ UTR of DENV1. Figure S3: Gel photograph showing the amplified products of the DENV 3′ UTR region of dengue-confirmed human serum samples. Figure S4: Gel photograph showing the amplified products of the DENV 3′ UTR region of dengue positive mosquito tissue samples. Figure S5: Calibrated maximum-clade-credibility tree for DENV3 and DENV1, domain II, 3′ UTR. Table S1: Source of DENV3 virus isolates used for sequence analysis. Table S2: Source of DENV1 virus isolates used for sequence analysis. Table S3: Homologous GenBank DENV sequences respective to domain II 3′ UTR of DENV3 study-identified sequences. Table S4: Homologous GenBank DENV sequences respective to domain II 3′ UTR of DENV1 study-identified sequences. Table S5: GenBank accession numbers obtained for the study sequences. Table S6: Mfold- and RNAfold-predicted secondary structures for RNA alignments of DENV3, domain II region sequences identified in the study, Sri Lankan isolates and DENV3 genotypes. Table S7: Mfold- and RNAfold-predicted secondary structures for RNA alignments of DENV3, domain II region sequences identified in the study, Sri Lankan isolates and DENV1 genotypes.
