# Supplementary material for: Genetic Variation in the Domain II, 3′ Untranslated Region of Human and Mosquito Derived Dengue Virus Strains in Sri Lanka
Source: Viruses. 2021 Mar 5;13(3):421. doi: 10.3390/v13030421 (PMC8001906; doi:10.3390/v13030421)
Supplement: Supplementary file 1 [file viruses-13-00421-s001.zip › Supplimentry files/Supplimentry figures/Figure caption- Figure S5.pdf]

Figure S5. Calibrated maximum-clade-credibility tree for DENV3 and DENV1. DENV3 and DENV1 study identified sequences, so far reported Sri Lankan isolates and reference genotype strains, based on Domain II, 3′ UTR. GTR model was used for a 154 base pair dataset of the 3′ UTR fragment. The numbers above each branch represent mean branch lengths obtained in the Bayesian Index (1.00). DENV2 (AF038403, KM204118) and DENV4 (AY947539, KR011349) reference strains were used as outgroups.
