# Supplementary material for: Genetic Variation in the Domain II, 3′ Untranslated Region of Human and Mosquito Derived Dengue Virus Strains in Sri Lanka
Source: Viruses. 2021 Mar 5;13(3):421. doi: 10.3390/v13030421 (PMC8001906; doi:10.3390/v13030421)
Supplement: Supplementary file 1 [file viruses-13-00421-s001.zip › Supplimentry files/Supplimentry figures/Figure S1.pdf]

```
(10,406)                                   “A2”                                          RCS2
                       10         20         30         40         50         60         70         80         90        100
                 ....|....| ....|....| ....|....| ....|....| ....|....| ....|....| ....|....| ....|....| ....|....| ....|....|
M93130_3DV_Ref   TCCGTCGTGG GGACGTAAAG -CCTGGGAGG CTGCAAACCG TGGAAGCTGT ACGCACGGTG TAGCAGACTA GTGGTTAGAG GAGACCCCTC CCATGACACA
D4H_2019SL_D3    ......A... .........A -......... ........T. .......... ......A... .......... .C........ .......... ..........
D4M1_2019SL_D3   ......A... .........A -......... ........T. .......... ......A... .......... .C........ .......... ..........
AY099336_3DSL    .......... .........A -......... ........T. .......... .......... .......... .C........ .......... ..........
AY585845_3DSL    .......... .......... -......... ........T. .......... .......... .......... .C........ .......... ..........
AY585846_3DSL    .......... .......... -......... ........T. .......... .......... .......... .C........ .......... ..........
GQ199889_3DSL    .......... .......... -......... ........T. .......... .......... .......... .C........ .......... ..........
FJ882571_3DSL    .......... .........A -......... ........T. .......... .......... .......... .C........ .......... ..........
AY585847_3DSL    .......... .......... -......... ........T. .......... .......... .......... .C........ .......... ..........
GQ199888_3DSL    .......... .......... -......... ........T. .......... .......... .......... .C........ .......... ..........
FJ882573_3DSL    .......... .........A -......... ........T. .......... .......... .......... .C........ .......... ..........
GQ199887_3DSL    .......... .......... -......... ........T. .......... .......... .......... .C........ .......... ..........
FJ882574_3DSL    .......... .......... -......... ........T. .......... .......... .......... .C........ .......... ..........
GQ252674_3DSL    .......... .........A -......... ........T. .......... .......... .......... .C........ .......... ....A.....
AY585848_3DSL    .......... .........A -......... ........T. .......... .......... .......... .C........ .......... ..........
AY585851_3DSL    .......... .........A -......... ........T. .......... .......... .......... .C........ .......... ..........
FJ882572_3DSL    .......... .......... -......... ........T. .......... .......... .......... .C........ .......... ..........
AY585852_3DSL    .......... .........A -......... ........T. .......... .......... .......... .C........ .......... ..........
KF955474_3DSL    .......... .........A -......... ........T. .......... .......... .......... .C........ .......... ..........
JQ411814_3DSL    .......... .........A -......... ........T. .......... .......... .......... .C........ .......... ..........
AY648961_3DI     .......... .........A -......... ........T. .......... .......... .......... .C........ .......... ..........
AY744681_3DI     .......... .........A -......... .......... .......... .......... .......... .......... .......... ..........
AB189125_3DI     .......... .........A -......... ........T. .......... ......A... .......... .C........ .......... ......T...
AY858037_3DI     .......... .........A -......... ........T. .......... ......A... .......... .......... .......... ..........
AY858043_3DI     .......... .........A -......... ........T. .......... .......... .......... .C........ .......... ..........
AY858046_3DI     .......... .......... -......... ........T. .......... .......... .......... .C........ .......... ..........
AY858038_3DI     .......... .........A -......... ........T. .......... .......... .......... .C........ .......... ..........
EU081223_3DI     .......... .........A -......... ........T. .......... .......... .......... .C........ .......... ..........
DQ401690_3DI     .......... .........A -......... ........T. .......... ......A... .......... .C........ .......... ..........
AY858041_3DI     .......... .........A -......... ........T. .......... .......... .......... .C........ .......... ..........
AB189128_3DI     .......... .........A -......... ........T. .......... .......... .......... .C........ .......... ..........
KX380839_3DI     .......... .........A -......... ........T. .......... .......... .......... .C........ .......... ..........
AY858046_3DIF    .......... .........A -......... ........T. .......... .......... .......... .C........ .......... ..........
AY676352_3DII    .......... .......... -......... .......... .......... .......... .......... .C........ .......... ..........
DQ675522_3DII    .......... .......... -......... .......... .......... .......... .......... .C........ .......... ..........
DQ863638_3DII    .......... .......... -......... ........T. .......... .......... .......... .C........ .......... ..........
EU482453_3DII    .......... .......... -......... .......... .......... .......... .......... .C........ .......... .........G
AY876494_3DII    .......... .......... -......... .......... .......... .......... .......... .C........ .......... ..........
FJ744734_3DII    .......... .......... -......... .......... .......... .......... .......... .C........ .......... ..........
EU482459_3DII    .......... .......... -......... .......... .......... .......... .......... .C........ .......... .........G
FJ744728_3DII    .......... .......... -......... .......... .......... .......... .......... .C........ .......... ..........
EU482461_3DII    .......... .......... -......... .......... .......... .......... .......... .C........ .......... .........G
AY676350_3DII    .......... .......... -......... .......... .......... .......... .......... .C........ .......... ..........
FJ461337_3DII    .......... .......... -......... .......... .......... .......... .......... .C........ .......... .........G
FJ687448_3DII    .......... .......... -......... .......... .......... .......... .......... .C........ .......... ..........
FJ744726_3DII    .......... .......... -......... .......... .......... .......... .......... .C........ .......... ..........
KF955457_3DII    .......... .......... -......... .......... .......... .......... .......... .C........ .......... .........G
AY496873_3DII    .......... .......... -......... .......... .......... .......... .......... .C........ .......... .........G
KJ622197_3DII    ......A... .........A -......... .......... .......... .......... .......... .C........ .......... ..........
KC261634_3DII    .......... .........A -..C...... .......... .......... .......... .......... .C........ .......... ....A.....
KF824903_3DII    .......... .........A -......... .......... .......... .......... .......... .C........ .......... ..........
KF824902_3DII    .......... .........A -......... .......... .......... .......... .......... .C........ .......... ..........
KJ737429_3DII    .......... .......... -......... .......... .......... .......... .......... .C........ .......... ..........
KY849772_3DII    .......... .........A -......... .......... .......... .......... .......... .C........ .......... ..........
KY849771_3DII    .......... .........A -......... .......... .......... .......... .......... .C........ .......... ..........
KY849774_3DII    .......... .........A -......... .......... .......... .......... .......... .C........ .......... ..........
KY849770_3DII    .......... .........A -......... .......... .......... .......... .......... .C........ .......... ..........
KY849773_3DII    .......... .........A -......... .......... .......... .......... .......... .C........ .......... ..........
KR296743_3DII    .......... .........A -......... .......... .......... .......... .......... .C........ .......... ..........
KY849769_3DII    .......... .........A -......... .......... .......... .......... .......... .C........ .......... ..........
KY849775_3DII    .......... .........A -......... .......... .......... .......... .......... .C........ .......... ..........
KJ622198_3DII    ......A... .........A -......... .......... .......... .......... .......... .C........ .......... ..........
GQ868571_3DIII   .......... .........A -......... ........T. .......... .......... .......... .C........ .......... ..........
EU529691_3DIII   .......... .........A -......... ........T. .......... .......... .......... .C........ .......... ..........
AY662691_3DIII   .......... .........A -......... ........T. .......... .......... .......... .C........ .......... ..........
AY770511_3DIII   .......... .........A -......... ........T. .......... .......... .......... .C........ .......... ..........
GQ466079_3DIII   .......... .........A -......... ........T. .......... .......... .......... .C........ .......... ..........
AY099336_3DIII   .......... .........A -......... ........T. .......... .......... .......... .C........ .......... ..........
GU131872_3DIII   .......... .........A -......... ........T. .......... ......A... .......... .C........ .......... ..........
EU081182_3DIII   .......... .........A -......... ......G.T. .......... .......... .......... .C........ .......... ..........
HQ705618_3DIII   .......... .........A -......... ........T. .......... ......A... .......... .C........ .......... ..........
JF504679_3DIII   .......... .........A -......... ........T. .......... .......... .......... .C........ .......... ....C.....
FJ182013_3DIII   .......... .........A -......... ........T. .......... .......... .......... .C........ .......... ..........
FJ882573_3DIII   .......... .........A -......... ........T. .......... .......... .......... .C........ .......... ..........
FJ898440_3DIII   .......... .........A -......... ........T. .......... .......... .......... .C........ .......... ..........
JQ922557_3DIII   .......... .........A -......... ........T. .......... .......... .......... .C........ .......... ..........
KF041254_3DIII   .......... .........A -......... ........T. .......... .......... .......... .C........ .......... ..........
KF041255_3DIII   .......... .........A -......... ........T. .......... .......... .......... .C........ .......... ..........
KF041257_3DIII   .......... .........A -......... ........T. .......... .......... .......... .C........ .......... ..........
KF041258_3DIII   .......... .........A -......... ........T. .......... .......... .......... .C........ .......... ..........
KF041259_3DIII   .......... .........A -......... ........T. .......... .......... .......... .C........ .......... ..........
KF954945_3DIII   .......... .........A -......... ........T. .......... ......A... .......... .......... .......... ..........
KF954947_3DIII   .......... .........A -......... ........T. .......... ......A... .......... .......... .......... ..........
KJ643590_3DIII   .......... .........A -......... ........T. .......... ......A... .......... .C........ .......... ..........
JF808129_3DIII   .......... .........A A......... ........T. .......... ......A... .......... .......... .......... ..........
KF954946_3DIII   .......... .........A -......... ........T. .......... ......A... .......... .......... .......... ..........
KT726350_3DIII   .......... .........A -......... ........T. .......... ......A... .......... .C........ .......... ..........
MF370226_3DIII   .......... .........A A......... ........T. .......... .......... .......... .C........ .......... ..........
KU509282_3DV     .......... .......... -......... .......... .......... .......... .......... .......... .......... ..........
JN697379_3DV     .......... .......... -......... .......... .......... .......... .......... .......... .......... ..........
KU050695_3DV     .......... .......... -......... .......... .......... .......... .......... .......... .......... ..........
```

```
                                                              "A3"              CS2
                          110        120        130        140        150        160        170        180        190
                    ....|....| ....|....| ....|....| ....|....| ....|....| ....|....| ....|....| ....|....| ....|....| .
M93130_3DV_Ref      ACGCAGCAGC GGGGCCCGAG CACTGAGGGA AGCTGTACCT CCTTGCAAAG GACTAGAGGT TATAGGAGAC CCCCCGCAAA CAAAAACAGC A
D4H_2019SL_D3       .......... .......... .......... .......... .......... .......... ..G....... .......... .......... .
D4M1_2019SL_D3      .......... .......... .......... .......... .......... .......... ..G....... .......... .......... .
AY099336_3DSL       .......... .......... .......... .......... .......... .......... ..G....... .......... T......... .
AY585845_3DSL       .......... .......... .......... .......... .......... .......... ..G....... .......... T......... .
AY585846_3DSL       .......... .......... .......... .......... .......... .......... ..G....... .......... T......... .
GQ199889_3DSL       .......... .......... .......... .......... .......... .......... ..G....... .......... T......... .
FJ882571_3DSL       .......... .......... .......... .......... .......... .......... ..G....... .......... .......... .
AY585847_3DSL       .......... .......... .......... .......... .......... .......... ..G....... .......... T......... .
GQ199888_3DSL       .......... .......... .......... .......... .......... .......... ..G....... .......... T......... .
FJ882573_3DSL       .......... .......... .G........ .......... .......... .......... ..G....... .......... T......... .
GQ199887_3DSL       .......... .......... .......... .......... .......... .......... ..G....... .......... T......... .
FJ882574_3DSL       .......... .......... .......... .......... .......... .......... ..G....... .......... T......... .
GQ252674_3DSL       .......... .......... .......... .......... .......... .......... ..G....... .......... .......... .
AY585848_3DSL       .......... .......... .G........ .......... .......... .......... ..G....... .......... T......... .
AY585851_3DSL       .......... .......... .......... .......... .......... .......... ..G....... .......... T......... .
FJ882572_3DSL       .......... .......... .......... .......... .......... .......... ..G....... .......... T......... .
AY585852_3DSL       .......... .......... .......... .......... .......... .......... ..G....... .......... T......... .
KF955474_3DSL       .......... .......... .......... .......... .......... .......... ..G....... .......... .......... .
JQ411814_3DSL       .......... .......... .......... .......... .......... .......... ..G....... .......... .......... .
AY648961_3DI        .......... .......... .T........ .......... .......... .......... ..G....... .......... T......... .
AY744681_3DI        .......... .......... .......... .......... ..C....... .......... ..G....... .......... T......... .
AB189125_3DI        .......... .......... .......... .......... .......... .......... ..G....... .......... T......... .
AY858037_3DI        .......... .......... .......... .......... .......... .......... ..G....... .......... T......... .
AY858043_3DI        .......... .......... .......... .......... .......... .......... ..G....... .......... T......... .
AY858046_3DI        .......... .......... .......... .......... .......... .......... ..G....... .......... .......... .
AY858038_3DI        .......... .......... .......... .......... .......... .......... ..G....... .......... .......... .
EU081223_3DI        .......... .......... .......... .......... .......... .......... ..G....... .......... .......... .
DQ401690_3DI        .......... .......... .......... .......... .......... .......... ..G....... .......... T......... .
AY858041_3DI        .......... .......... .......... .......... .......... .......... ..G....... .......... .......... .
AB189128_3DI        .......... .......... .......... .......... .......... .......... ..G....... .......... .......... .
KX380839_3DI        .......... .......... .......... .......... .......... .......... ..G....... .......... G......... .
AY858046_3DIF       .......... .......... .......... .......... .......... .......... ..G....... .......... .......... .
AY676352_3DII       .......... .......... .......... .......... .......... .......... ..G....... .......... T......... .
DQ675522_3DII       .......... .......... .......... .......... .......... .......... ..G....... .......... T......... .
DQ863638_3DII       .......... .......... .......... .......... .......... .......... ..G....... .......... .......... .
EU482453_3DII       .......... .......... .......... .......... .......... .......... ..G....... .......... T......... .
AY876494_3DII       .......... .......... .......... .......... .......... .......... ..G....... .......... T......... .
FJ744734_3DII       .......... .......... .......... .......... .......... .......... ..G....... .......... T......... .
EU482459_3DII       .......... .......... .......... .......... .......... .......... ..G....... .......... T......... .
FJ744728_3DII       .......... .......... .......... .......... .......... .......... ..G....... .......... T......... .
EU482461_3DII       .......... .......... .......... .......... .......... .......... ..G....... .......... T......... .
AY676350_3DII       .......... .......... .......... .......... .......... .......... ..G....... .......... T......... .
FJ461337_3DII       .......... .......... .......... .......... .......... .......... ..G....... .......... .......... .
FJ687448_3DII       .......... .......... .......... .......... .......... .......... ..G....... .......... T......... .
FJ744726_3DII       .......... .......... .......... .......... .......... .......... ..G....... .......... T......... .
KF955457_3DII       .......... .......... .......... .......... .......... .......... ..G....... .......... .......... .
AY496873_3DII       .......... .......... .......... .......... .......... .......... ..G....... .......... T......... .
KJ622197_3DII       .......... .......... .......... .......... .T........ .......... ..GT...... .......... T......... .
KC261634_3DII       .......... .......... .......... .......... .......... .......... ..G....... ......T... T......... .
KF824903_3DII       .......... .......... .......... .......... .T........ .......... ..GT...... .......... T......... .
KF824902_3DII       .......... .......... .......... .......... .T........ .......... ..GT...... .......... T......... .
KJ737429_3DII       .......... .......... .......... .......... .......... .......... ..G....... .......... T......... .
KY849772_3DII       .......... .......... .......... .......... .T........ .......... ..G....... .......... T......... .
KY849771_3DII       .......... .......... .......... .......... .T........ .......... ..G....... .......... T......... .
KY849774_3DII       .......... .......... .......... .......... .T........ .......... ..G....... .......... T......... .
KY849770_3DII       .......... .......... .......... .......... .T........ .......... ..G....... .......... T......... .
KY849773_3DII       .......... .......... .......... .......... .T........ .......... ..G....... .......... T......... .
KR296743_3DII       .......... .......... .......... .......... .T........ .......... ..GT...... .......... T......... .
KY849769_3DII       .......... .......... .......... .......... .T........ .......... ..G....... .......... T......... .
KY849775_3DII       .......... .......... .......... .......... .T........ .......... ..G....... .......... T......... .
KJ622198_3DII       .......... .......... .......... .......... .T........ .......... ..GT...... .......... T......... .
GQ868571_3DIII      .......... .......... .......... .......... .......... .......... ..G....... .......... T......... .
EU529691_3DIII      .........T .......... .......... .......... .......... .......... ..G....... .......... T......... .
AY662691_3DIII      .......... .......... .......... .....C.... .......... .......... ..G....... .......... TG........ .
AY770511_3DIII      .......... .......... .......... .......... .......... .......... ..G....... .......... .......... .
GQ466079_3DIII      .......... .......... .......... .......... .......... .......... ..G....... .......... .......... .
AY099336_3DIII      .......... .......... .......... .......... .......... .......... ..G....... .......... T......... .
GU131872_3DIII      .......... .......... .......... .......... ..C....... .......... ..G....... .......... .......... .
EU081182_3DIII      .......... .......... .......... .......... .......... .......... ..G....... .......... T......... .
HQ705618_3DIII      .......... .......... .......... .......... .......... .......... ..G....... .......... T......... .
JF504679_3DIII      .......... .......... .......... .......... .......... .......... ..G....... .......... .......... .
FJ182013_3DIII      .......... .......... .......... .......... .......... .......... ..G....... .......... T......... .
FJ882573_3DIII      .......... .......... .G........ .......... .......... .......... ..G....... .......... T......... .
FJ898440_3DIII      .......... .......... .......... .......... .....T.... .......... ..G....... .......... T......... .
JQ922557_3DIII      .......... .......... .......... .......... .......... .......... ..G....... .......... .......... .
KF041254_3DIII      .......... .......... .......... .......... .......... .......... ..G....... .......... .......... .
KF041255_3DIII      .......... .......... .......... .......... .......... .......... ..G....... .......... .......... .
KF041257_3DIII      .......... .......... .......... .......... .......... .......... ..G....... .......... .......... .
KF041258_3DIII      .......... .......... .......... .......... .......... .......... ..G....... .......... .......... .
KF041259_3DIII      .......... .......... .......... .......... .......... .......... ..G....... .......... .......... .
KF954945_3DIII      .......... .......... .......... .......... .......... .......... ..G....... .......... .......... .
KF954947_3DIII      .......... .......... .......... .......... .......... .......... ..G....... .......... .......... .
KJ643590_3DIII      .......... .......... .......... .......... .......... .......... ..G....... .......... T......... .
JF808129_3DIII      .......... .......... .......... .......... .......... .......... ..G....... .......... .......... .
KF954946_3DIII      .......... .......... .......... .......... .......... .......... ..G....... .......... .......... .
KT726350_3DIII      .......... .......... .......... .......... .......... .......... ..G....... .......... T......... .
MF370226_3DIII      .......... .......... .......... .......... .......... .......... ..G....... .......... .......... .
KU509282_3DV        .......... .......... .......... .......... .......... .......... .......... .......... .......... .
JN697379_3DV        .......... .......... .......... .......... .......... .......... .......... .......... .......... .
KU050695_3DV        .......... .......... .......... .......... .......... .......... .......... .......... .......... .
```

Figure S1. Nucleotide variation analysis of the Domain II, 3'UTR of DENV3

Nucleotide sequence alignment of the Domain II, 3'UTR of; DENV3 human (only D4H_2019SL is represented) and mosquito (only D4M1_2019SL is represented) derived sequences identified in the study, DENV3 isolates of Sri Lanka and DENV3 GI-V genotypes beginning with the 10,406-nucleotide position. Nucleotide position indicated in parentheses is according to the complete genome of D3-H87 reference strain (GenBank accession number M93130). Dots (.) indicate identity to the reference DENV3 strain and dashes (-) indicate gaps in the alignment. The conserved sequence regions (RCS2 and CS2) reported by Hahn et al., (1987) are highlighted in gray. Conserved areas A2-A3 are underlined (Shurtleff et al., 2001).
