# Supplementary material for: Genetic Variation in the Domain II, 3′ Untranslated Region of Human and Mosquito Derived Dengue Virus Strains in Sri Lanka
Source: Viruses. 2021 Mar 5;13(3):421. doi: 10.3390/v13030421 (PMC8001906; doi:10.3390/v13030421)
Supplement: Supplementary file 1 [file viruses-13-00421-s001.zip › Supplimentry files/Supplimentry figures/Figure S2.pdf]

```
                                                                      "A2"                 RCS2
            (10,443)        10         20         30         40         50         60         70
                      ....|....| ....|....| ....|....| ....|....| ....|....| ....|....| ....|....|
EU848545_1DI_Ref      CTCCATCGTG GGGATGTAAA AACCCGGGAG GCTGCAACCC ATGGAAGCTG TACGCATGGG GTAGCAGACT
D1H_2019SL            .......... .......... ....T..... .......... .......... .......... ..........
D1M1_2019SL           .......... .......... ....T..... .......... .......... .......... ..........
KJ726665_1DSL         .......... .......... ....T..... .......... .......... .......... ..........
KJ726663_1DSL         .......... .......... ....T..... .......... .......... .......... ..........
HQ891315_1DSL         .......... .......... ....T..... .......... .......... .......... ..........
JN054256_1DSL         .......... .......... ....T..... .......... .......... .......... .  .........
KJ468234_1DSL         .......... .......... ....T..... .......... .......... .......... ..........
KP398852_1DSL         .......... .......... ....T..... .......... .......... .......... ..........
KJ726664_1DSL         .......... .......... ....T..... .......... .......... .......... ..........
HQ891314_1DSL         .......... .......... .......... .......... .......... .......... ..........
KJ726662_1DSL         .......... .......... ....T..... .......... .......... .......... ..........
HQ891316_1DSL         .......... .......... ....T..... .......... .......... .......... ..........
JN054255_1DSL         .......... .......... ....T..... .......... .......... .......... ..........
AF309641_1DI          .......... .......... ....T..... .......... .......... ..GCG..... ..........
AB074760_1DI          .......... .......... .......... .......... .......... .......... ..........
AF350498_1DI          .......... .......... ....T..... .......... .......... .......... ..........
AF298807_1DI          .......... .......... .......... .......... .......... .......... ..........
AY726555_1DI          .......... .......... ....T..... .......... .......... .......... ..........
AY732477_1DI          .......... .......... ....T..... .......... .......... .......... ..........
AY732479_1DI          .......... .......... ....T..... .......... .......... .......... ..........
AY732480_1DI          .......... .......... ....T..... .......... .......... .......... ..........
AY732483_1DI          .......... .......... ....T..... ......A... .......... .......... ..........
AY835999_1DI          .......... .......... ....T..... .......... .......... .......... ..........
DQ285561_1DI          .......... .......... ....T..... ......T... .......... .......... ..........
EU081226_1DI          .......... .......... ....T..... .......... .......... .......... ..........
HG316481_1DI          .......... .......... ....T..... .......... .......... .......... ..........
HG316482_1DI          .......... .......... ....T..... .......... .......... .......... ..........
HQ891316_1DI          .......... .......... ....T..... .......... .......... .......... ..........
JN638340_1DI          .......... .......... ....T..... .......... .......... .......... ..........
JN638342_1DI          .......... .......... ....T..... .......... .......... .......... ..........
JN638344_1DI          .......... .......... ....T..... .......... .......... .......... ..........
U88537_1DI            .......... .......... .......... .......... .......... .......... ..........
KJ726662_1DI          .......... .......... ....T..... .......... .......... .......... ..........
EF457905_1DIII        .......... .......... .......... .......... .......... .......... ..........
DQ672564_1DIV         .......... .......... .......... ......A... .......... .......... ..........
EU863650_1DIV         .......... .......... ....T..... ......A... .......... .......... ..........
FJ196842_1DIV         .......... .......... ....T..... ......G... .......... .......... ..........
FJ196845_1DIV         .......... .......... .G........ ......A... .......... .......... ..........
U88535_1DIV           .......... .......... .......... .......... .......... .......... ..........
AF226687_1DV          .......... .......... .......... .......... .......... .......... ..........
AF514889_1DV          .......... .......... .......... .......... .......... .......... ..........
AY732474_1DV          .......... .......... .......... .......... .......... .......... ..........
AY732476_1DV          .......... .......... .......... .......... .......... .......... ..........
AF514883_1DV          .......... .......... .......... .......... .......... .......... ..........
AF298808_1DV          .......... .......... ....T..... .......... .......... .......... ..........
AY762084_1DV          .......... .......... .......... .......... .......... .......... ..........
EU081258_1DV          .......... .......... .......... .......... .......... .......... ..........
GU131962_1DV          .......... .......... ....T..... .......... .......... .......... ..........
HQ332182_1DV          .......... .......... .......... .......... .......... .......... ..........
JN903579_1DV          .......... .......... .......... ......A... .......... .......... ..........
JN903581_1DV          .......... .......... .......... .......... .......... .......... ..........
JQ915080_1DV          .......... .......... ....T..... ......A... .......... .......... ..........
JQ922544_1DV          .......... .......... .......... .......... .......... .......... ..........
JQ922546_1DV          .......... .......... .......... .......... .......... .......... ..........
JQ922548_1DV          .......... .......... .......... .......... .......... .......... ..........
EU596501_1DV          .......... .......... .......... .......... .......... .......... ..........
KC692512_1DV          .......... .......... .......... .......... .......... .......... ..........
KF289072_1DV          .......... .......... .......... .......... .......... .......... ..........
M87512_1DV            ..T....... .......... ....T..... .......... .......... .......... ..........
```

```
                   RCS2                 "A2"                                        "A3"
                            80         90         100        110        120        130        140
                   ....|....| ....|....| ....|....| ....|....| ....|....| ....|....| ....|....|
EU848545_1DI_Ref   AGTGGTTAGA GGAGACCCCT CCCAAAACAC AACGCAGCAG CGGGGCCCAA CACCAGGGGA AGCTGTACCC
D1H_2019SL         .......... .......... .....G.T.. .......... .......... .......... ..........
D1M1_2019SL        .......... .......... .....G.T.. .......... .....  ..... .......... ..........
KJ726665_1DSL      .......... .......... ...G.....T .......... .......... .......... ..........
KJ726663_1DSL      .......... .......... ...G.....T .......... .......... .......... ..........
HQ891315_1DSL      .......... .......... ...G.....T .......... .......... .......... ..........
JN054256_1DSL      .......... .......... ...G.....T .......... .......... .......... ..........
KJ468234_1DSL      .......... .......... ...G.....T .......... .......... .......... ..........
KP398852_1DSL      .......... .......... ...G.....T .......... .......... .......... ..........
KJ726664_1DSL      .......... .......... ...G.....T .......... .......... .......... ..........
HQ891314_1DSL      .......... .......... .....G.T.. .......... .......... .......... ..........
KJ726662_1DSL      .......... .......... ...G.....T .......... .......... .......... ..........
HQ891316_1DSL      .......... .......... ...G.....T .......... .......... .......... ..........
JN054255_1DSL      .......... .......... ...G.....T .......... .......... .......... ..........
AF309641_1DI       .......... .......... ...G.....T .......... .......... .......... ..........
AB074760_1DI       .......  ... .......... .......... .......... .......... .......... ..........
AF350498_1DI       .......... .......... .......... .......... .......... .......... ..........
AF298807_1DI       .......... .......... .........T .......... .......... .......... ..........
AY726555_1DI       .......... .......... .......... .......... .......... .......... ..........
AY732477_1DI       .......... .......... .......... .......... .......... .......... ..........
AY732479_1DI       .......... .......... ...G...T.T .......... .......... .......... ..........
AY732480_1DI       .......... .......... .........T .......... .......... .......... .......T..
AY732483_1DI       .......... .......... .....G.... .......... .......... .......... ..........
AY835999_1DI       .......... .......... ...G.....T .......... .......... .......... ..........
DQ285561_1DI       .......... .......... .....G.... .......... .......... ........A. .......T..
EU081226_1DI       .......... .......... ...G...... .......... .......... .......... ..........
HG316481_1DI       .......... .......... ...G...... .......... .......... .......... ..........
HG316482_1DI       .......... .......... ...G.....T .......... .......... .......... ..........
HQ891316_1DI       .......... .......... ...G.....T .......... .......... .......... ..........
JN638340_1DI       .......... .......... .........T .......... .......... .......... .......T..
JN638342_1DI       .......... .......... .......... .......... .......... .......... ..........
JN638344_1DI       .......... .......... .......... .......... .......... .......... ..........
U88537_1DI         .......... .......... ...T.G...T .......... .......... .....T.... .........T
KJ726662_1DI       .......... .......... ...G.....T .......... .......... .......... ..........
EF457905_1DIII     .......... .......... .....G...T .......... .......... .......... ..........
DQ672564_1DIV      .......... .......... .....G.... .......... .......... .......... ..........
EU863650_1DIV      .......... .......... .......... .......... .......... ....T...A. ..........
FJ196842_1DIV      .......... .......... .....G.... .......... .........G .......... ..........
FJ196845_1DIV      ..C....... .......... .....G.... .......... .......... .......... ..........
U88535_1DIV        .......... .......... .....G.T.. .......... .......... .......... ..........
AF226687_1DV       .......... .......... .....G...T .......... .......... .......... .........T
AF514889_1DV       .......... .......... .....G.... .......... .......... .......... .........T
AY732474_1DV       .......... .......... .....G.T.. .......... .......... .......... ..........
AY732476_1DV       .......... .......... .....G.T.. .......... .......... .......... ..........
AF514883_1DV       .......... .......... ...T.G...T .......... .......... .....T.... .........T
AF298808_1DV       .......... .......... .......... .......... .......... ...T...... T.........
AY762084_1DV       .......... .......... .....G.T.. .......... .......... .......... ..........
EU081258_1DV       ..C....... .......... .........T .......... .......... .......... .........T
GU131962_1DV       .......... .......... ...G.....T .......... .......... .......... ..........
HQ332182_1DV       .......... .......... ...C.G.... .......... .......... .......... .........T
JN903579_1DV       .......... .......... .....G.... .......... .......... .......... ..........
JN903581_1DV       .......... .......... .....G.T.. .......... .......... .......... ..........
JQ915080_1DV       .......... .......... .......... .......... .......... ....T...A. ..........
JQ922544_1DV       .......... .......... .....G...T .......... .......... .......... .........T
JQ922546_1DV       .......... .......... .....G...T .......... .......... .......... ..........
JQ922548_1DV       .......... .......... .....G.TG. .......... .......... .......... .........T
EU596501_1DV       .......... .......... ...T.G...T .......... .......... .......... .........T
KC692512_1DV       .......... .......... ...T.G...T .......... .......... .......... .........T
KF289072_1DV       .......... .......... .....G.T.. .......... .......... .......... ..........
M87512_1DV         .......... .......... .........T .......... .......... .......... .......T..
```

```
                                              CS2                 "A3"
                          150         160         170         180         190
                    ....|....| ....|....| ....|....| ....|....| ....|....| ...
EU848545_1DI_Ref    TGGTGGTAAG GACTAGAGGT TAGAGGAGAC CCCCCGCACA ACAATAAACA GCA
D1H_2019SL          .......... .......... .......... .......... .......... ...
D1M1_2019SL         .......... .......... .......... .......... .......... ...
KJ726665_1DSL       .......... .......... .......... ........T. .......... ...
KJ726663_1DSL       .......... .......... .......... ........T. .......... ...
HQ891315_1DSL       .......... .......... .......... ........T. .......... ...
JN054256_1DSL       .......... .......... .......... ........T. .......... ...
KJ468234_1DSL       .......... .......... .......... ........T. .......... ...
KP398852_1DSL       .......... .......... .......... .......... .T.......G AA-
KJ726664_1DSL       .......... .....  .....  .......... .........T.  ....A..... ...
HQ891314_1DSL       .......... .......... .......... .......... .......... ...
KJ726662_1DSL       .......... .......... .......... ........T. .......... ...
HQ891316_1DSL       .......... .......... .......... ........T. .......... ...
JN054255_1DSL       .......... .......... .......... ........T. .......... ...
AF309641_1DI        .......... .......... .......... ........T. .......... ...
AB074760_1DI        .......... .......... .......... ........T. .......... ...
AF350498_1DI        .......... .......... .......... ........T. .......... ...
AF298807_1DI        .......... .......... .......... .......... ....C..... ...
AY726555_1DI        .......... .......... .......... ........T. .......... ...
AY732477_1DI        .......... .......... .......... ........T. .......... ...
AY732479_1DI        .......... .......... .......... ........T. .......... ...
AY732480_1DI        .......... .......... .......... ........T. .......... ...
AY732483_1DI        .......... .......... .......... ........T. .......... ...
AY835999_1DI        .......... .......... .......... ........T. .......... ...
DQ285561_1DI        .......... .......... .......... ........T. .......... ...
EU081226_1DI        .......... .......... .......... ........T. .......... ...
HG316481_1DI        .......... .......... .......... ........T. ....A..... ...
HG316482_1DI        .......... .......... .......... .......TT. .......... ...
HQ891316_1DI        .......... .......... .......... ........T. .......... ...
JN638340_1DI        .......... .......... .......... ........T. .....G.... ...
JN638342_1DI        .......... .......... .......... ........T. .......... ...
JN638344_1DI        ...C...... .......... .......... ........T. .......... ...
U88537_1DI          .......... .......... .......... .......... ....C..... ...
KJ726662_1DI        .......... .......... .......... ........T. .......... ...
EF457905_1DIII      .......... .......... .......... .......... .......... ...
DQ672564_1DIV       .......... .......... .......... ........T. ....C..... ...
EU863650_1DIV       .......... .......... .......... ........T. .......... ...
FJ196842_1DIV       .......... .......... .......... .......... ....C..... ...
FJ196845_1DIV       .......... .......... .......... .......... .......... ...
U88535_1DIV         .......... .......... .......... .......... .......... ...
AF226687_1DV        .......... .......... .......... .......... ....C..... ...
AF514889_1DV        .......... .......... .......... .......... ....C..... ...
AY732474_1DV        .......... .......... .......... .......... .......... ...
AY732476_1DV        .......... .......... .......... .......... .......... ...
AF514883_1DV        .......... .......... .......... ........T. ....C..... ...
AF298808_1DV        .......... .......... .......... ........T. .......... ...
AY762084_1DV        .......... .......... .......... .......... .......... ...
EU081258_1DV        .......... .......... .......... .......... ....C..... ...
GU131962_1DV        .......... .......... .......... ........T. .......... ...
HQ332182_1DV        .......... .......... .......... .......... ....C..... ...
JN903579_1DV        .......... .......... .......... .......... ....C..... ...
JN903581_1DV        .......... .......... .......... .......... .......... ...
JQ915080_1DV        .......... .......... .......... ........T. .......... ...
JQ922544_1DV        .......... .......... .......... ........A. ...CC..... ...
JQ922546_1DV        .......... .......... .TT....... ........T. ....C..... ...
JQ922548_1DV        .......... .......... .......... ........T. .T........ ...
EU596501_1DV        .......... .......... .......... .......... ....C..... ...
KC692512_1DV        .......... .......... .......... .......... ....C..... ...
KF289072_1DV        .......... .......... .......... .......... .......... ...
M87512_1DV          .......... .......... .......... ....G...T. .......... ...
```

Figure S2. Nucleotide variation analysis of the Domain II, 3’UTR of DENV1

Nucleotide sequence alignment of the Domain II, 3’UTR of DENV1 human (only D1H2019SL is represented) and mosquito (Only D1M1_2019SL is represented) derived sequences identified in the study, DENV1 isolates of Sri Lanka and DENV1 GI-V genotypes beginning with the 10,443 position. Nucleotide position indicated in parentheses is according to the complete genome of EU8845 reference strain (GenBank accession number EU8485). Dots (.) indicate identity to the reference DENV3 strain and dashes (-) indicate gaps in the alignment. The conserved sequence regions (RCS2 and CS2) reported by Hahn et al., (1987) are highlighted in gray. Conserved areas A2-A3 are underlined (Shurtleff et al., 2001).
