# Supplementary material for: Genetic Variation in the Domain II, 3′ Untranslated Region of Human and Mosquito Derived Dengue Virus Strains in Sri Lanka
Source: Viruses. 2021 Mar 5;13(3):421. doi: 10.3390/v13030421 (PMC8001906; doi:10.3390/v13030421)
Supplement: Supplementary file 1 [file viruses-13-00421-s001.zip › Supplimentry files/Supplimentry figures/Figure S3.pdf]

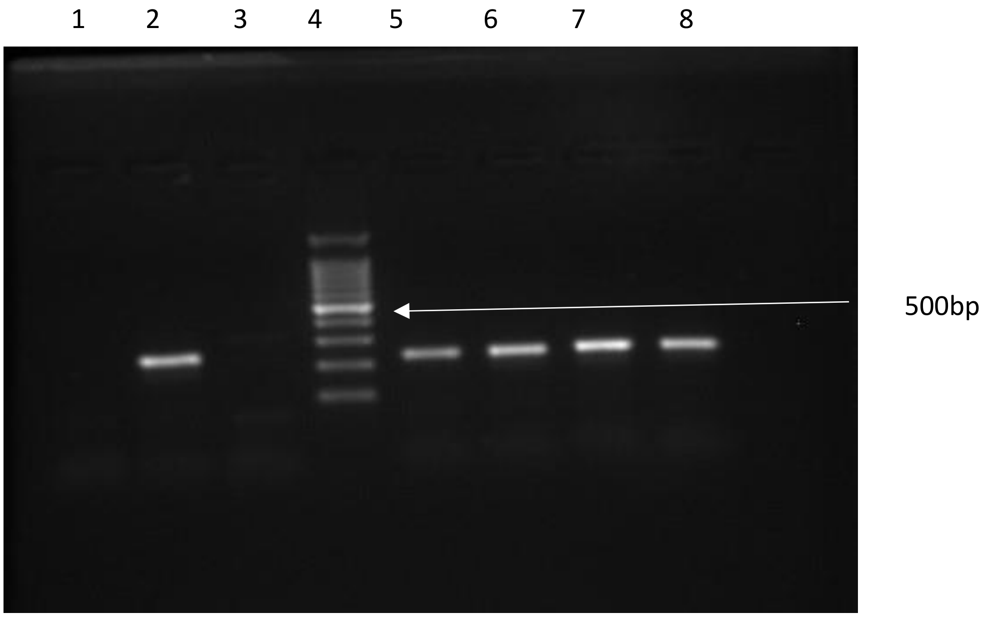

Figure S3. Gel photograph showing the amplified products of the DENV 3'UTR region of dengue confirmed human serum samples. Lane 1: Negative control, Lane 4: 100 bp ladder. Lanes 2,3, 5-8: PCR products for DENV 3'UTR region (lanes 2, 5, 6, 7, 8 showing positive results (D1H_2019SL, D3H_2019SL, D4H_2019SL, D5H_2019SL) and lane 3 showing a negative result (D2H_2019SL)).
