# Supplementary material for: Genetic Variation in the Domain II, 3′ Untranslated Region of Human and Mosquito Derived Dengue Virus Strains in Sri Lanka
Source: Viruses. 2021 Mar 5;13(3):421. doi: 10.3390/v13030421 (PMC8001906; doi:10.3390/v13030421)
Supplement: Supplementary file 1 [file viruses-13-00421-s001.zip › Supplimentry files/Supplimentry figures/Figure S4.pdf]

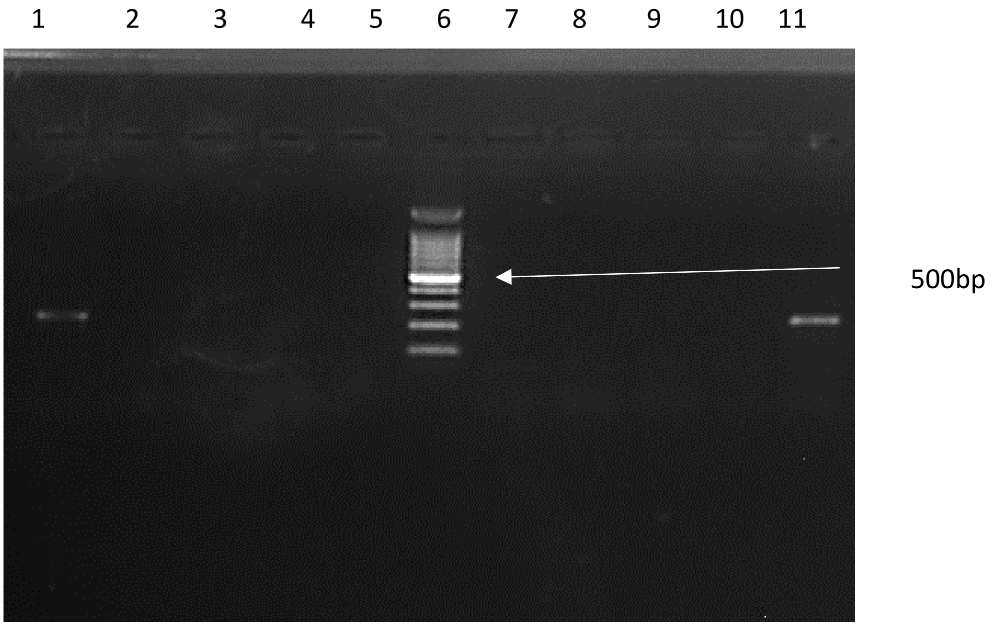

Figure S4. Gel photograph showing the amplified products of the DENV 3’UTR region of mosquito tissue samples. Lane 2: Negative control, Lane 6: 100 bp ladder. Lanes 1, 3-4, 7-11: PCR products for DENV 3’UTR region, (lanes, 1 and 11 showing positive results, (only two positive results are shown here) and lanes 3-4 and 7-10 showing negative results, for mosquito tissue samples from inoculate D1H_2019SL).
