# Supplementary material for: Genetic Variation in the Domain II, 3′ Untranslated Region of Human and Mosquito Derived Dengue Virus Strains in Sri Lanka
Source: Viruses. 2021 Mar 5;13(3):421. doi: 10.3390/v13030421 (PMC8001906; doi:10.3390/v13030421)
Supplement: Supplementary file 1 [file viruses-13-00421-s001.zip › Supplimentry files/Supplimentry tables/Table S1.docx]

Table S1: Source of DENV3 virus isolates used for sequence analysis.

|  | **Isolate Label** | **Genotype** | **Year of Collection** | **Gen Bank accession number** | **Country** |
| --- | --- | --- | --- | --- | --- |
| 1 | M93130_3DV_Ref | v | 1956 | M93130 | Philippines |
| 2 | D4H_2019SL_D3 |  | 2019 |  | Sri Lanka |
| 3 | D4M1_2019SL_D3 |  | 2019 |  | Sri Lanka |
| 4 | D4M2_2019SL_D3 |  | 2019 |  | Sri Lanka |
| 5 | D4M3_2019SL_D3 |  | 2019 |  | Sri Lanka |
| 6 | D4M4_2019SL_D3 |  | 2019 |  | Sri Lanka |
| 7 | D4M5_2019SL_D3 |  | 2019 |  | Sri Lanka |
| 8 | D4M6_2019SL_D3 |  | 2019 |  | Sri Lanka |
| 9 | D4M7_2019SL_D3 |  | 2019 |  | Sri Lanka |
| 10 | D5H_2019SL_D3 |  | 2019 |  | Sri Lanka |
| 11 | D5M1_2019SL_D3 |  | 2019 |  | Sri Lanka |
| 12 | D5M2_2019SL_D3 |  | 2019 |  | Sri Lanka |
| 13 | D5M3_2019SL_D3 |  | 2019 |  | Sri Lanka |
| 14 | D5M4_2019SL_D3 |  | 2019 |  | Sri Lanka |
| 15 | D5M_2019SL_D3 |  | 2019 |  | Sri Lanka |
| 16 | AY099336_3DSL | III | 2000 | AY099336 | Sri Lanka |
| 17 | AY585845_3DSL | III | 1983 | AY585845 | Sri Lanka |
| 18 | AY585846_3DSL | III | 1989 | AY585846 | Sri Lanka |
| 19 | GQ199889_3DSL | III | 1983 | GQ199889 | Sri Lanka |
| 20 | FJ882571_3DSL | III | 1989 | FJ882571 | Sri Lanka |
| 21 | AY585847_3DSL | III | 1989 | AY585847 | Sri Lanka |
| 22 | GQ199888_3DSL | III | 1983 | GQ199888 | Sri Lanka |
| 23 | FJ882573_3DSL | III | 1993 | FJ882573 | Sri Lanka |
| 24 | GQ199887_3DSL | III | 1983 | GQ199887 | Sri Lanka |
| 25 | FJ882574_3DSL | III | 1985 | FJ882574 | Sri Lanka |
| 26 | GQ252674_3DSL | III | 1997 | GQ252674 | Sri Lanka |
| 27 | AY585848_3DSL | III |  | AY585848 | Sri Lanka |
| 28 | AY585851_3DSL | III |  | AY585851 | Sri Lanka |
| 29 | FJ882572_3DSL | III | 1989 | FJ882572 | Sri Lanka |
| 30 | AY585852_3DSL | III |  | AY585852 | Sri Lanka |
| 31 | KF955474_3DSL | III | 1989 | KF955474 | Sri Lanka |
| 32 | JQ411814_3DSL | III | 1989 | JQ411814 | Sri Lanka |
| 33 | AY648961_3DI | I |  | AY648961 | Indonesia |
| 34 | AY744681_3DI | I | 1990 | AY744681 | French Polynesia |
| 35 | AB189125_3DI | I | 1998 | AB189125 | Indonesia: Sumatra |

|  | **Isolate Label** | **Genotype** | **Year of Collection** | **Gen Bank**  **accession number** | **Country** |
| --- | --- | --- | --- | --- | --- |
| 36 | AY858037_3DI | I | 2004 | AY858037 | Indonesia:  Jakarta |
| 37 | AY858043_3DI | I | 2004 | AY858043 | Indonesia:  Jakarta |
| 38 | AY858046_3DI | I | 2004 | AY858046 | Indonesia:  Jakarta |
| 39 | AY858038_3DI | I | 1988 | AY858038 | Indonesia: Jakarta |
| 40 | EU081223_3DI | I | 2005 | EU081223 | Singapore |
| 41 | DQ401690_3DI | I |  | DQ401690 | Indonesia |
| 42 | AY858041_3DI | I | 2004 | AY858041 | Indonesia: Jakarta |
| 43 | AB189128_3DI | I | 1998 | AB189128 | Indonesia: Sumatra |
| 44 | KX380839_3DI | I | 2012 | KX380839 | Singapore |
| 45 | AY858046_3DI | I | 2004 | AY858046 | Indonesia: Jakarta |
| 46 | AY676352_3DII | II |  | AY676352 | Thailand: Bangkok |
| 47 | DQ675522_3DII | II | 1998 | DQ675522 | Taiwan |
| 48 | DQ863638_3DII | II | 1973 | DQ863638 | Thailand: Bangkok |
| 49 | EU482453_3DII | II | 2006 | EU482453 | Viet Nam: south |
| 50 | AY876494_3DII | II | 1994 | AY876494 | Thailand |
| 51 | FJ744734_3DII | II | 2001 | FJ744734 | Thailand: Kamphaeng |
| 52 | EU482459_3DII | II | 2006 | EU482459 | Viet Nam: south |
| 53 | FJ744728_3DII | II | 2001 | FJ744728 | Thailand: Kamphaeng |
| 54 | EU482461_3DII | II | 2007 | EU482461 | Viet Nam: south |
| 55 | AY676350_3DII | II | 1993 | AY676350 | Thailand: Bangkok |
| 56 | FJ461337_3DII | II | 2008 | FJ461337 | Viet Nam: south |
| 57 | FJ687448_3DII | II | 2001 | FJ687448 | Thailand: Kamphaeng |
| 58 | FJ744726_3DII | II | 2001 | FJ744726 | Thailand: Kamphaeng |

|  | **Isolate Label** | **Genotype** | **Year of Collection** | **Gen Bank accession**  **number** | **Country** |
| --- | --- | --- | --- | --- | --- |
| 59 | KF955457_3DII | II | 2007 | KF955457 | Viet Nam |
| 60 | AY496873_3DII | II | 2002 | AY496873 | Bangladesh |
| 61 | KJ622197_3DII | II | 2013 | KJ622197 | China |
| 63 | KF824903_3DII | II | 2013 | KF824903 | China |
| 64 | KF824902_3DII . | II | 2013 | KF824902 | China |
| 65 | KJ737429_3DII | II | 1994 | KJ737429 | Thailand |
| 66 | KY849772_3DII | II | 2010 | KY849772 | Laos: Vientiane |
| 67 | KY849771_3DII | II | 2010 | KY849771 | Laos: Vientiane |
| 68 | KY849774_3DII | II | 2010 | KY849774 | Laos: Vientiane |
| 69 | KY849770_3DII | II | 2010 | KY849770 | Laos: Vientiane |
| 70 | KY849773_3DII | II | 2010 | KY849773 | Laos: Vientiane |
| 71 | KR296743_3DII | II | 2013 | KR296743 | China |
| 72 | KY849769_3DII | II | 2010 | KY849769 | Laos: Vientiane |
| 73 | KY849775_3DII | II | 2010 | KY849775 | Laos: Vientiane |
| 74 | KJ622198_3DII | II | 2013 | KJ622198 | China |
| 75 | GQ868571_3DIII | III | 2002 | GQ868571 | Colombia: Santander |
| 76 | EU529691_3DIII | III | 2001 | EU529691 | Venezuela: DF/Caracas |
| 77 | AY662691_3DIII | III |  | AY662691 | Singapore |
| 78 | AY770511_3DIII | III |  | AY770511 | India: Gwalior |
| 79 | GQ466079_3DIII | III | 2008 | GQ466079 | India: Delhi |
| 80 | AY099336_3DIII | III |  | AY099336 | Sri Lanka |
| 81 | GU131872_3DIII | III | 2007 | GU131872 | Brazil: Sao Paulo |
| 82 | EU081182_3DIII | III | 2005 | EU081182 | Singapore |
| 83 | HQ705618_3DIII | III | 2009 | HQ705618 | Nicaragua: Managua |
| 84 | JF504679_3DIII | III | 2009 | JF504679 | China: Zhejiang Yaw |
| 85 | FJ182013_3DIII | III | 1998 | FJ182013 | USA: Puerto Rico |
| 86 | FJ882573_3DIII | III | 1993 | FJ882573 | Sri Lanka |
| 87 | FJ898440_3DIII | III | 2003 | FJ898440 | Mexico: Morelos |
| 88 | JQ922557_3DIII | III | 2005 | JQ922557 | India: Kolkata, West Bengal |
| 89 | KF041254_3DIII | III | 2008 | KF041254 | Pakistan: Karachi |
| 90 | KF041255_3DIII . | III | 2007 | KF041255 | Pakistan: Hyderabad |

|  | **Isolate Label** | **Genotype** | **Year of Collection** | **Gen Bank**  **accession number** | **Country** |
| --- | --- | --- | --- | --- | --- |
| 91 | KF041257_3DIII | III | 2006 | KF041257 | Pakistan:  Karachi |
| 92 | KF041258_3DIII | III | 2009 | KF041258 | Pakistan:  Karachi |
| 93 | KF041259_3DIII | III | 2006 | KF041259 | Pakistan:  Karachi |
| 94 | KF954945_3DIII | III | 2013 | KF954945 | China:  Zhongshan |
| 95 | KF954947_3DIII | III | 2013 | KF954947 | China: Zhongshan |
| 96 | KJ643590_3DIII | III | 2007 | KJ643590 | Peru |
| 97 | JF808129_3DIII | III | 2003 | JF808129 | Paraguay: Asuncion (Central) |
| 98 | KF954946_3DIII | III | 2013 | KF954946 | China: Zhongshan |
| 99 | KT726350_3DIII | III | 2001 | KT726350 | Cuba |
| 100 | MF370226_3DIII | III | 2013 | MF370226 | China |
| 101 | KU509282_3DV | III | 2009 | KU509282 | Senegal |
| 102 | JN697379_3DV | III | 2006 | JN697379 | Brazil |
| 103 | KU050695_3DV | III | 1956 | KU050695 | Philippines |
