# Supplementary material for: Genetic Variation in the Domain II, 3′ Untranslated Region of Human and Mosquito Derived Dengue Virus Strains in Sri Lanka
Source: Viruses. 2021 Mar 5;13(3):421. doi: 10.3390/v13030421 (PMC8001906; doi:10.3390/v13030421)
Supplement: Supplementary file 1 [file viruses-13-00421-s001.zip › Supplimentry files/Supplimentry tables/Table S2.docx]

Table S2: Source of DENV1 virus isolates used for sequence analysis.

|  | **Isolate Label** | **Genotype** | **Year of Collection** | **Gen Bank accession number** | **Country** |
| --- | --- | --- | --- | --- | --- |
| 1 | EU848545_1DI_Ref |  |  | EU848545 |  |
| 2 | D1H_2019SL |  | 2019 |  | Sri Lanka |
| 3 | D1M1_2019SL |  | 2019 |  | Sri Lanka |
| 4 | D1M2_2019SL |  | 2019 |  | Sri Lanka |
| 5 | D1M3_2019SL |  | 2019 |  | Sri Lanka |
| 6 | D1M4_2019SL |  | 2019 |  | Sri Lanka |
| 7 | D1M5_2019SL |  | 2019 |  | Sri Lanka |
| 8 | D3H_2019SL |  | 2019 |  | Sri Lanka |
| 9 | D3M1_2019SL |  | 2019 |  | Sri Lanka |
| 10 | D3M2_2019SL |  | 2019 |  | Sri Lanka |
| 11 | D3M3_2019SL |  | 2019 |  | Sri Lanka |
| 12 | D3M4_2019SL |  | 2019 |  | Sri Lanka |
| 13 | D3M5_2019SL |  | 2019 |  | Sri Lanka |
| 14 | D3M6_2019SL |  | 2019 |  | Sri Lanka |
| 15 | KJ726665_1DSL |  | 2012 | KJ726665 | Sri Lanka |
| 16 | KJ726663_1DSL |  | 2012 | KJ726663 | Sri Lanka |
| 17 | HQ891315_1DSL |  | 2009 | HQ891315 | Sri Lanka |
| 18 | JN054256_1DSL |  | 2009 | JN054256 | Sri Lanka |
| 19 | KJ468234_1DSL |  | 2013 | KJ468234 | Germany |
| 20 | KP398852_1DSL | I | 2014 | KP398852 | Sri Lanka |
| 21 | KJ726664_1DSL | I | 2012 | KJ726664 | Sri Lanka |
| 22 | HQ891314_1DSL |  | 2009 | HQ891314 | Sri Lanka |
| 23 | KJ726662_1DSL |  | 2012 | KJ726662 | Sri Lanka |
| 24 | HQ891316_1DSL |  | 2009 | HQ891316 | Sri Lanka |
| 25 | JN054255_1DSL |  | 2010 | JN054255 | Sri Lanka |
| 26 | AF309641_1DI | I |  | AF309641 | Colombia |
| 27 | AB074760_1DI | I |  | AB074760 |  |
| 28 | AF350498_1DI | I |  | AF350498 |  |
| 29 | AF298807_1DI | I |  | AF298807 | Cote d'Ivoire:  Abidjan |
| 30 | AY726555_1DI | I | 1998 | AY726555 | Myanmar |
| 31 | AY732477_1DI | I | 1991 | AY732477 | Thailand, Bangkok |
| 32 | AY732479_1DI | I | 2001 | AY732479 | Thailand,  Bangkok |
| 33 | AY732480_1DI | I | 1994 | AY732480 | Thailand, Bangkok |
| 34 | AY732483_1DI | I | 1981 | AY732483 | Thailand, Bangkok |

|  | **Isolate Label** | **Genotype** | **Year of Collection** | **Gen Bank accession number** | **Country** |
| --- | --- | --- | --- | --- | --- |
| 35 | AY835999_1DI | I | 2004 | AY835999 | Chinese |
| 36 | DQ285561_1DI | I | 2004 | DQ285561 | Seychelles |
| 37 | EU081226_1DI | I | 2005 | EU081226 | Singapore |
| 38 | HG316481_1DI | I | 2010 | HG316481 | Singapore |
| 39 | HG316482_1DI | I | 2010 | HG316482 | Thailand |
| 40 | HQ891316_1DI | I | 2009 | HQ891316 | Sri Lanka |
| 41 | JN638340_1DI | I | 1997 | JN638340 | Thailand |
| 42 | JN638342_1DI | I | 1990 | JN638342 | Thailand |
| 43 | JN638344_1DI | I | 1995 | JN638344 | Thailand |
| 44 | U88537_1DI | I |  | U88537 |  |
| 45 | KJ726662_1DI | I | 2012 | KJ726662 | Sri Lanka |
| 46 | EF457905_1DIII | III | 1972 | EF457905 | Malaysia |
| 47 | DQ672564_1DIV | IV | 2001 | DQ672564 | Hawaii |
| 48 | EU863650_1DIV | IV | 2002 | EU863650 | Chile |
| 49 | FJ196842_1DIV | IV | 2003 | FJ196842 | China |
| 50 | FJ196845_1DIV | IV | 1991 | FJ196845 | China |
| 51 | U88535_1DIV | IV |  | U88535 |  |
| 52 | AF226687_1DV | V |  | AF226687 | FGA/89 |
| 53 | AF514889_1DV | V |  | AF514889 | 297arg00 |
| 54 | AY732474_1DV | V | 1980 | AY732474 | Thailand |
| 55 | AY732476_1DV | V | 1980 | AY732476 | Thailand |
| 56 | AF514883_1DV | V | 1980 | AF514883 | Thailand |
| 57 | AF298808_1DV | V | 1998 | AF298808 | Djibouti |
| 58 | AY762084_1DV | V | 1993 | AY762084 | Singapore |
| 59 | EU081258_1DV | V | 2005 | EU081258 | Singapore |
| 60 | GU131962_1DV | V | 2007 | GU131962 | Mexico |
| 61 | HQ332182_1DV | V | 2006 | HQ332182 | Venezuela |
| 62 | JN903579_1DV | V | 2008 | JN903579 | India |
| 63 | JN903581_1DV | V | 2009 | JN903581 | India |
| 64 | JQ915080_1DV | V | 2010 | JQ915080 | New Caledonia |
| 65 | JQ922544_1DV | V | 1963 | JQ922544 | India |
| 66 | JQ922546_1DV | V | 1971 | JQ922546 | India |
| 67 | JQ922548_1DV | V | 2005 | JQ922548 | India |
| 68 | EU596501_1DV | V | 2004 | EU596501 | Nicaragua |
| 69 | KC692512_1DV | V | 2010 | KC692512 | Argentina |
| 70 | KF289072_1DV | V | 2011 | KF289072 | India |
| 71 | M87512_1DV | V | 1990 | M87512 | Singapore |
