# Supplementary material for: Genetic Variation in the Domain II, 3′ Untranslated Region of Human and Mosquito Derived Dengue Virus Strains in Sri Lanka
Source: Viruses. 2021 Mar 5;13(3):421. doi: 10.3390/v13030421 (PMC8001906; doi:10.3390/v13030421)
Supplement: Supplementary file 1 [file viruses-13-00421-s001.zip › Supplimentry files/Supplimentry tables/Table S3.docx]

Table S3. Homologous GenBank DENV sequences respective to Domain II 3'UTR of DENV3 study identified sequences

|  | **GenBank Accession number** | **Year of collection** | **Country** | **Strain** | **Genotype** | **Host** |
| --- | --- | --- | --- | --- | --- | --- |
| 1 | DQ118905 | 2003 | Brazil | D3BR/RP1/03 |  | Human |
| 2 | EF643017 | 2003 | Brazil | D3BR/RP1/2003 | III | Human |
| 3 | FJ639784 | 2003 | Venezuela | DENV-3/VE/BID-V2217/2003 |  | Human |
| 4 | FJ639804 | 2005 | Venezuela | DENV-3/VE/BID-V2240/2005 |  | Human |
| 5 | FJ898474 | 2007 | Venezuela | DENV-3/VE/BID-V2971/2007 |  | Human |
| 6 | GU131877 | 2007 | Brazil | DENV-3/BR/BID-V3609/2007 |  | Human |
| 7 | HQ671176 | 2009 | Nicaragua | DENV-3/NI/BID-V4856/2009 |  | Human |
| 8 | HQ705610 | 2009 | Nicaragua | DENV-3/NI/BID-V4860/2009 |  | Human |
| 9 | JF808122 | 2003 | Paraguay | D3PY/SUS/2003 | III | Human |
| 10 | JF937639 | 2010 | Nicaragua | DENV-3/NI/BID-V5680/2010 |  | Human |
| 11 | JN380903 | 2006 | Brazil | 80996/2006/BR/RJ |  | Human |
| 12 | JN383344 | 2006 | Brazil | mosq/81200/BR/RJ |  | Mosquito *Aedes aegypti* |
| 13 | JX669489 | 2003 | Brazil | 101905/BR-PE/03 |  | Human |
| 14 | JX669491 | 2002 | Brazil | 81257/BR-PE/02 |  | Human |
| 15 | JX669494 | 2005 | Brazil | 277/BR-PE/05 |  | Human |
| 16 | JX669496 | 2006 | Brazil | 603/BR-PE/06 |  | Human |
| 17 | JX669501 | 2005 | Brazil | 263/BR-PE/05 |  | Human |
| 18 | JX669502 | 2005 | Brazil | 283/BR-PE/05 |  | Human |
| 19 | KC425218 | 2002 | Brazil | 95016/BR-PE/02_clone_p |  | Human |
| 20 | KF921928 | 2006 | Nicaragua | DENV-3/NI/BID-V5483/2010 |  | Human |
| 21 | KF955485 | 2008 | Venezuela | DENV-3/VE/BID-V2488/2008 |  | Human |
