# Supplementary material for: Genetic Variation in the Domain II, 3′ Untranslated Region of Human and Mosquito Derived Dengue Virus Strains in Sri Lanka
Source: Viruses. 2021 Mar 5;13(3):421. doi: 10.3390/v13030421 (PMC8001906; doi:10.3390/v13030421)
Supplement: Supplementary file 1 [file viruses-13-00421-s001.zip › Supplimentry files/Supplimentry tables/Table S4.docx]

Table S4. Homologous GenBank DENV sequences respective to Domain II 3'UTR of DENV1 study identified sequences

|  | **GenBank Accession number** | **Year of collection** | **Country** | **Strain** | **Genotype** | **Host** |
| --- | --- | --- | --- | --- | --- | --- |
| 1 | MH891771 | 2017 | India | MCVR1715AD/2017 |  | Human |
| 2 | MH891767 | 2017 | India | MCVR0543AC/2017 |  | Human |
| 3 | MG097875 | 2012 | Singapore | SG(EHI)D1/18640Y12 |  | Human |
| 4 | MH214106 | 2014 | China | DV1E/China/GDgz/14243/2014 |  | Human |
| 5 | MH271405 | 2014 | China | DV1D/China/GDfs/14264/2014 |  | Human |
| 6 | KY672944 | 2013 | China | DENV-1/China/YN/YNH22 |  | Human |
| 7 | MG560151 | 2014 | India | UoH_6538 |  | Human |
| 8 | MG560150 | 2014 | India | UoH_5684 |  | Human |
| 9 | MG560149 | 2014 | India | UoH_5840 |  | Human |
| 10 | MG560148 | 2014 | India | UoH_7111 |  | Human |
| 11 | MH822959 | 2012 | India | T28_S69 | V | Human |
| 12 | MF033255 | 2016 | Singapore | 31808 |  | Human |
| 13 | MF033253 | 2016 | Singapore | 19492 |  | Human |
| 14 | MF033252 | 2016 | Singapore | 15628 |  | Human |
| 15 | MF033251 | 2016 | Singapore | 13085 |  | Human |
| 16 | MF033243 | 2016 | Singapore | 3720 |  | Human |
| 17 | MF033242 | 2015 | Singapore | 30014 |  | Human |
| 18 | MF033241 | 2015 | Singapore | 11053 |  | Human |
| 19 | MF033238 | 2015 | Singapore | 29793 |  | Human |
| 20 | MF033237 | 2015 | Singapore | 22125 |  | Human |
|  | **GenBank Accession number** | **Year of collection** | **Country** | **Strain** | **Genotype** | **Host** |
| 21 | MF033229 | 2015 | Singapore | 6038 |  | Human |
| 22 | MF033227 | 2015 | Singapore | 3329 |  | Human |
| 23 | MF033224 | 2014 | Singapore | 31741 |  | Human |
| 24 | MF033213 | 2014 | Singapore | 5538 |  | Human |
| 25 | MF033212 | 2013 | Singapore | 50458 |  | Human |
| 26 | MF033211 | 2013 | Singapore | 42381 |  | Human |
| 27 | MF033205 | 2012 | Singapore | 49862 |  | Human |
| 28 | MF033204 | 2012 | Singapore | 44692 |  | Human |
| 29 | MF033200 | 2012 | Singapore | 29368 |  | Human |
| 30 | MF033249 | 2014 | China | 8826 |  | Human |
| 31 | MF033250 | 2014 | China | 9778 |  | Human |
| 32 | MF033251 | 2014 | China | 13085 |  | Human |
| 33 | KX618706 | 2014 | India | 3083 |  | Human |
| 34 | KX618705 | 2014 | India | UOH_21749 |  | Human |
| 35 | KT827378 | 2015 | China | GZ/13986/D1/2015 |  | Human |
| 36 | KT827377 | 2014 | China | GZ/33206/D1/2014 |  | Human |
| 37 | KT827375 | 2014 | China | GZ/31358/D1/2014 |  | Human |
| 38 | KT827374 | 2014 | China | GZ/26977/D1/2014 |  | Human |
| 39 | KY921903 | 2015 | Singapore | SG(EHI)DED65008 | III | Human |
| 40 | GQ357692 | 2008 | Singapore | SG(EHI)DED65008 |  | Human |
| 41 | JF960211 | 2000 | Singapore | SG(EHI)D1/0091Y09 |  | Human |
| 42 | JN544409 | 2011 | Singapore | SG(EHI)D1/09106Y11 | III | Human |
| 43 | JN544407 | 2011 | Singapore | SG(EHI)D1/15834Y11 | III | Human |
| 44 | KR779783 | 2011 | Singapore | SG(EHI)D1/44614Y11 |  | Human |
|  | **GenBank Accession number** | **Year of collection** | **Country** | **Strain** | **Genotype** | **Host** |
| 45 | KJ806950 | 2013 | Singapore | SG(EHI)D1/50903Y13 |  | Human |
| 46 | KX459392 | 2014 | China | GZ8_81/S/Panyu/2014/DEV |  | Human |
| 47 | KX459391 | 2014 | China | GZ8_50/S/Zengcheng/2014/DEV1 |  | Human |
| 48 | KX459390 | 2014 | China | GZ8_48/S/Yuexiu/2014/DEV1 |  | Human |
| 49 | KX459389 | 2014 | China | GZ8_44/S/Tianhe/2014/DEV1 |  | Human |
| 51 | KX459387 | 2014 | China | Gz-2/M/GZ/2014/DEV1 |  | Human |
| 52 | KX459386 | 2014 | China | GZ8_9/S/Yuexiu/2014/DEV1 |  | Human |
| 53 | KX459385 | 2014 | China | GZ8_2/S/Yuexiu/2014/DEV1 |  | Human |
| 54 | KP974821 | 2014 | India | UOH 3041 |  | Human |
| 55 | KP974821 | 2014 | India | UOH_2999 |  | Human |
| 56 | KX380806 | 2013 | Singapore | D1/SG/CT49/2013 |  | Human |
| 57 | KX380805 | 2013 | Singapore | D1/SG/CT47/2013 |  | Human |
| 58 | KX380804 | 2013 | Singapore | D1/SG/CT48/2013 |  | Human |
| 59 | KX380803 | 2013 | Singapore | D1/SG/CT45/2013 |  | Human |
| 60 | KX380802 | 2012 | Singapore | D1/SG/CT25/2012 |  | Human |
| 61 | KX380801 | 2012 | Singapore | D1/SG/CT17/2012 |  | Human |
| 62 | KX380800 | 2012 | Singapore | D1/SG/CT16/2012 |  | Human |
| 63 | KX380799 | 2012 | Singapore | D1/SG/CT11/2012 |  | Human |
| 64 | KX380796 | 2012 | Singapore | D1/SG/CT1/2012 |  | Human |
| 65 | KM403636 | 2013 | Singapore | SGEHI(D1)03120Y13 | III | Human |
| 66 | KM403635 | 2013 | Singapore | SGEHI(D1)22176Y13 | III | Human |
| 67 | KM403634 | 2013 | Singapore | SGEHI(D1)40115Y13 | III | Human |
| 68 | KM403633 | 2013 | Singapore | SGEHI(D1)09125Y13 | III | Human |
| 69 | KM403632 | 2013 | Singapore | SGEHI(D1)51687Y13 | III | Human |
|  | **GenBank Accession number** | **Year of collection** | **Country** | **Strain** | **Genotype** | **Host** |
| 70 | KM403631 | 2013 | Singapore | SGEHI(D1)50722Y1 | III | Human |
| 71 | KM403630 | 2013 | Singapore | SGEHI(D1)22272Y13 | III | Human |
| 72 | KM403628 | 2013 | Singapore | SGEHI(D1)10498Y13 | III | Human |
| 73 | KM403627 | 2013 | Singapore | SGEHI(D1)03513Y13 | III | Human |
| 74 | KM403626 | 2013 | Singapore | SGEHI(D1)50837Y13 | III | Human |
| 75 | KM403625 | 2013 | Singapore | SGEHI(D1)50688Y13 | III | Human |
