# Supplementary material for: Genetic Variation in the Domain II, 3′ Untranslated Region of Human and Mosquito Derived Dengue Virus Strains in Sri Lanka
Source: Viruses. 2021 Mar 5;13(3):421. doi: 10.3390/v13030421 (PMC8001906; doi:10.3390/v13030421)
Supplement: Supplementary file 1 [file viruses-13-00421-s001.zip › Supplimentry files/Supplimentry tables/Table S5.docx]

Table S5: GenBank accession numbers obtained for the study sequences.

| **Study isolates** | **GenBank Accession numbers** |
| --- | --- |
| D4H_2019SL | MT355923 |
| D4M1_2019SL | MT355924 |
| D1H_2019SL | MT355925 |
| D1M1_2019SL | MT355926 |
