# Supplementary material for: Genetic Variation in the Domain II, 3′ Untranslated Region of Human and Mosquito Derived Dengue Virus Strains in Sri Lanka
Source: Viruses. 2021 Mar 5;13(3):421. doi: 10.3390/v13030421 (PMC8001906; doi:10.3390/v13030421)
Supplement: Supplementary file 1 [file viruses-13-00421-s001.zip › Supplimentry files/Supplimentry tables/Table S6.docx]

**Table S6. Mfold and RNAfold predicted secondary structures for RNA alignments of DENV3, Domain II region of 3’UTR sequences identified in the study, Sri Lankan isolates and DENV1 reference genotypes.**

| **DENV3** | | **Mfold predicted secondary structures** | **RNAfold predicted secondary structures** | | | | |
| --- | --- | --- | --- | --- | --- | --- | --- |
|  |  | **MFE structure** | **MFE structure** | | | **Centroid structure** | |
| **DENV3 reference** | M93130 | 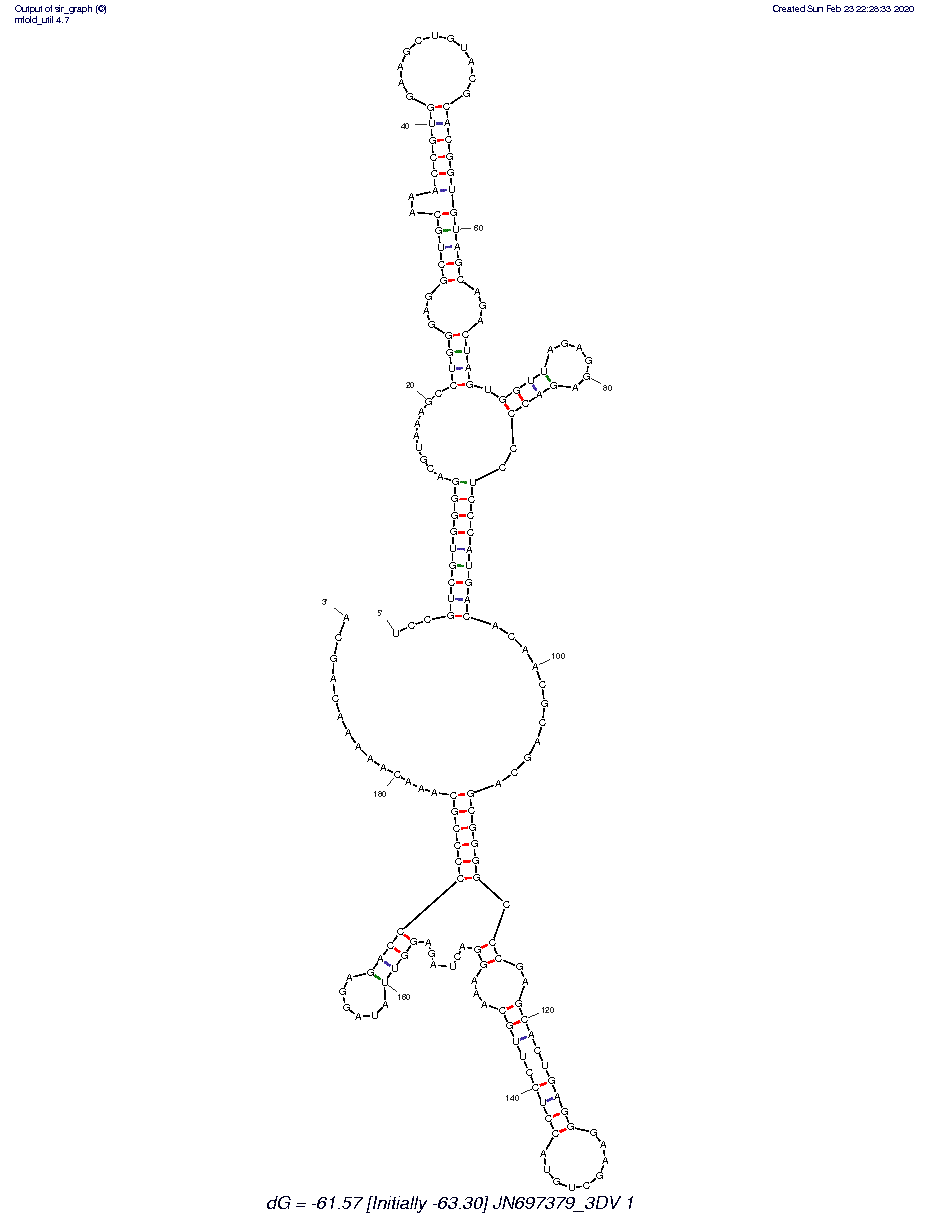  DB1    DB2 | -63.30 kcal/mol | 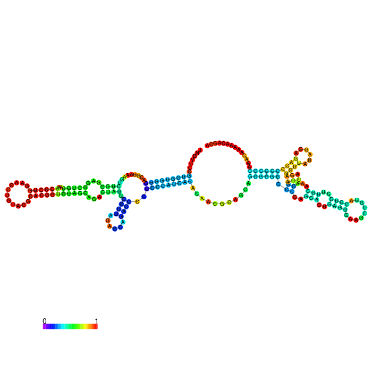  DB2  DB1 | | -62.711 kcal/mol | 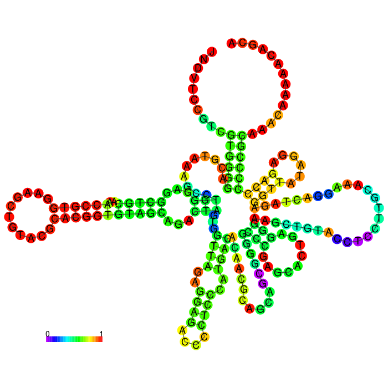 |
| **Study** | D1H_2019SL | 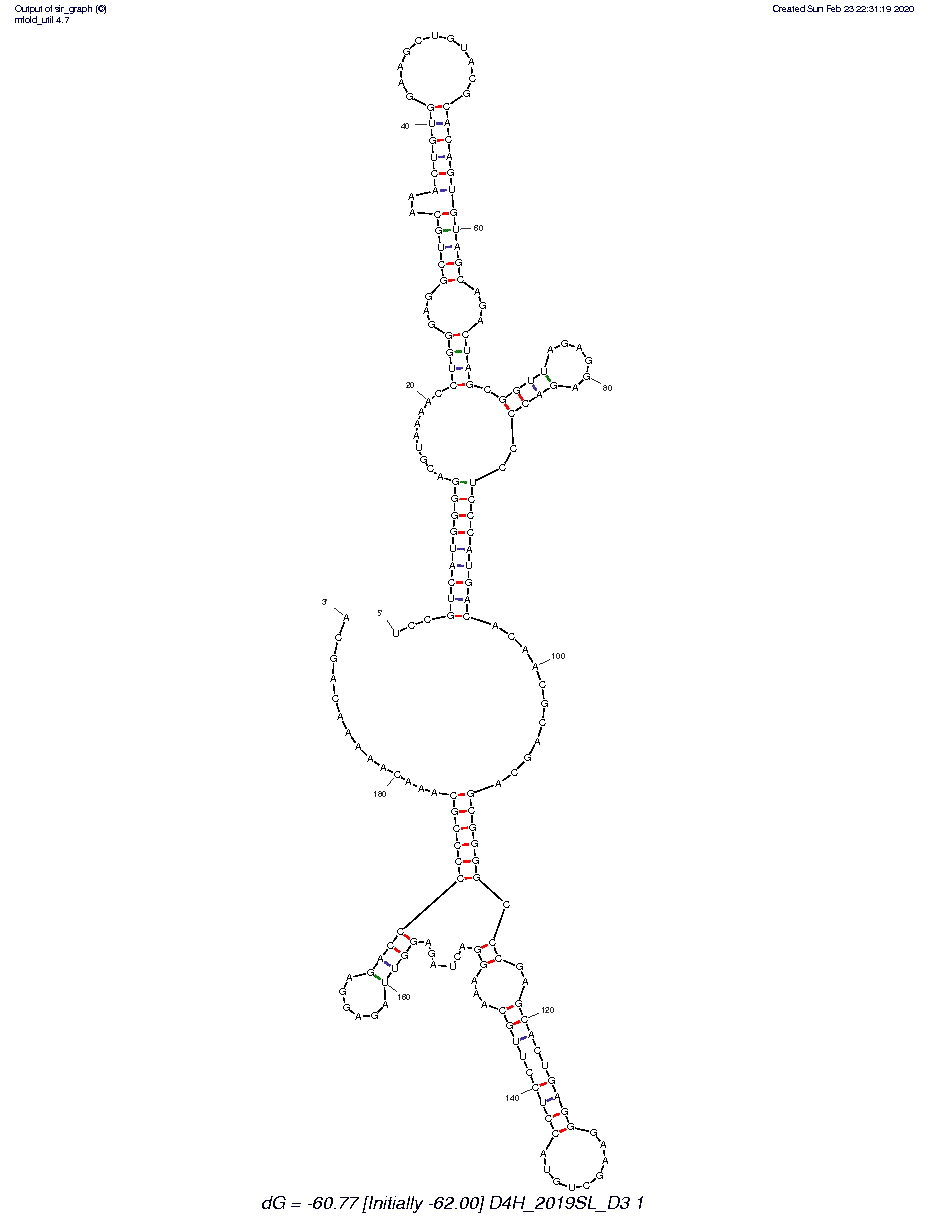   DB2  DB1 | -60.77 kcal/mol | 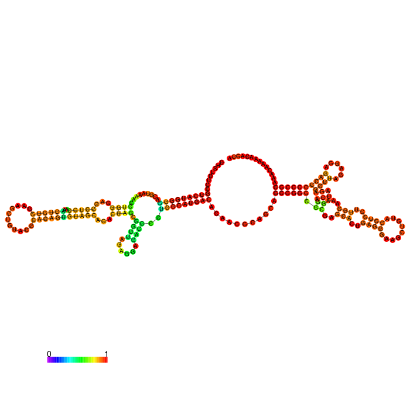  DB1  DB2 | | -60.78 kcal/mol | 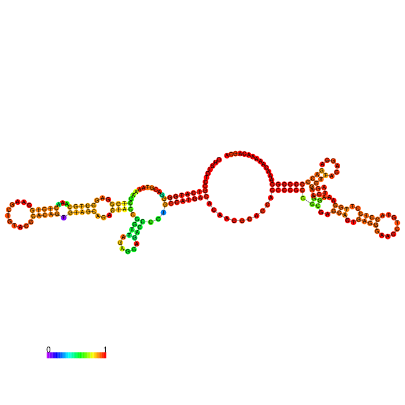 |
| **DENV3** | | **Mfold predicted secondary structures** | **RNAfold predicted secondary structures** | | | | |
|  |  | **MFE structure** | **MFE structure** | | | **Centroid structure** | |
| **Sri Lankan isolates of DENV3** | AY585848  FJ882573 | 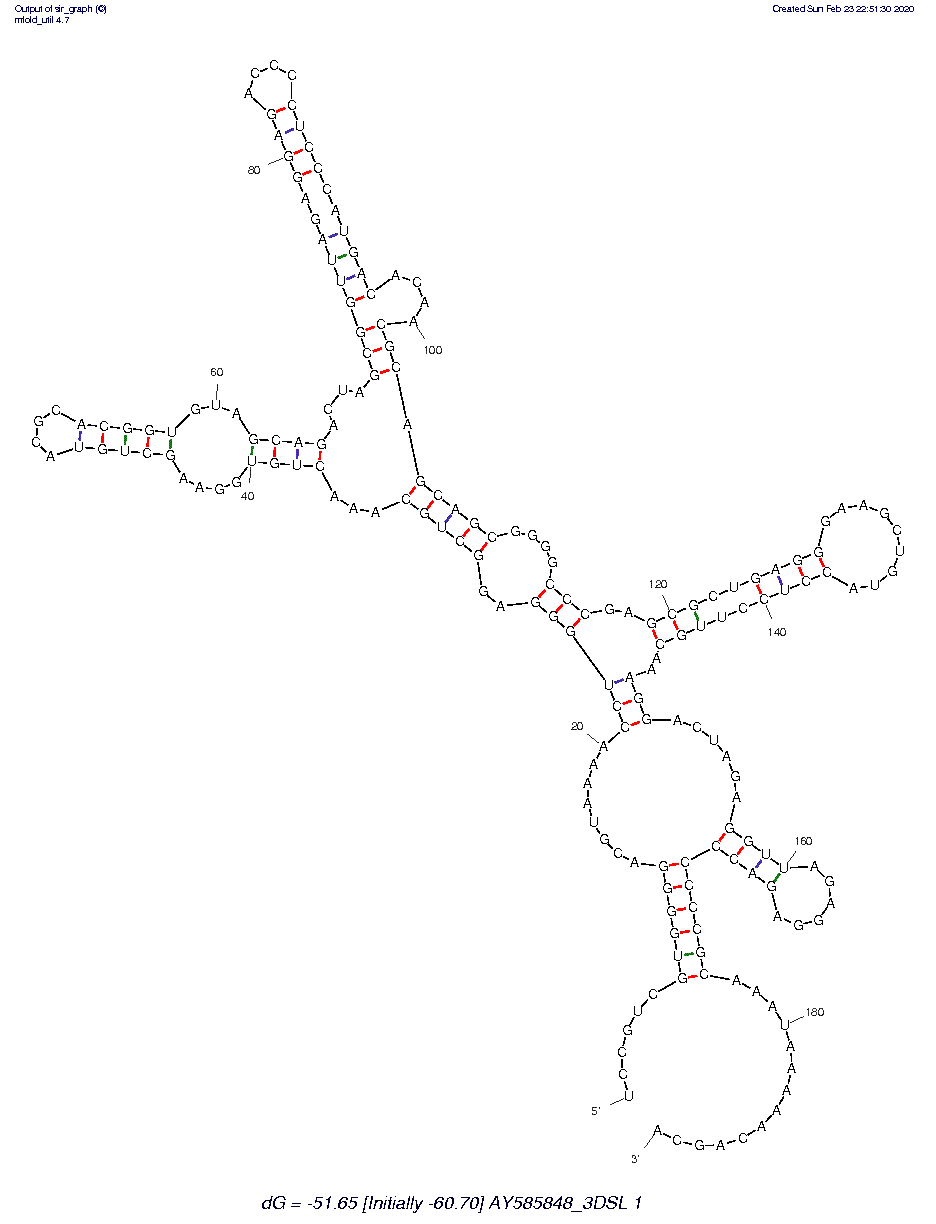 DB1  DB2 | -60.77 kcal/mol | 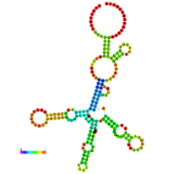  DB2  DB1 | | -59.71 kcal/mol | 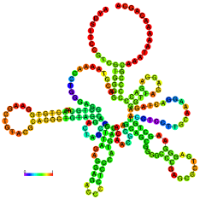 |
|  | AY85845  AY585846  GQ199886  AY585847  GQ199888  GQ199887  FJ582574  FJ882574 | 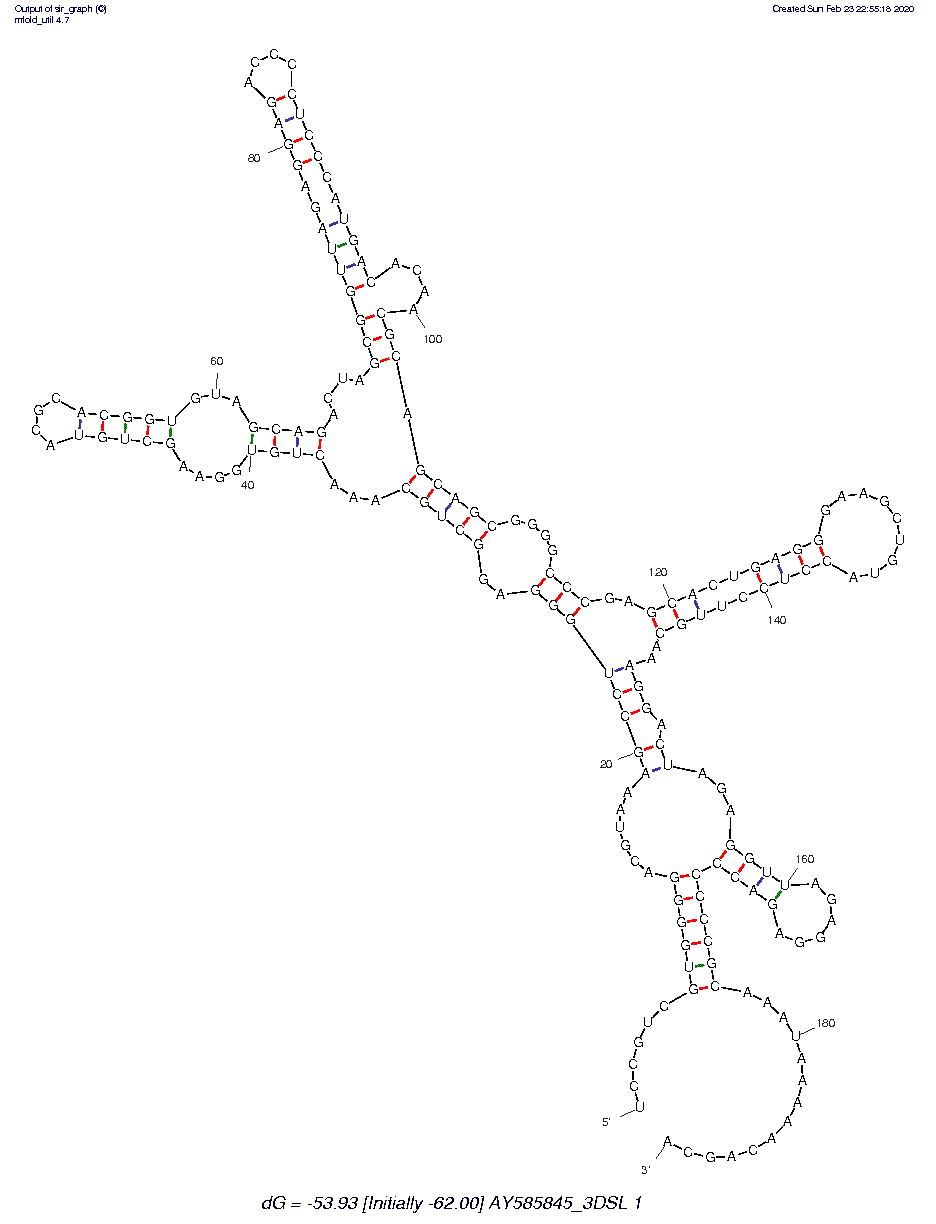 DB1  DB2 | -62.00  kcal/mol | 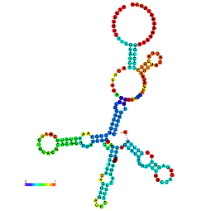  DB2  DB1 | | -61.23  kcal/mol | 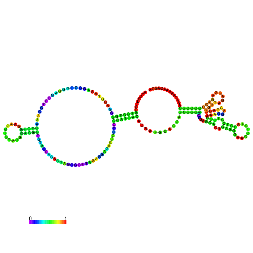 |
|  | GQ252674 | 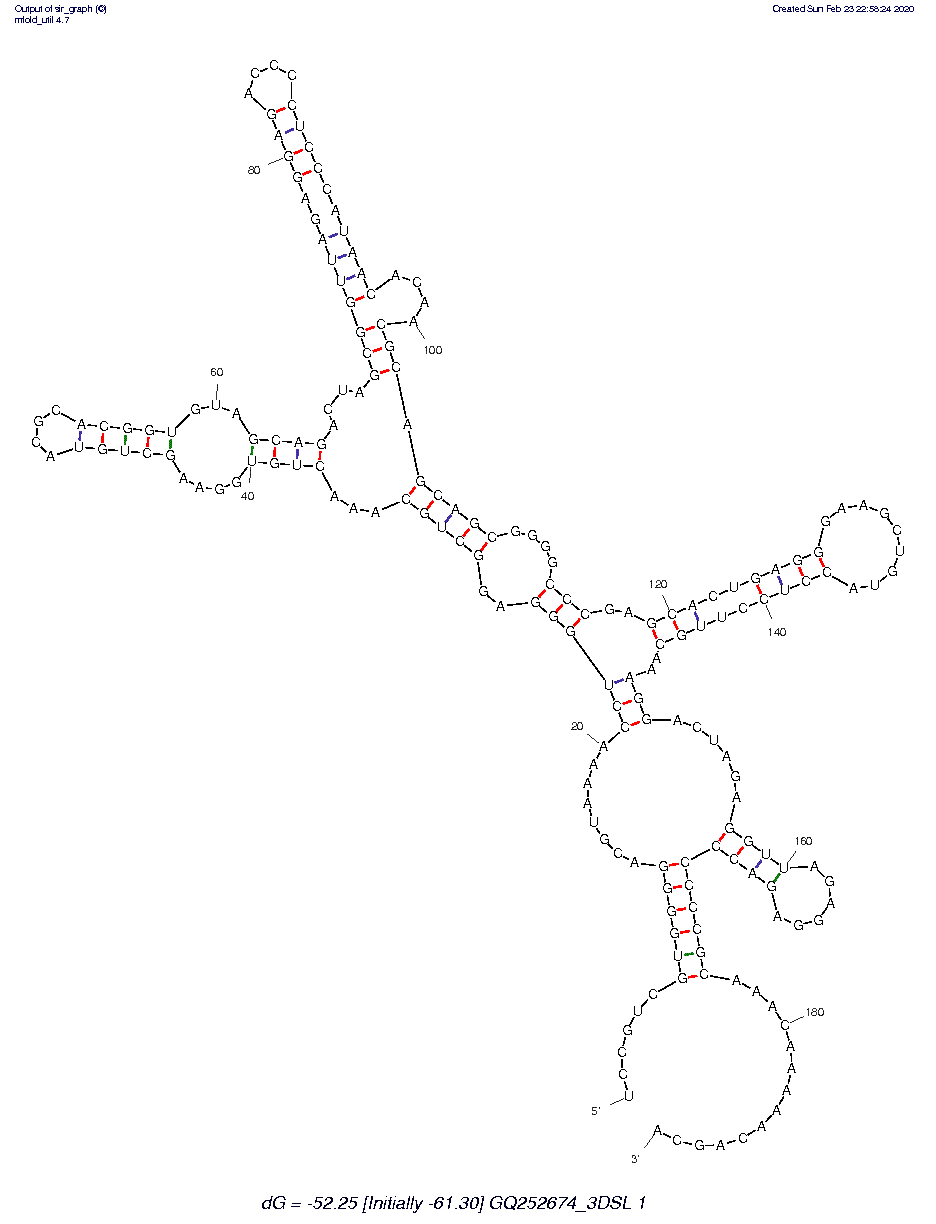 DB1  DB2 | -61.30 kcal/mol | 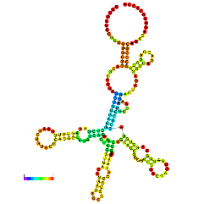  DB2  DB1 | | -59.61  kcal/mol | 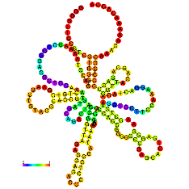 |
|  | AY099336 GQ199889  AY585851  AY585852 | 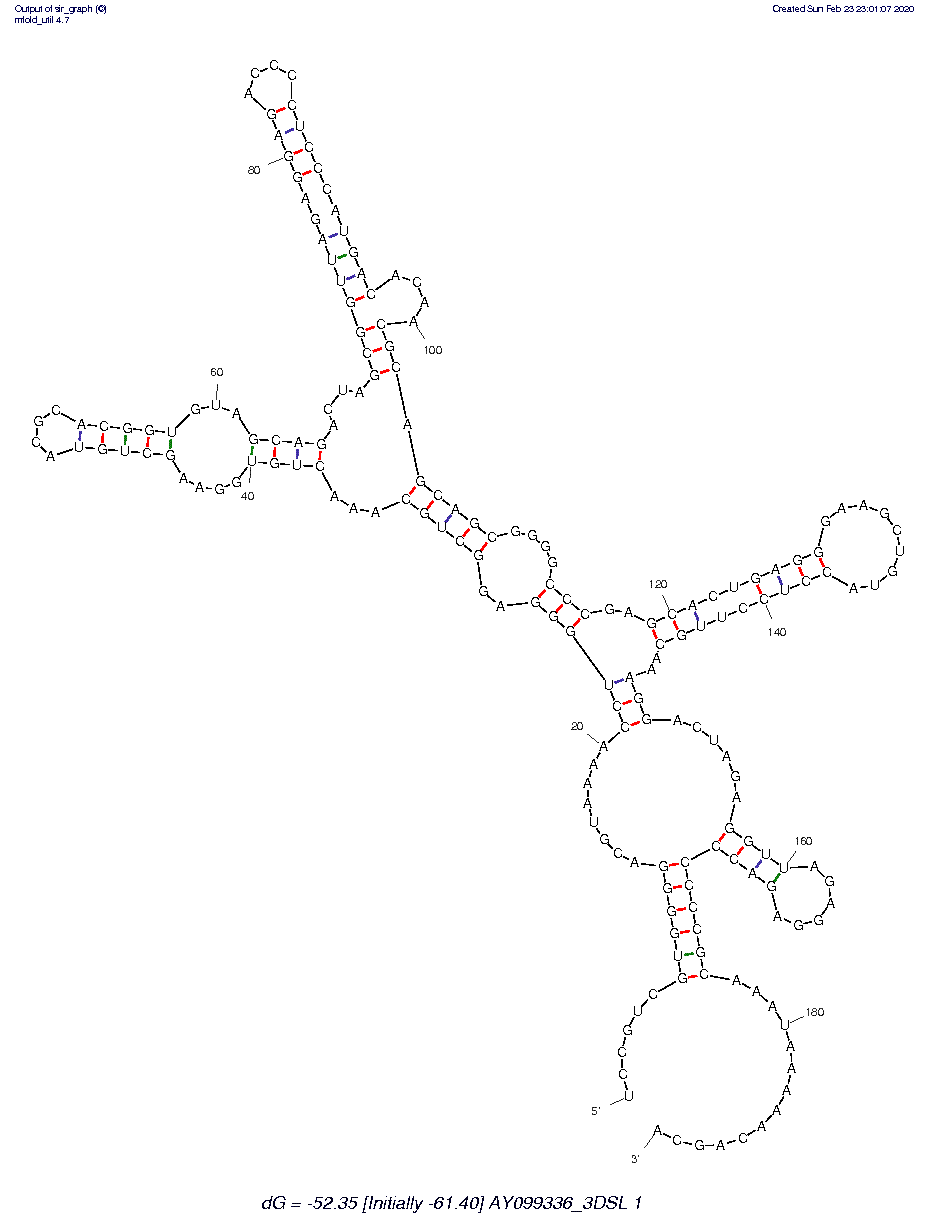 DB1    DB2 | -61.40  kcal/mol | 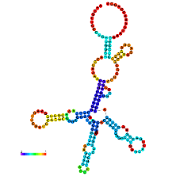  DB2  DB1 | | -60.09  kcal/mol | 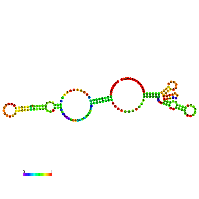 |
|  | JQ411814  FJ882571  KF955474 | 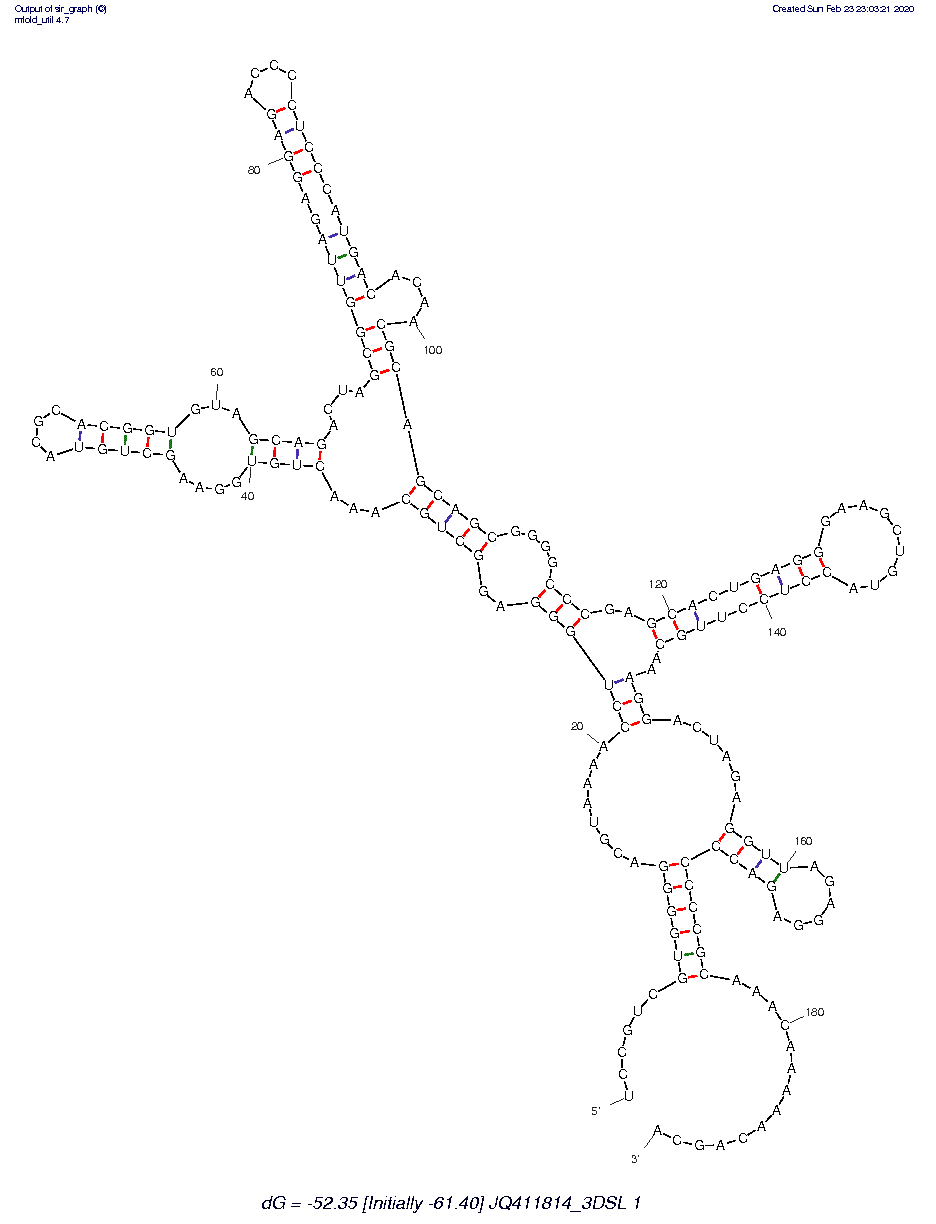 DB2  DB1 | -61.40 kcal/mol | 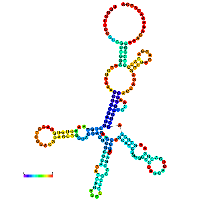  DB2  DB1 | | -62.71 kcal/mol | 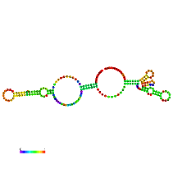 |
| **DENV3** | | **Mfold predicted secondary structures** | **RNAfold predicted secondary structures** | | | | |
|  |  | **MFE structure** | **MFE structure** | | | **Centroid structure** | |
| **DENV3 Genotype I** | AB189125 | 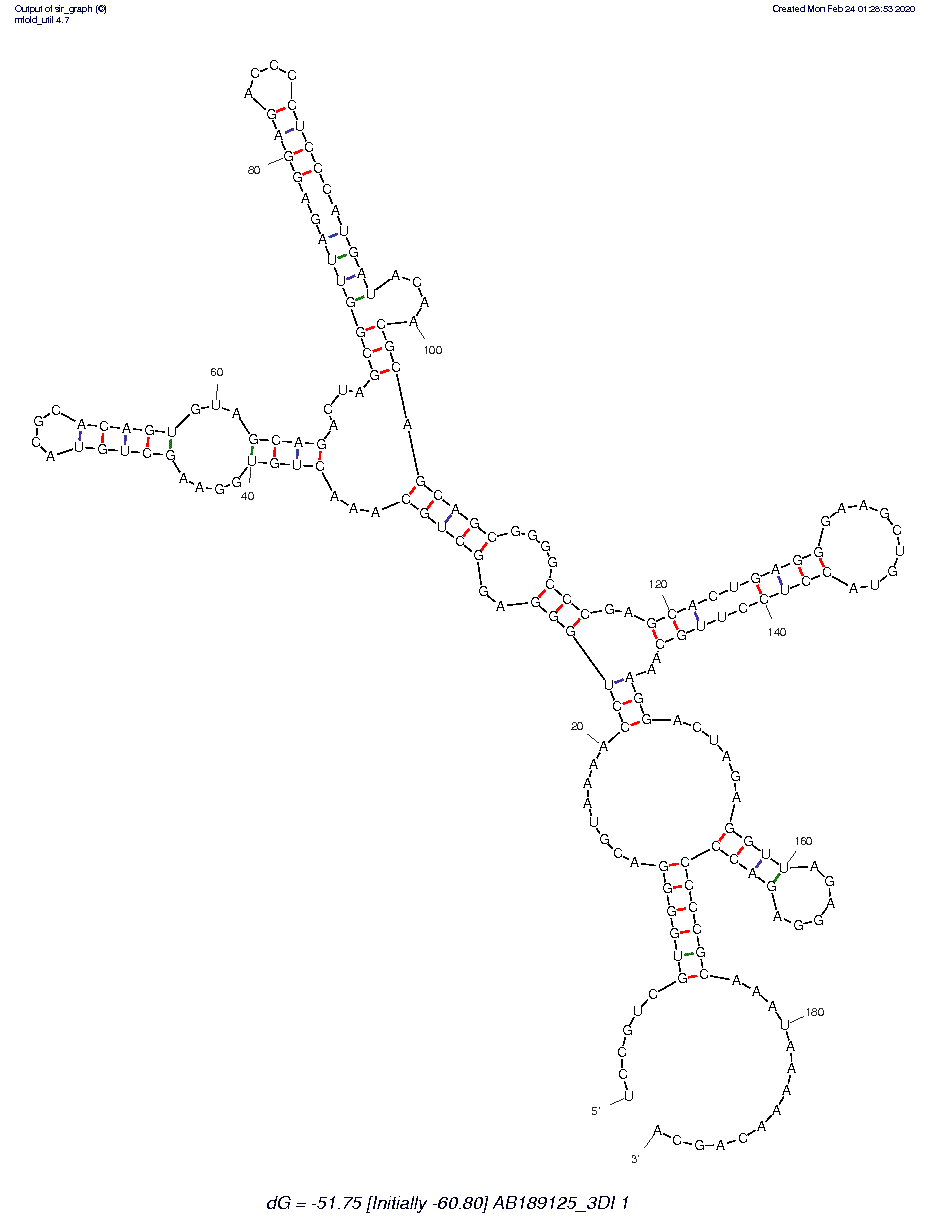 DB1  DB2 | 60.88 kcal/mol | 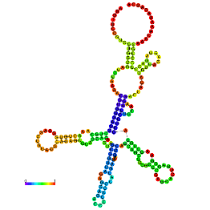  DB2  DB1 | | -59.52 kcal/mol | 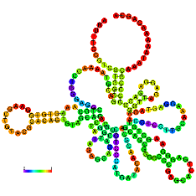 |
|  | AY648961 | 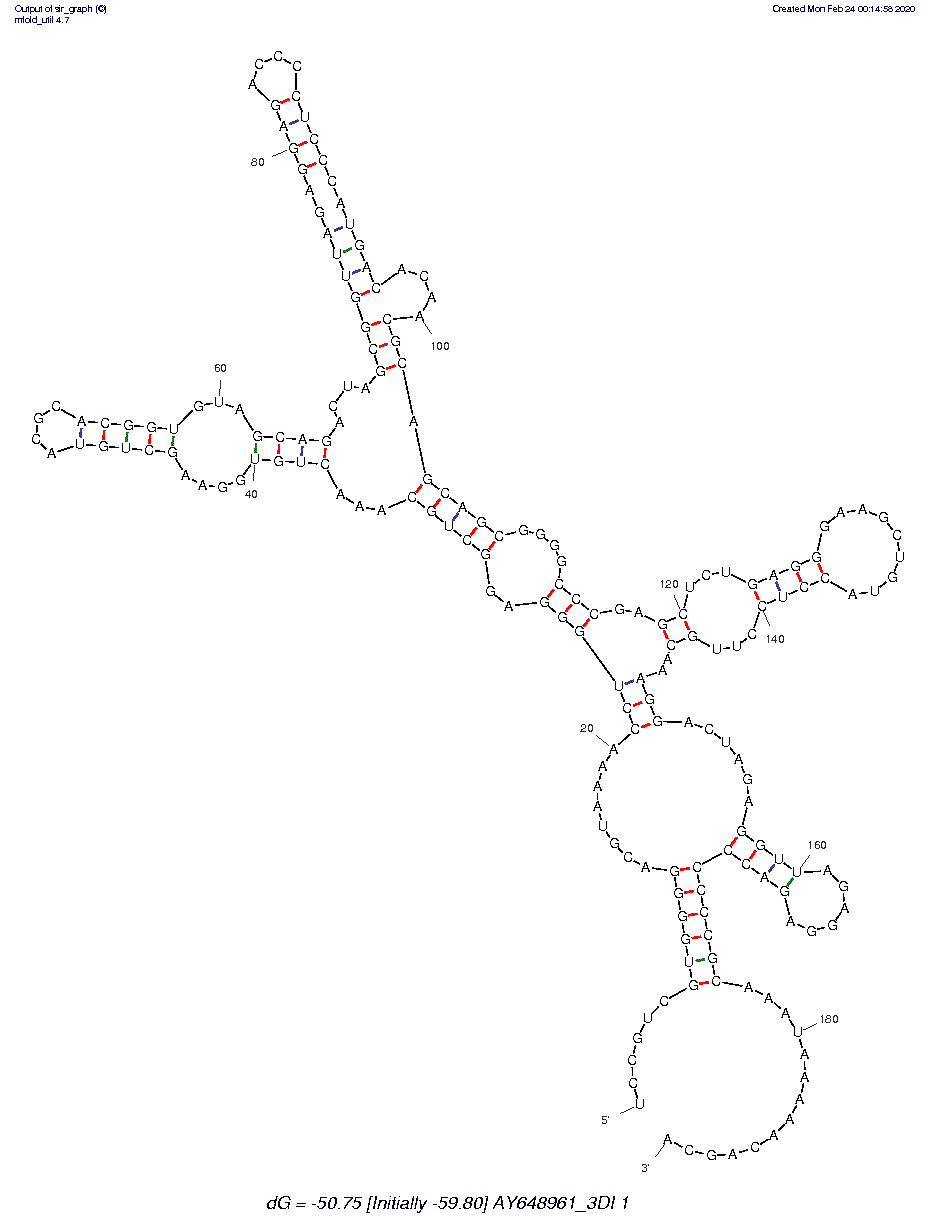 DB1  DB2 | -59.80 kcal/mol | 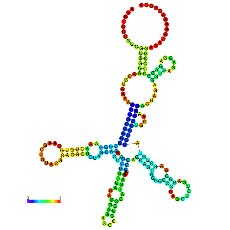  DB2  DB1 | | -59.99 kcal/mol | 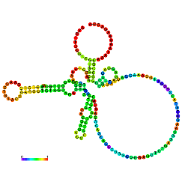 |
|  | AY858037 | 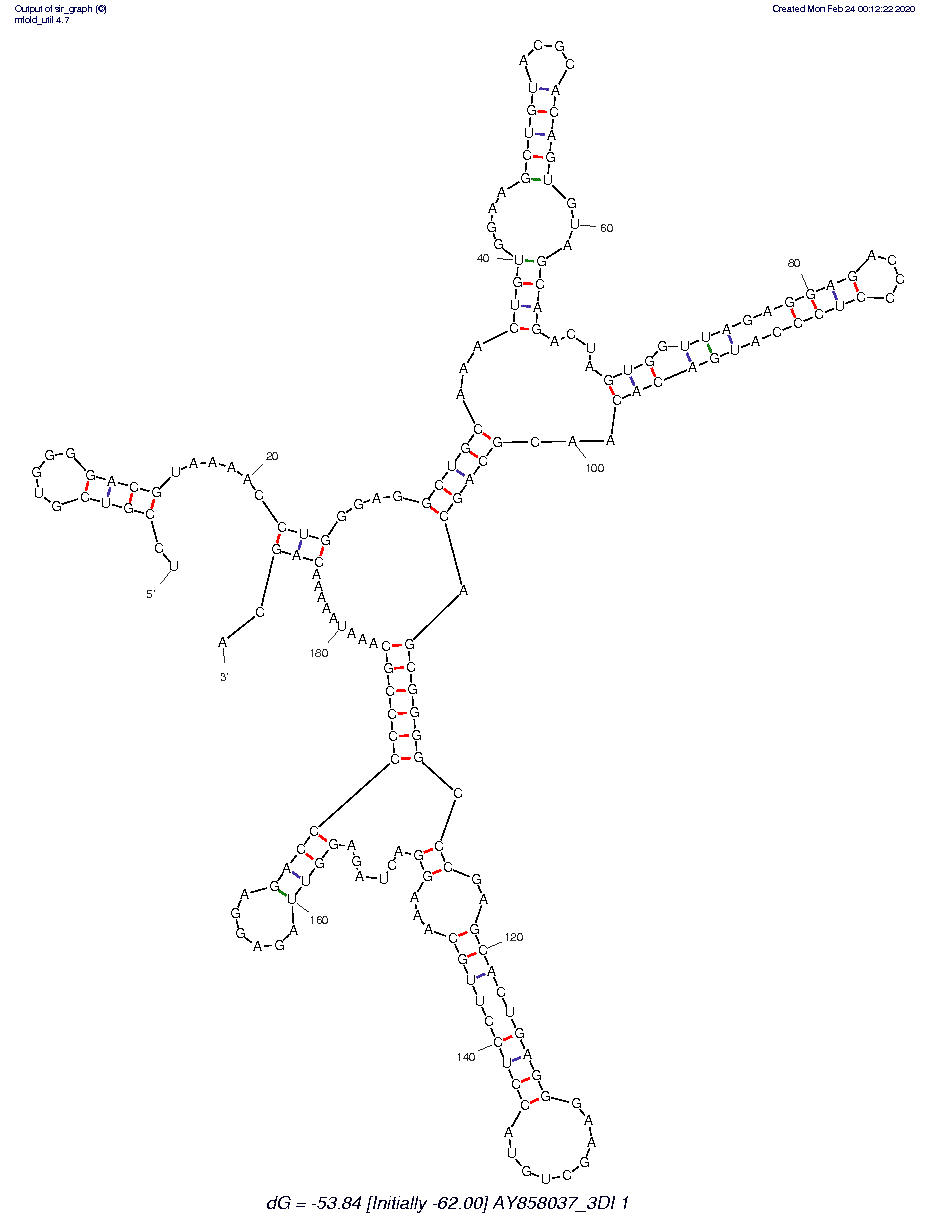 DB1  DB2 | -62.00  kcal/mol | 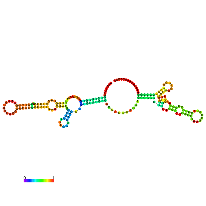  DB2  DB1 | | -60.84  kcal/mol | 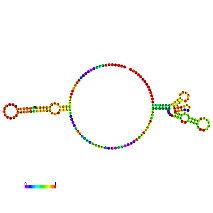 |
|  | AB189128  AY858046 | 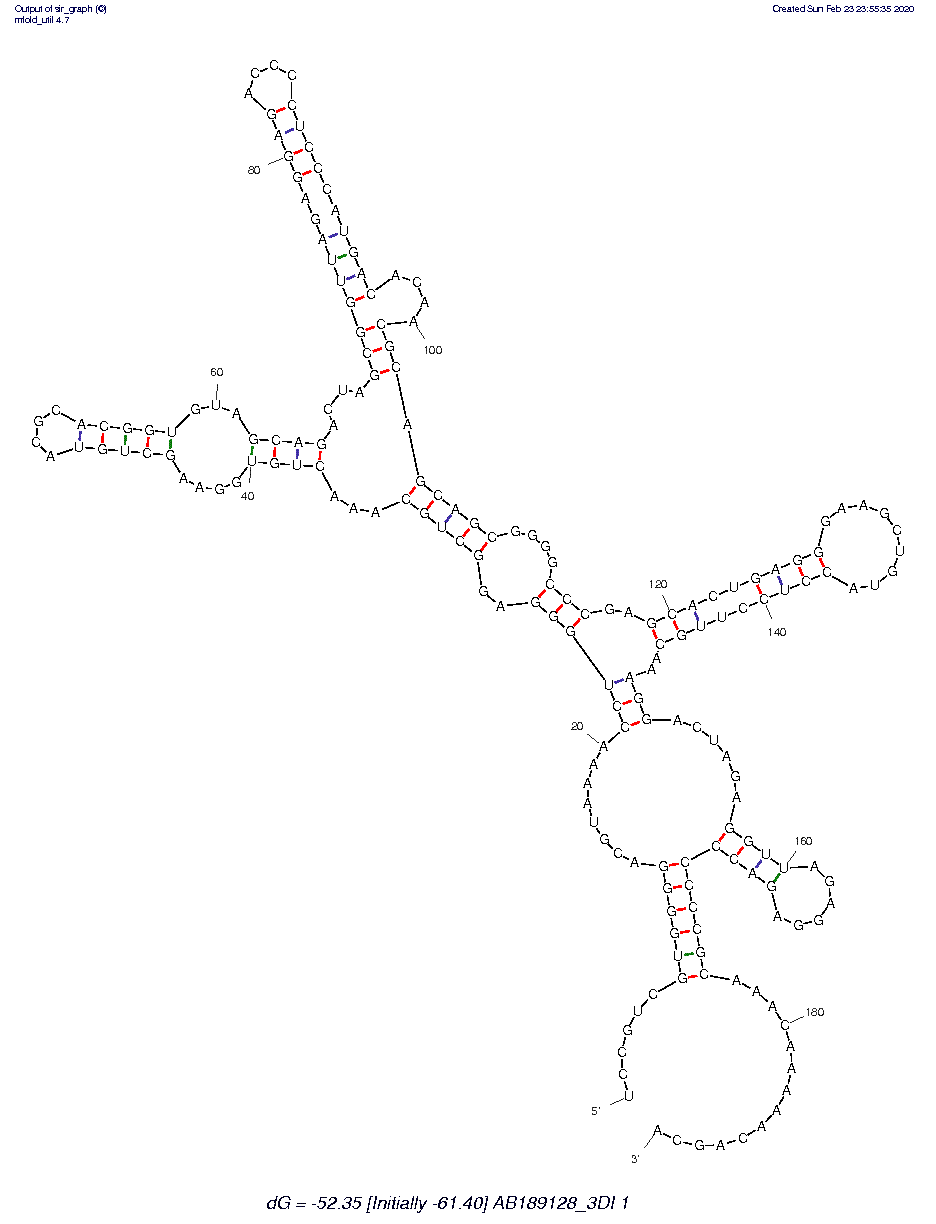 DB1  DB2 | -61.40  kcal/mol | 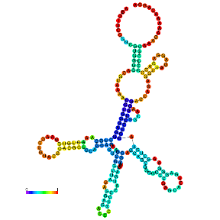  DB2  DB1 | | -60.15  kcal/mol | 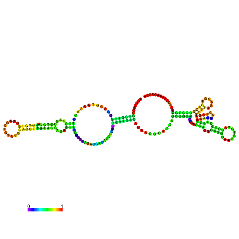 |
|  | DQ401690 | 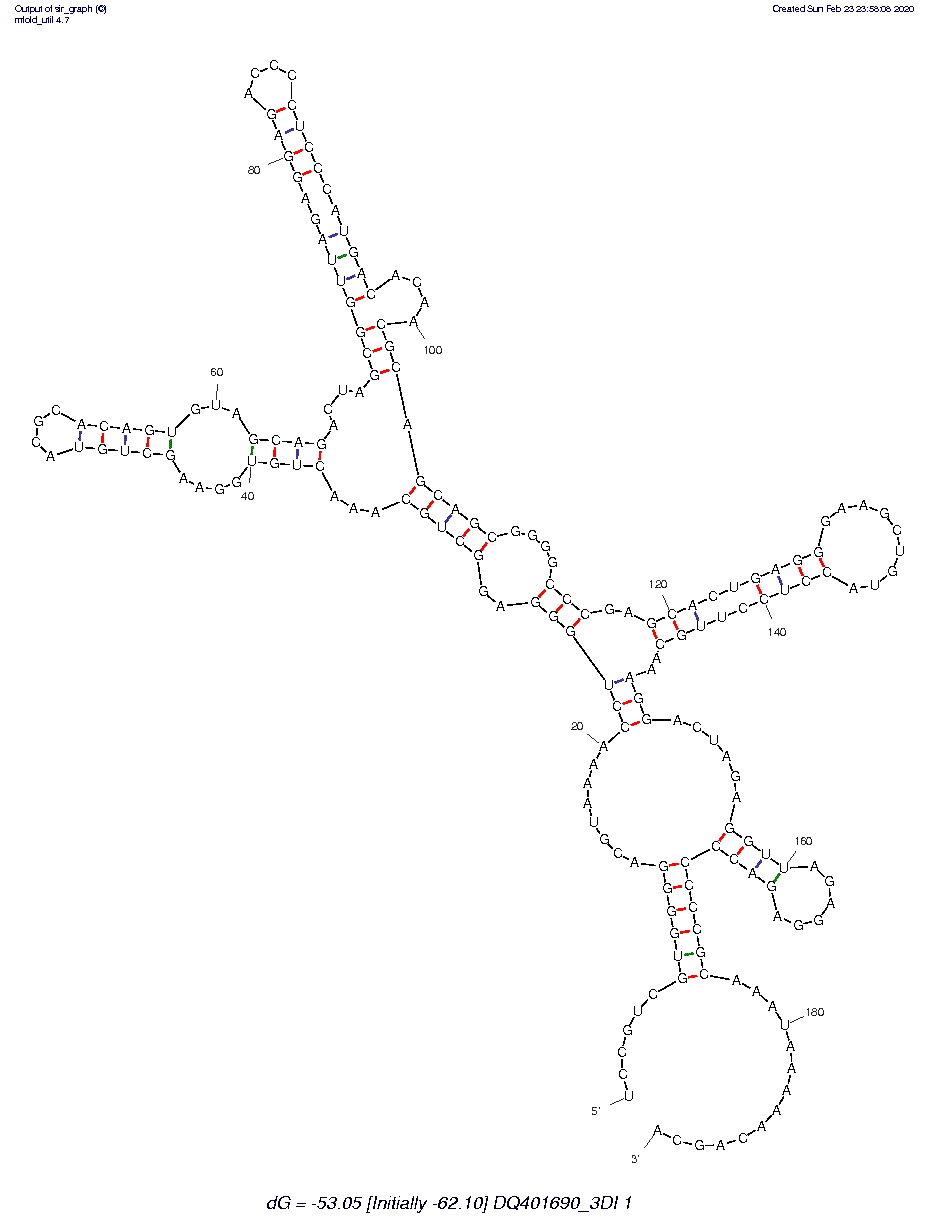 DB1  DB2 | -62.10  kcal/mol | 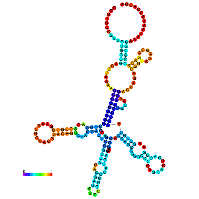  DB1  DB2 | | -60.72  kcal/mol | 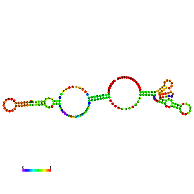 |
| **DENV3** | | **Mfold predicted secondary structures** | **RNAfold predicted secondary structures** | | | | |
|  |  | **MFE structure** | **MFE structure** | | | **Centroid structure** | |
| **DENV3 Genotype I** | AY858043  AY858038  EU081223  AY858041 | 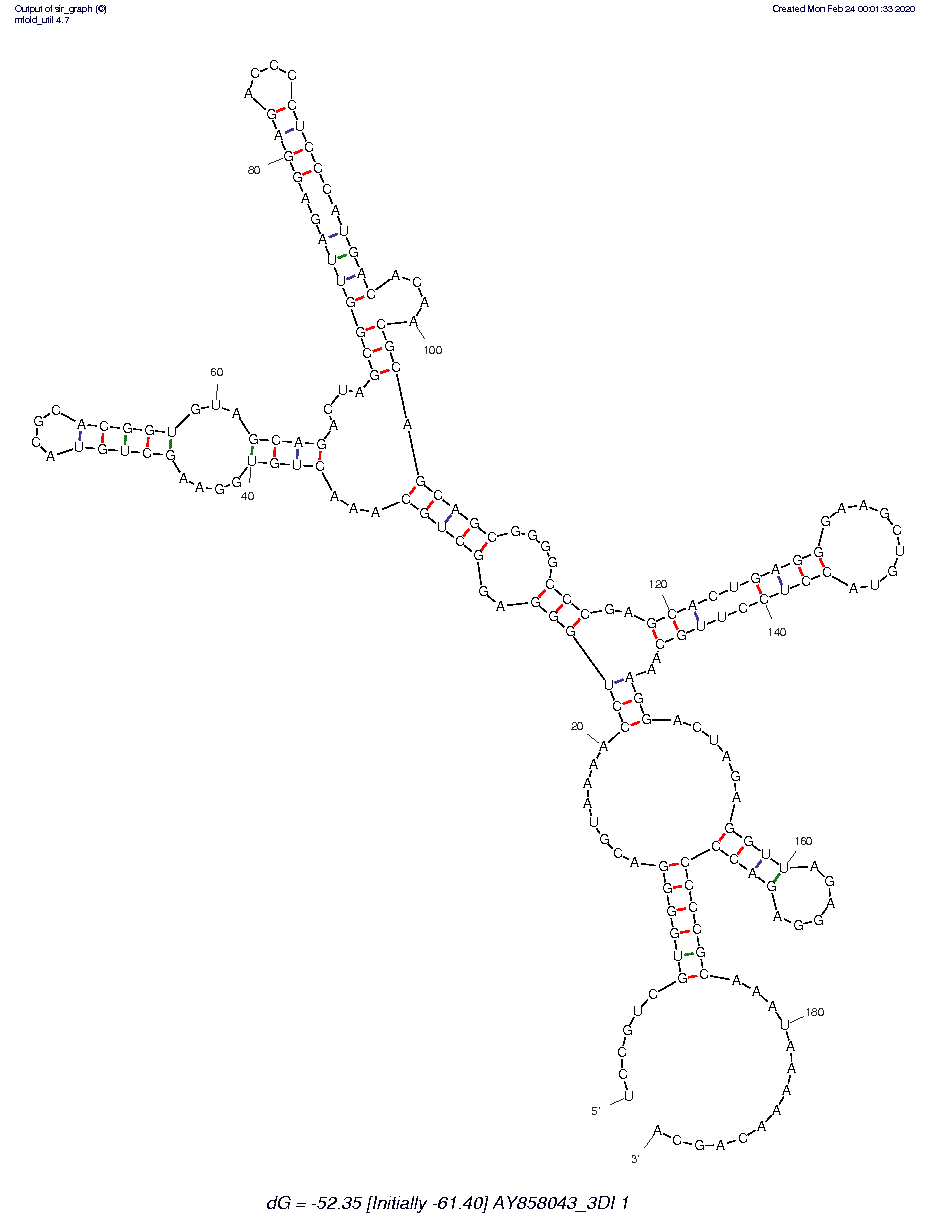 DB1  DB2 | -61.40  kcal/mol | 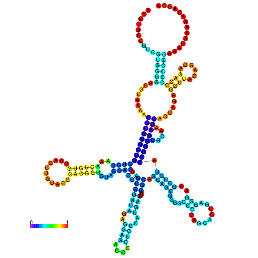  DB2  DB1 | | -60.09 kcal/mol | 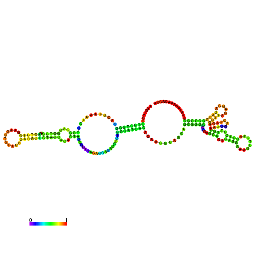 |
|  | KX380839 | 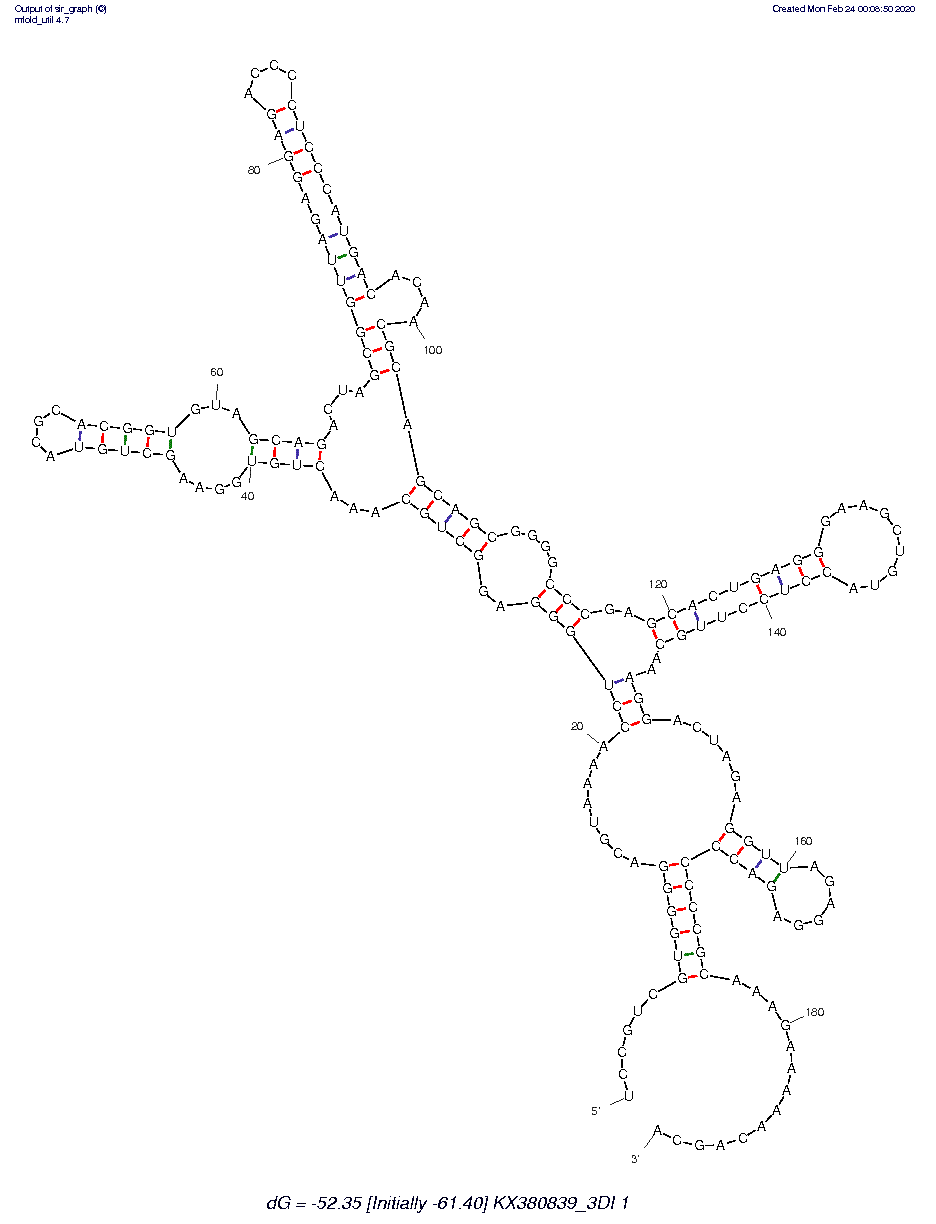 DB1  DB2 | -61.4  kcal/mol | 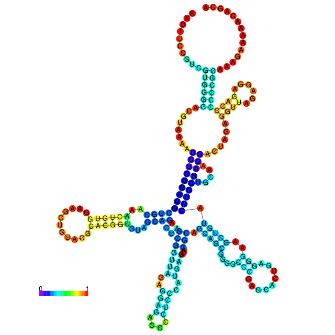  DB1  DB2 | | -60.13  kcal/mol | 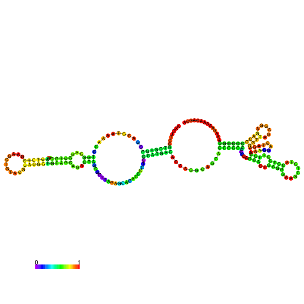 |
|  | AY744681 | 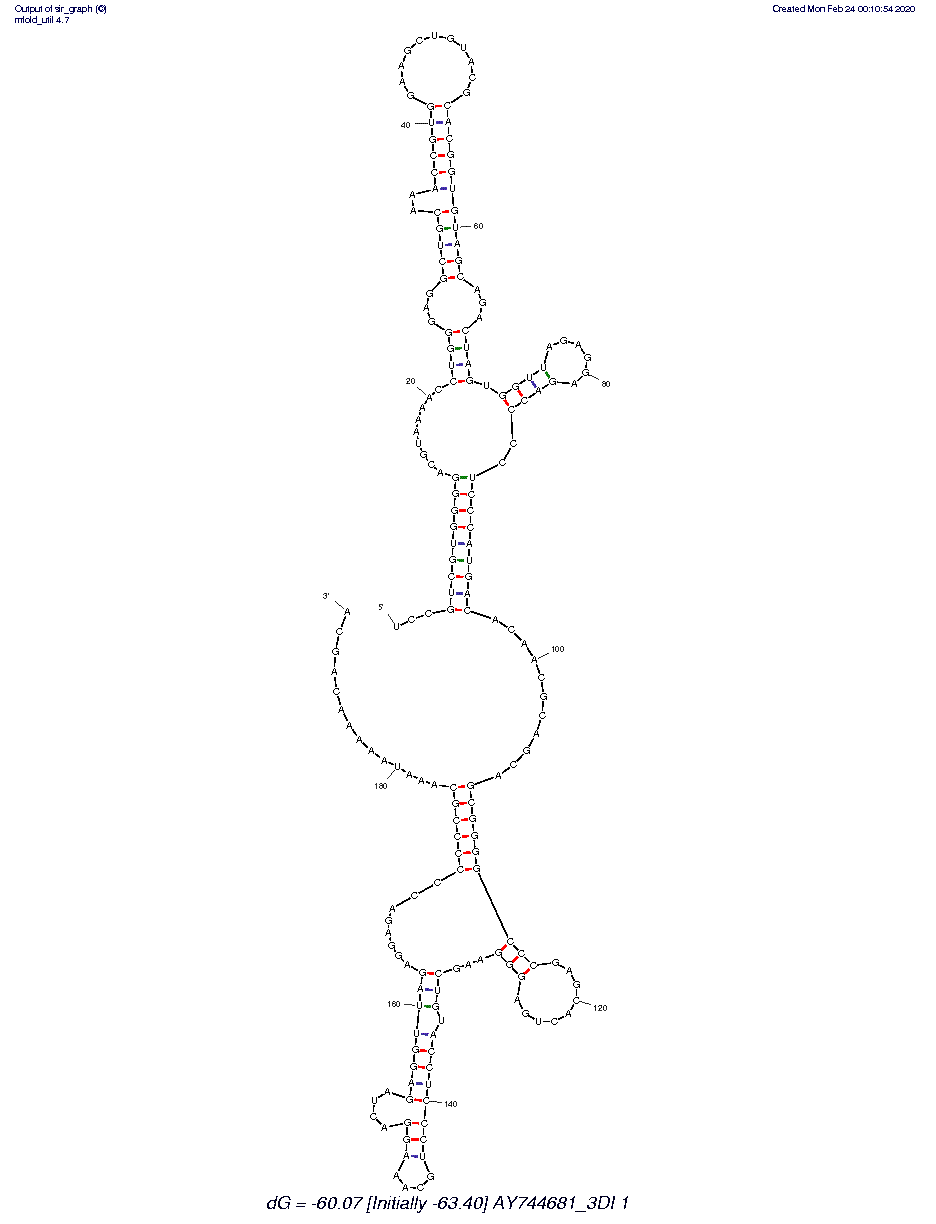 DB1  DB2 | -63.4  kcal/mol | 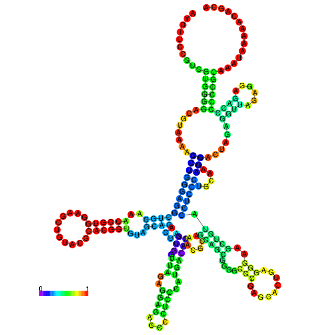  DB2  DB1 | | -63.03  kcal/mol | 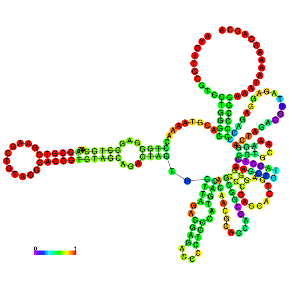 |
| **DENV3** | | **Mfold predicted secondary structures** | **RNAfold predicted secondary structures** | | | | |
|  |  | **MFE structure** | **MFE structure** | | | **Centroid structure** | |
| **DENV3 Genotype II** | KC261634 | 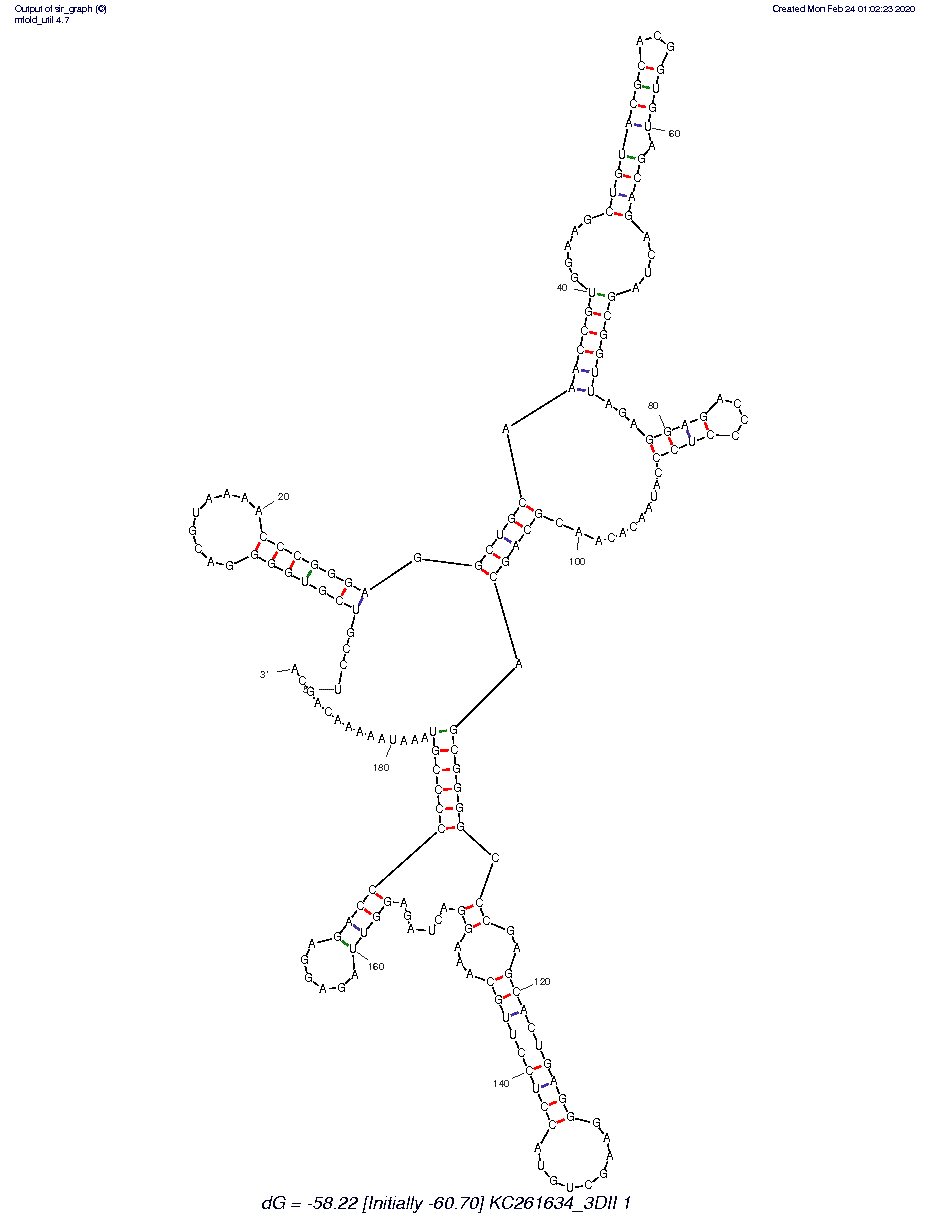 DB1  DB2 | -60.70 kcal/mol | | 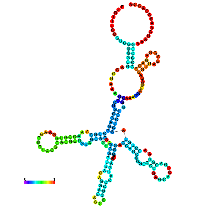  DB2  DB1 | -63.34  kcal/mol | 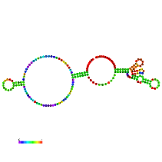 |
|  | FJ461337  AY876494  FJ744734  FJ687448  FJ744728  AY676350  AY496873 | 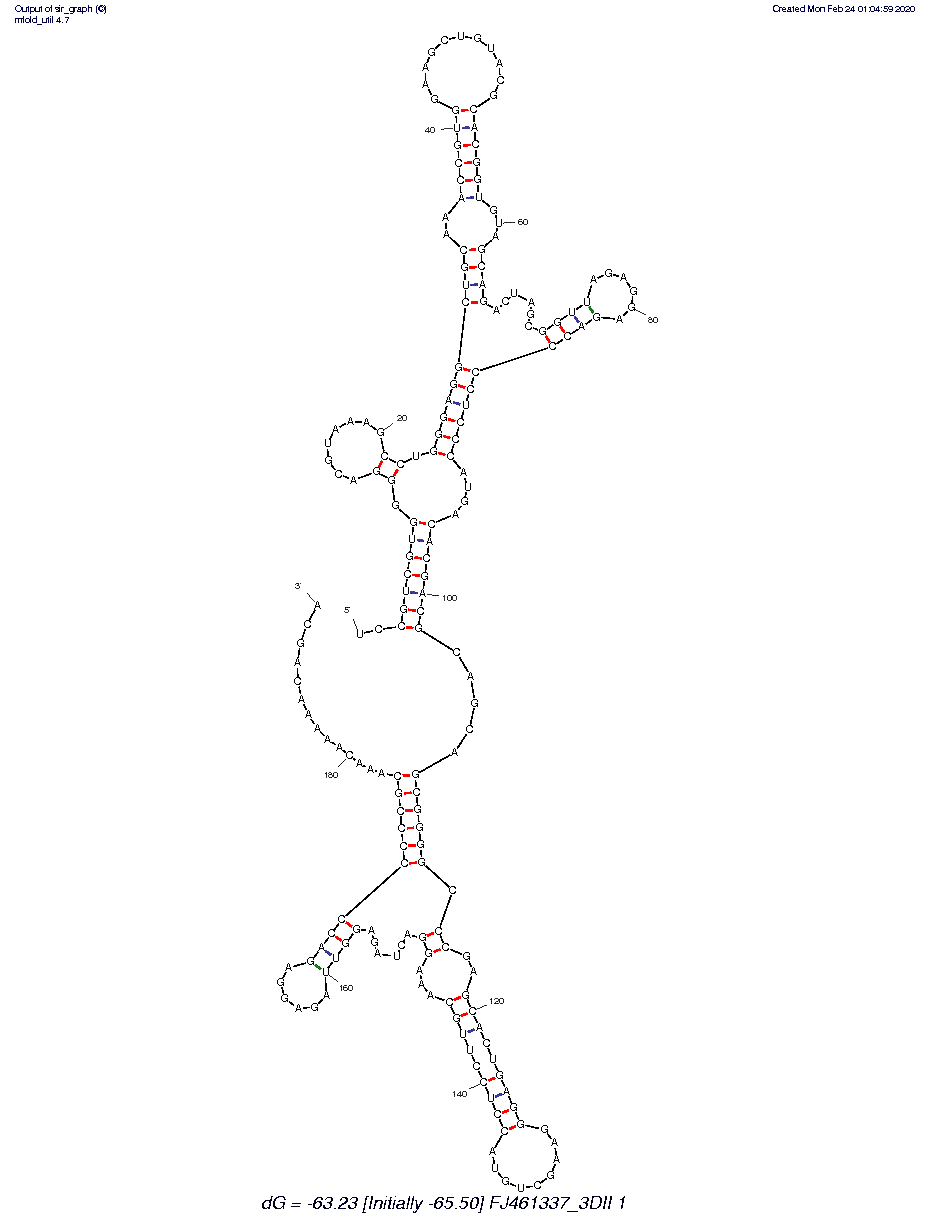 DB2  DB1 | -65.50  kcal/mol | | 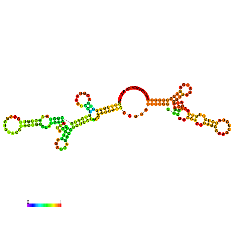  DB2  DB1 | -64.82  kcal/mol | 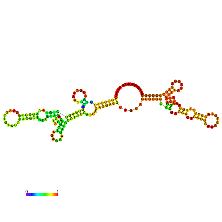 |
|  | DQ863638 | 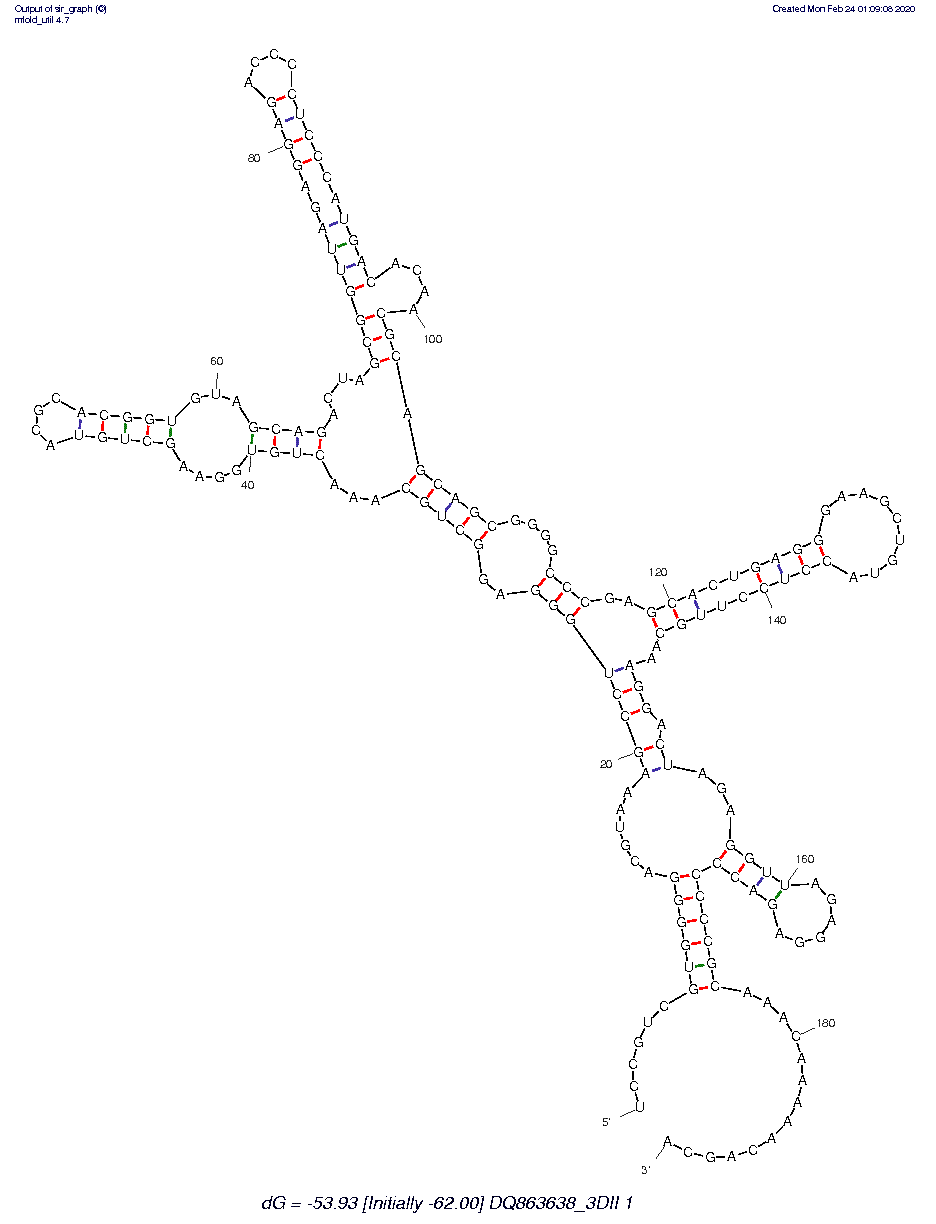 DB1  DB2 | -62.00  kcal/mol | 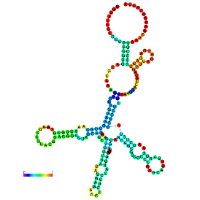  DB2  DB1 | | -61.29  kcal/mol | 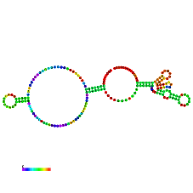 |
|  | EU482453  EU482459  EU482461 | 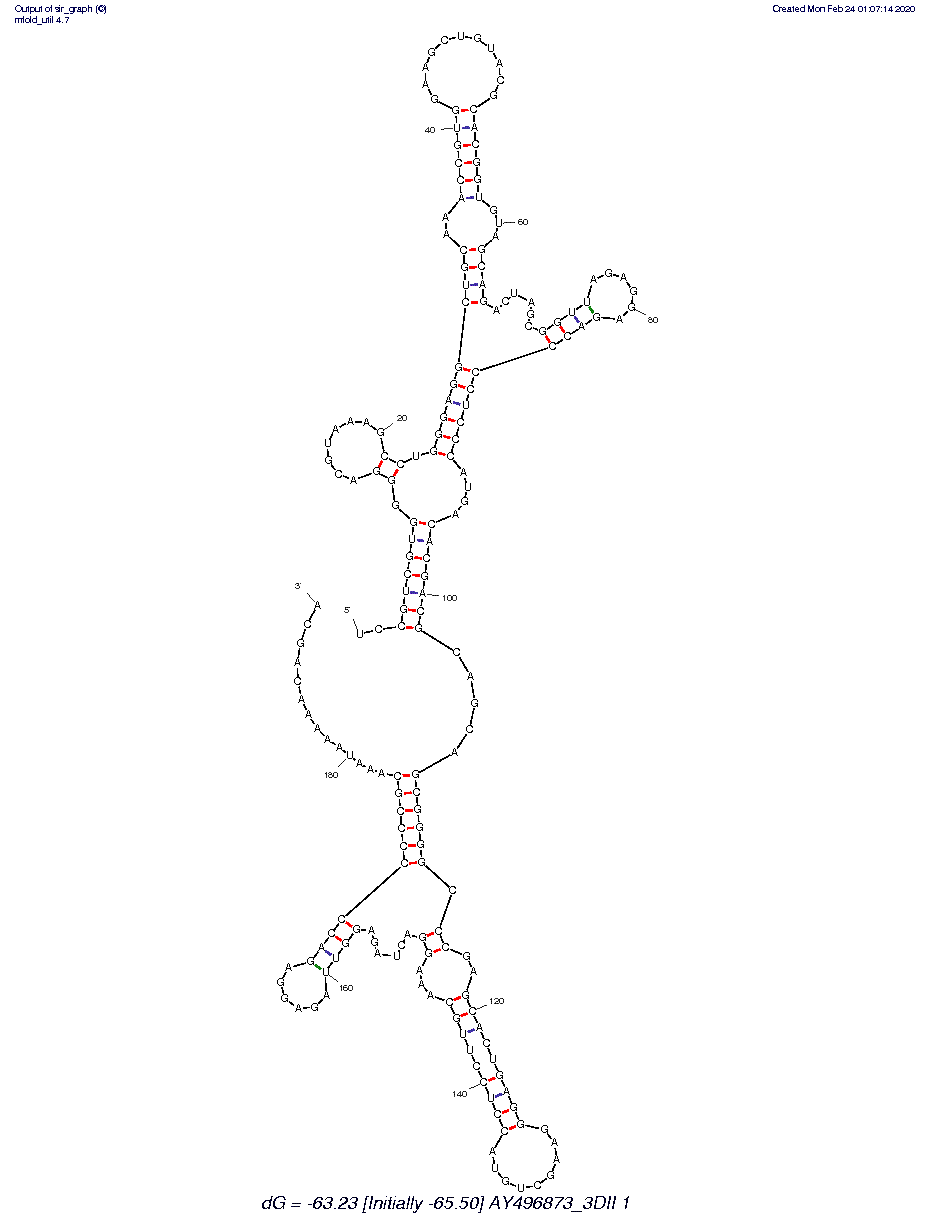 DB1  DB2 | -65.50  kcal/mol | 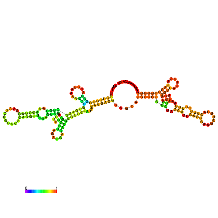  DB2  DB1 | | -64.80  kcal/mol | 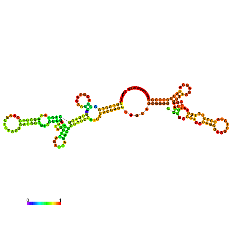 |
| **DENV3** | | **Mfold predicted secondary structures** | **RNAfold predicted secondary structures** | | | | |
|  |  | **MFE structure** | **MFE structure** | | | **Centroid structure** | |
| **DENV3 Genotype II** | KY849770  KY849769  KY849773  KY849775 | 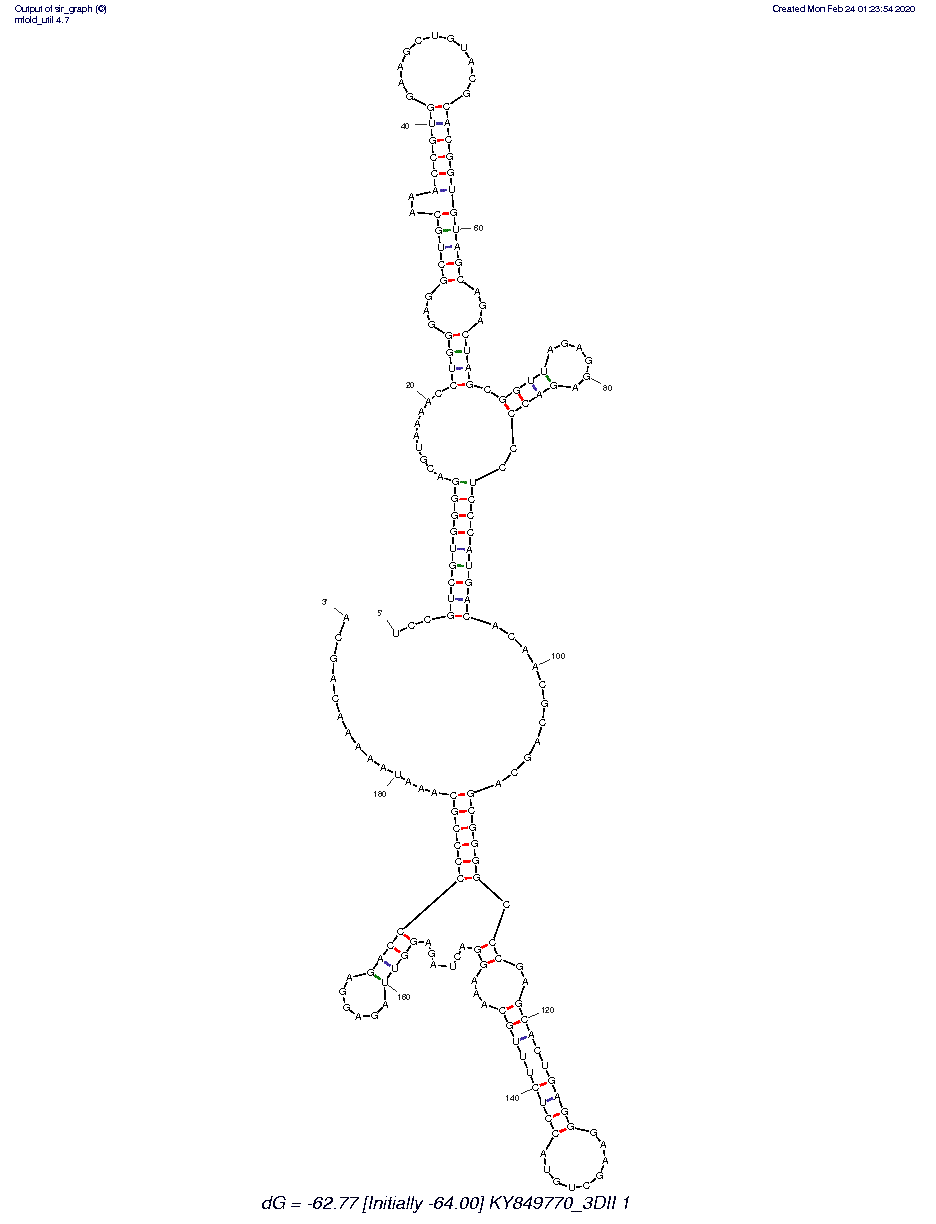 DB1  DB2 | -64.00  kcal/mol | 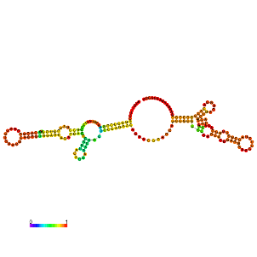  DB2  DB1 | | -62.83  kcal/mol | 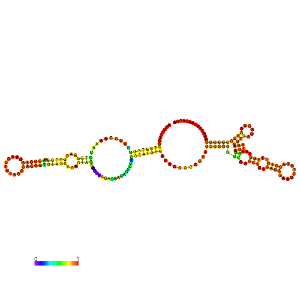 |
|  | KJ622198  KJ622197 | 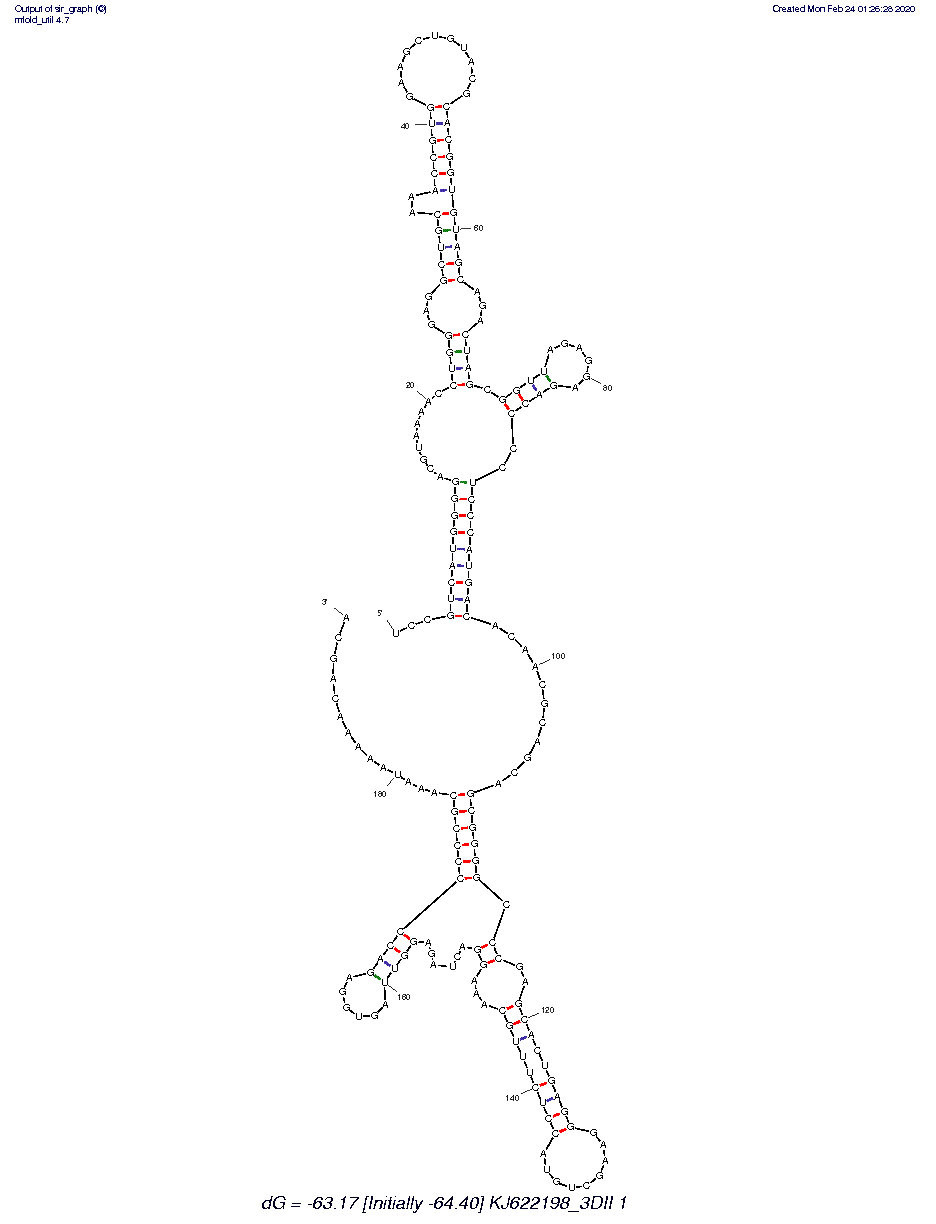 DB1  DB2 | -64.40  kcal/mol | 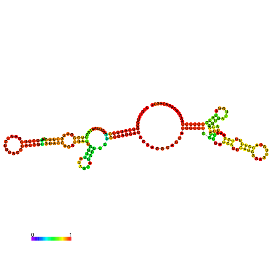  DB2  DB1 | | -63.30  kcal/mol | 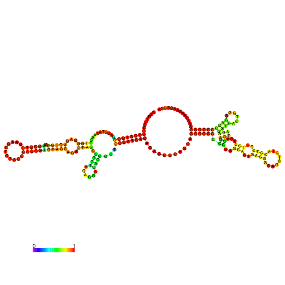 |
|  | KR296743  KF824904  KF824902 | 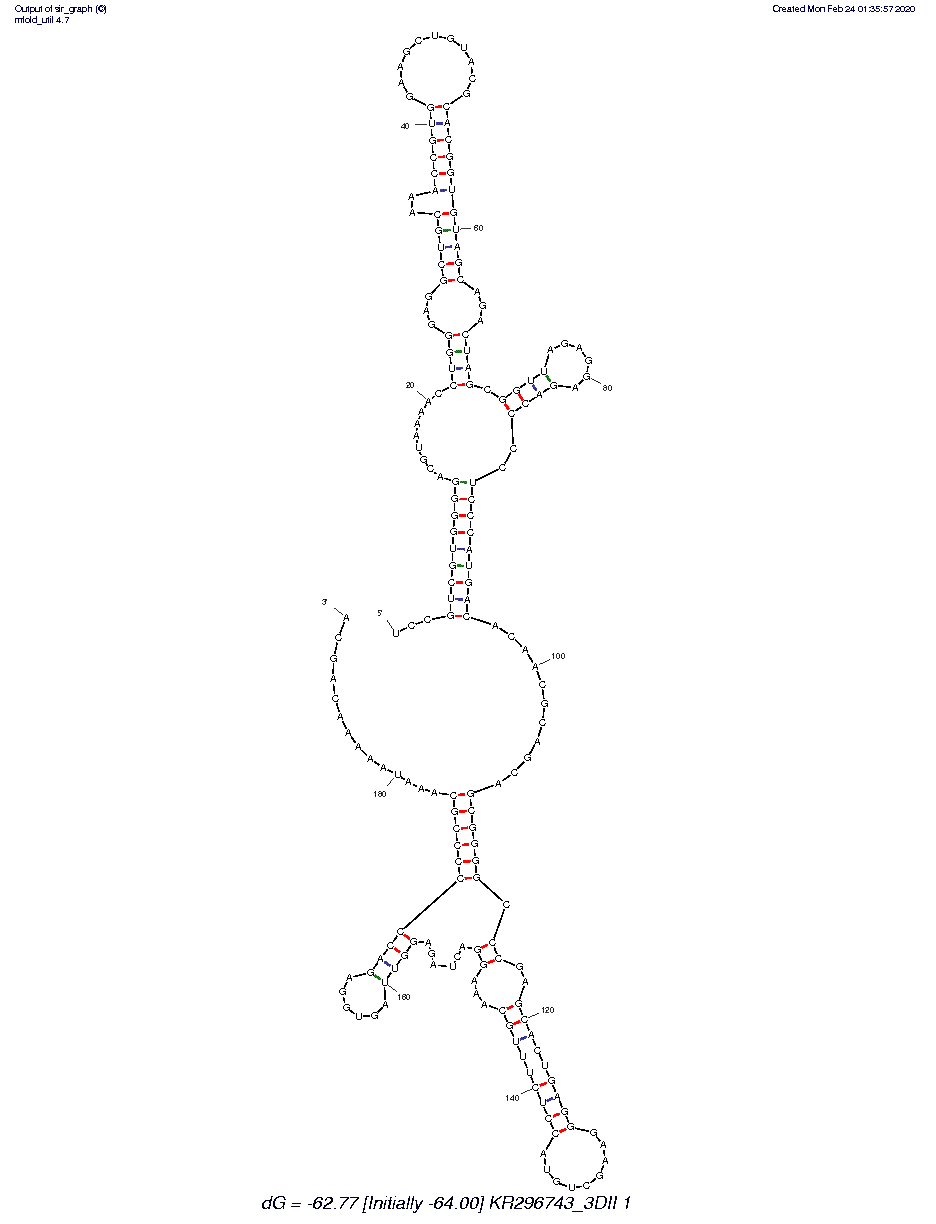 DB1  DB2 | -64.00  kcal/mol | 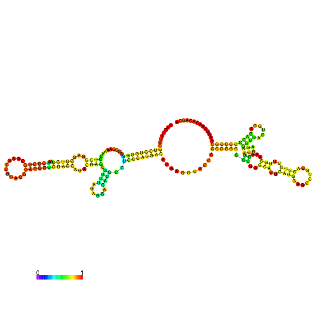  DB2  DB1 | | -63.02  kcal/mol | 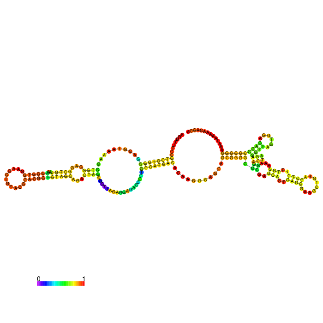 |
|  | FJ744726 | 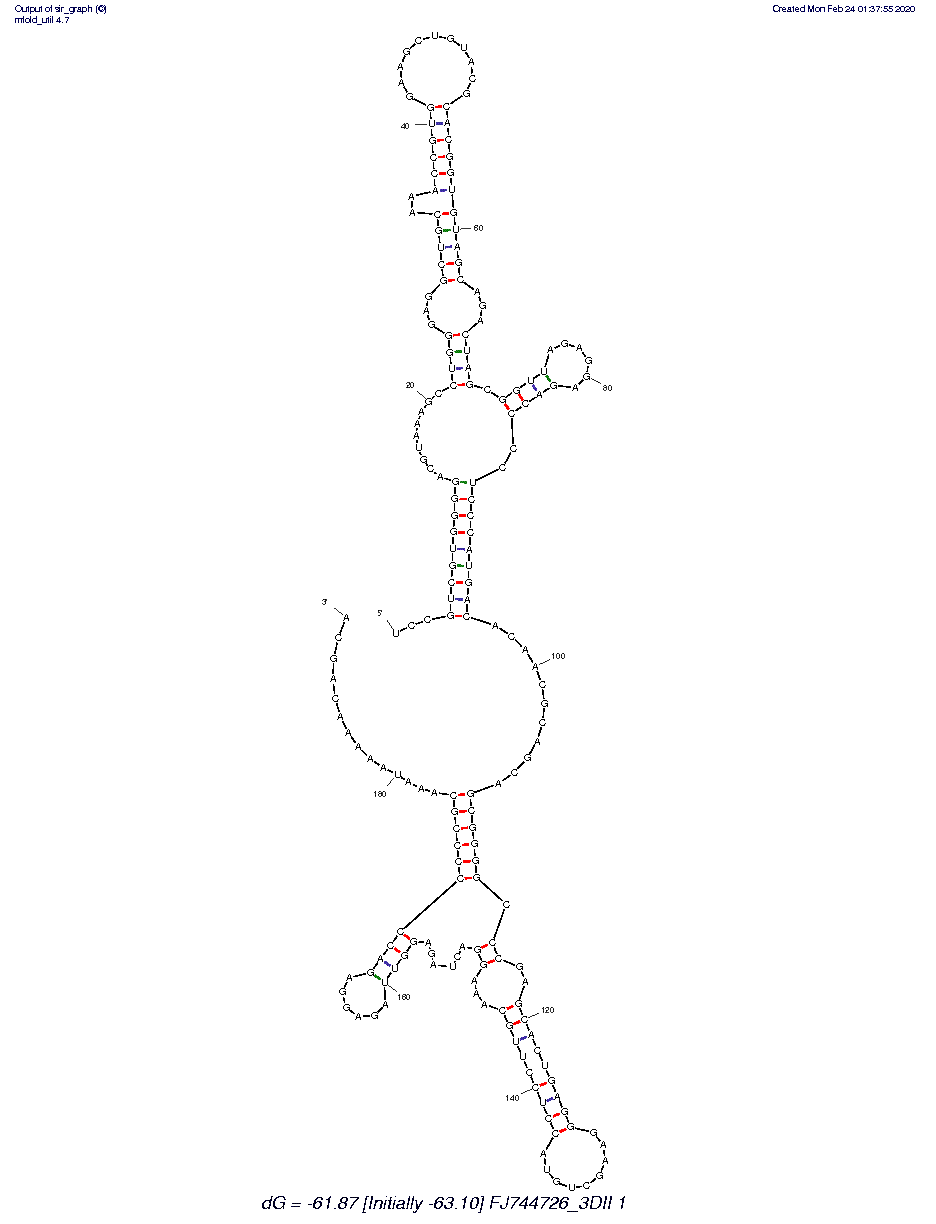 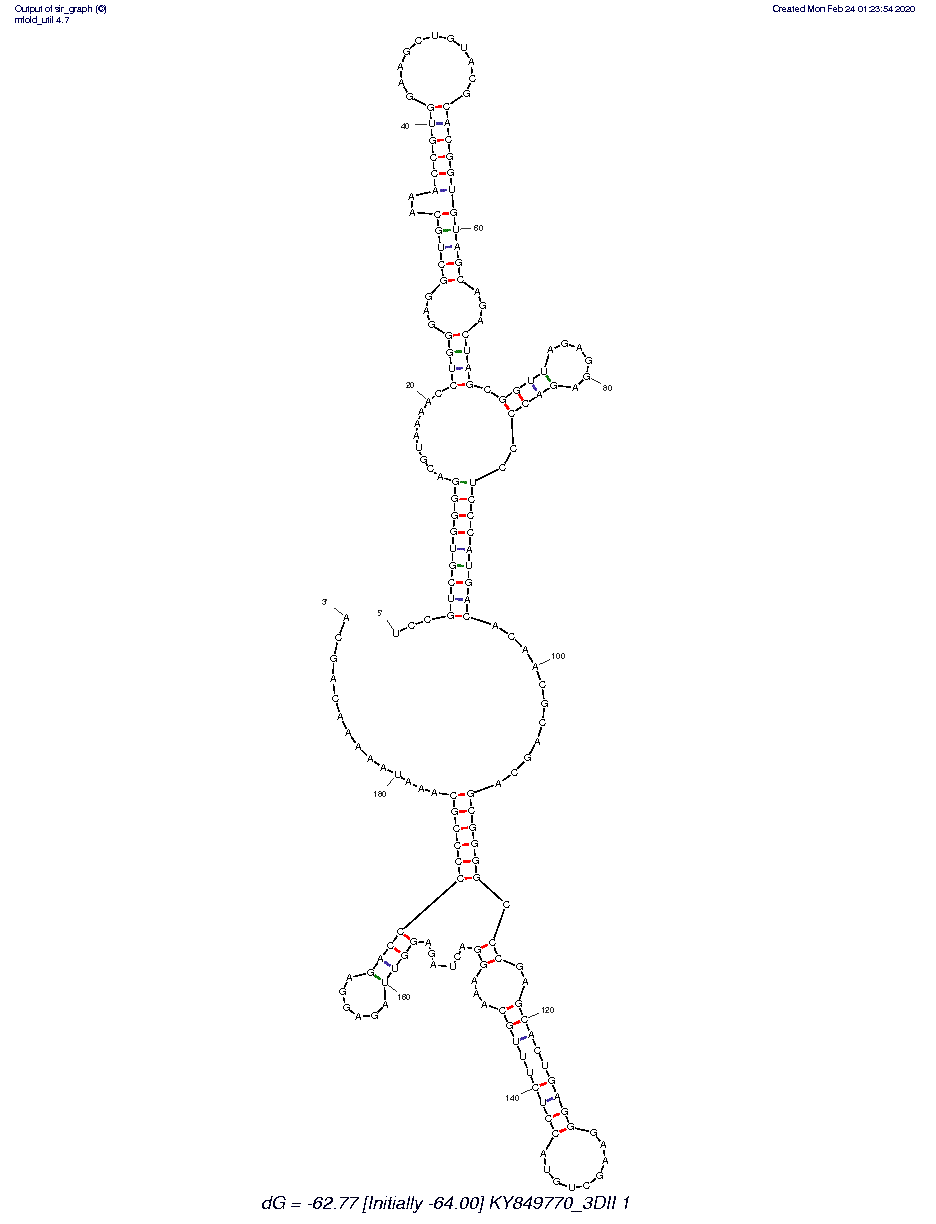 DB1  DB2 | -63.10  kcal/mol | 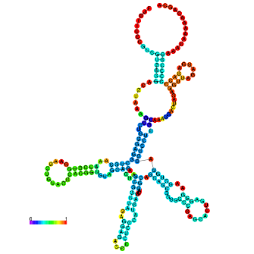  DB2  DB1 | | -63.34  kcal/mol | 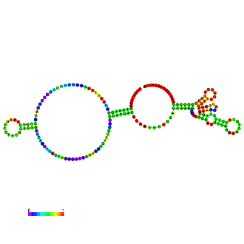 |
| **DENV3** | | **Mfold predicted secondary structures** | **RNAfold predicted secondary structures** | | | | |
|  |  | **MFE structure** | **MFE structure** | | | **Centroid structure** | |
| **DENV3 Genotype III** | EU081182 | 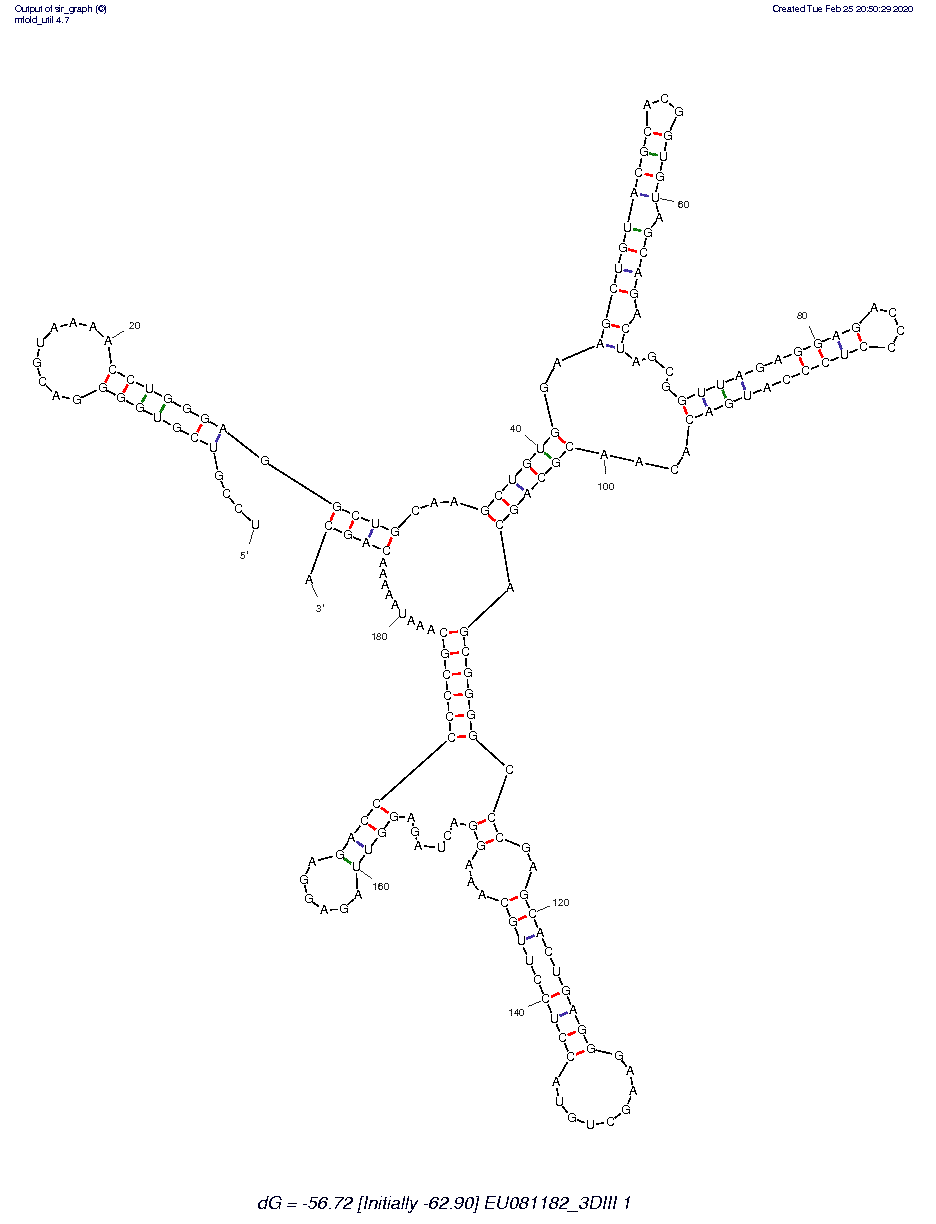 DB1  DB2 | -62.90  kcal/mol | 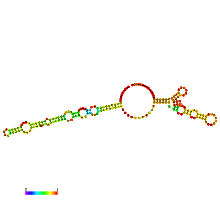  DB2  DB1 | | -61.27  kcal/mol | 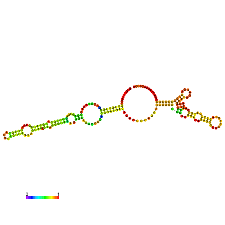 |
|  | AY662691 | 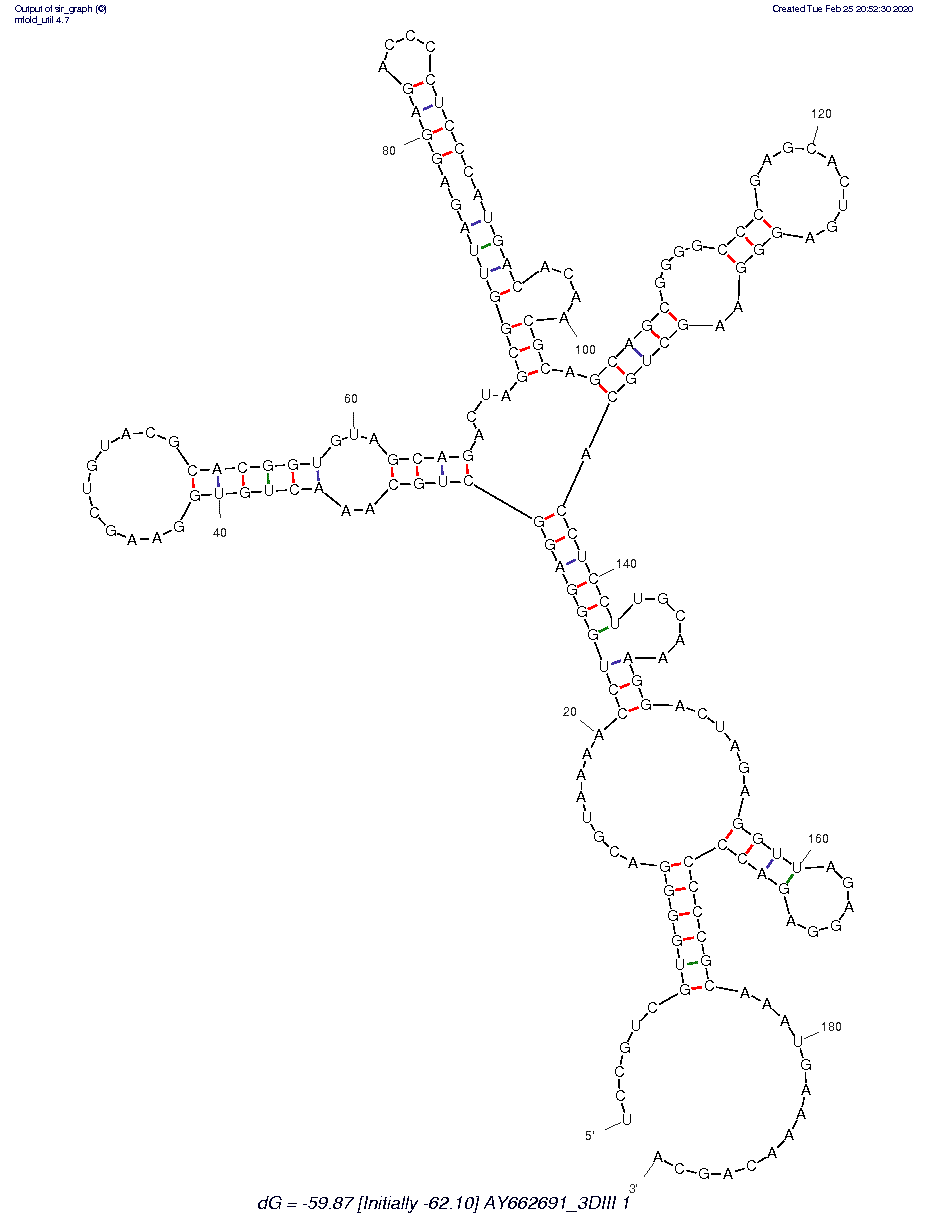 DB1  DB2 | -62.10  kcal/mol | 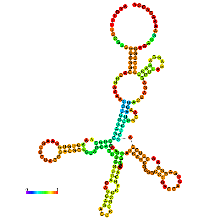  DB2  DB1 | | -61.76  kcal/mol | 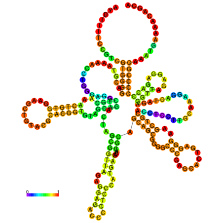 |
|  | GU131872 | 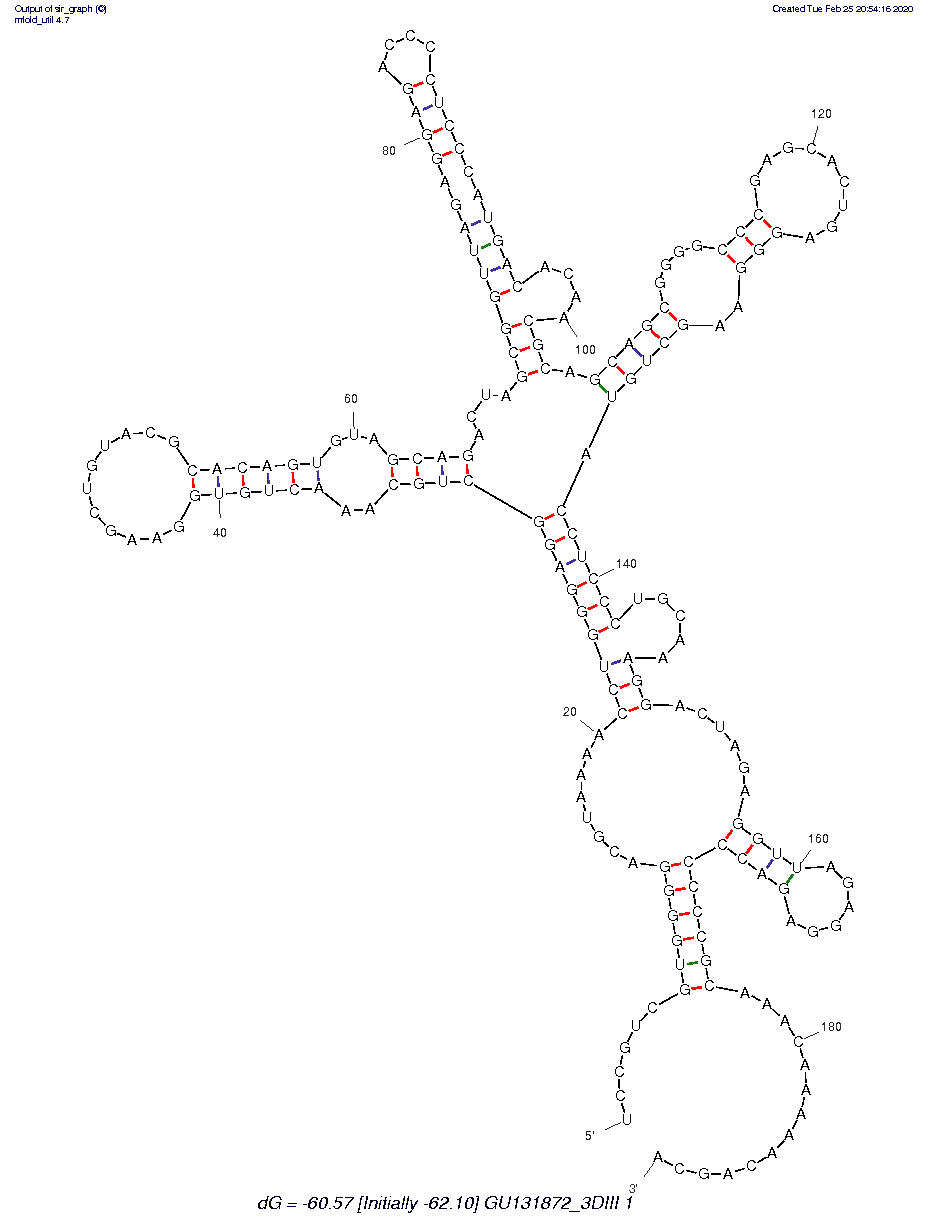 DB1  DB2 | -62.10  kcal/mol | 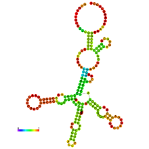  DB2  DB1 | | -61.96  kcal/mol | 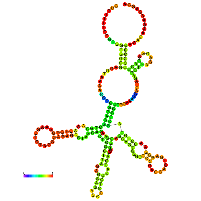 |
|  | FJ882573 | 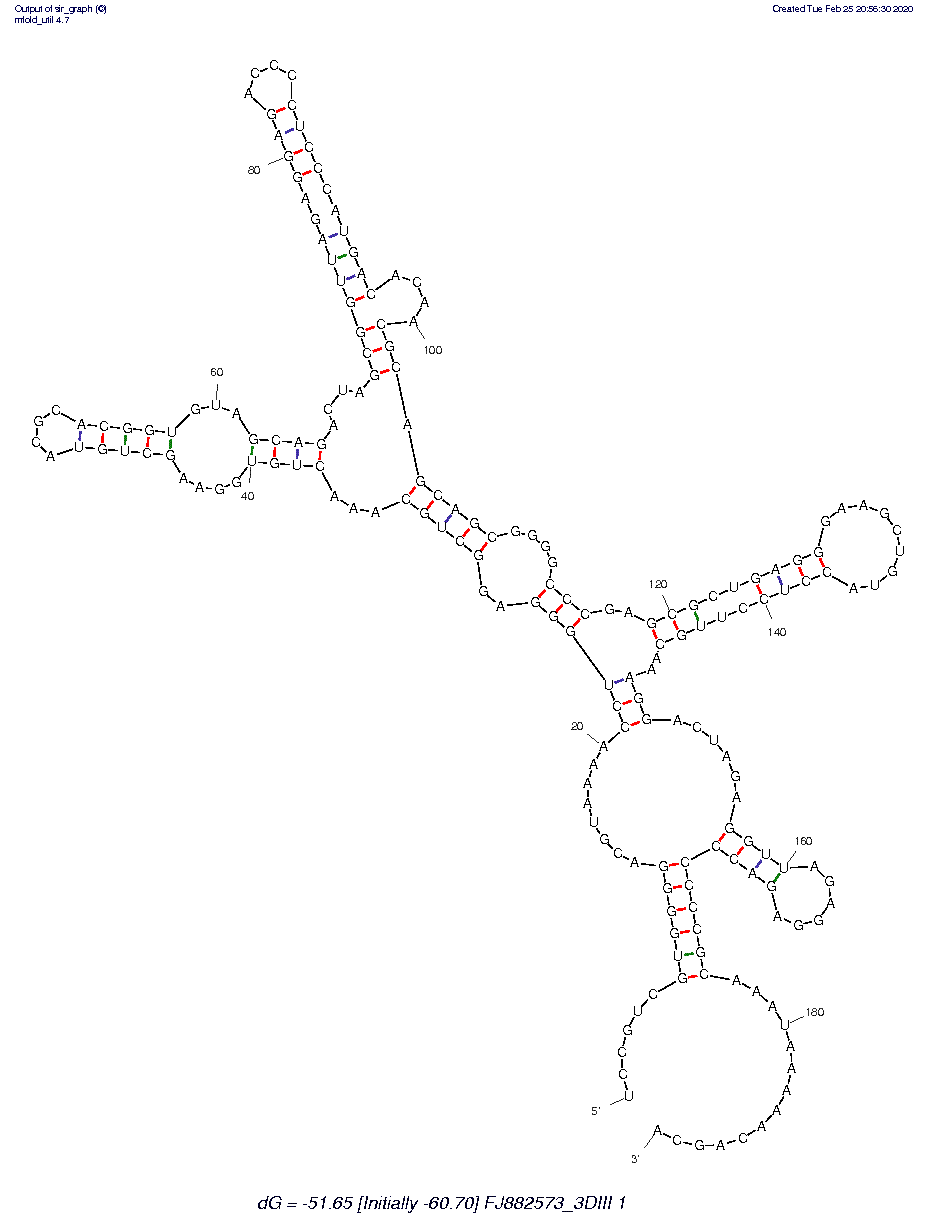   DB1  DB2 | -60.70  kcal/mol | 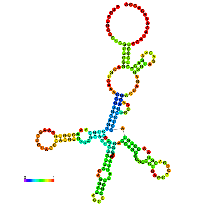  DB2  DB1 | | -59.71  kcal/mol | 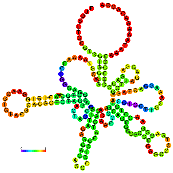 |
|  | EU529691 | 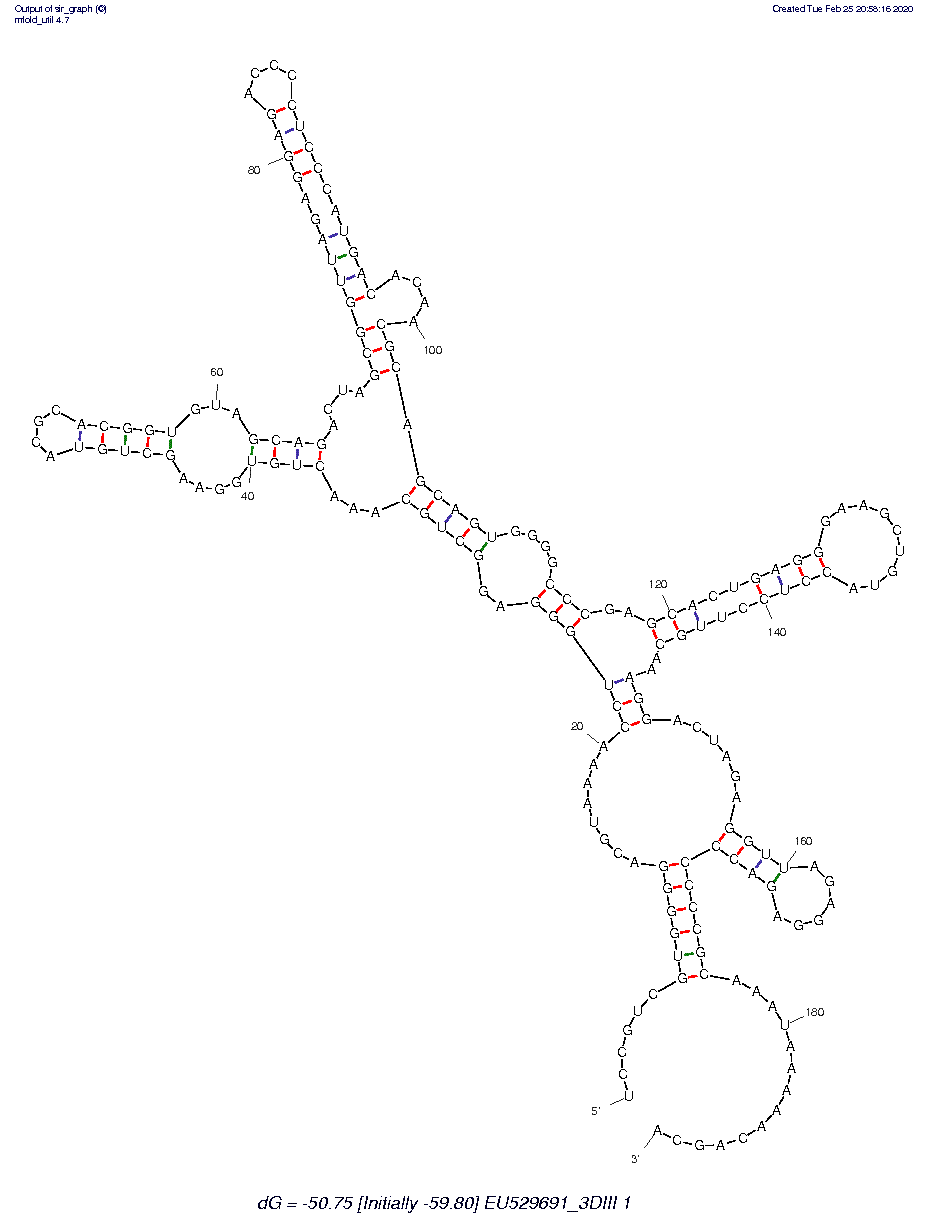 DB1  DB2 | -59.80  kcal/mol | 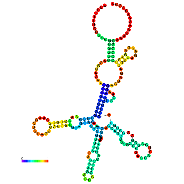  DB2  DB1 | | -58.34  kcal/mol | 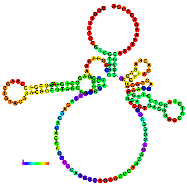 |
| **DENV3** | | **Mfold predicted secondary structures** | **RNAfold predicted secondary structures** | | | | |
|  |  | **MFE structure** | **MFE structure** | | | **Centroid structure** | |
| **DENV3 Genotype III** | FJ898440 | 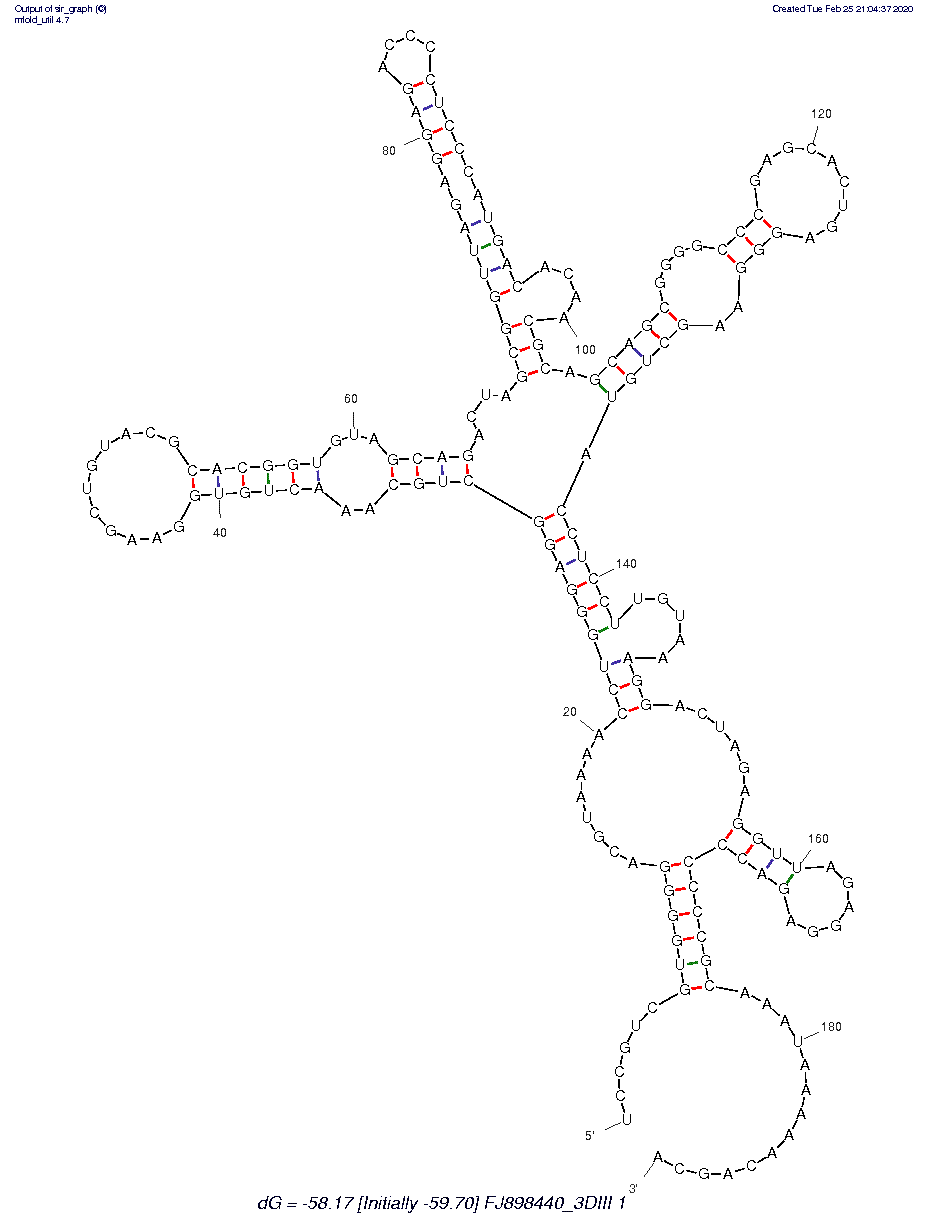 DB1  DB2 | -59.70  kcal/mol | 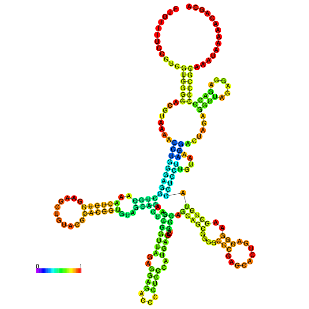  DB2  DB1 | | -59.63  kcal/mol | 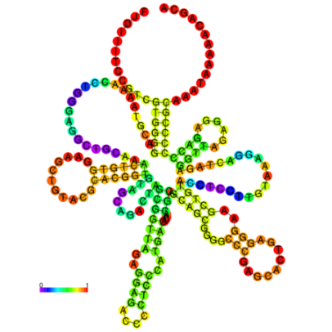 |
|  | JF808129 | 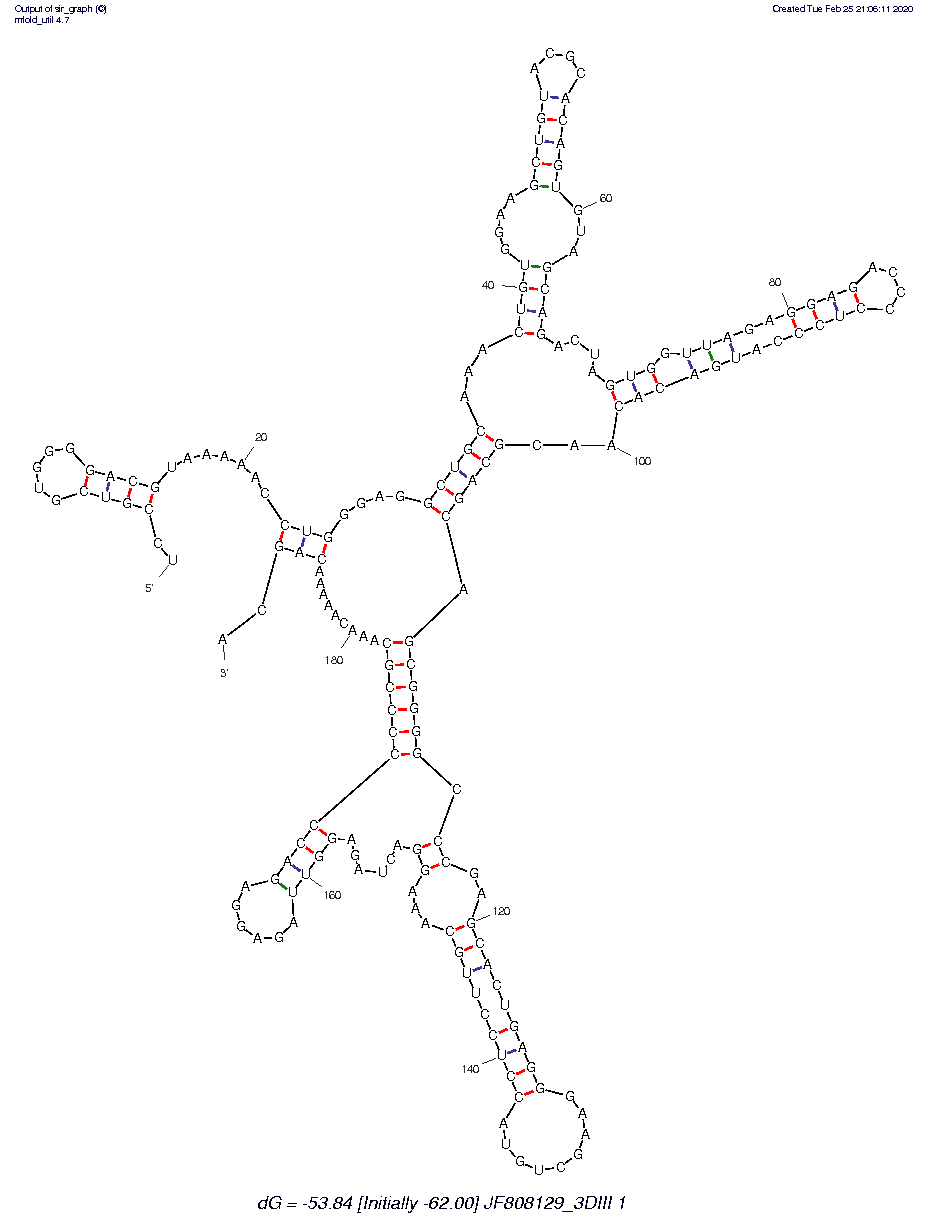 DB1  DB2 | -62.00  kcal/mol | 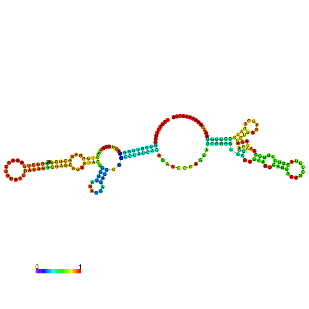  DB2  DB1 | | -60.98  kcal/mol | 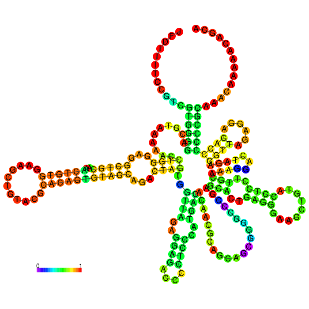 |
|  | JF504679 | 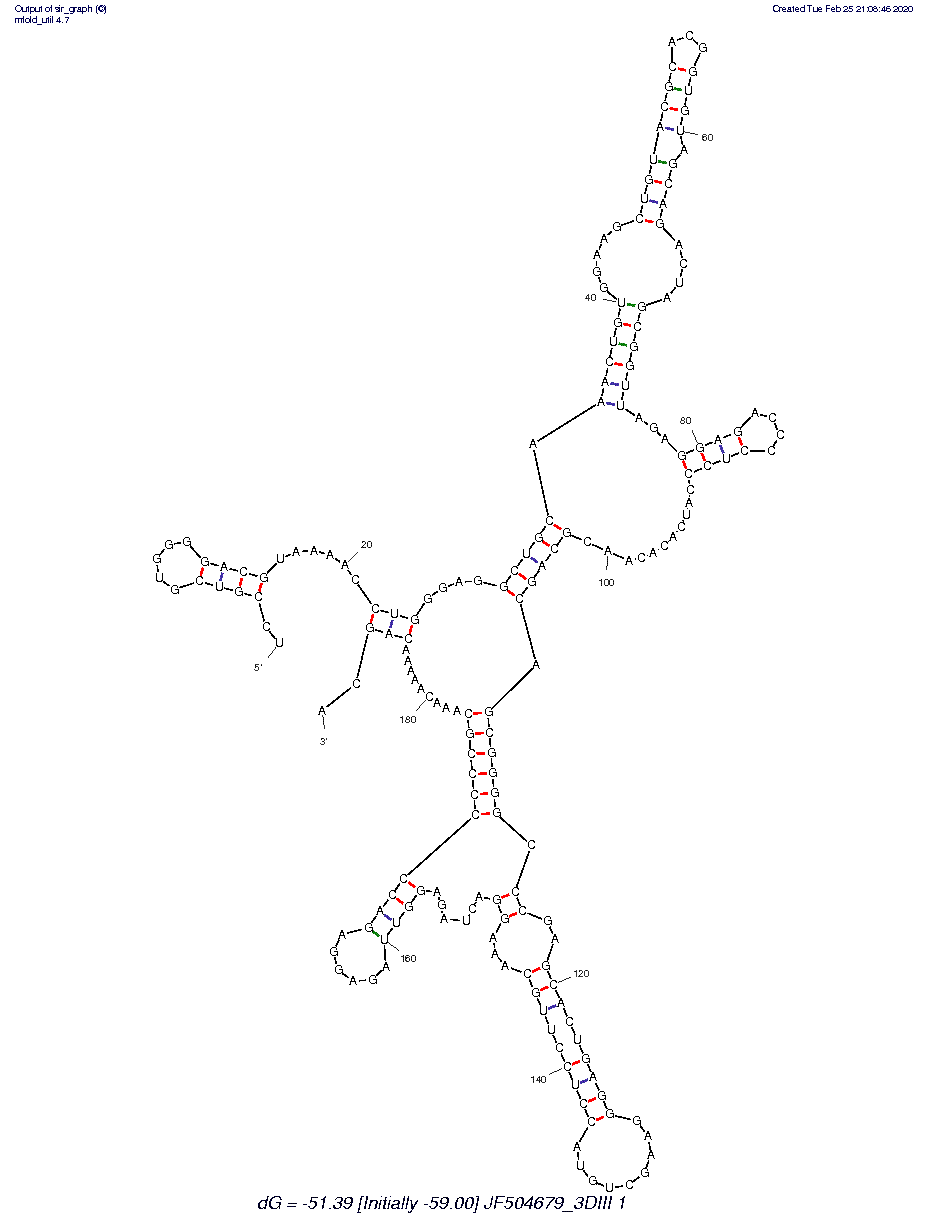 DB1  DB2 | -59.00  kcal/mol | 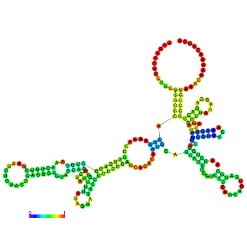  DB2  DB1 | | -58.71  kcal/mol | 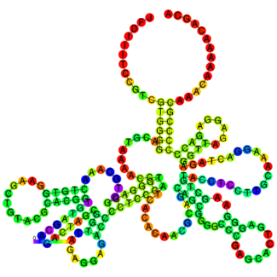 |
| **DENV3** | | **Mfold predicted secondary structures** | **RNAfold predicted secondary structures** | | | | |
|  |  | **MFE structure** | **MFE structure** | | | **Centroid structure** | |
| **DENV3 Genotype III** | KF954945 | 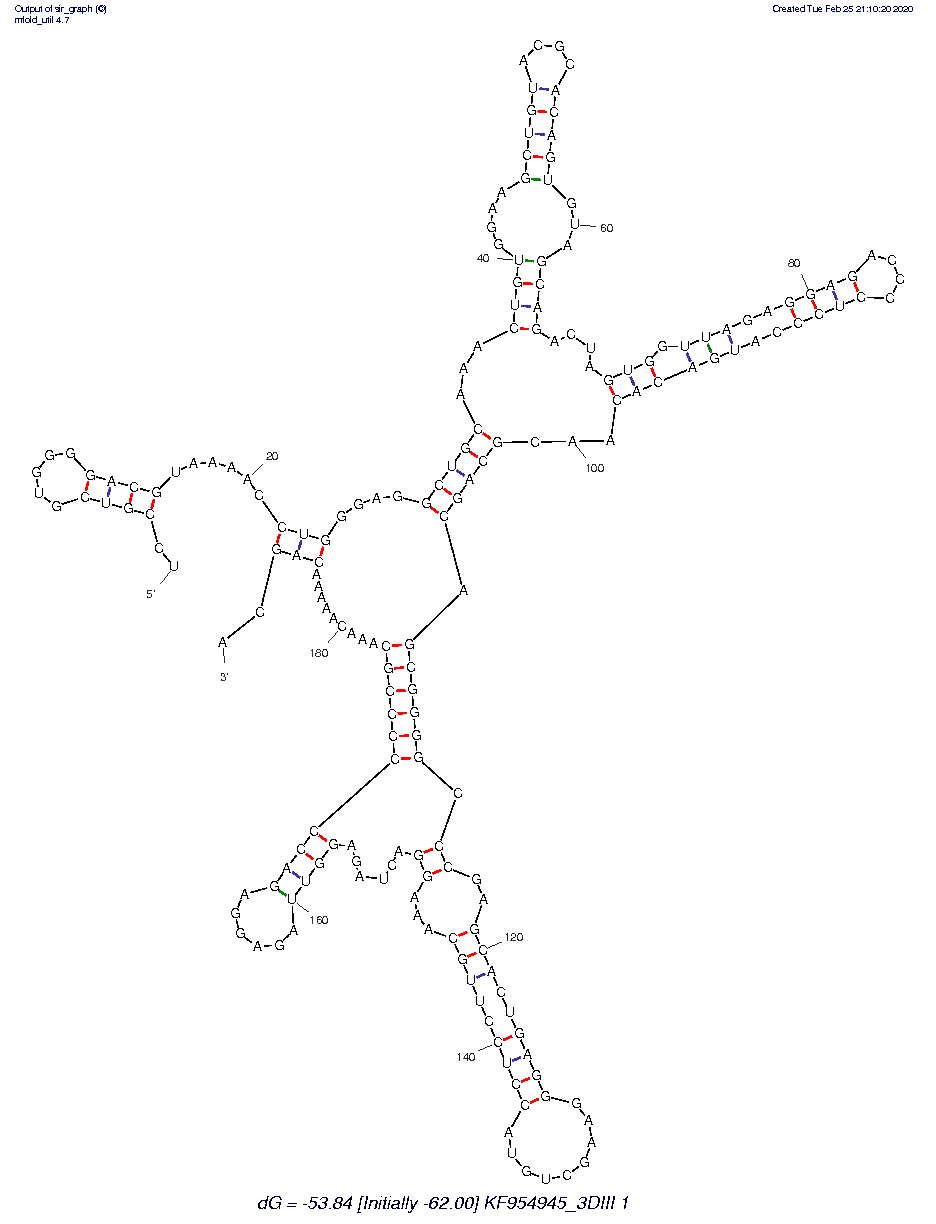 DB1  DB2 | -62.00  kcal/mol | 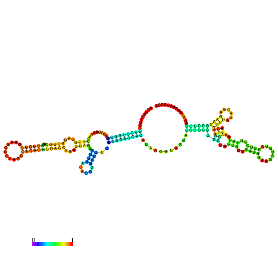  DB2  DB1 | | -60.91  kcal/mol | 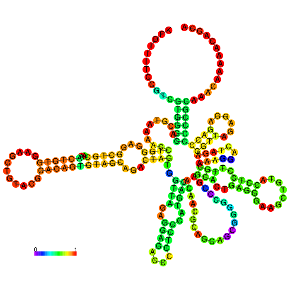 |
|  | FJ182013  GQ868571  AY099336 | 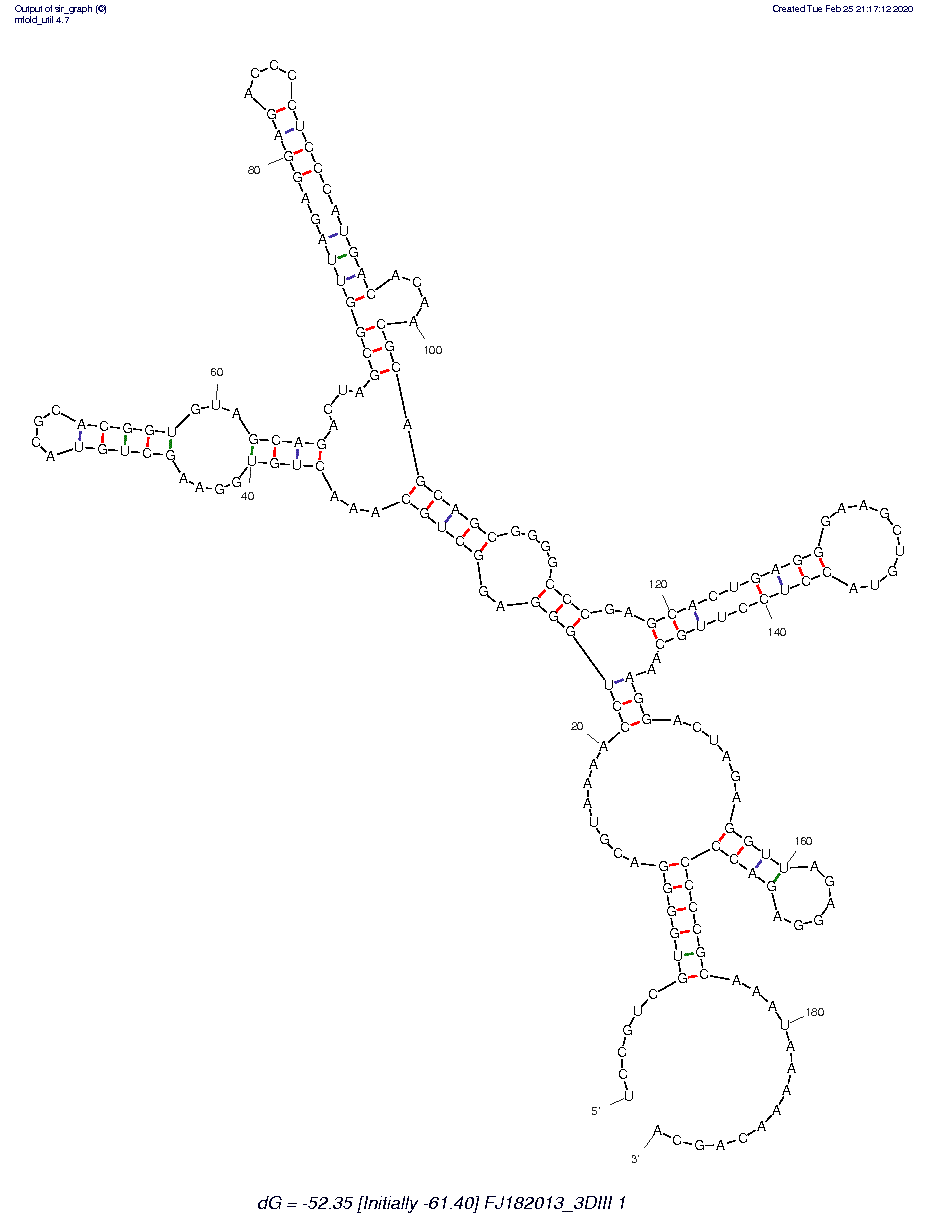 DB1  DB2 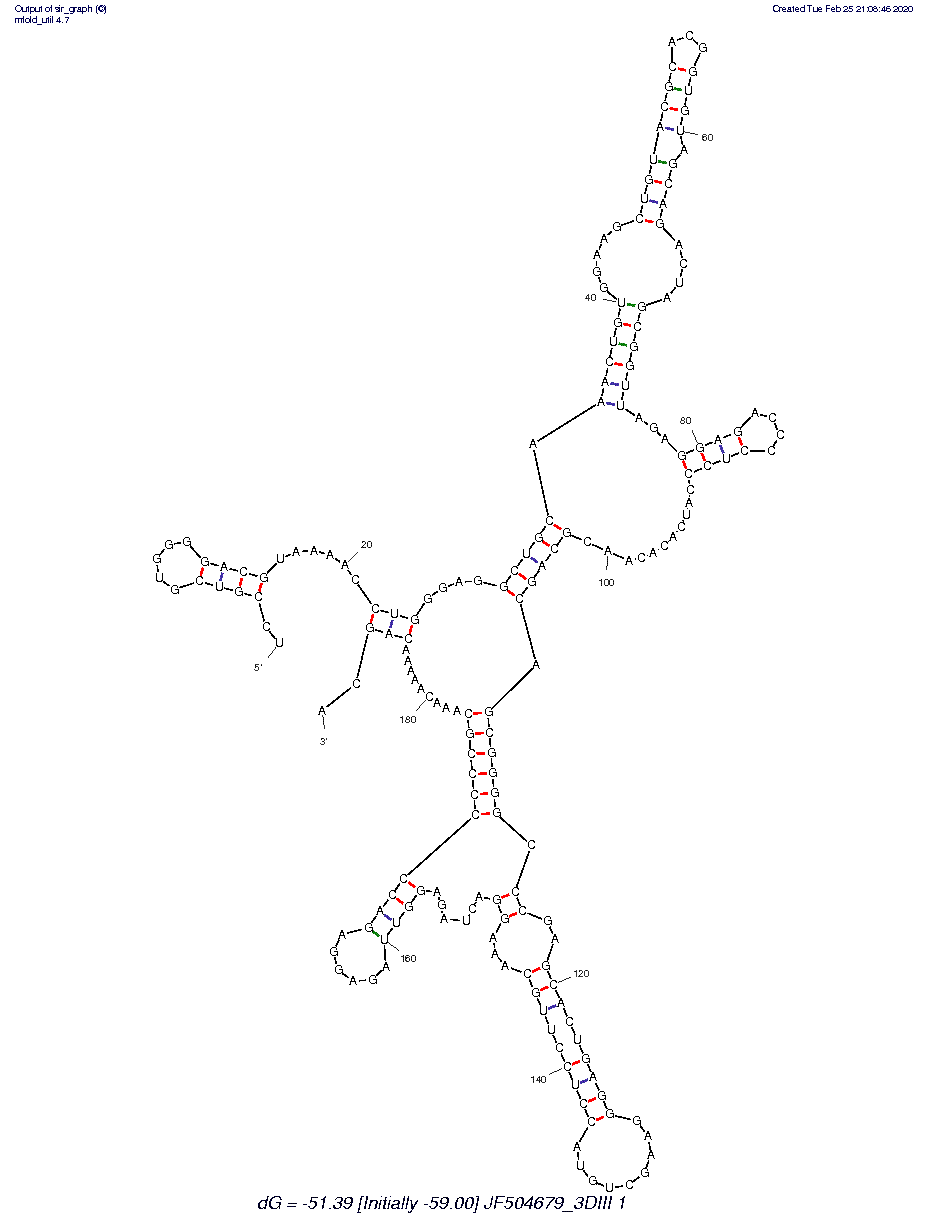 DB1  DB2 | -61.40  kcal/mol | 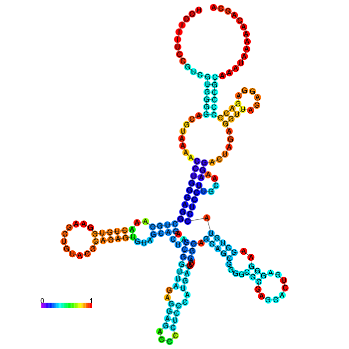  DB2  DB1 | | -60.09  kcal/mol |  |
|  | HQ705618  KJ643590  KT726350 | DB1  DB2 | -62.10  kcal/mol | DB1  DB2 | | -60.72  kcal/mol |  |
| **DENV3** | | **Mfold predicted secondary structures** | **RNAfold predicted secondary structures** | | | | |
|  |  | **MFE structure** | **MFE structure** | | | **Centroid structure** | |
| **DENV3 Genotype V** | KU509282  KU509695  JN697379 | DB2  DB1 | -63.30 kcal/mol | DB1  DB2 | | -62.711 kcal/mol |  |

*SNVs for each DENV3 isolate structure is highlighted in red on each Mfold Predicted structure*. *Base pairing probability in MEF and Centroid structures of RNA predicted secondary structures, is denoted by the colored nucleotides. Colours are rated from 1-0 to indicate strong to weak base pairing probabilities (Red - strongest probability, Green - medium probability, Blue - lowest probability) in the colour scale.*
