# Supplementary material for: Genetic Variation in the Domain II, 3′ Untranslated Region of Human and Mosquito Derived Dengue Virus Strains in Sri Lanka
Source: Viruses. 2021 Mar 5;13(3):421. doi: 10.3390/v13030421 (PMC8001906; doi:10.3390/v13030421)
Supplement: Supplementary file 1 [file viruses-13-00421-s001.zip › Supplimentry files/Supplimentry tables/Table S6.pdf]

Table S6. Mfold and RNAfold predicted secondary structures for RNA alignments of DENV3, Domain II region of 3'UTR sequences identified in the study, Sri Lankan isolates and DENV1 reference genotypes.

**DENV3**		**Mfold predicted secondary structures**	**RNAfold predicted secondary structures**			
		**MFE structure**	**MFE structure**		**Centroid structure**	
**DENV3 reference**	M93130	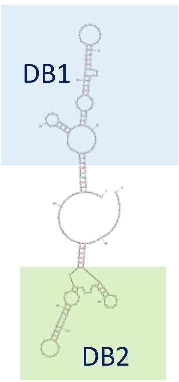	-63.30 kcal/mol	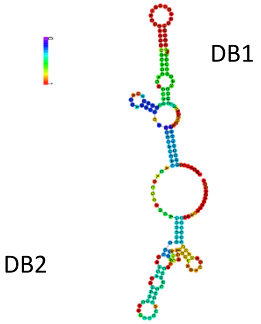	-62.711 kcal/mol	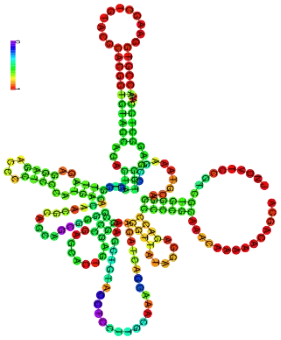
**Study**	D1H_2019SL	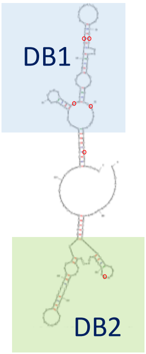	-60.77 kcal/mol	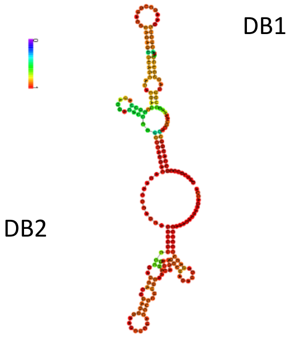	-60.78 kcal/mol	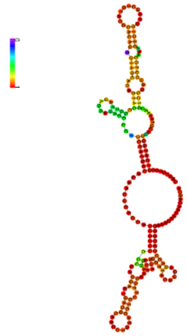

DENV3		Mfold predicted secondary structures	RNAfold predicted secondary structures			
		MFE structure	MFE structure		Centroid structure	
Sri Lankan isolates of DENV3	AY585848<br>FJ882573	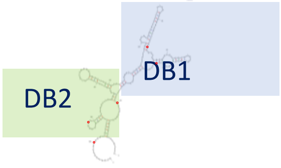	-60.77 kcal/mol	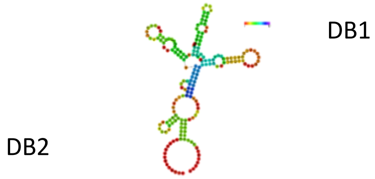	-59.71 kcal/mol	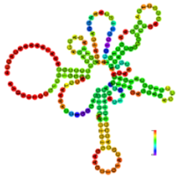
	AY85845<br>AY585846<br>GQ199886<br>AY585847<br>GQ199888<br>GQ199887<br>FJ582574<br>FJ882574	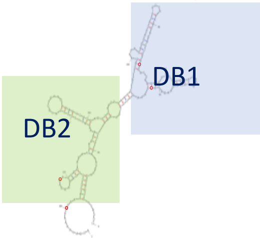	-62.00 kcal/mol	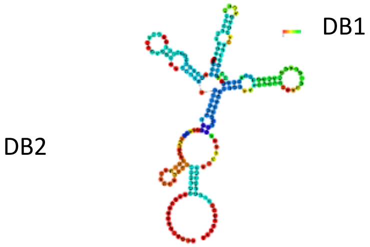	-61.23 kcal/mol	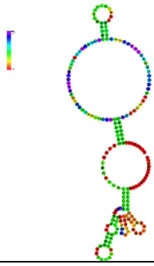
	GQ252674	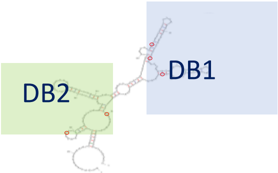	-61.30 kcal/mol	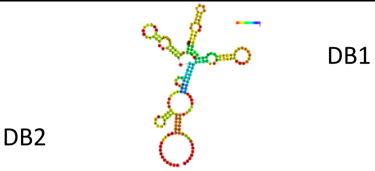	-59.61 kcal/mol	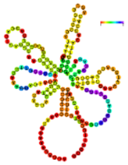
	AY099336<br>GQ199889<br>AY585851<br>AY585852	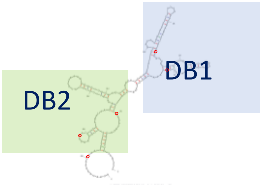	-61.40 kcal/mol	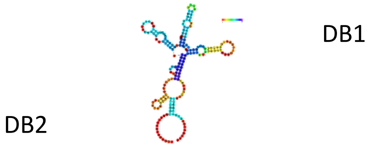	-60.09 kcal/mol	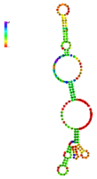
	JQ411814<br>FJ882571<br>KF955474	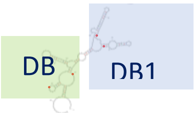	-61.40 kcal/mol	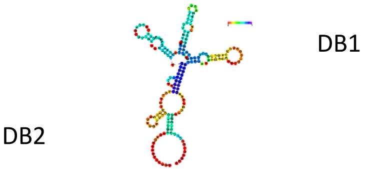	-62.71 kcal/mol	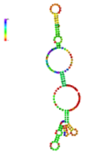

DENV3		Mfold predicted secondary structures	RNAfold predicted secondary structures			
		MFE structure	MFE structure		Centroid structure	
**DENV3 Genotype I**	AB189125	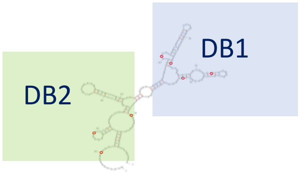	60.88 kcal/mol	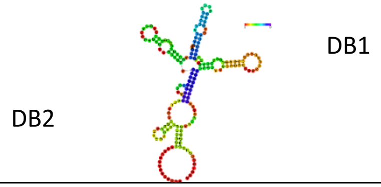	-59.52 kcal/mol	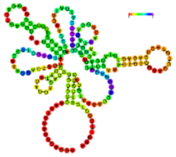
	AY648961	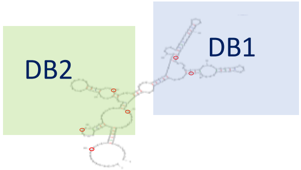	-59.80 kcal/mol	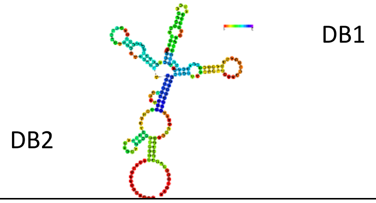	-59.99 kcal/mol	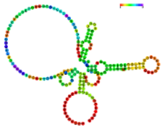
	AY858037	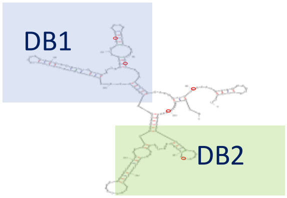	-62.00 kcal/mol	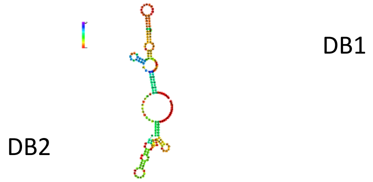	-60.84 kcal/mol	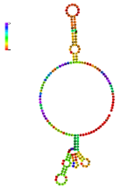
	AB189128<br>AY858046	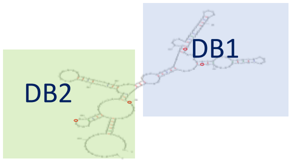	-61.40 kcal/mol	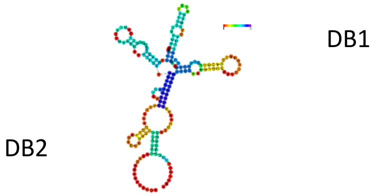	-60.15 kcal/mol	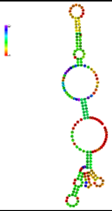
	DQ401690	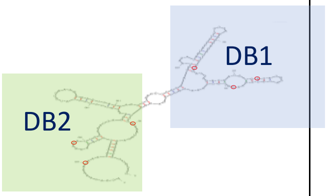	-62.10 kcal/mol	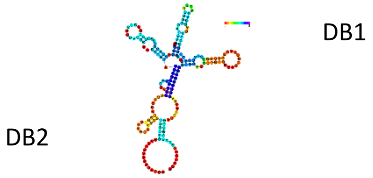	-60.72 kcal/mol	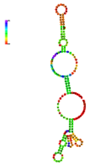

DENV3		Mfold predicted secondary structures	RNAfold predicted secondary structures			
		MFE structure	MFE structure		Centroid structure	
DENV3 Genotype I	AY858043<br>AY858038<br>EU081223<br>AY858041	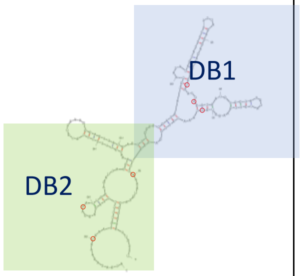	-61.40 kcal/mol	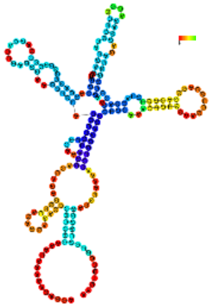 DB1 DB2	-60.09 kcal/mol	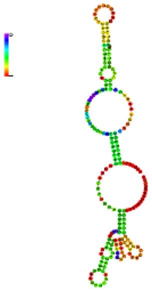
DENV3 Genotype I	KX380839	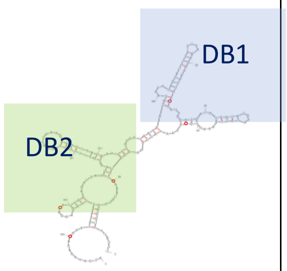	-61.4 kcal/mol	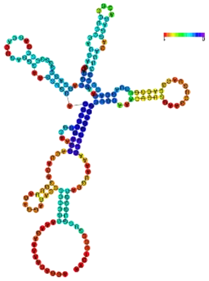 DB1 DB2	-60.13 kcal/mol	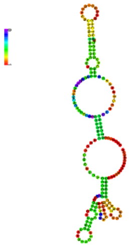
DENV3 Genotype I	AY744681	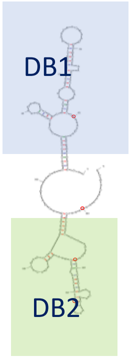	-63.4 kcal/mol	DB1 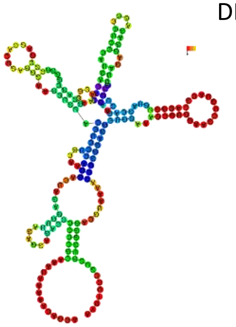 DB2	-63.03 kcal/mol	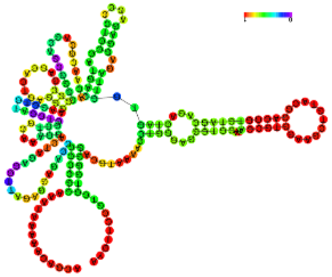

DENV3		Mfold predicted secondary structures	RNAfold predicted secondary structures			
		MFE structure	MFE structure		Centroid structure	
DENV3 Genotype II	KC261634	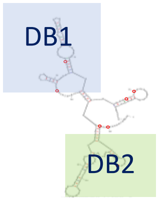	-60.70 kcal/mol	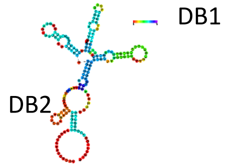	-63.34 kcal/mol	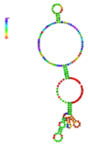
	FJ461337<br>AY876494<br>FJ744734<br>FJ687448<br>FJ744728<br>AY676350<br>AY496873	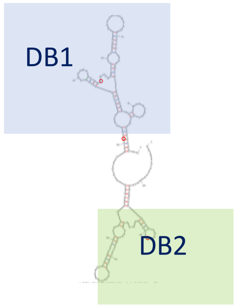	-65.50 kcal/mol	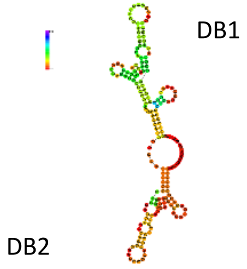	-64.82 kcal/mol	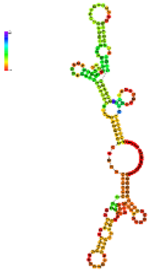
	DQ863638	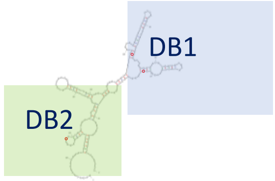	-62.00 kcal/mol	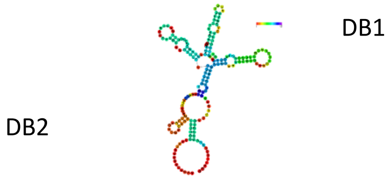	-61.29 kcal/mol	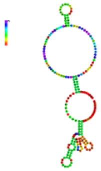
	EU482453<br>EU482459<br>EU482461	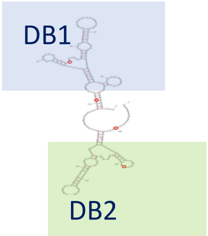	-65.50 kcal/mol	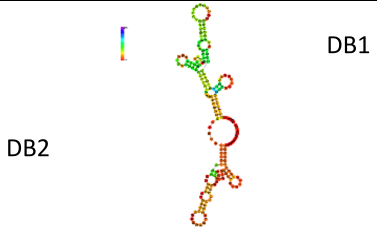	-64.80 kcal/mol	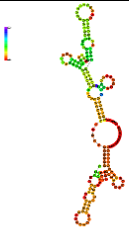

DENV3		Mfold predicted secondary structures	RNAfold predicted secondary structures			
		MFE structure	MFE structure		Centroid structure	
DENV3 Genotype II	KY849770<br>KY849769<br>KY849773<br>KY849775	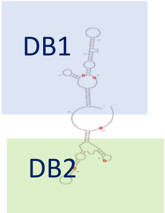	-64.00 kcal/mol	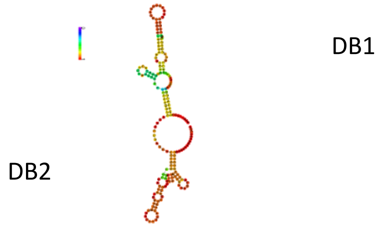	-62.83 kcal/mol	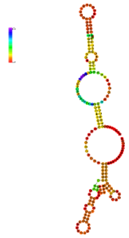
	KJ622198<br>KJ622197	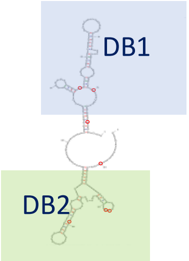	-64.40 kcal/mol	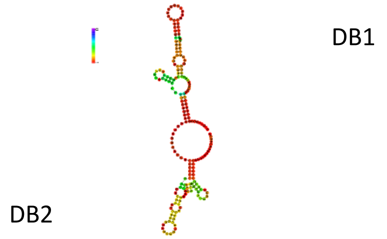	-63.30 kcal/mol	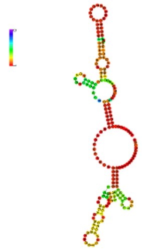
	KR296743<br>KF824904<br>KF824902	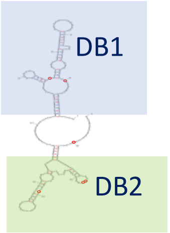	-64.00 kcal/mol	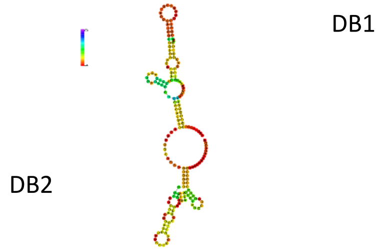	-63.02 kcal/mol	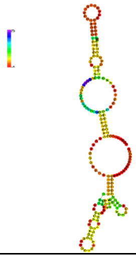
	FJ744726	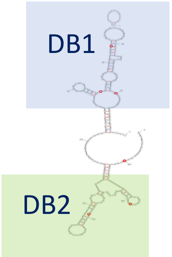	-63.10 kcal/mol	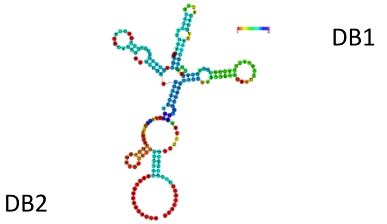	-63.34 kcal/mol	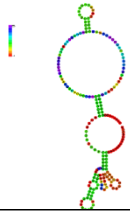

DENV3		Mfold predicted secondary structures	RNAfold predicted secondary structures			
		MFE structure	MFE structure		Centroid structure	
DENV3 Genotype III	EU081182	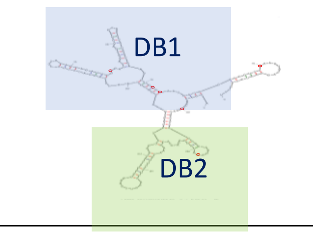	-62.90 kcal/mol	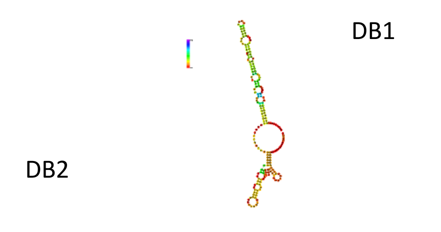	-61.27 kcal/mol	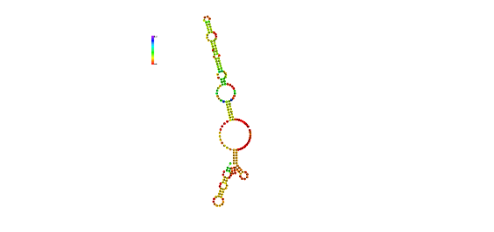
	AY662691	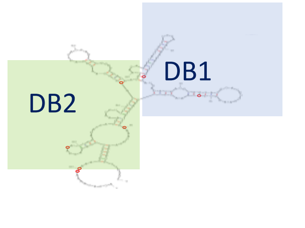	-62.10 kcal/mol	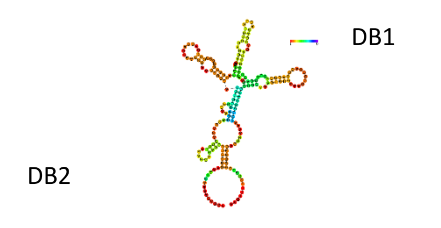	-61.76 kcal/mol	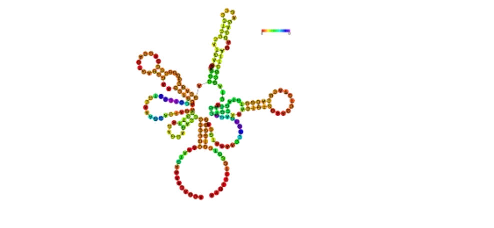
	GU131872	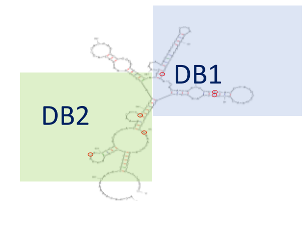	-62.10 kcal/mol	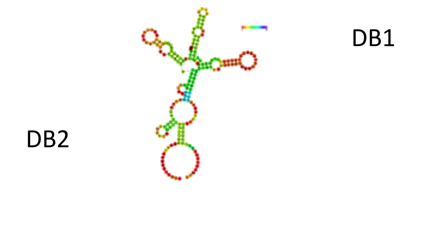	-61.96 kcal/mol	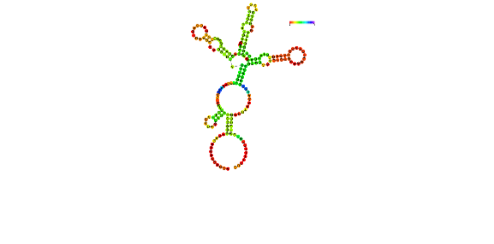
	FJ882573	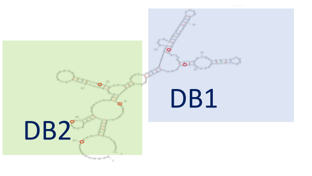	-60.70 kcal/mol	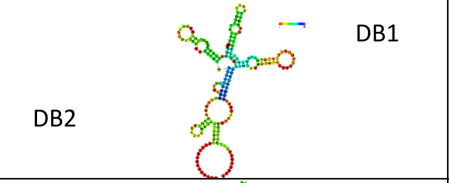	-59.71 kcal/mol	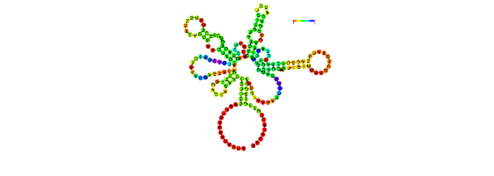
	EU529691	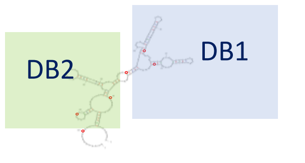	-59.80 kcal/mol	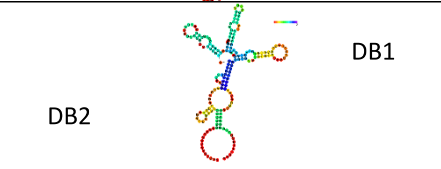	-58.34 kcal/mol	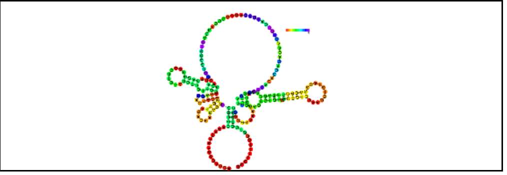

DENV3		Mfold predicted secondary structures	RNAfold predicted secondary structures			
		MFE structure	MFE structure		Centroid structure	
DENV3 Genotype III	FJ898440	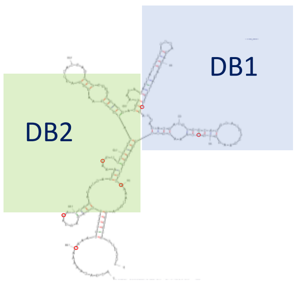	-59.70 kcal/mol	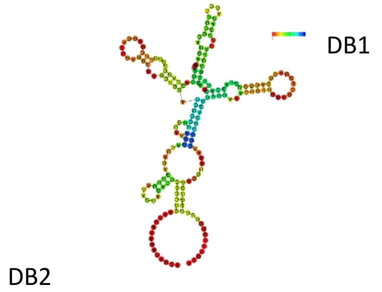	-59.63 kcal/mol	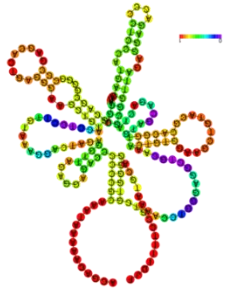
	JF808129	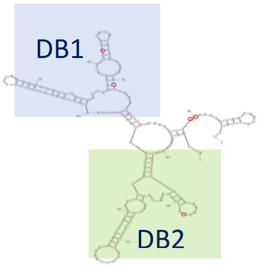	-62.00 kcal/mol	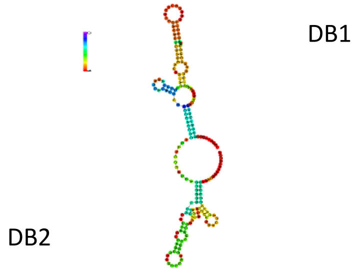	-60.98 kcal/mol	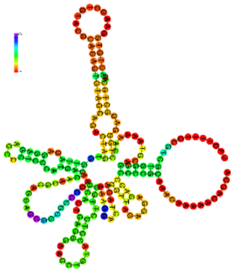
	JF504679	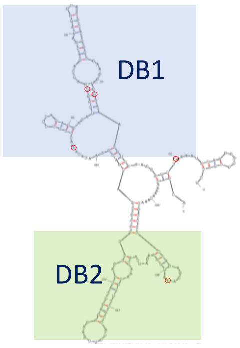	-59.00 kcal/mol	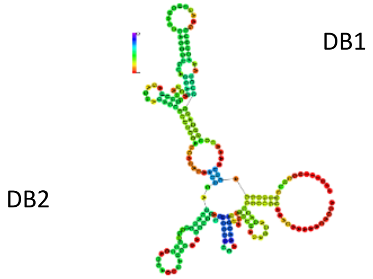	-58.71 kcal/mol	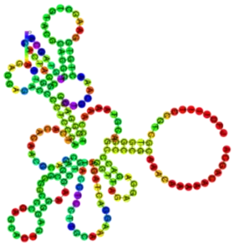

DENV3		Mfold predicted secondary structures	RNAfold predicted secondary structures			
		MFE structure	MFE structure		Centroid structure	
DENV3 Genotype III	KF954945	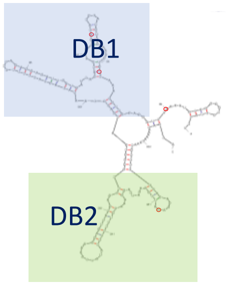	-62.00 kcal/mol	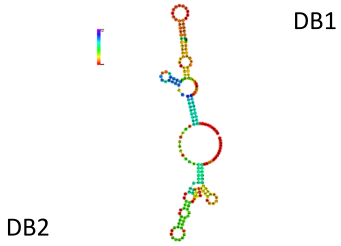	-60.91 kcal/mol	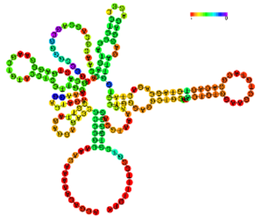
	FJ182013<br>GQ868571<br>AY099336	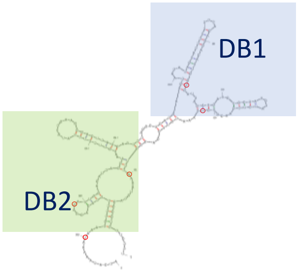	-61.40 kcal/mol	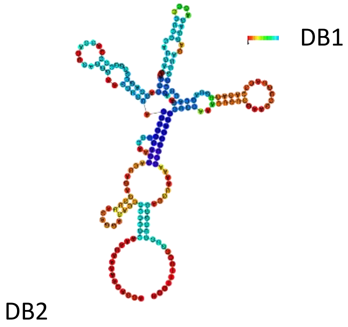	-60.09 kcal/mol	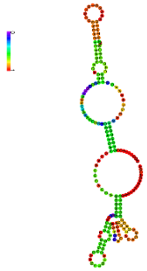
	HQ705618<br>KJ643590<br>KT726350	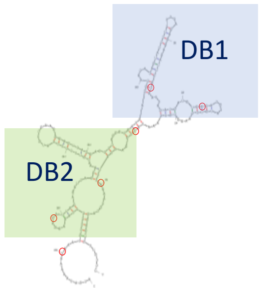	-62.10 kcal/mol	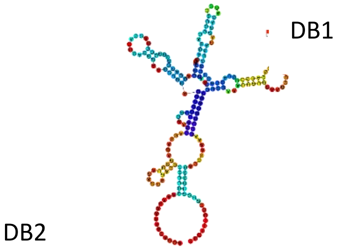	-60.72 kcal/mol	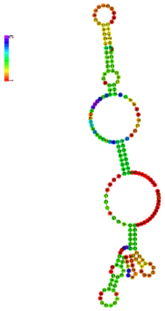

DENV3		Mfold predicted secondary structures	RNAfold predicted secondary structures			
		**MFE structure**	**MFE structure**		**Centroid structure**	
**DENV3 Genotype V**	KU509282<br>KU509695<br>JN697379	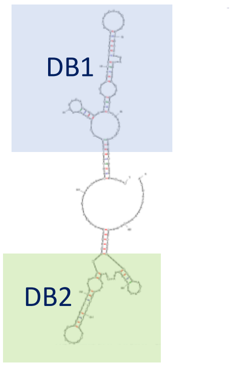	-63.30 kcal/mol	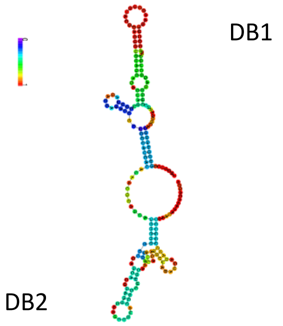	-62.711 kcal/mol	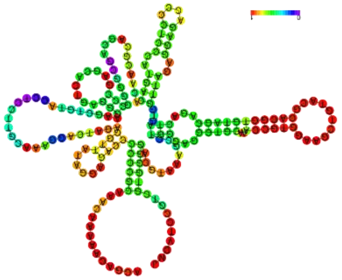

SNVs for each DENV3 isolate structure is highlighted in red on each Mfold Predicted structure. Base pairing probability in MEF and Centroid structures of RNA predicted secondary structures, is denoted by the colored nucleotides. Colours are rated from 1-0 to indicate strong to weak base pairing probabilities (Red - strongest probability, Green - medium probability, Blue - lowest probability) in the colour scale.
