# Supplementary material for: Genetic Variation in the Domain II, 3′ Untranslated Region of Human and Mosquito Derived Dengue Virus Strains in Sri Lanka
Source: Viruses. 2021 Mar 5;13(3):421. doi: 10.3390/v13030421 (PMC8001906; doi:10.3390/v13030421)
Supplement: Supplementary file 1 [file viruses-13-00421-s001.zip › Supplimentry files/Supplimentry tables/Table S7.docx]

**Table S7. Mfold and RNAfold predicted secondary structures for RNA alignments of DENV1, Domain II region of 3’UTR sequences identified in the study, Sri Lankan isolates and DENV1 reference genotypes.**

| **DENV1** | | **Mfold predicted secondary structures** | | **RNAfold predicted secondary structures** | | |
| --- | --- | --- | --- | --- | --- | --- |
|  |  | **MFE structure** | | **MFE structure** | | **Centroid structure** |
| **DENV1 reference** | EU848545 | 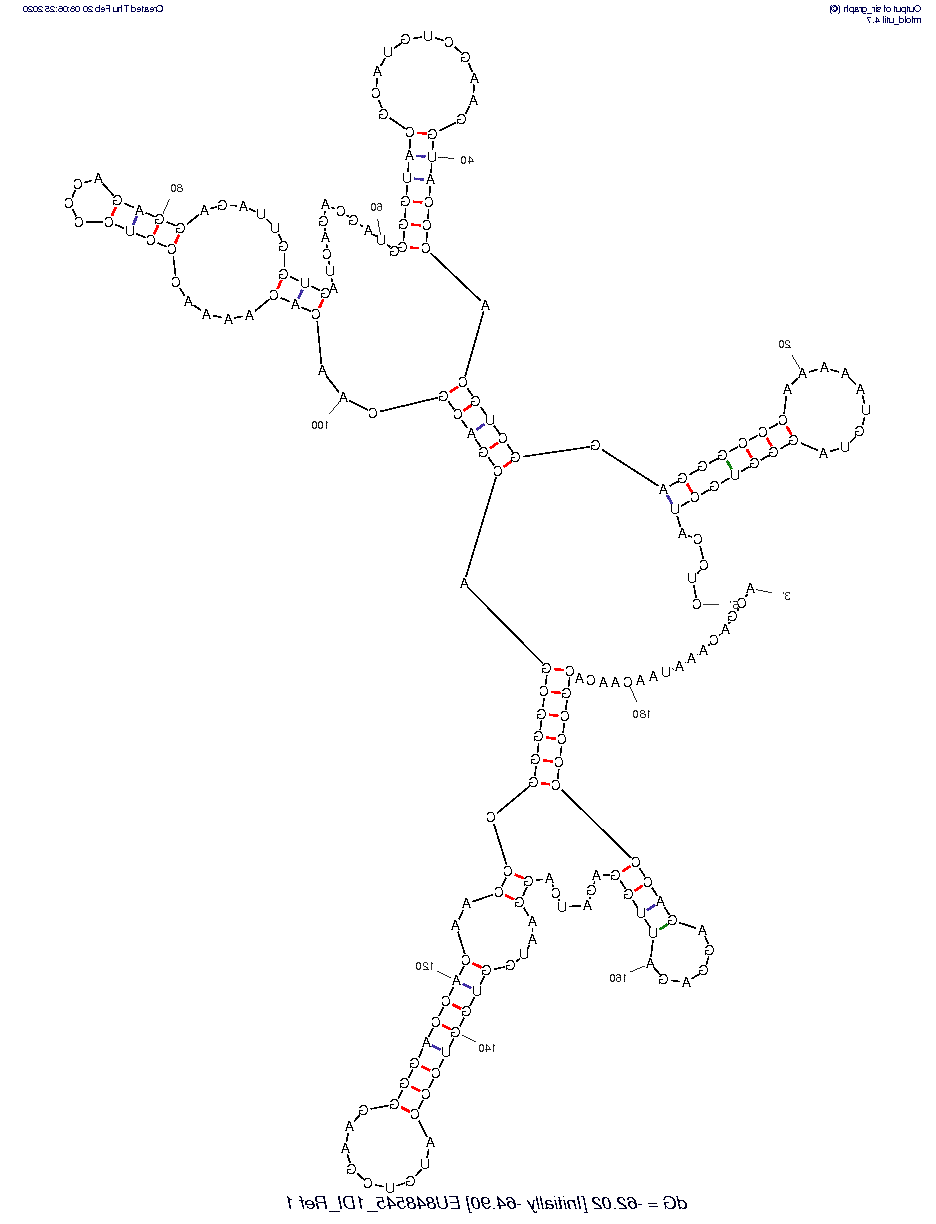 DB1  DB2 | -65.10 kcal/mol | DB1  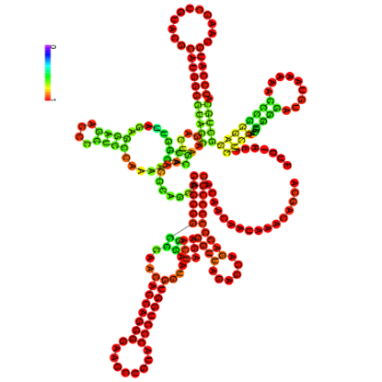  DB2 | -64.10 kcal/mol | 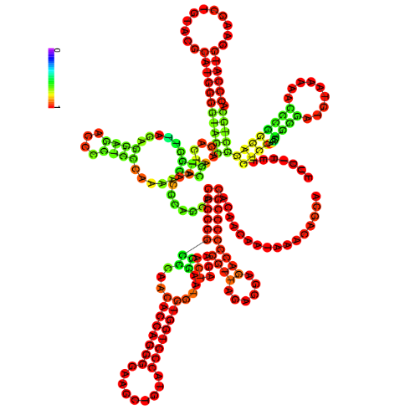 |
| **Study** | D1H_2019SL | 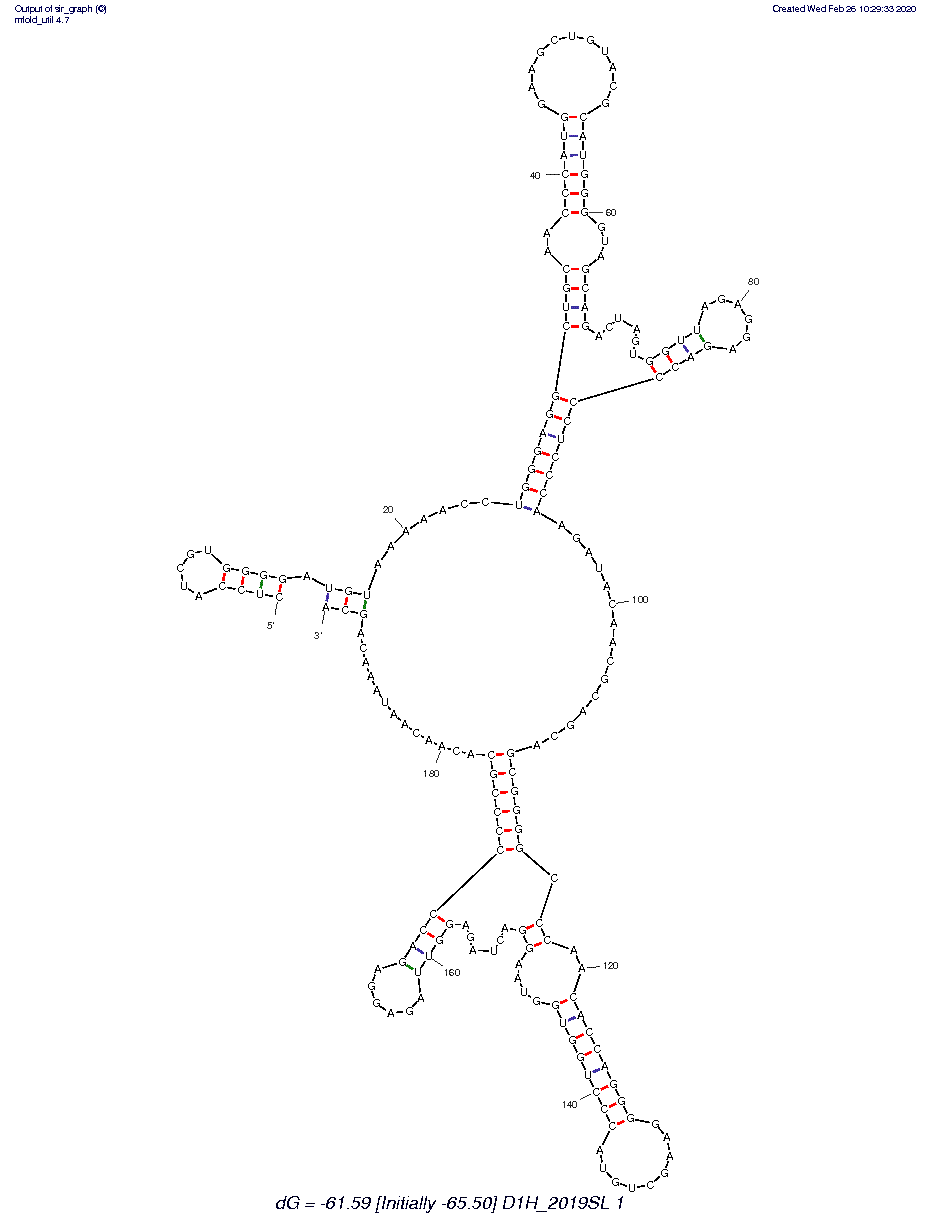  DB1  DB2 | -65.50 kcal/mol | 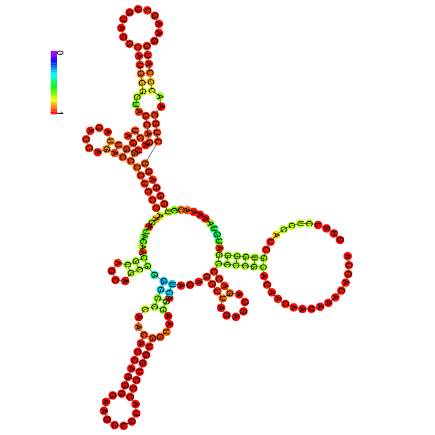  DB1  DB2 | -64.40 kcal/mol | 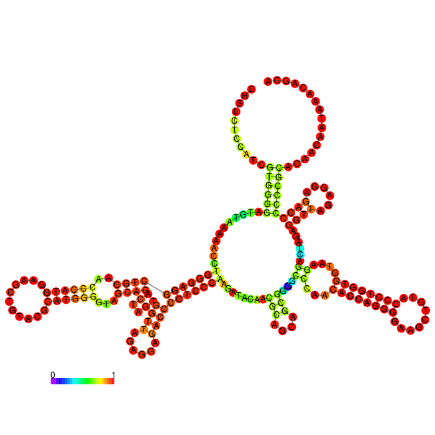 |
| **DENV1** | | **Mfold predicted secondary structures** | | **RNAfold predicted secondary structures** | | |
|  |  | **MFE structure** 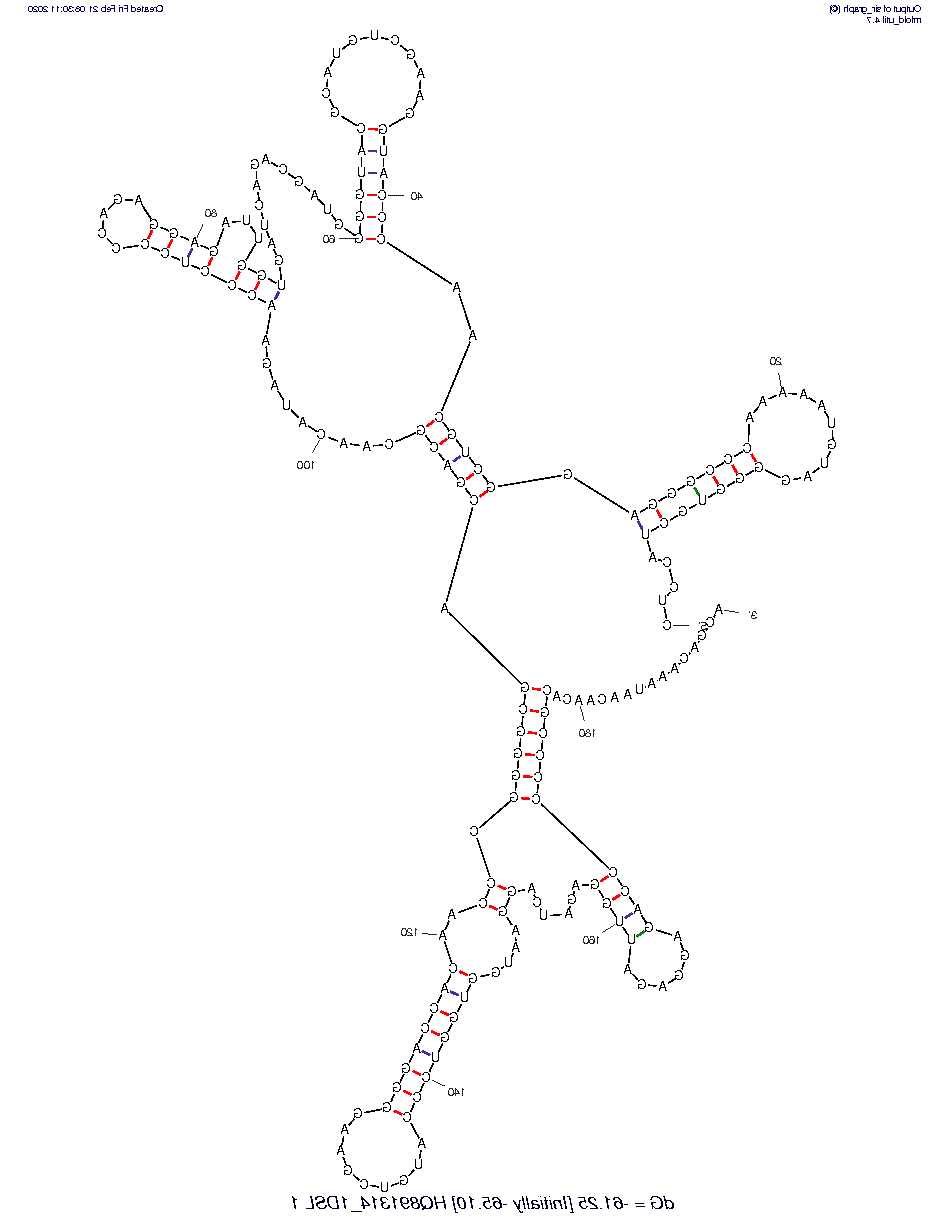 DB1  DB2 | | **MFE structure** | | **Centroid structure** |

| **Sri Lankan Isolates of DENV1** | HQ891314 |  | -65.10 kcal/mol | 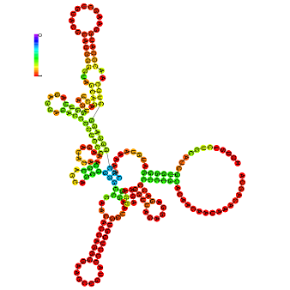  DB2  DB1 | -66.62 kcal/mol | 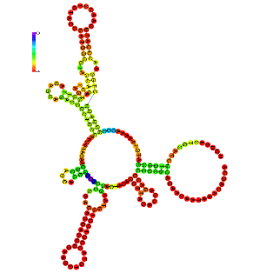 |
| --- | --- | --- | --- | --- | --- | --- |
|  | KJ726664 | 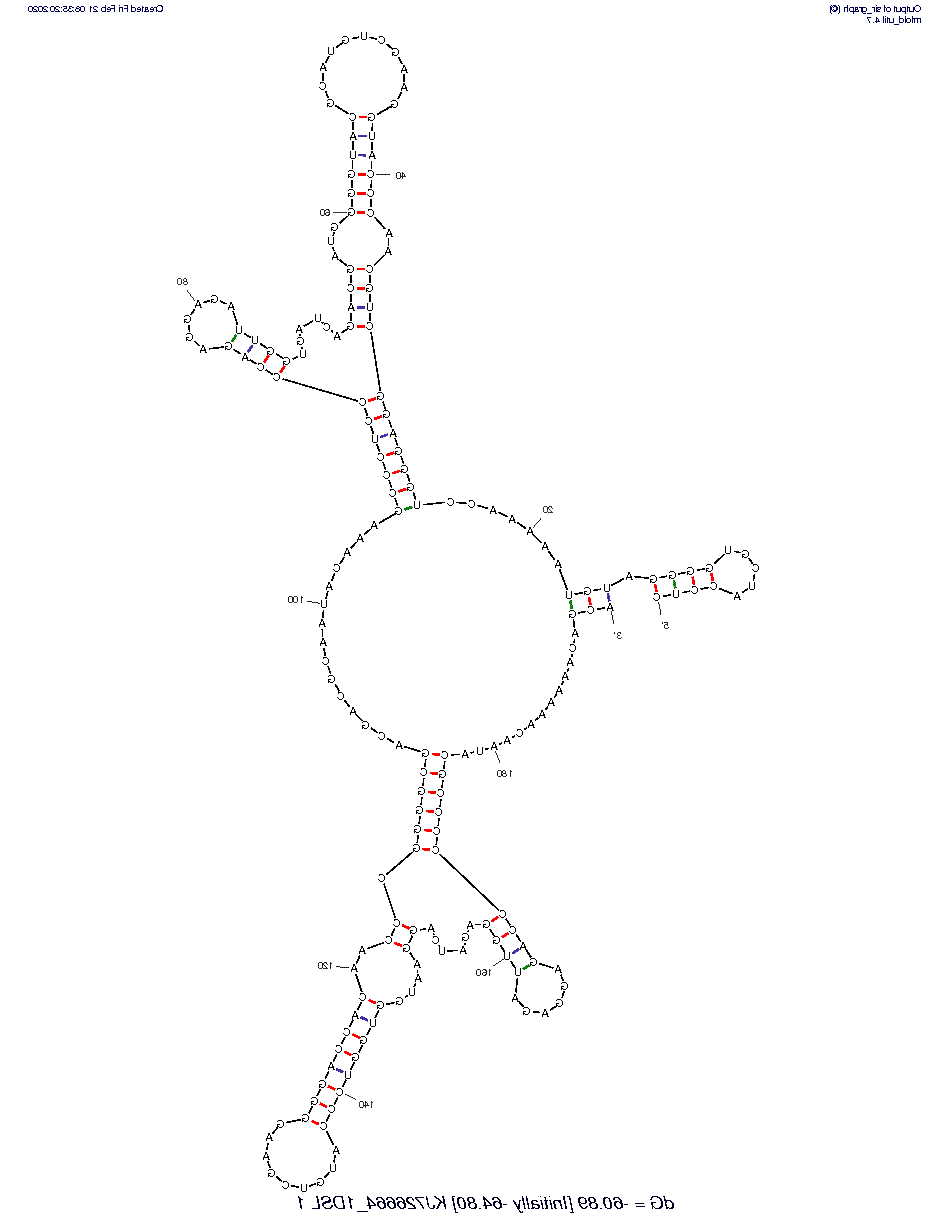 DB1  DB2 | -64.80 kcal/mol | 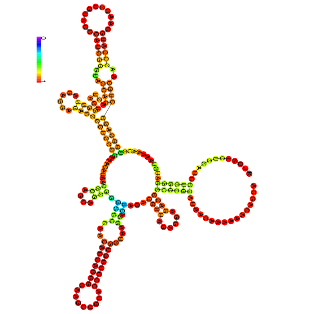  DB2  DB1 | -66.62 kcal/mol | 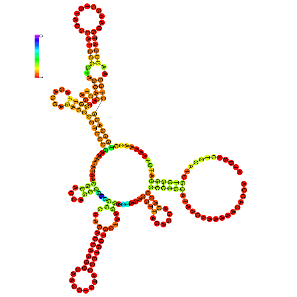 |
|  | KJ726665  KJ726663  HQ891315  JN054256  KJ468234  KJ726662  HQ891316  JN054255 | 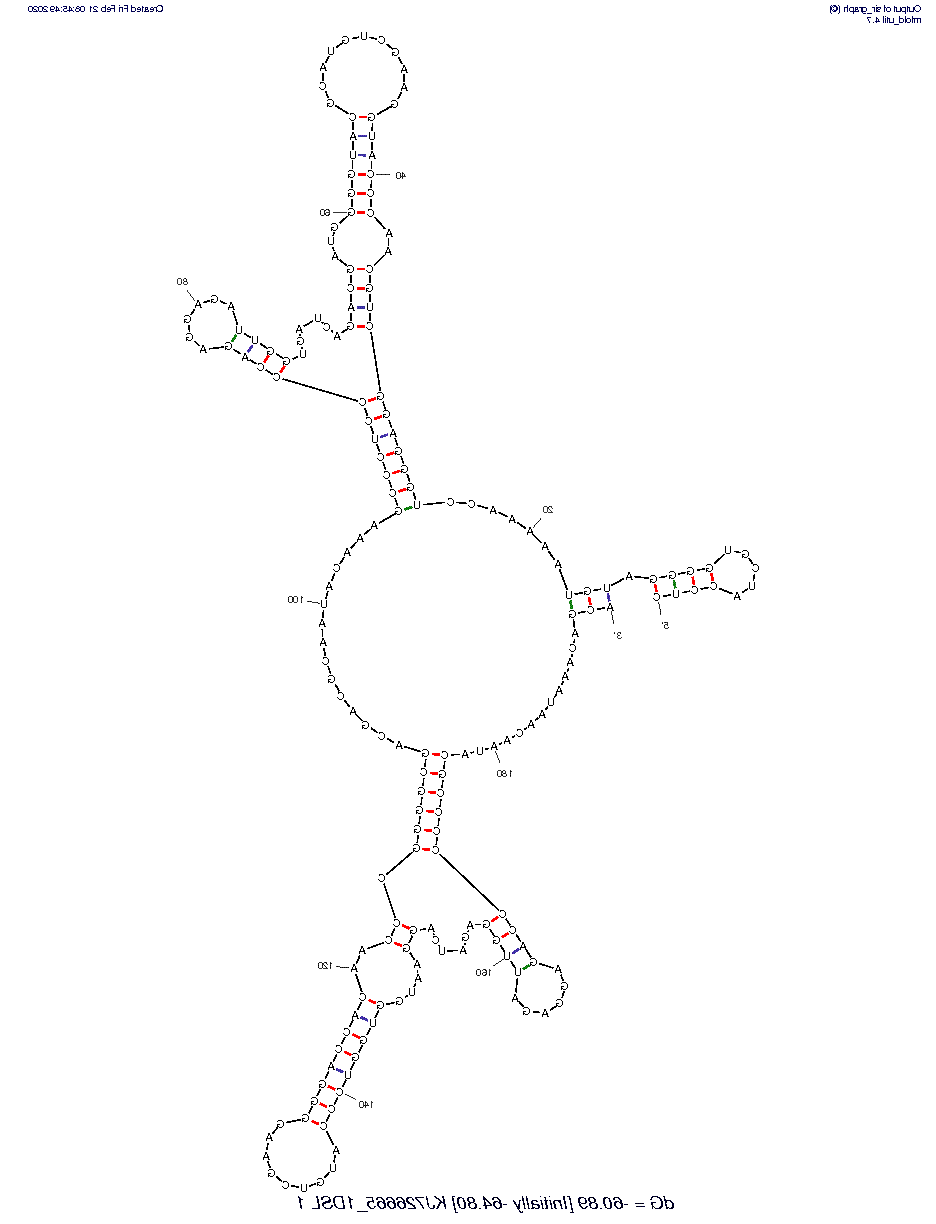 DB1  DB2 | -64.80 kcal/mol | 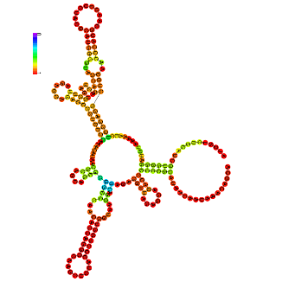  DB2  DB1 | -66.63 kcal/mol | 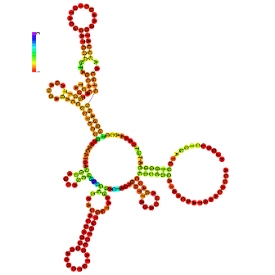 |
|  | KP398852 | 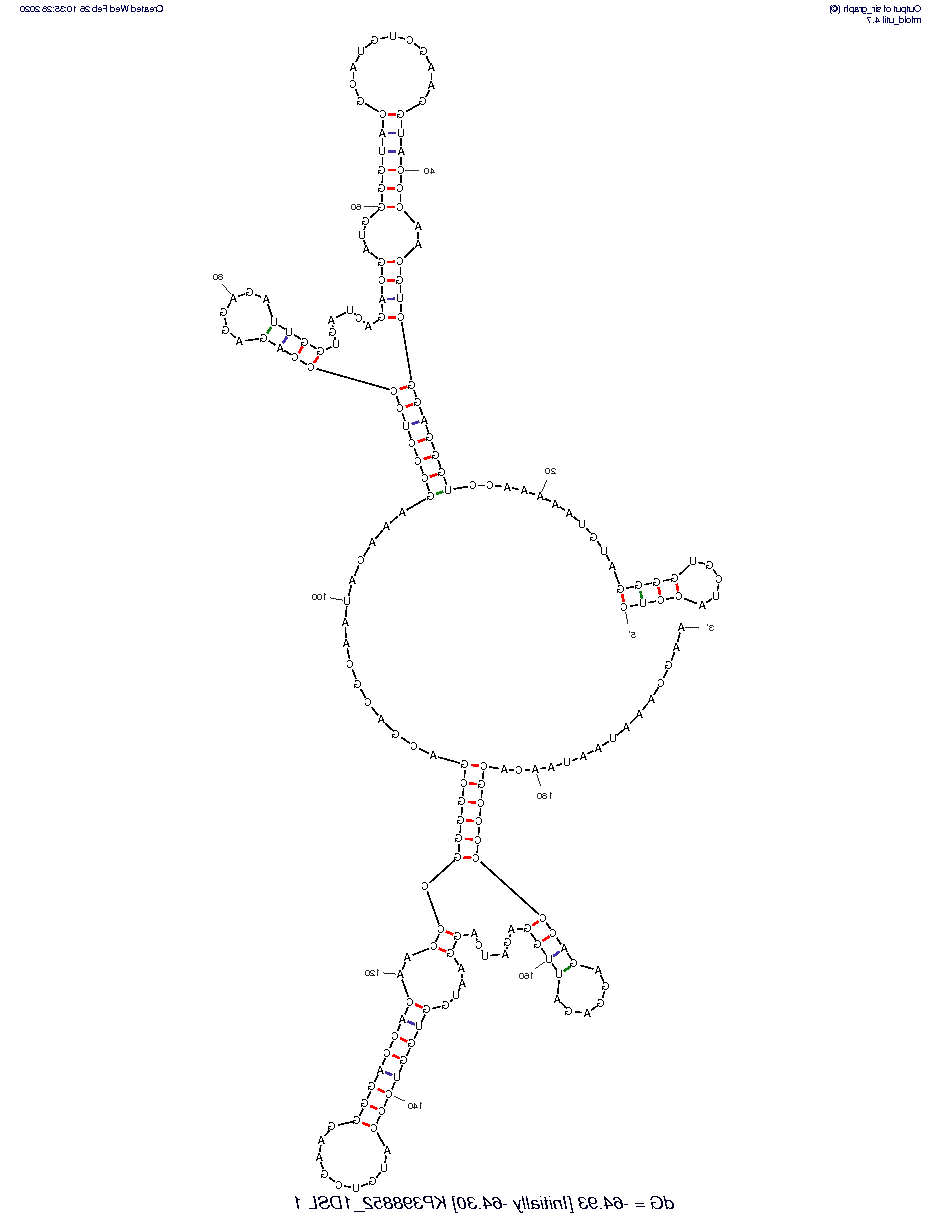 DB1  DB2 | -64.30 kcal/mol | 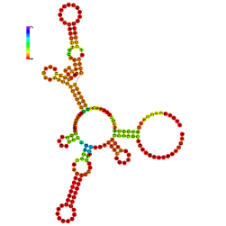  DB2  DB1 | -66.61 kcal/mol | 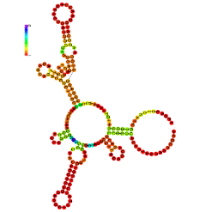 |

| **DENV1** | **Mfold predicted secondary structures** | **RNAfold predicted secondary structures** | |
| --- | --- | --- | --- |
|  | **MFE structure** | **MFE structure** | **Centroid structure** |

| DENV1 Genotype I | AF309641 | 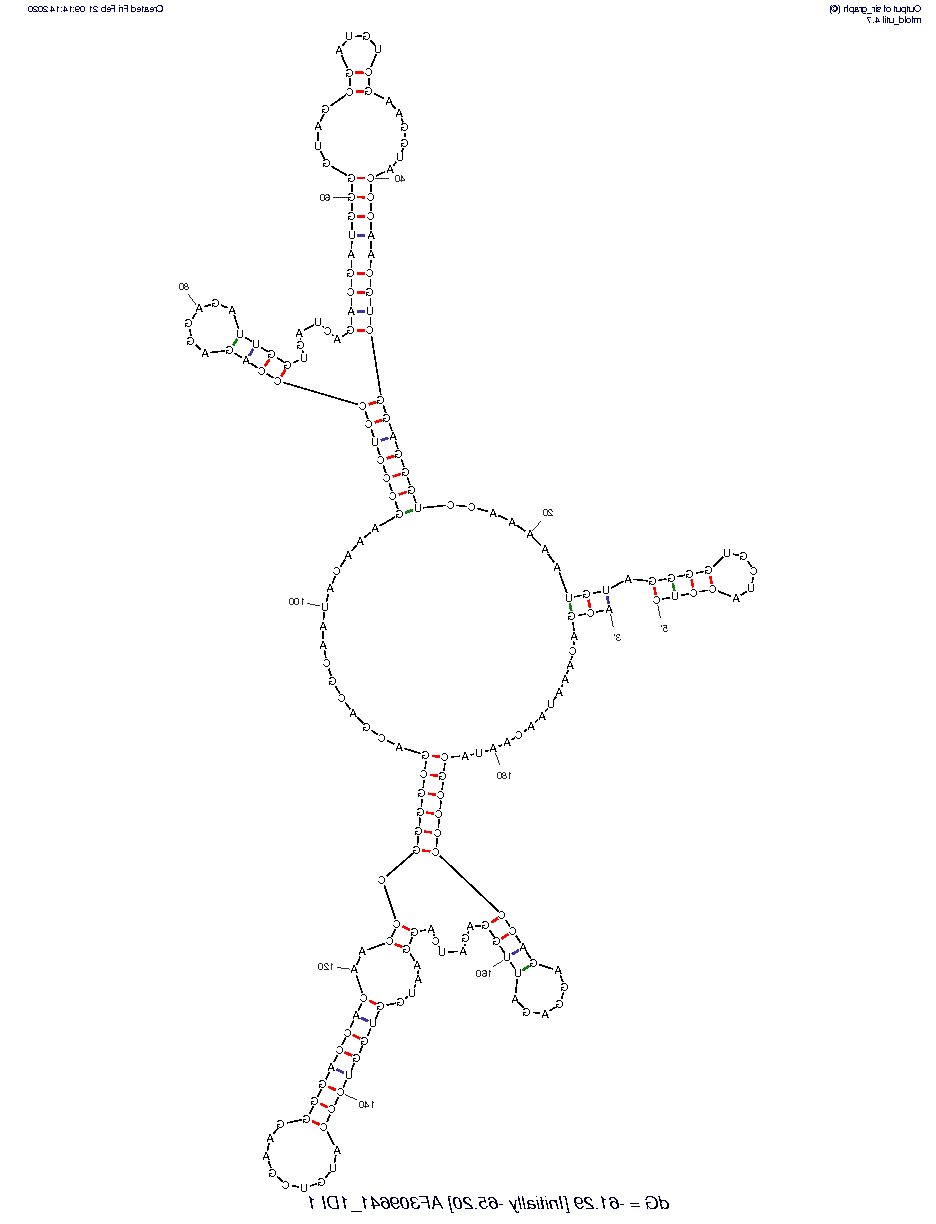 DB1  DB2 | -61.29  kcal/mol | 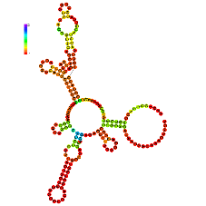  DB2  DB1 | -65.12  kcal/mol | 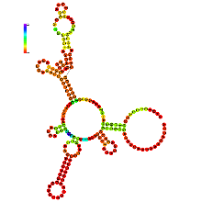 |
| --- | --- | --- | --- | --- | --- | --- |
|  | AB074760 | 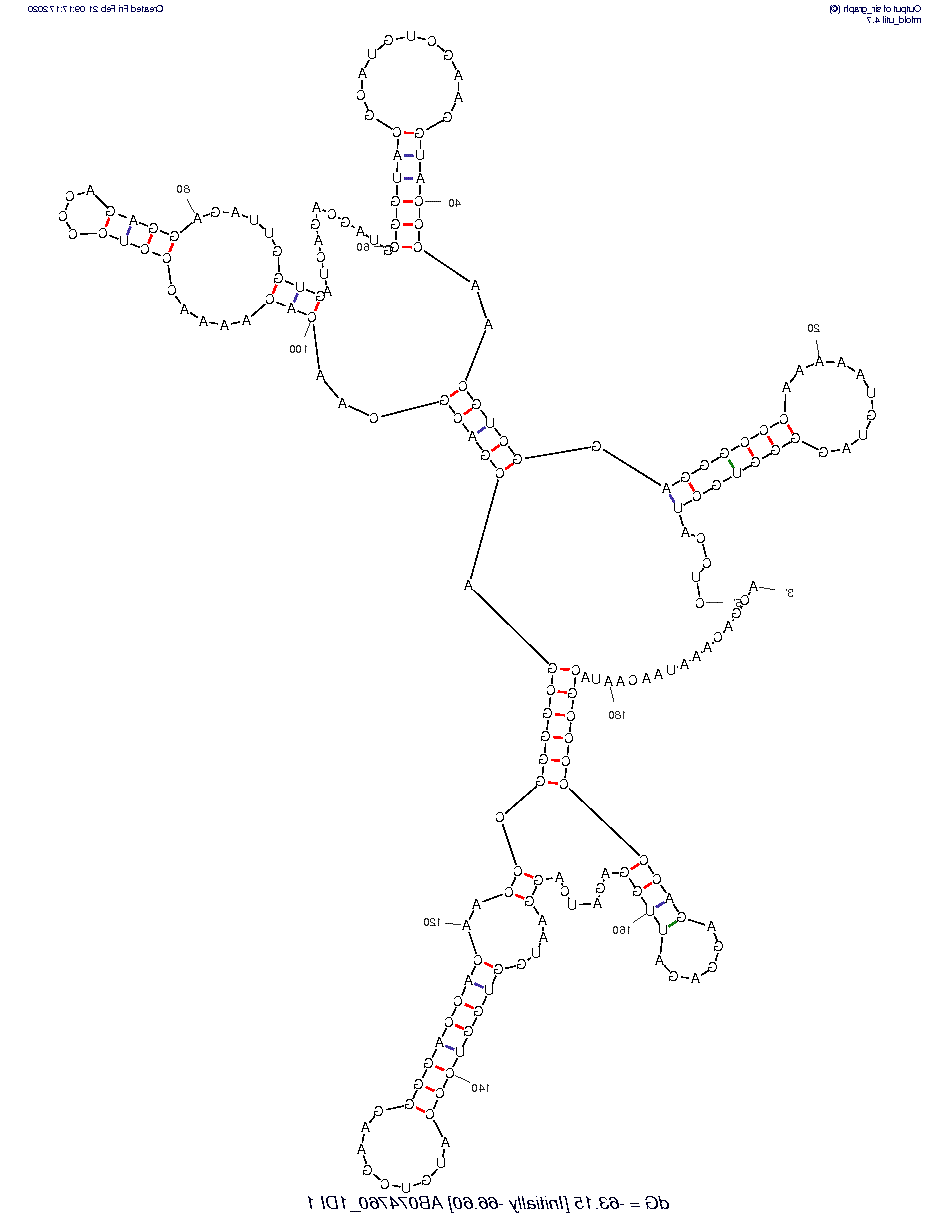 DB1  DB2 | -66.60 kcal/mol | 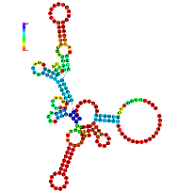  DB1  DB2 | -67.06  kcal/mol | 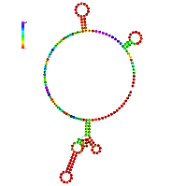 |
|  | AF350498  AY732477  JN638342 | 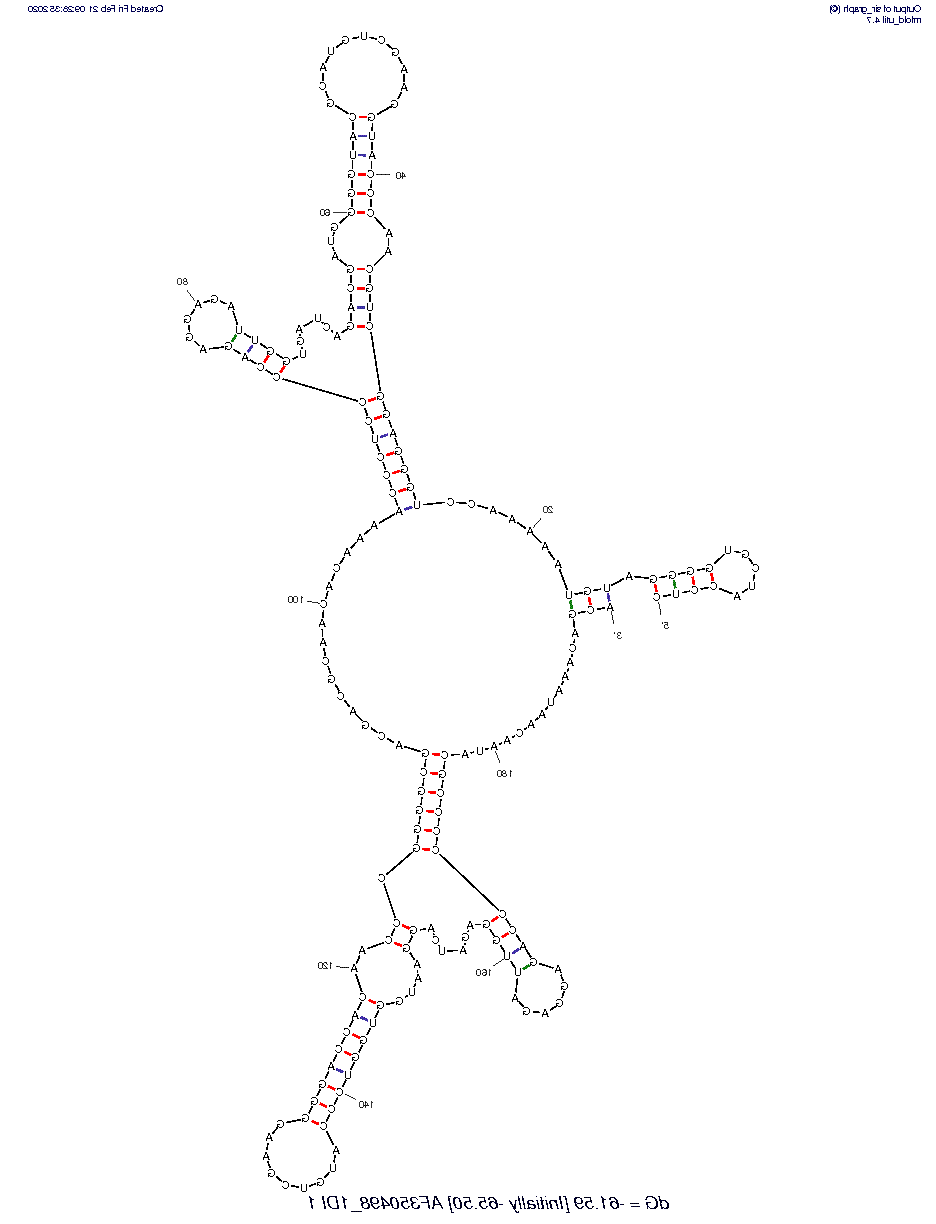 DB1  DB2 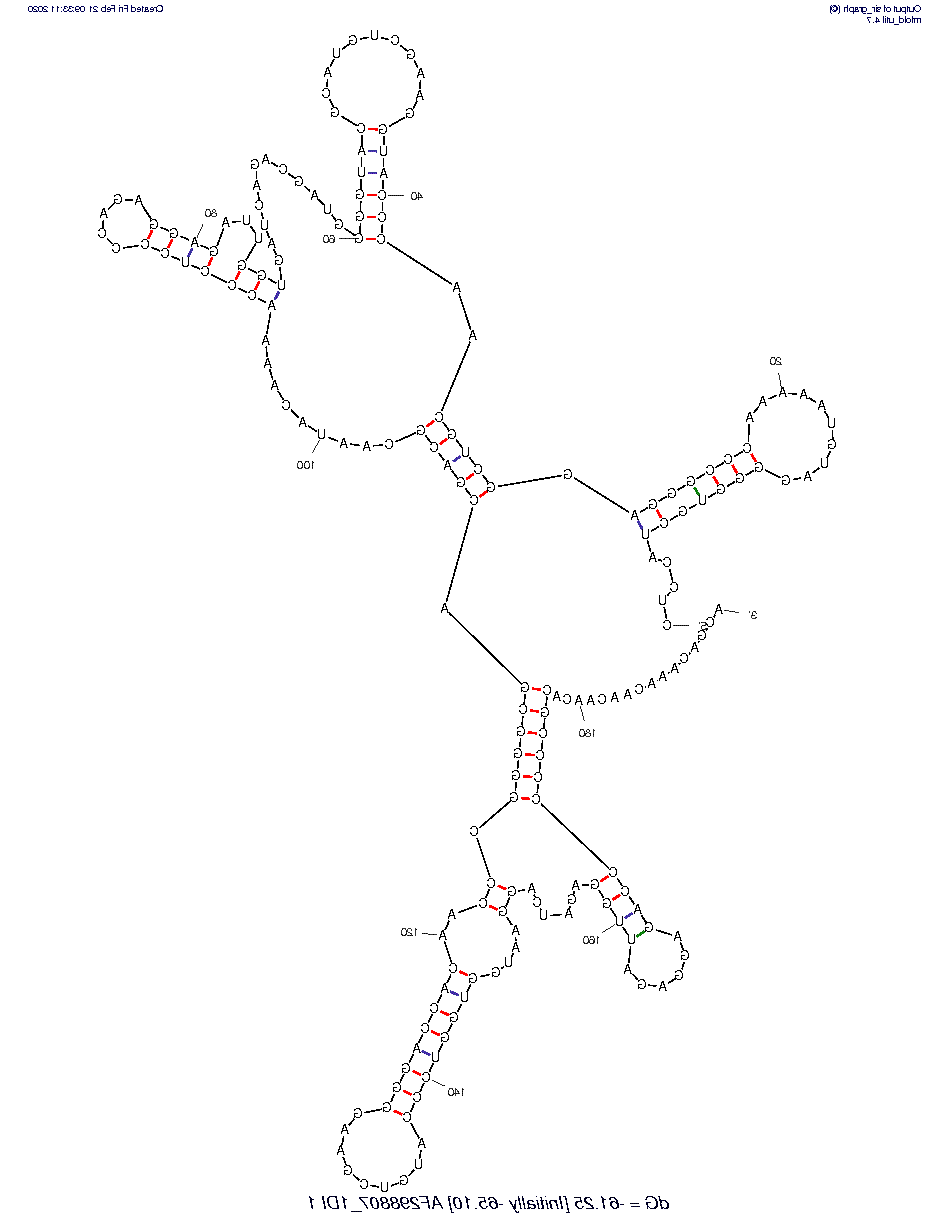 DB1  DB2 | -61.59  kcal/mol | 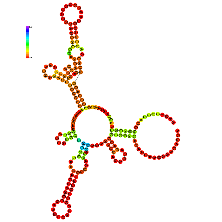  DB2  DB1 | -67.11  kcal/mol | 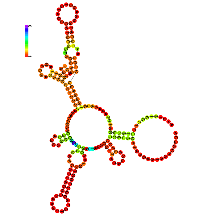 |
|  | AF298807 |  | -65.10 kcal/mol | 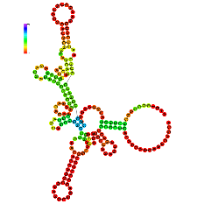  DB2  DB1 | -66.63 kcal/mol | 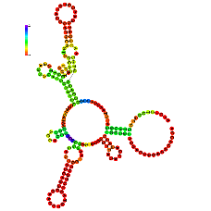 |
|  | AY726555  EU081226 | 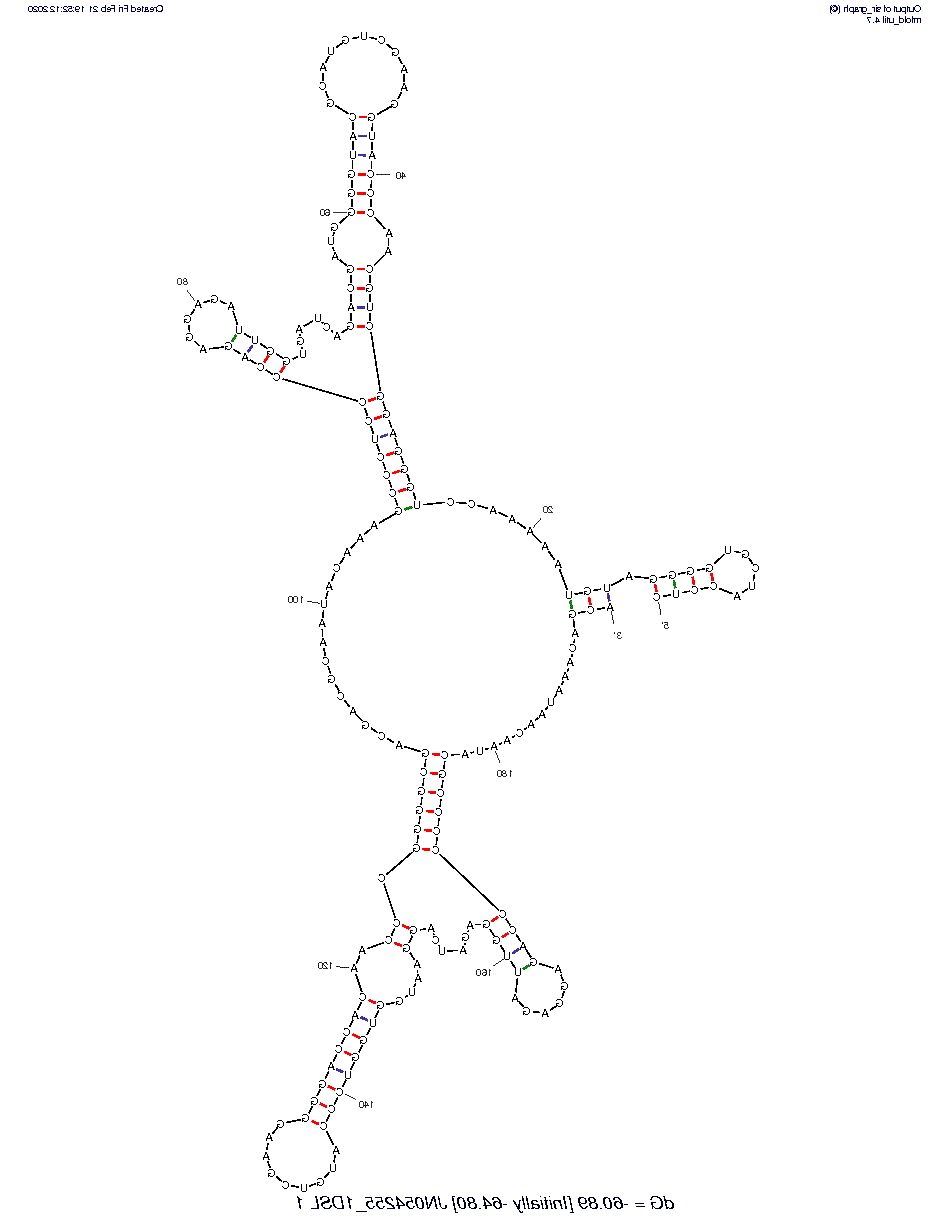 DB1  DB2 | -61.59  kcal/mol | 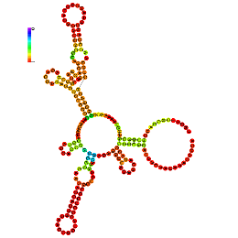  DB2  DB1 | -67.11  kcal/mol | 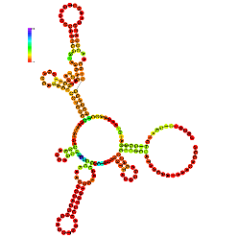 |
| **DENV1** | | **Mfold predicted secondary structures** | | **RNAfold predicted secondary structures** | | |
|  |  | **MFE structure** | | **MFE structure** | | **Centroid structure** |
| DENV1 Genotype I | AY732479 | 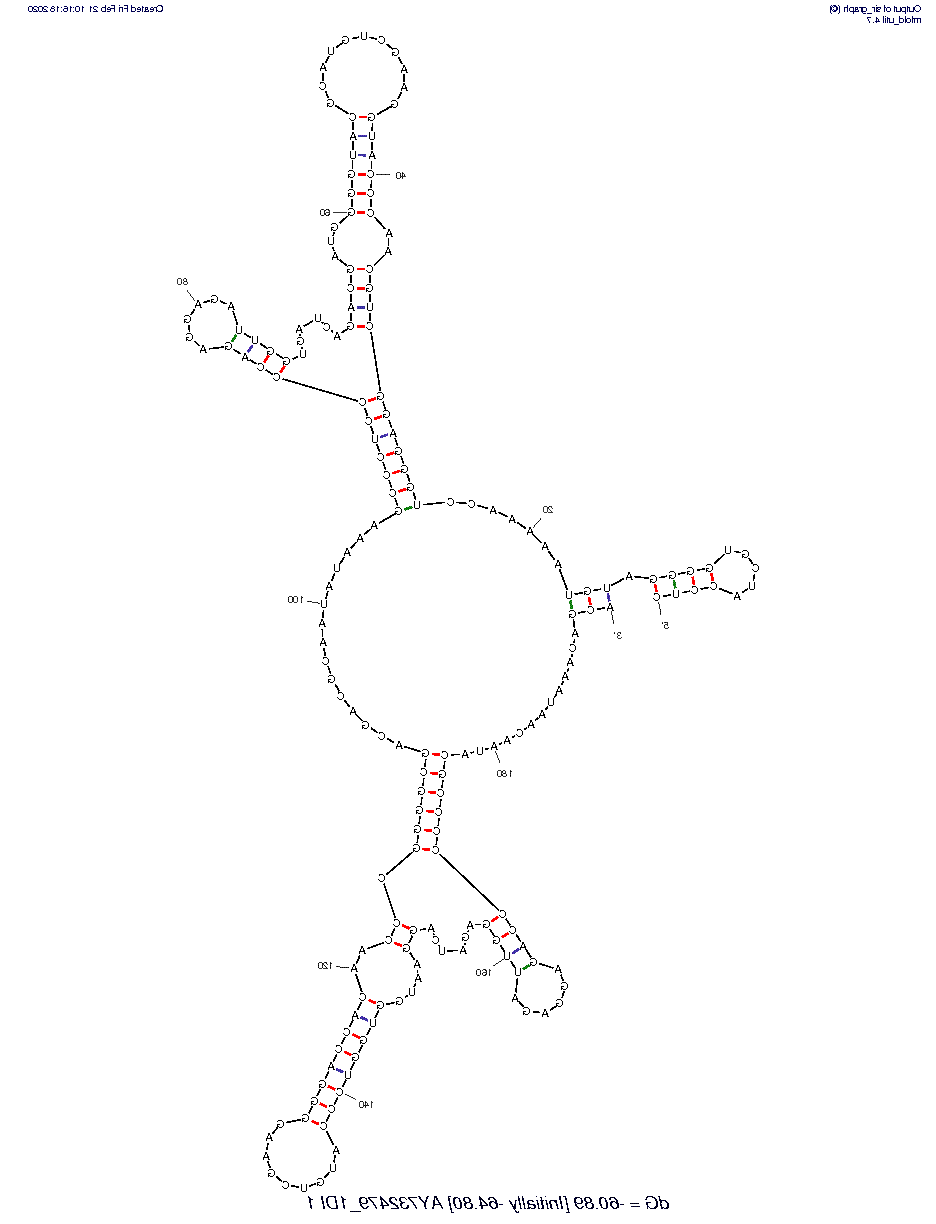 DB1  DB2 | -64.80  kcal/mol | 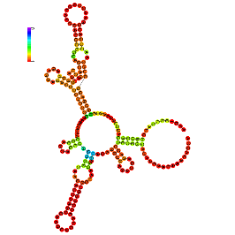  DB2  DB1 | -60.49  kcal/mol | 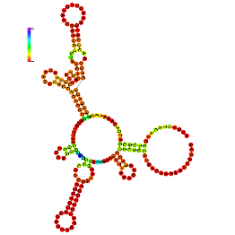 |
|  | AY732480 | 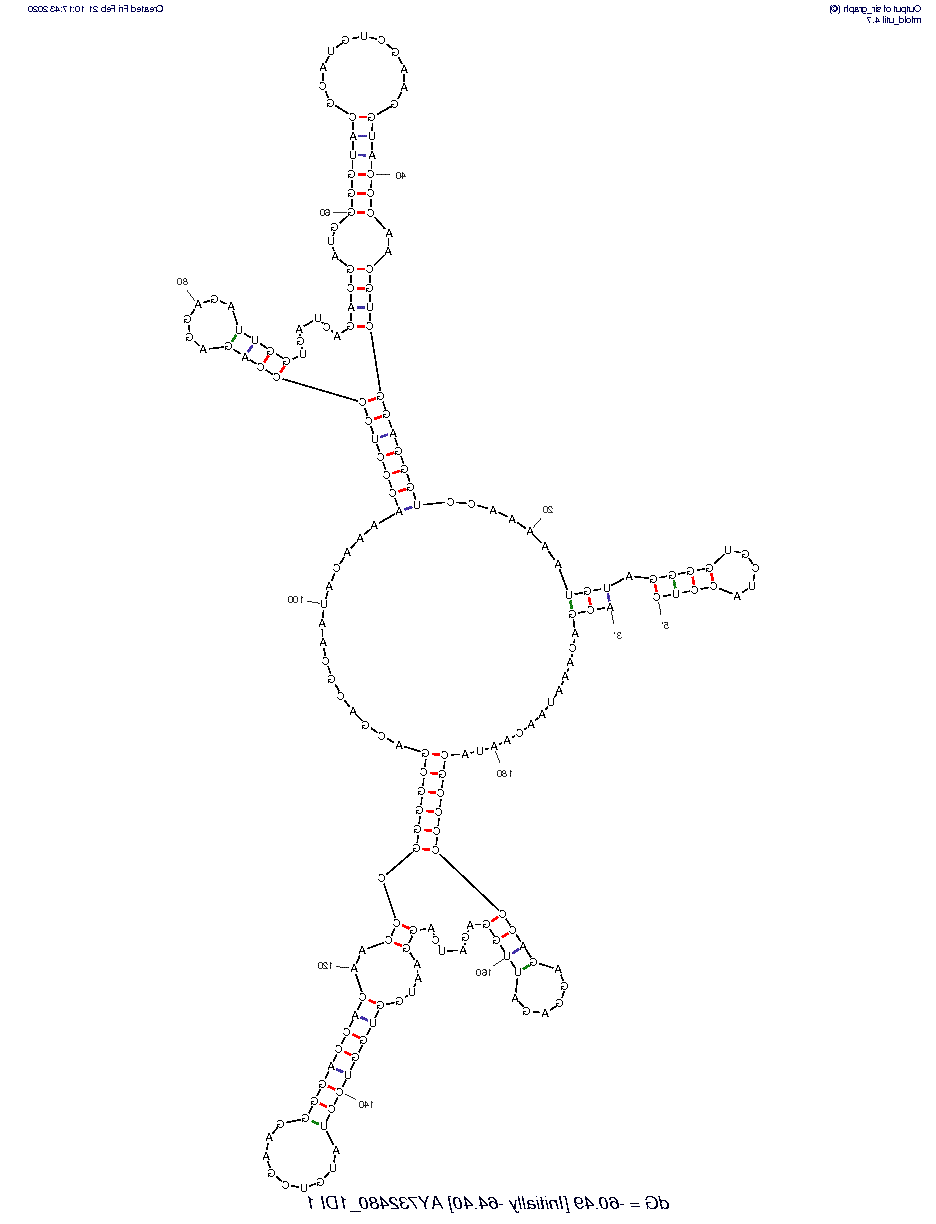 DB1  DB2 | -60.49  kcal/mol | 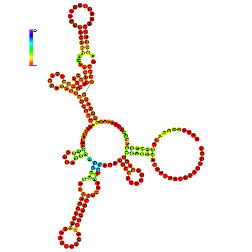  DB2  DB1 | -63.92  kcal/mol | 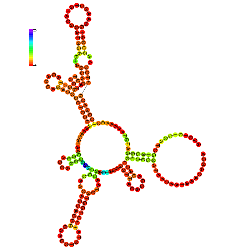 |
|  | AY732483 | 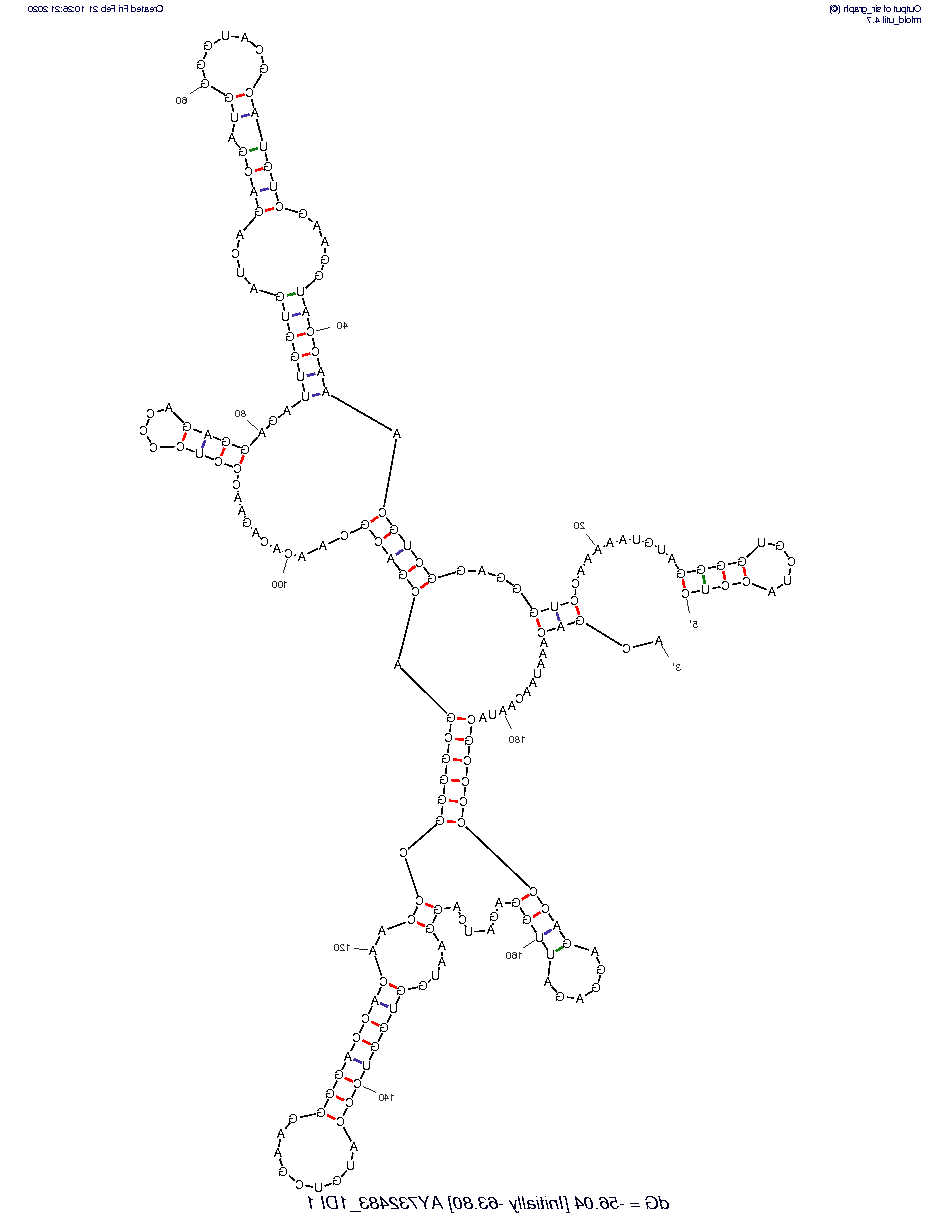 DB1  DB2 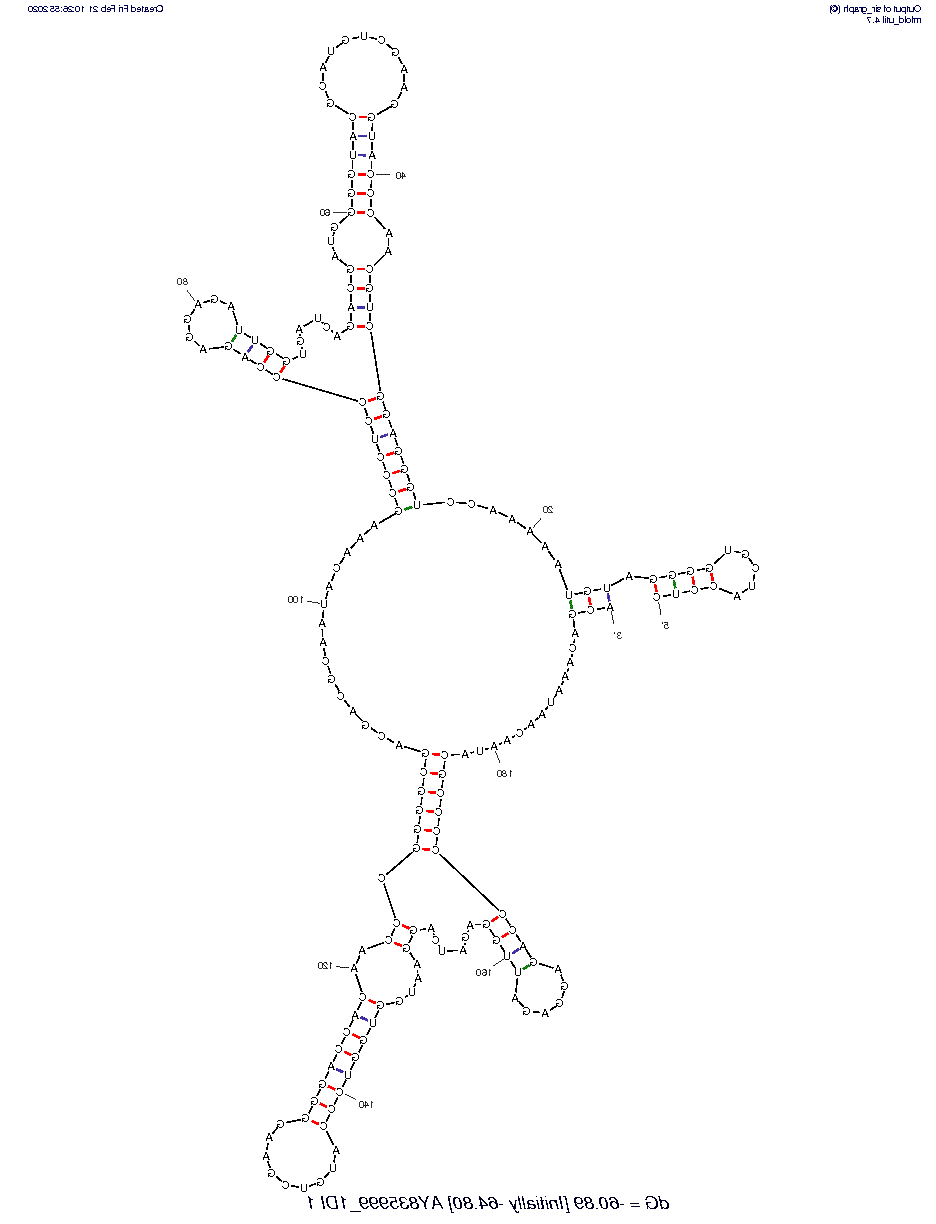 DB1  DB2 | -63.80  kcal/mol | 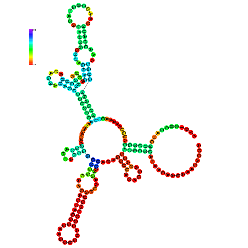  DB2  DB1 | -64.15 kcal/mol | 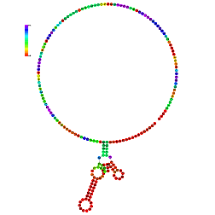 |
|  | AY835999  HQ891316  KJ726622 |  | -64.80  kcal/mol | 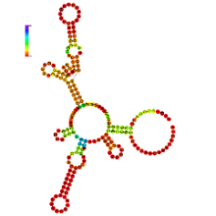  DB2  DB1 | -66.63  kcal/mol | 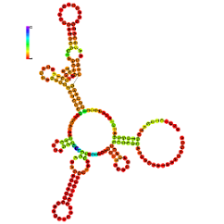 |
|  | DQ285561 | 3` 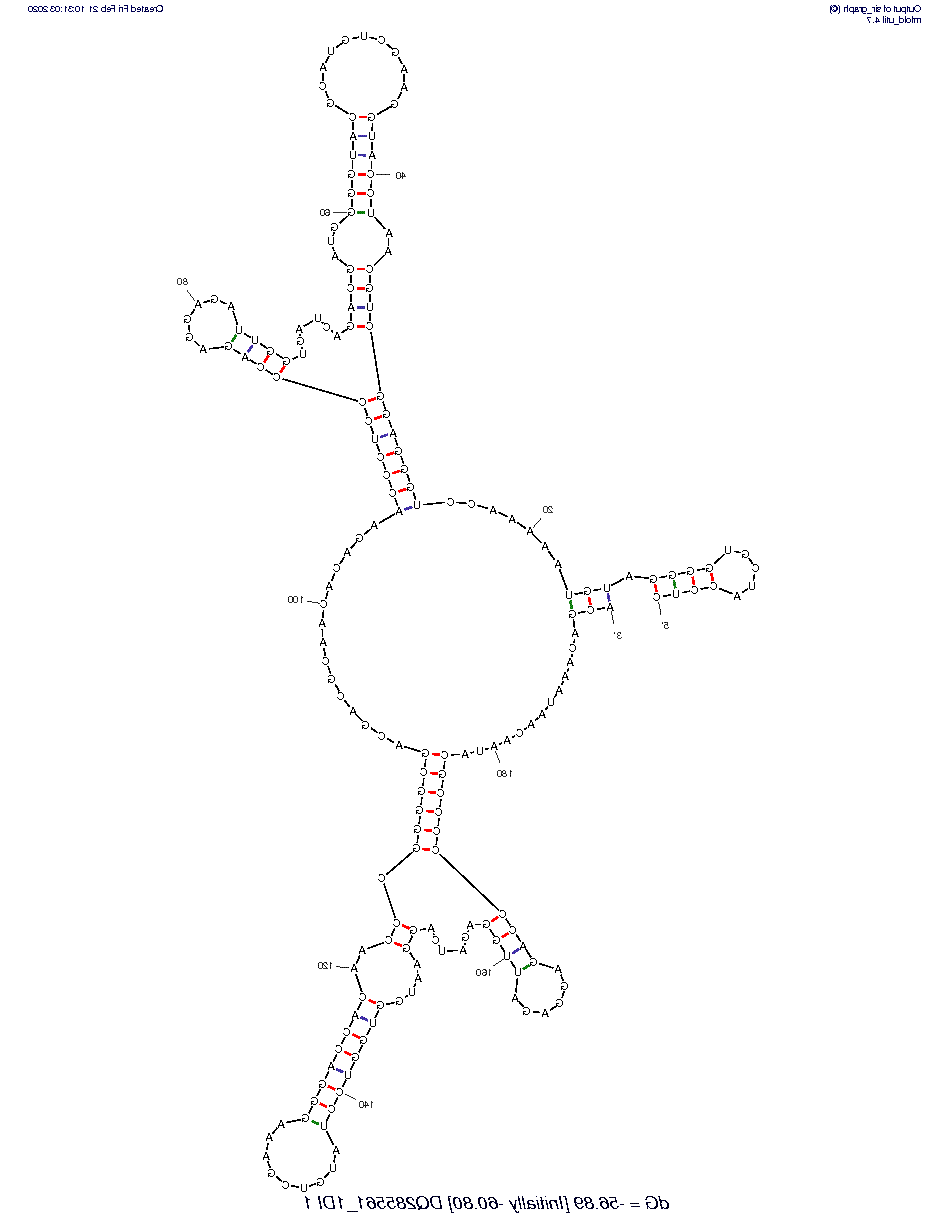 DB1  DB2 | -60.80  kcal/mol | 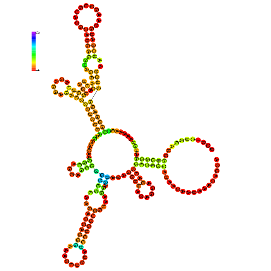  DB2  DB1 | -60.88  kcal/mol | 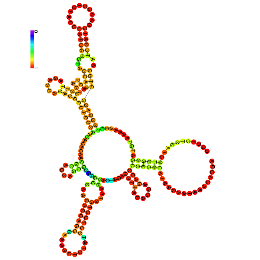 |
| **DENV1** | | **Mfold predicted secondary structures** | | **RNAfold predicted secondary structures** | | |
|  |  | **MFE structure** | | **MFE structure** | | **Centroid structure** |
| DENV1 Genotype I | 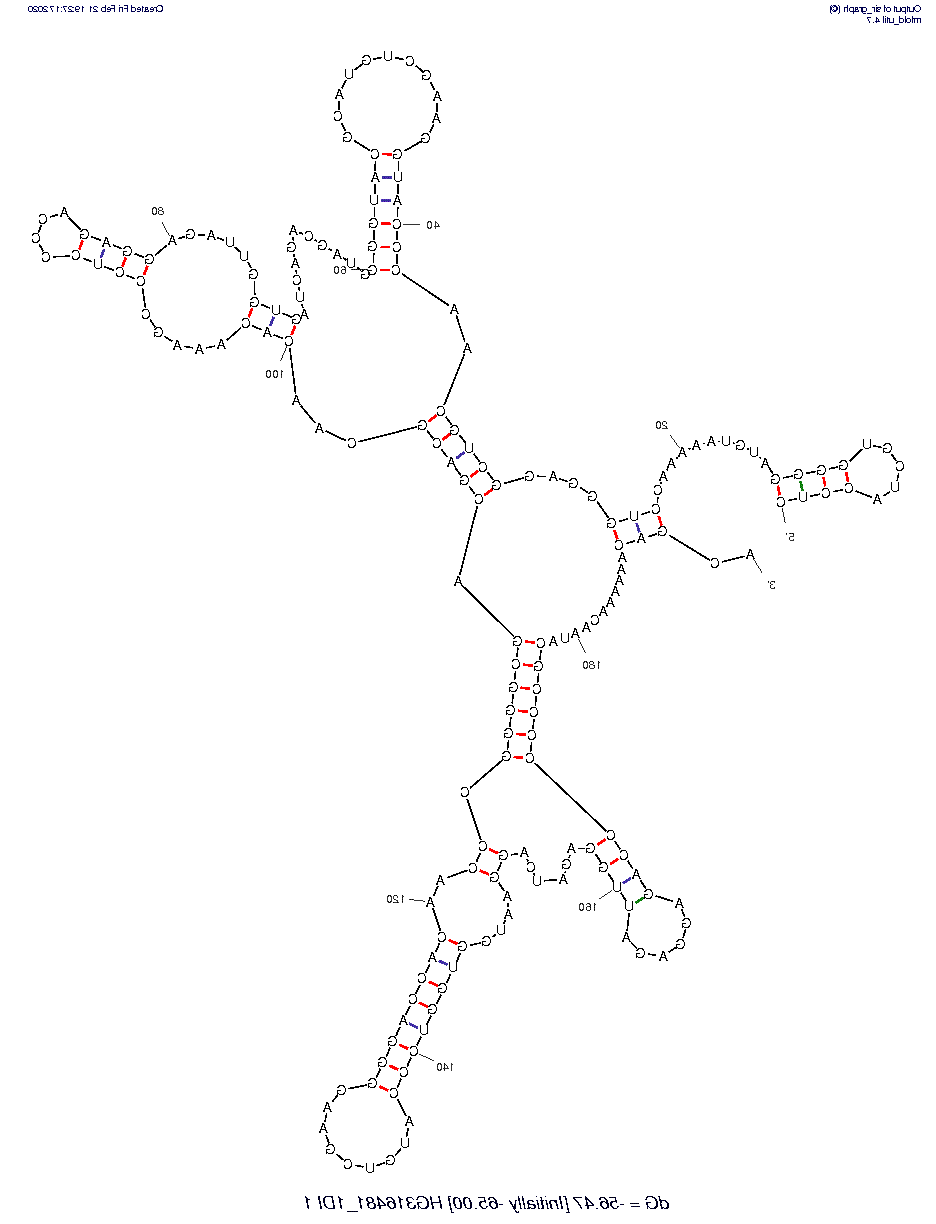 DB1  DB2  HG316481 |  | -65.00  kcal/mol | 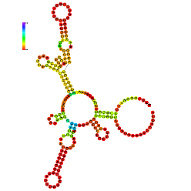  DB2  DB1 | -66.65 kcal/mol | 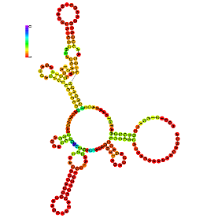 |
|  | HG316482 | 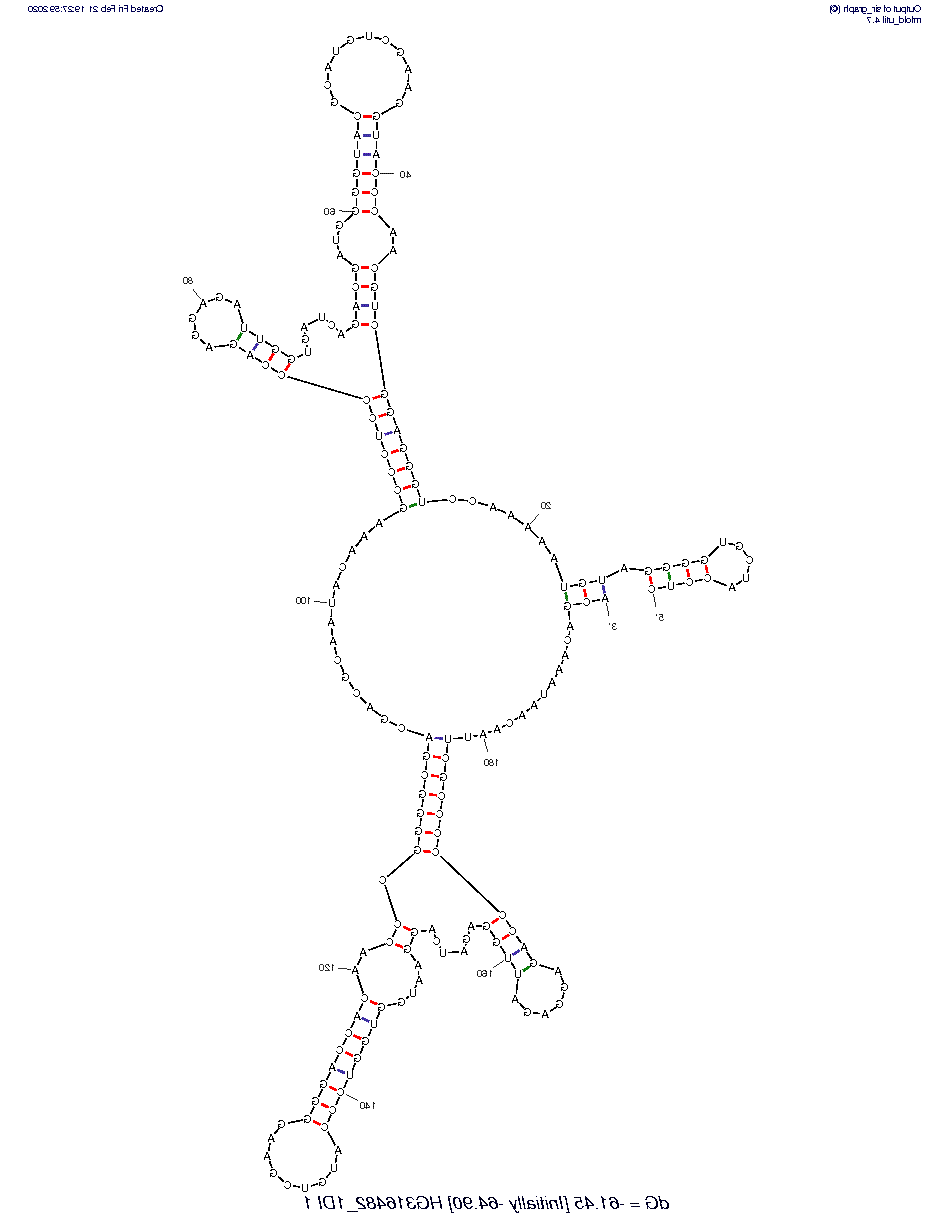 DB1  DB2 | -64.90  kcal/mol | 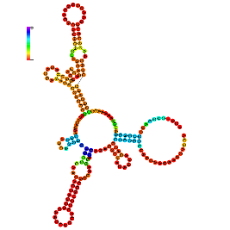  DB2  DB1 | -66.98  kcal/mol | 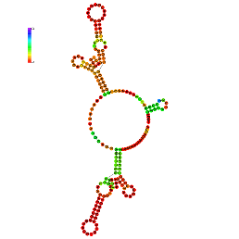 |
|  | JN638340 | 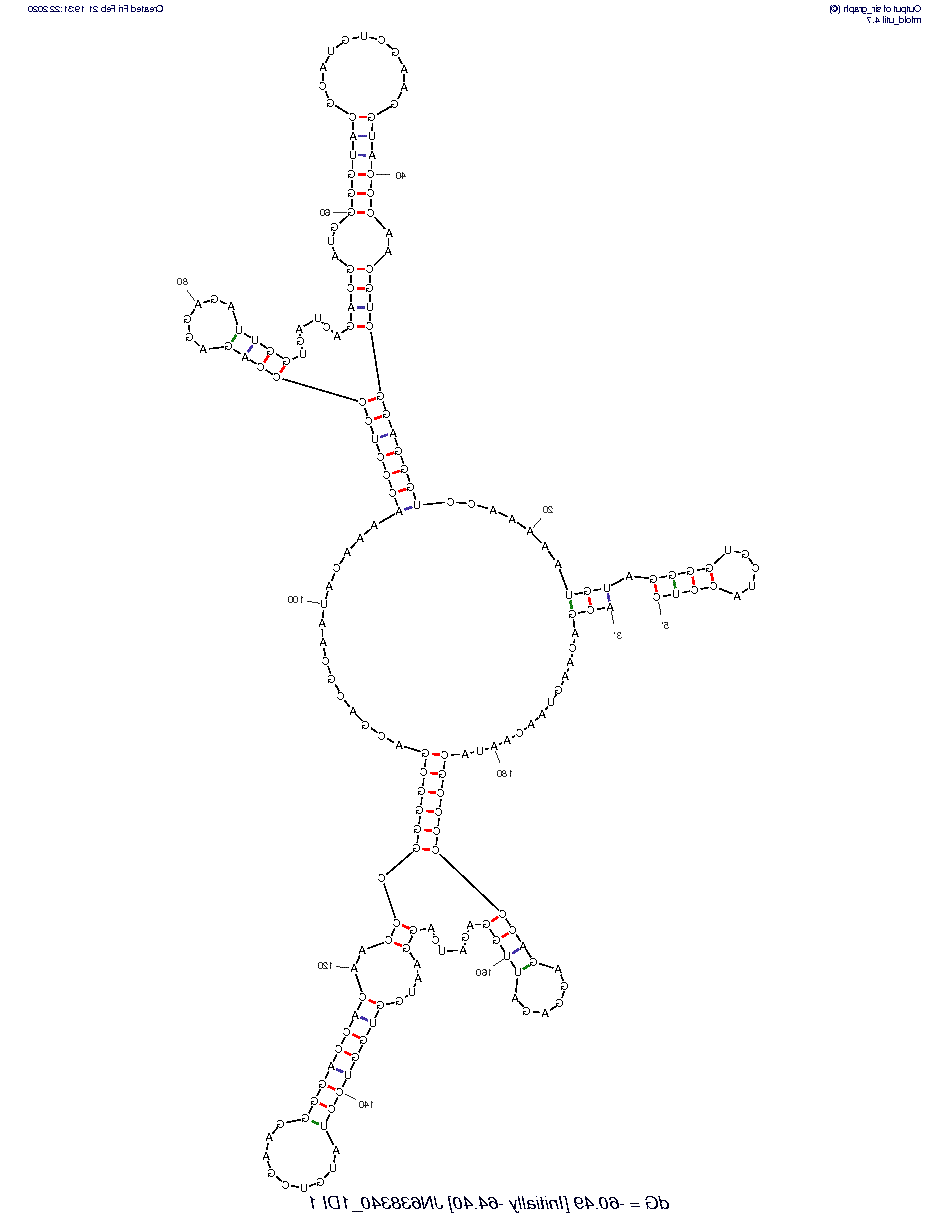 DB1  DB2 | -64.40  kcal/mol | 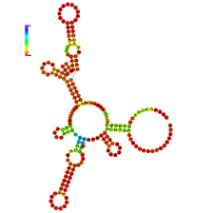  DB2  DB1 | -63.96  kcal/mol | 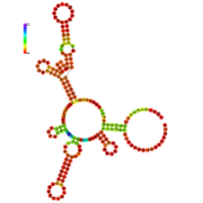 |
|  | JN638344 | 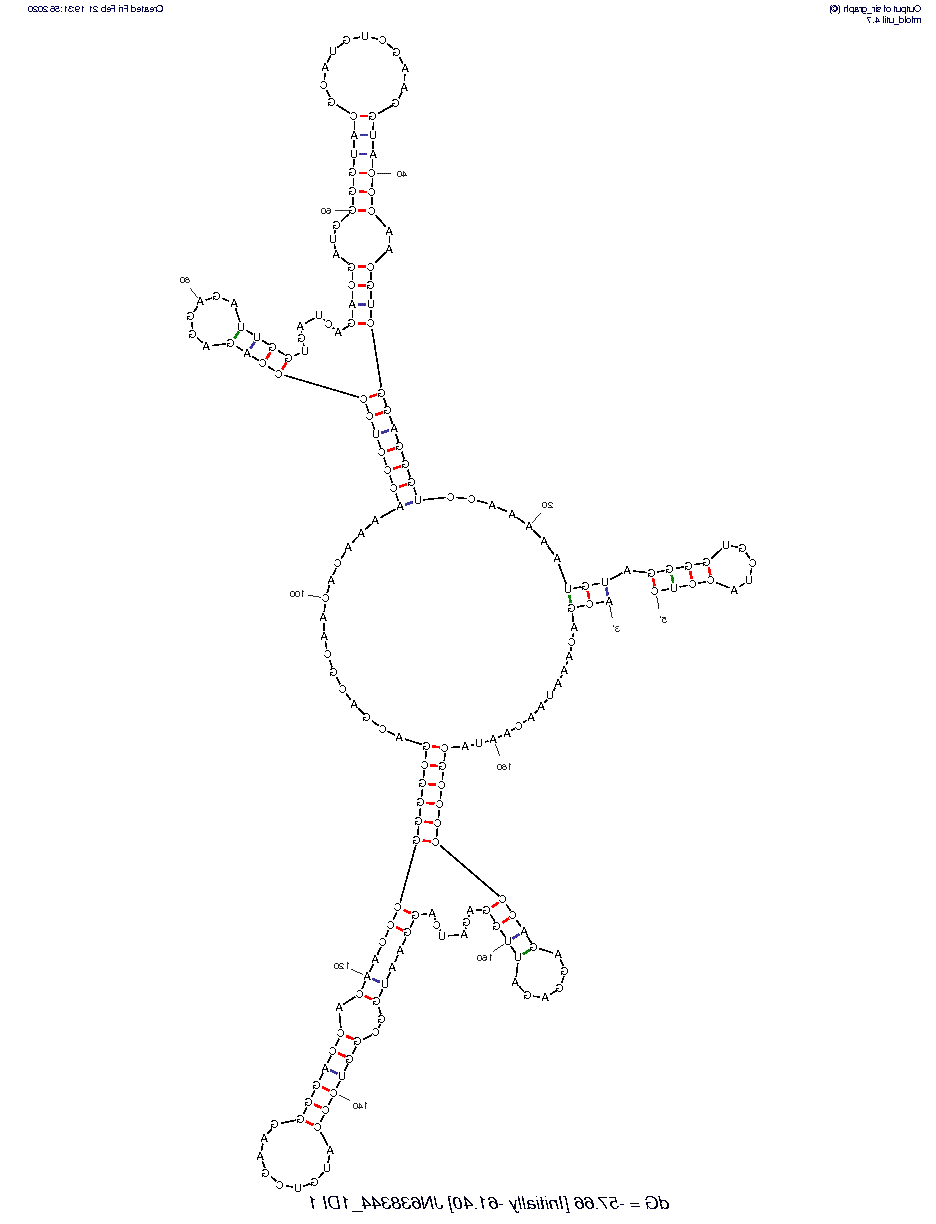 DB1  DB2 | -61.40  kcal/mol | 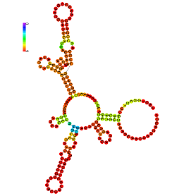  DB2  DB1 | -63.57  kcal/mol | 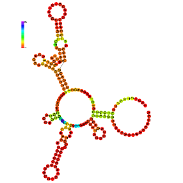 |
|  | U88537 | 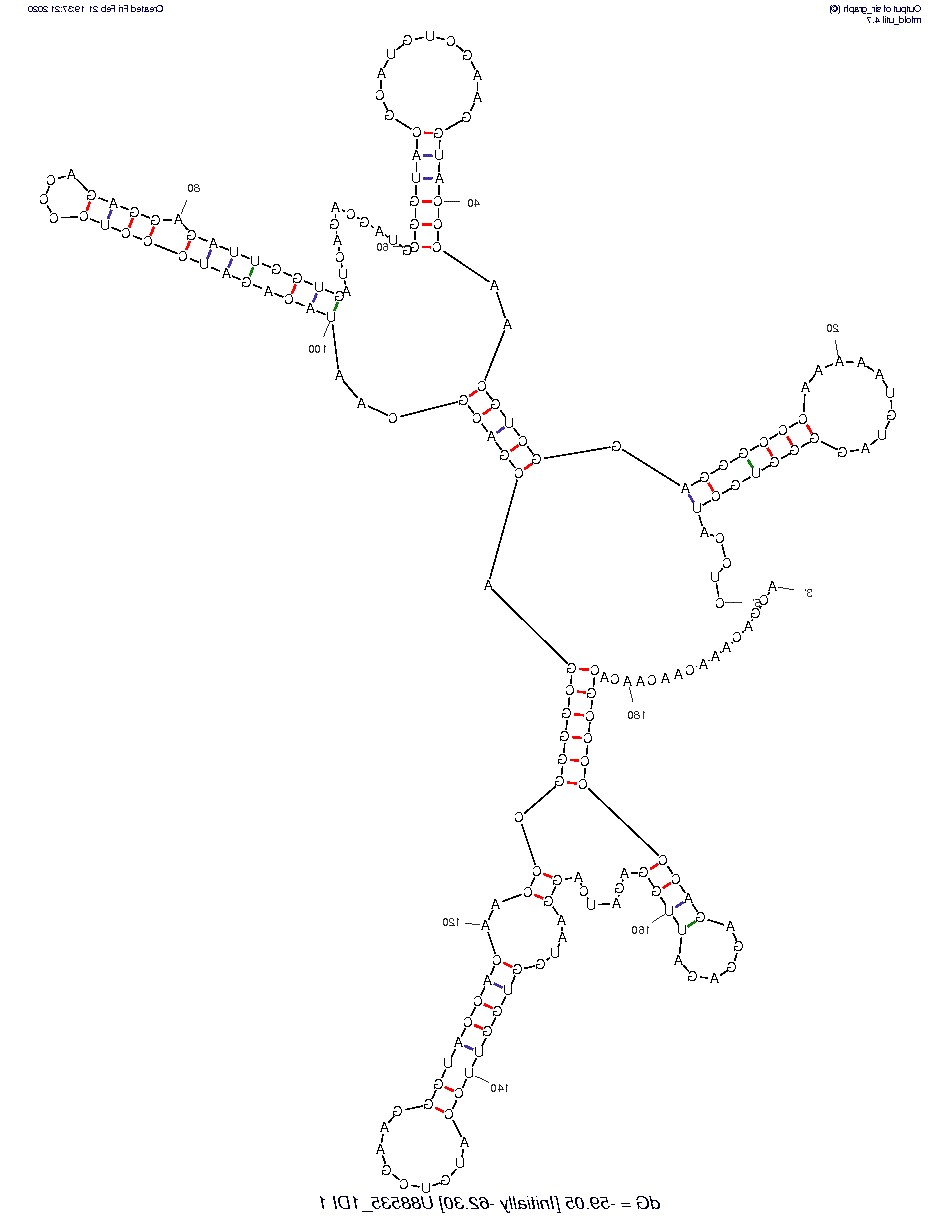 DB1  DB2 | -62.30  kcal/mol | 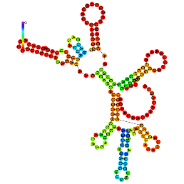  DB1  DB2 | -64.38 kcal/mol | 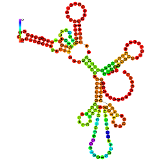 |
| **DENV1** | | **Mfold predicted secondary structures** | | **RNAfold predicted secondary structures** | | |
|  |  | **MFE structure** | | **MFE structure** | | **Centroid structure** |
| DENV1 Genotype III | 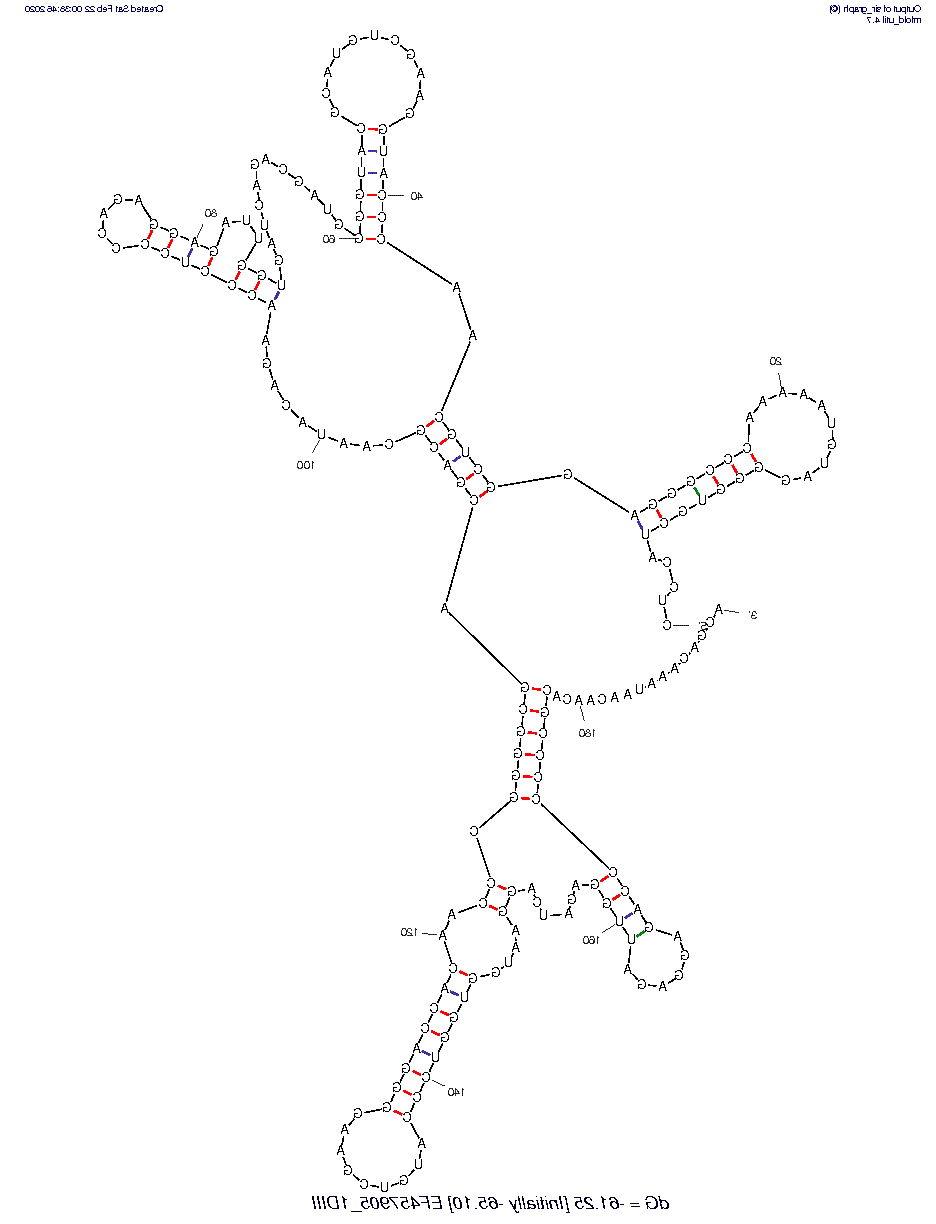 DB1    DB2  EF457905 |  | -65.10  kcal/mol | DB1  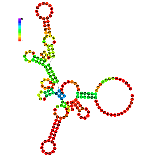  DB2 | -66.68  kcal/mol | 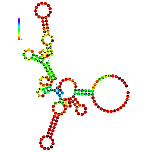 |

| **DENV1** | | **Mfold predicted secondary structures** | | **RNAfold predicted secondary structures** | | |
| --- | --- | --- | --- | --- | --- | --- |
|  |  | **MFE structure** | | **MFE structure** | | **Centroid structure** |
| DENV1 Genotype IV | DQ672564 | DB1  DB2 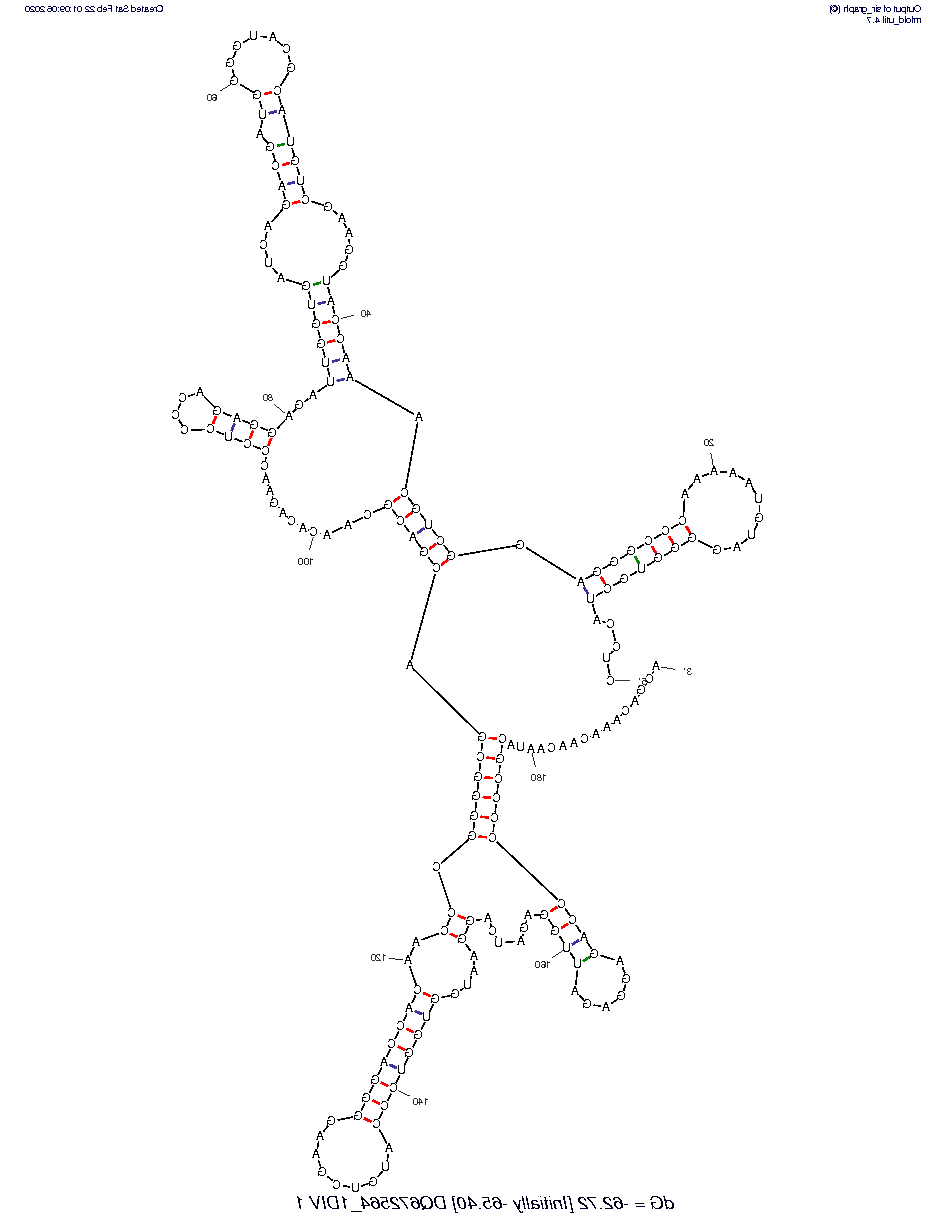 | -65.40  kcal/mol | 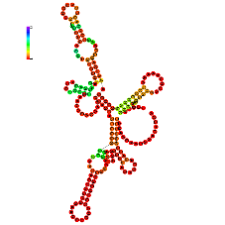  DB2  DB1 | -65.98  kcal/mol | 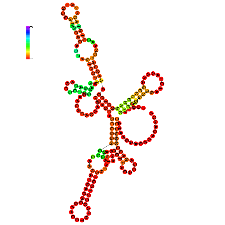 |
|  | EU863650 | 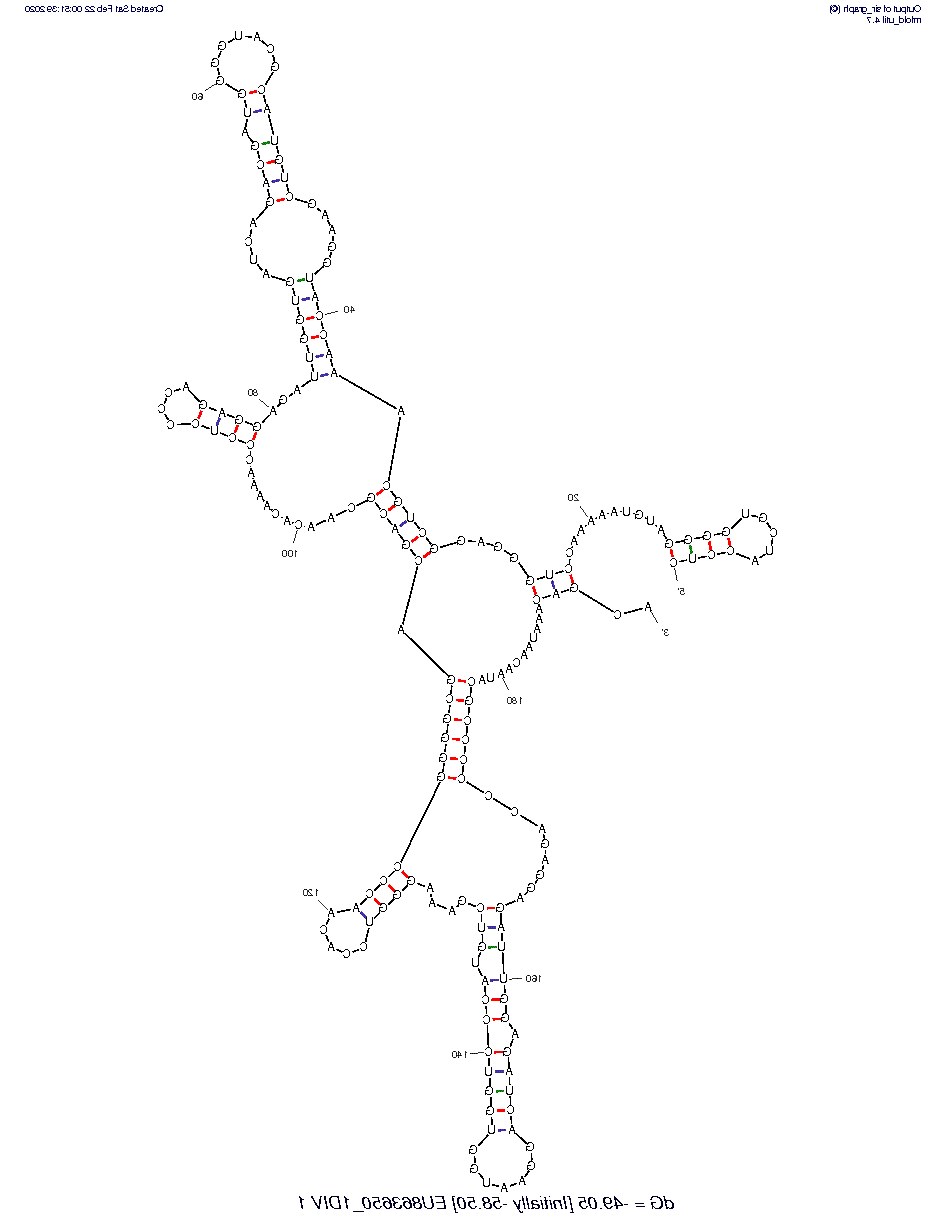 DB1  DB2 | -58.50  kcal/mol | 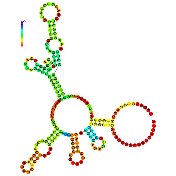  DB2  DB1 | -59.61 kcal/mol | 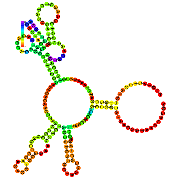 |
|  | FJ196842 | 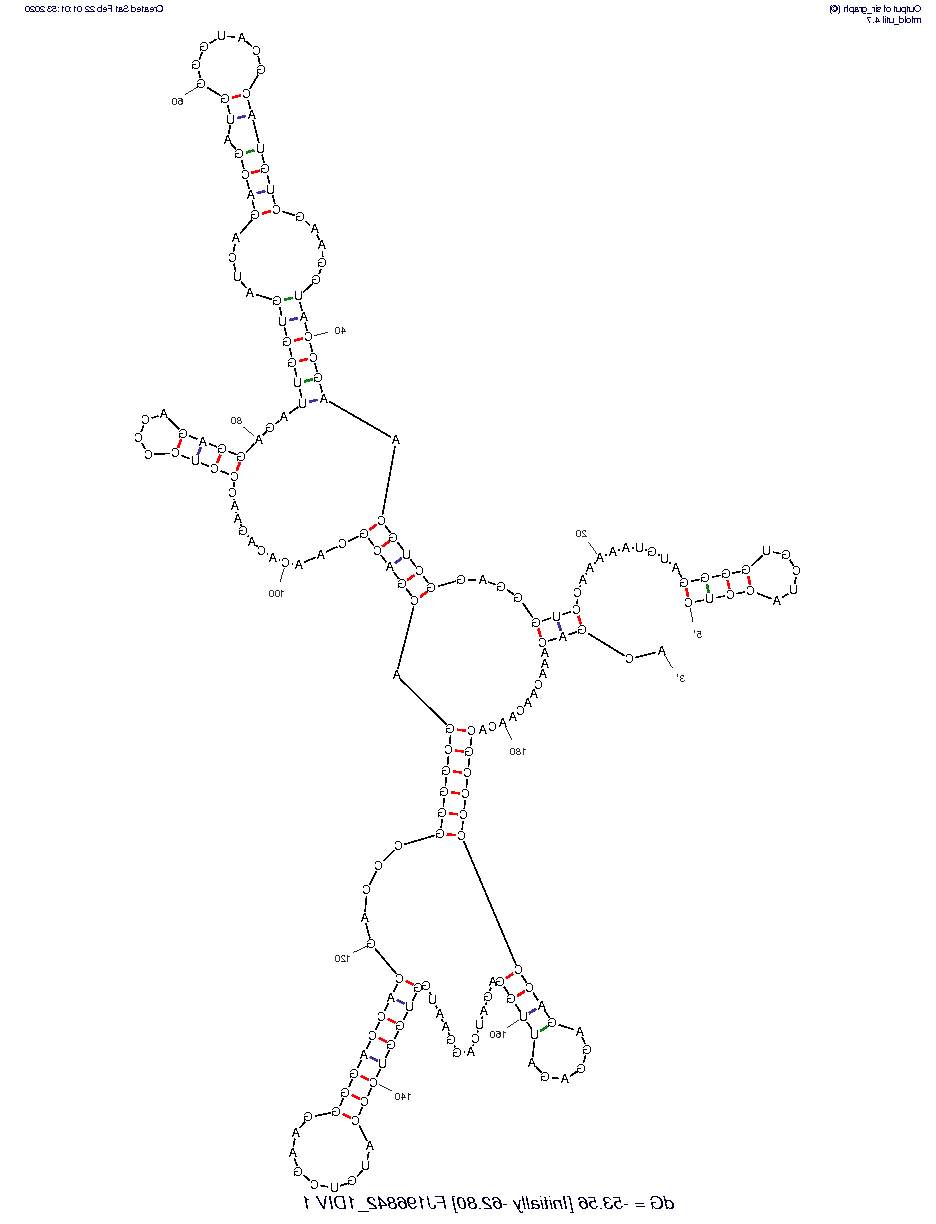 DB1  DB2 | -62.80  kcal/mol | 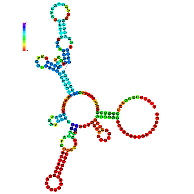  DB1  DB2 | -64.39  kcal/mol | 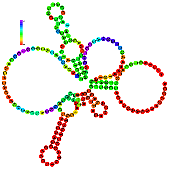 |
|  | FJ196845 | 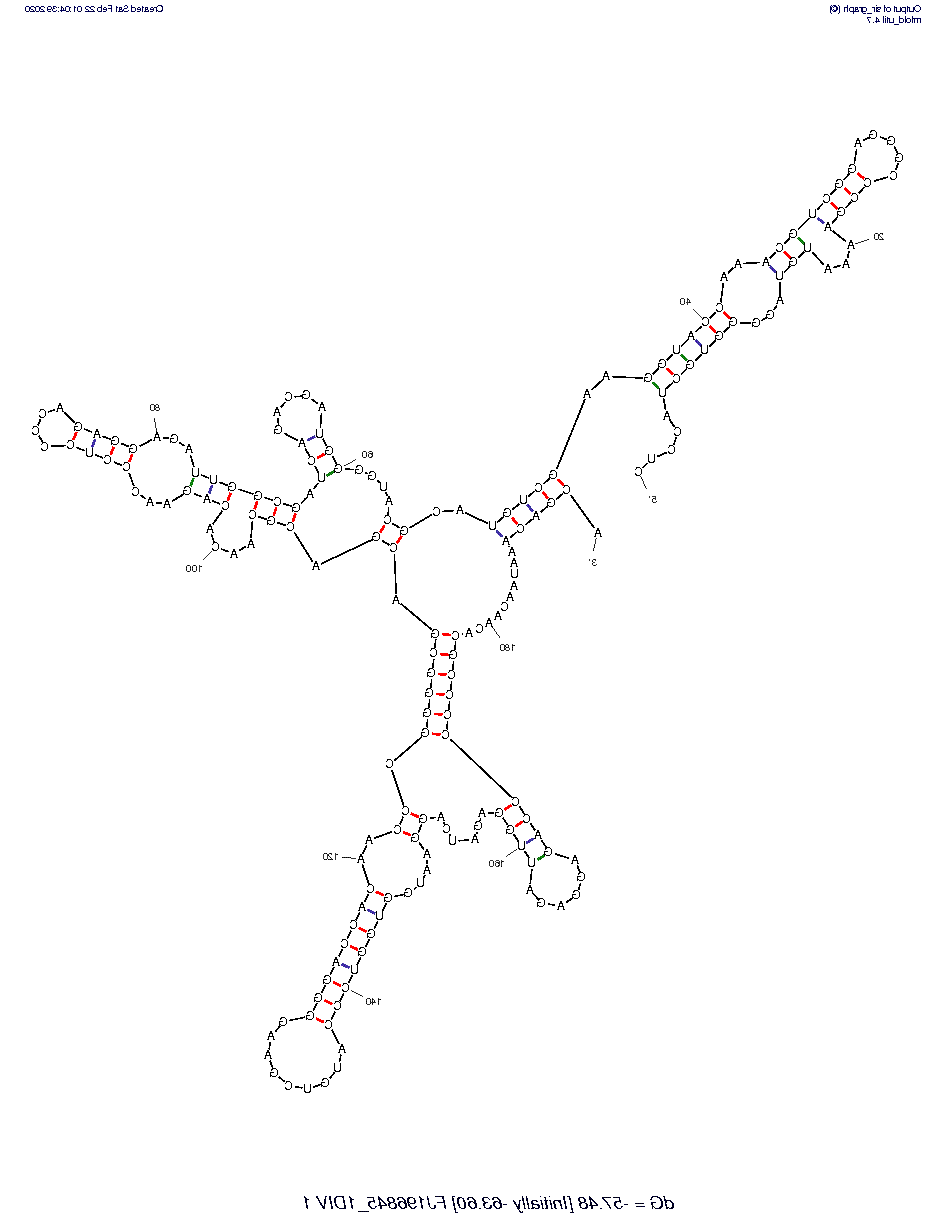 DB1  DB2 | -63.60  kcal/mol | 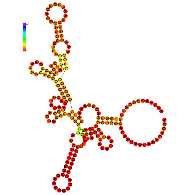  DB2  DB1 | -65.31  kcal/mol | 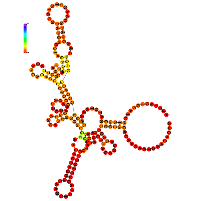 |
|  | 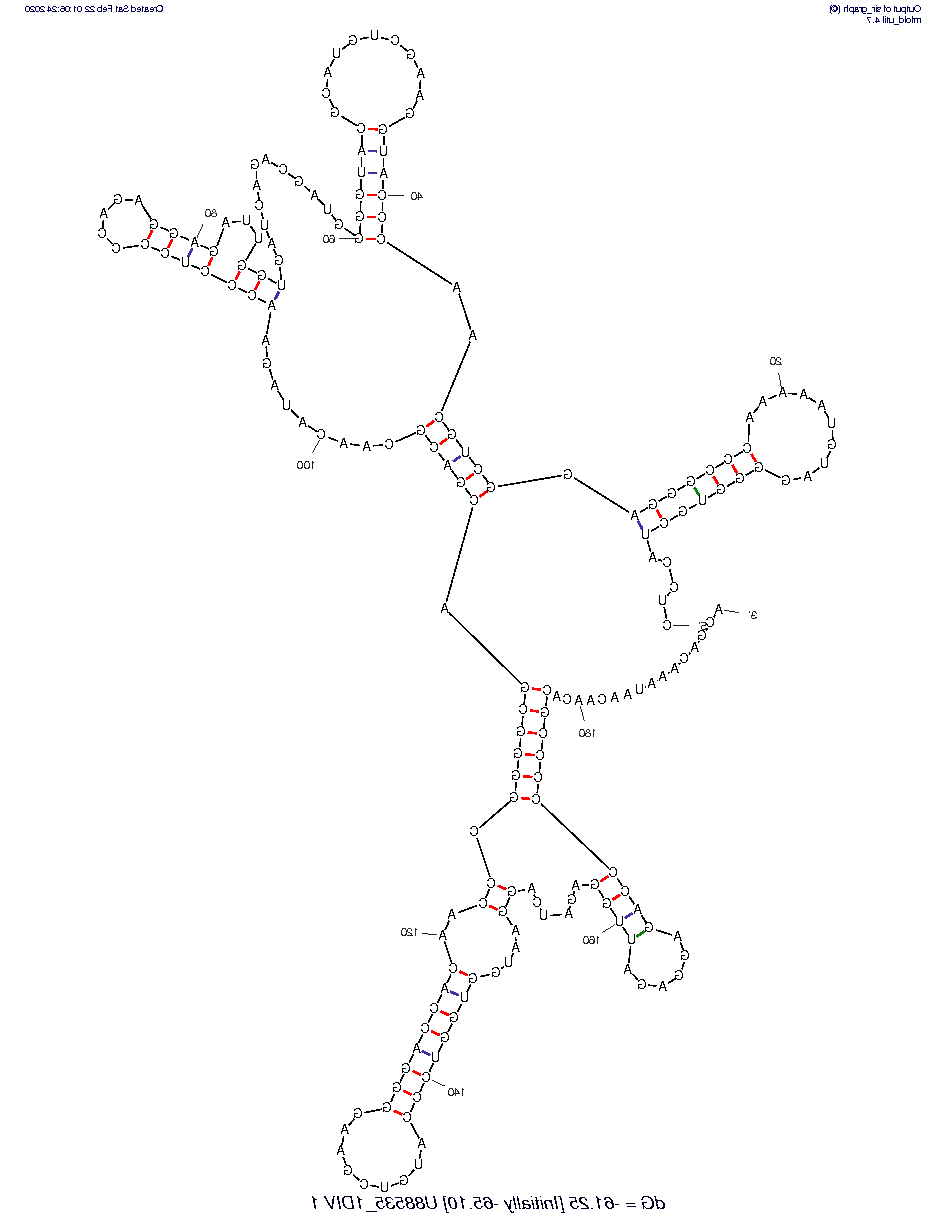 DB1  DB2  U88535 |  | -65.10  kcal/mol | 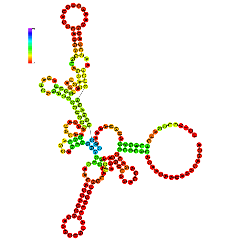  DB2  DB1 | -66.62  kcal/mol | 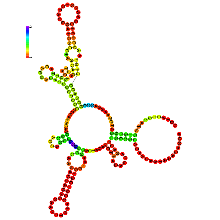 |
| **DENV1** | | **Mfold predicted secondary structures** | | **RNAfold predicted secondary structures** | | |
|  |  | **MFE structure** | | **MFE structure** | | **Centroid structure** |
| DENV1 Genotype V | 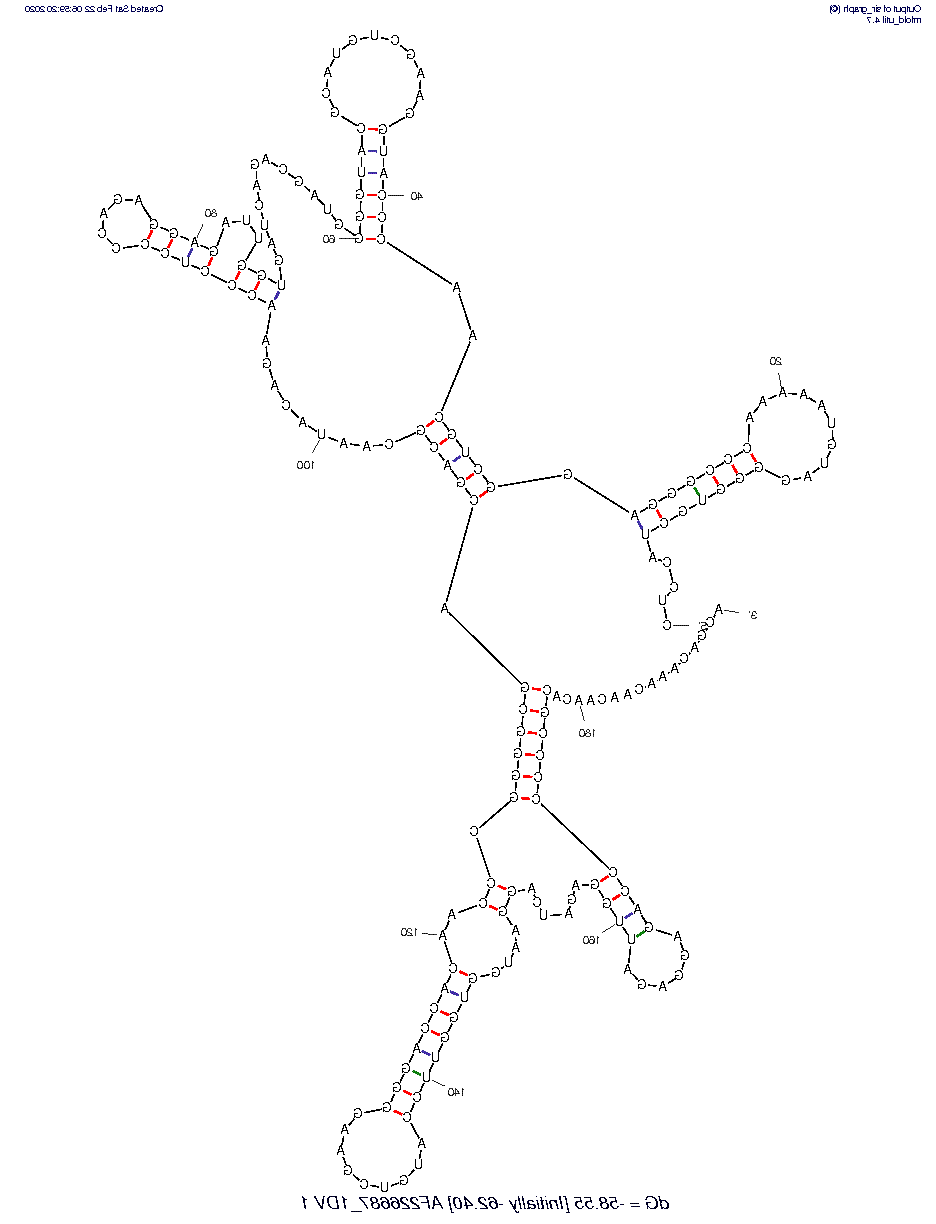 DB1  DB2  AF226687  EU596501  KC692512 |  | -62.40  kcal/mol | 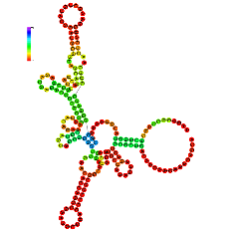  DB2  DB1 | -63.99  kcal/mol | 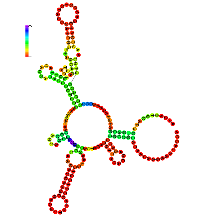 |
|  | 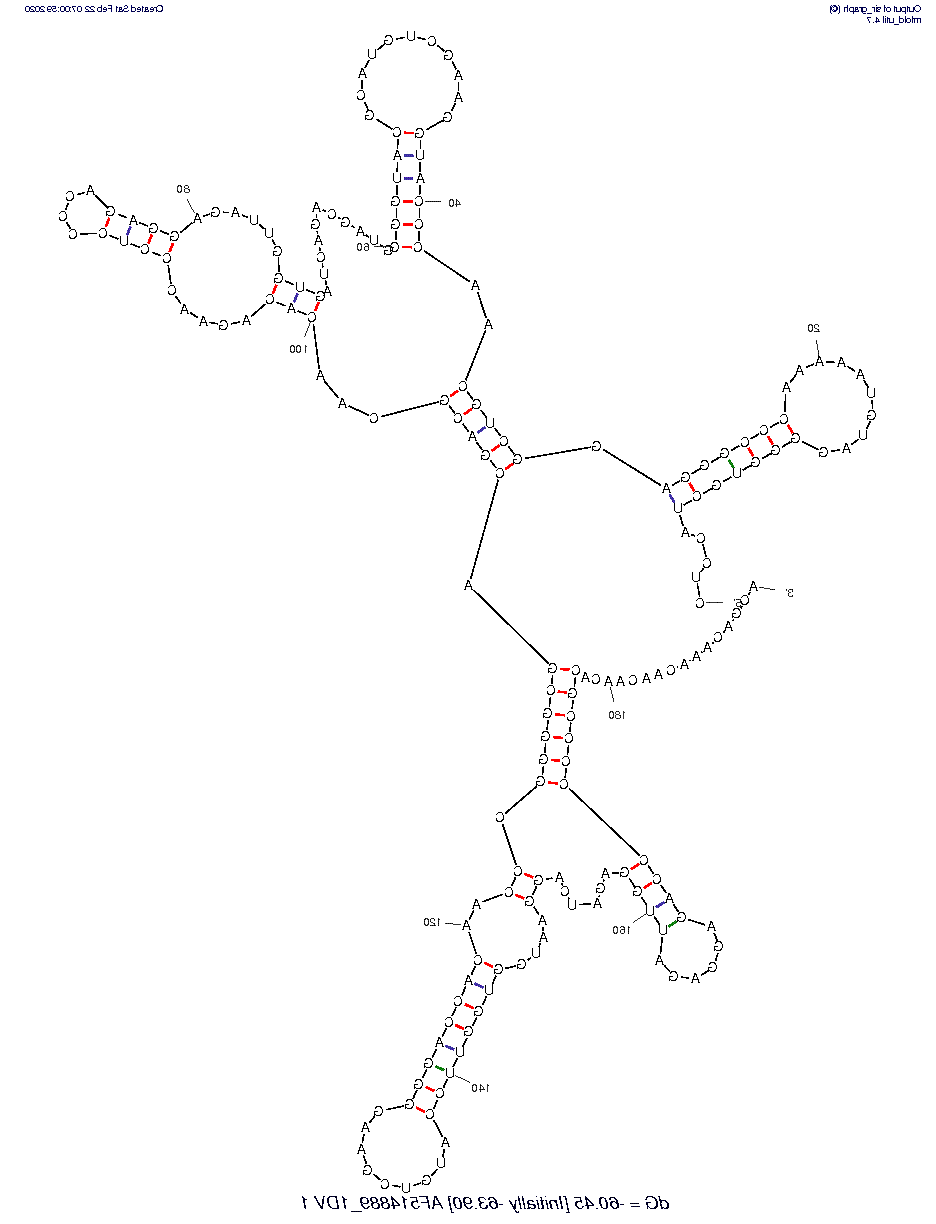   DB1  DB2  AF514889 |  | -60.45  kcal/mol | 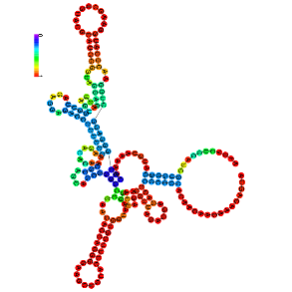  DB2  DB1 | -64.42  kcal/mol | 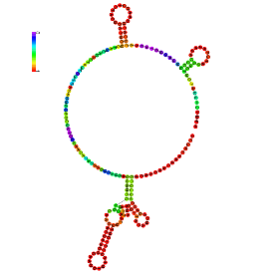 |
|  | AY732474  AY732476  AY762084  JN903581  KF289072 | 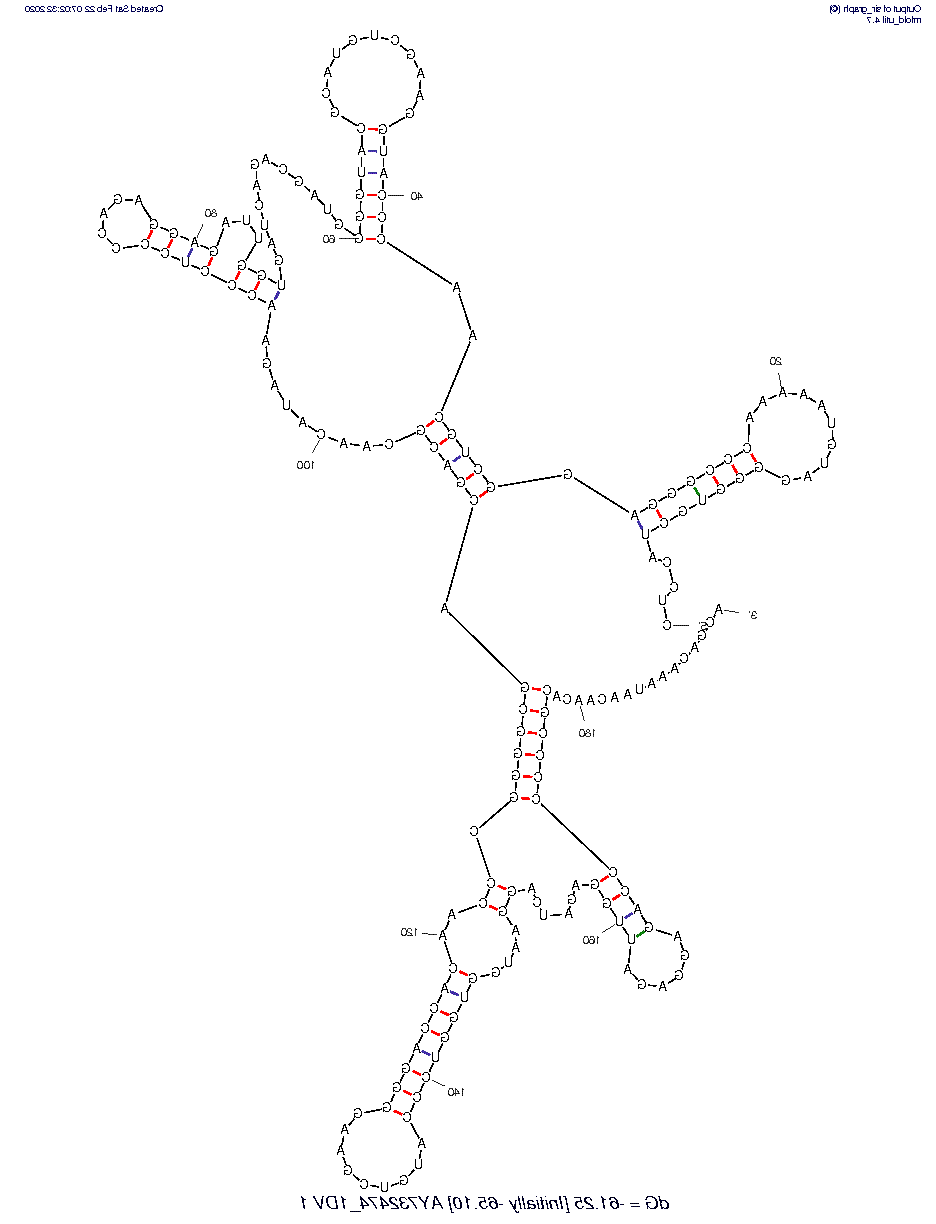 DB1  DB2 | -65.10  kcal/mol | 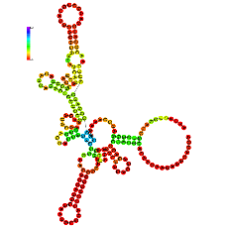  DB2  DB1 | -66.42 kcal/mol | 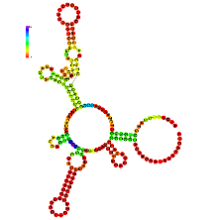 |
|  | AF514883 | DB2  DB1 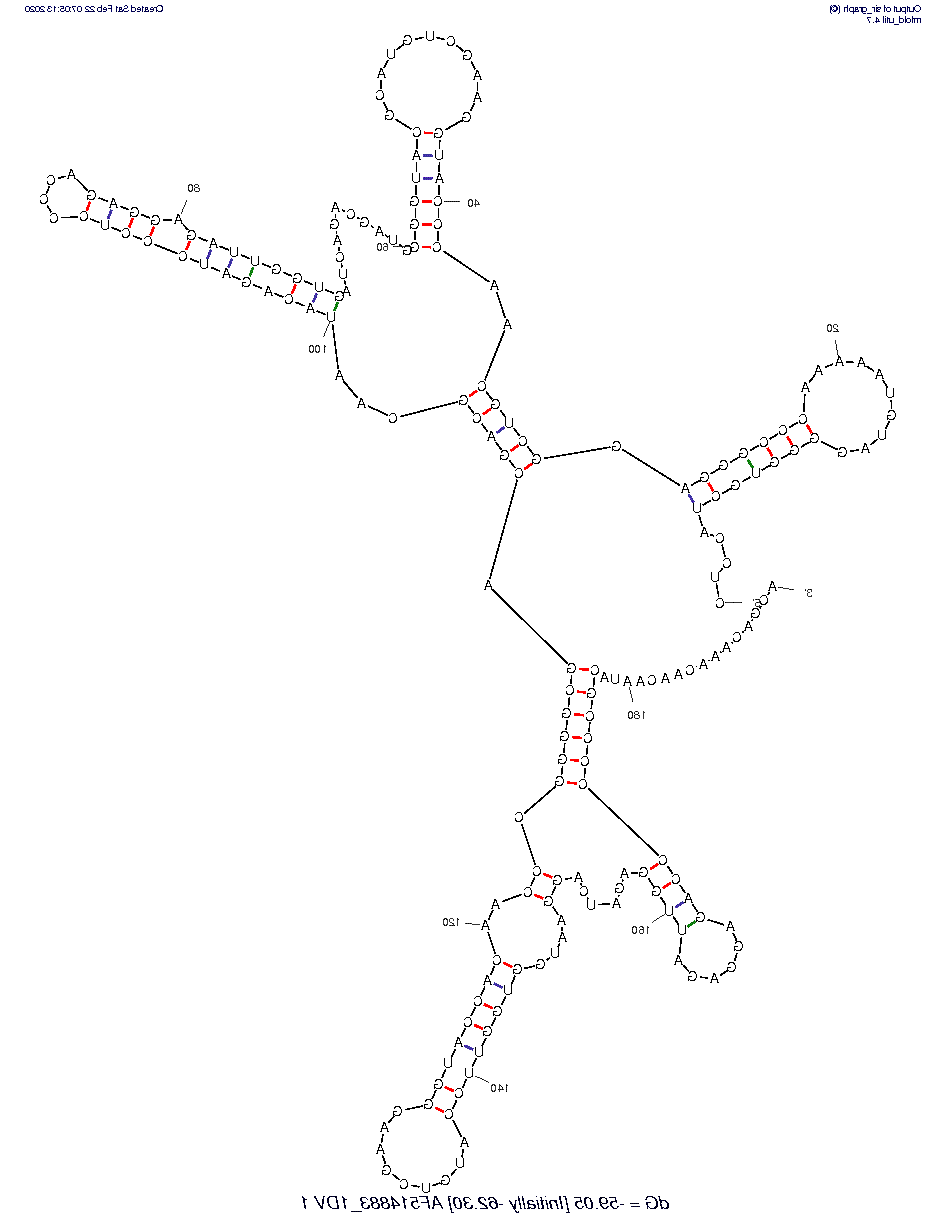 | -62.30 kcal/mol | 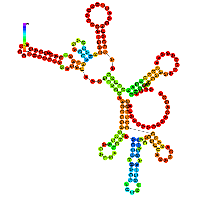  DB2  DB1 | -64.38 kcal/mol | 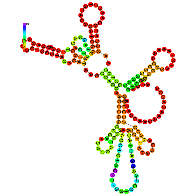 |
|  | AF298808 | 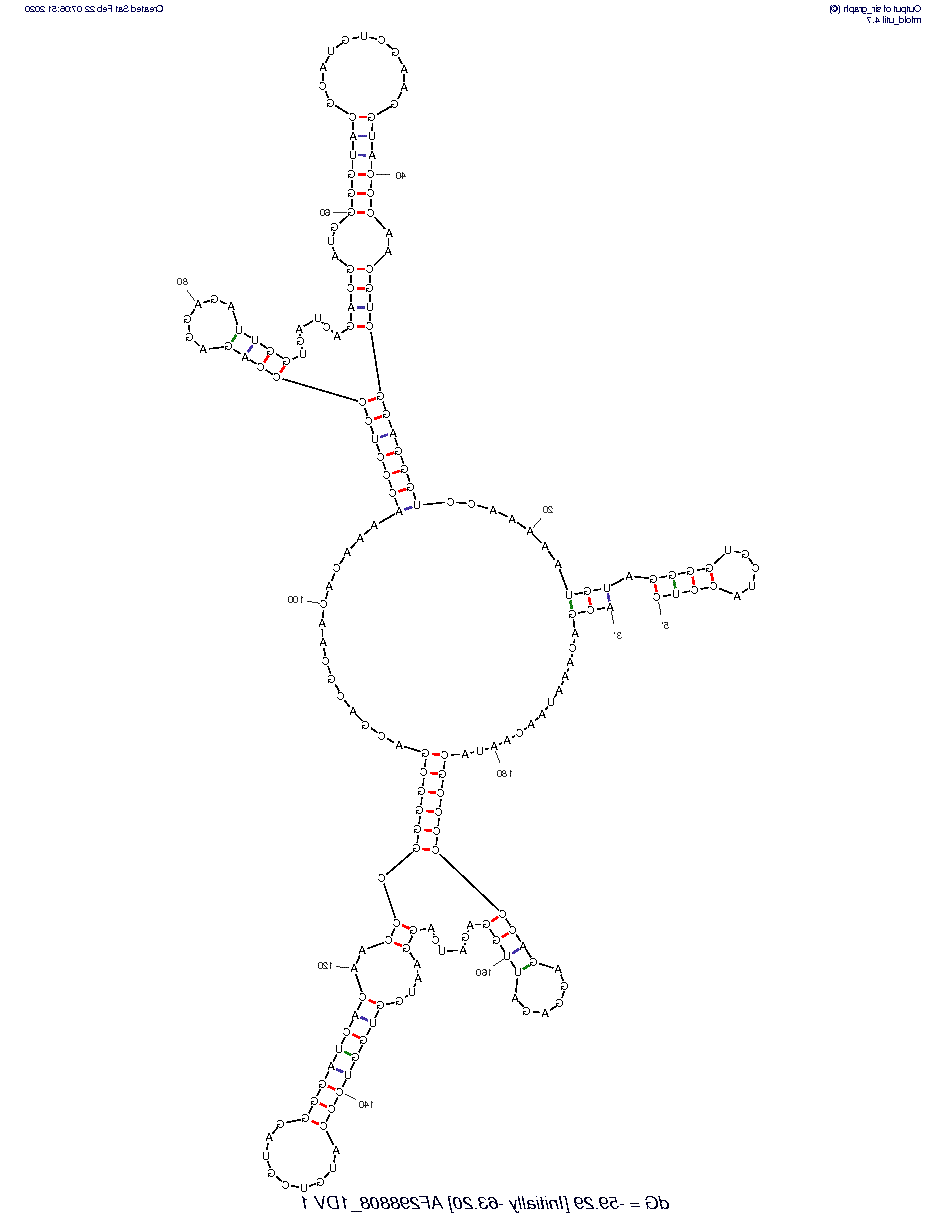 DB1  DB2 | -62.80 kcal/mol | 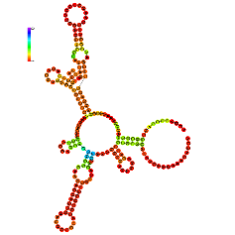  DB2  DB1 | -65.10 kcal/mol | 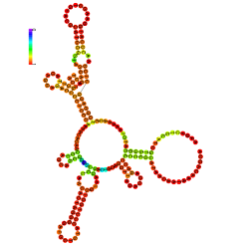 |

| **DENV1** | | **Mfold predicted secondary structures** | | | **RNAfold predicted secondary structures** | | | |
| --- | --- | --- | --- | --- | --- | --- | --- | --- |
|  |  | **MFE structure** | | | **MFE structure** | | | **Centroid structure** |
| DENV1 Genotype V | EU081258 | 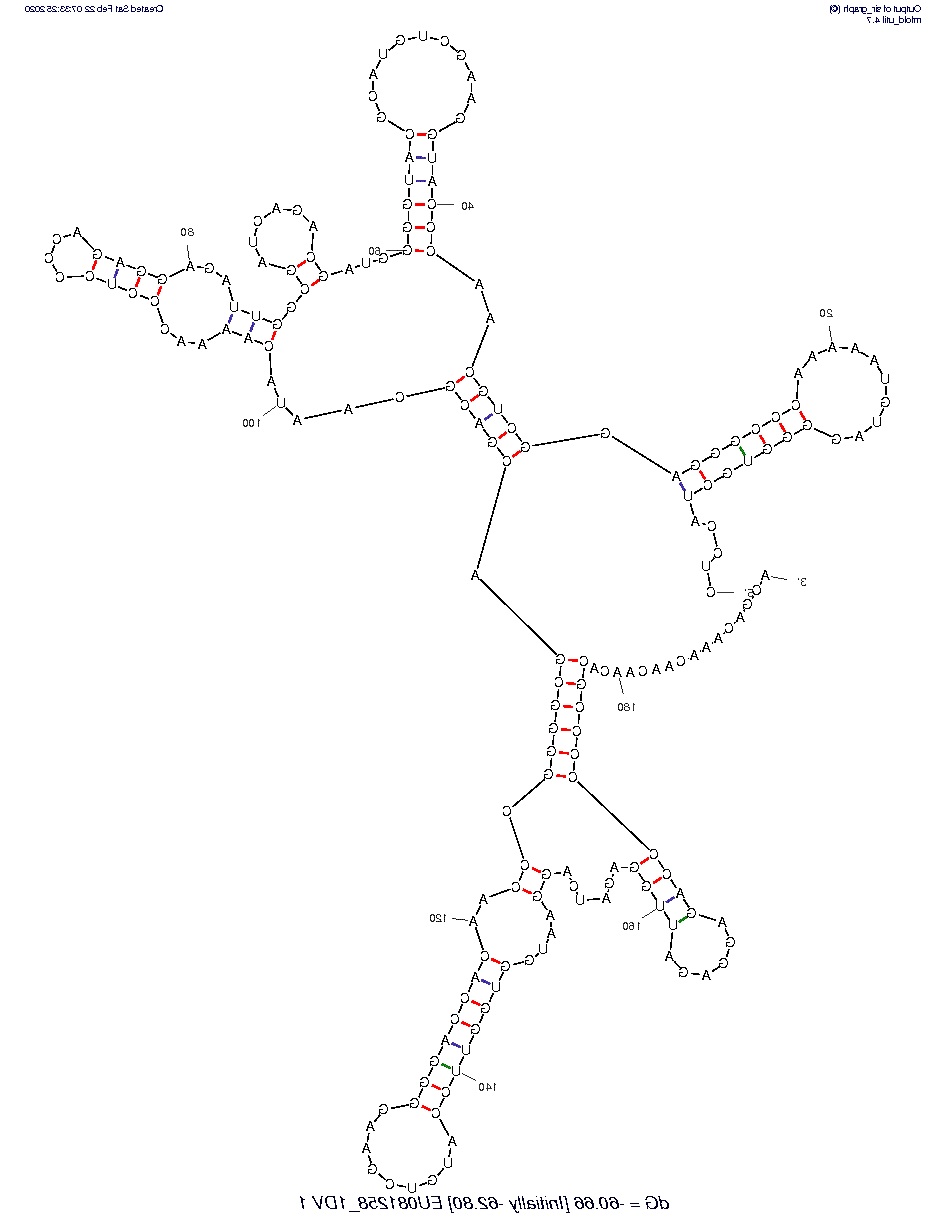 DB1  DB2 | | -62.80  kcal/mol | 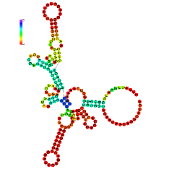  DB2  DB1 | | -64.47  kcal/mol | 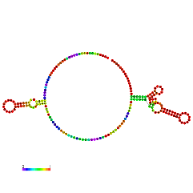 |
|  | GU13196 | 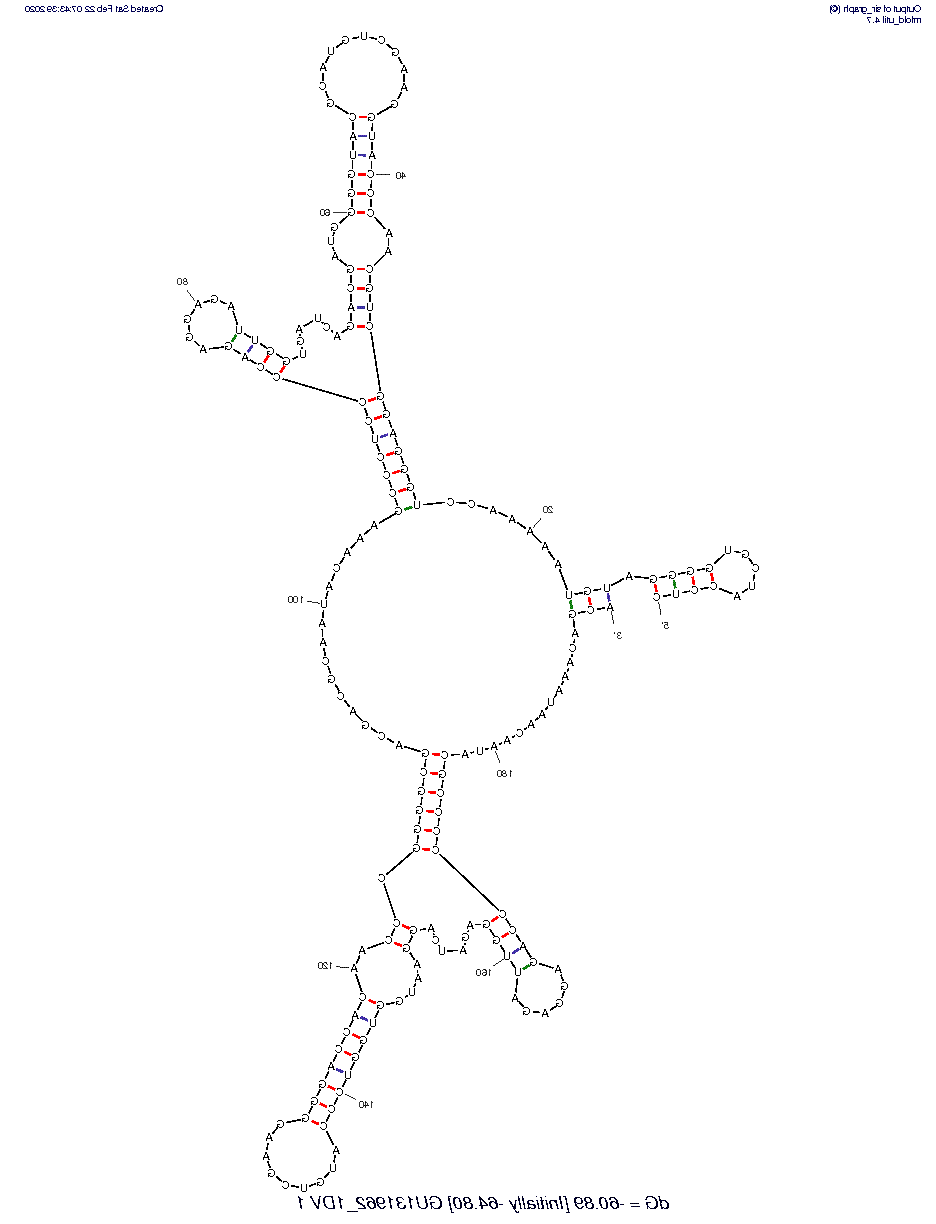 DB1  DB2 | | -64.80  kcal/mol | DB2  DB1 | | -66.70  kcal/mol |  |
|  | HQ332182 | | DB1  DB2 | | -60.45  kcal/mol | DB2  DB1 | -64.41  kcal/mol |  |
|  | JN903579 | DB2  DB1 | | -65.40  kcal/mol | DB2  DB1 | | -65.98  kcal/mol |  |
|  | JQ915080 | DB1  DB2 | | -58.50  kcal/mol | DB2  DB1 | | -59.61  kcal/mol |  |

| **DENV1** | | **Mfold predicted secondary structures** | | **RNAfold predicted secondary structures** | | |
| --- | --- | --- | --- | --- | --- | --- |
|  |  | **MFE structure** | | **MFE structure** | | **Centroid structure** |
| DENV1 Genotype V | JQ922544 | DB1  DB2 | -63.70  kcal/mol | DB2  DB1 | -64.04  kcal/mol |  |
|  | JQ922546 | DB1  DB2 | -28.64  kcal/mol | DB2  DB1 | -67.09  kcal/mol |  |
|  | JQ922548 | DB1  DB2 | -63.51  kcal/mol | DB1  DB2 | -66.34  kcal/mol |  |
|  | M87512 | DB1  DB2 | -60.80  kcal/mol | DB2  DB1 | -61.14  kcal/mol |  |

*SNVs for each DENV1 isolate structure is highlighted in red on each Mfold Predicted structure*. *Base pairing probability in MEF and Centroid structures of RNA predicted secondary structures, is denoted by the colored nucleotides. Colours are rated from 1-0 to indicate strong to weak base pairing probabilities (Red - strongest probability, Green - medium probability, Blue - lowest probability) in the colour scale.*
