# Supplementary material for: Genetic Variation in the Domain II, 3′ Untranslated Region of Human and Mosquito Derived Dengue Virus Strains in Sri Lanka
Source: Viruses. 2021 Mar 5;13(3):421. doi: 10.3390/v13030421 (PMC8001906; doi:10.3390/v13030421)
Supplement: Supplementary file 1 [file viruses-13-00421-s001.zip › Supplimentry files/Supplimentry tables/Table S7.pdf]

Table S7. Mfold and RNAfold predicted secondary structures for RNA alignments of DENV1, Domain II region of 3'UTR sequences identified in the study, Sri Lankan isolates and DENV1 reference genotypes.

DENV1		Mfold predicted secondary structures		RNAfold predicted secondary structures		
		MFE structure		MFE structure		Centroid structure
**DENV1 reference**	EU848545	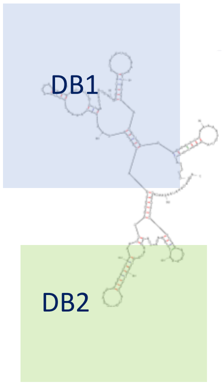	-65.10 kcal/mol	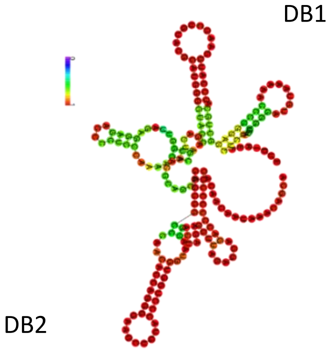	-64.10 kcal/mol	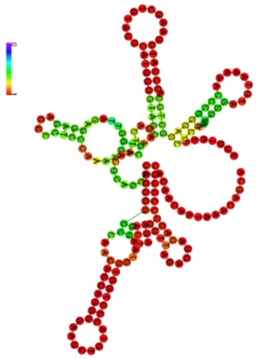
**Study**	D1H_2019SL	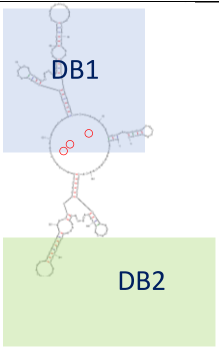	-65.50 kcal/mol	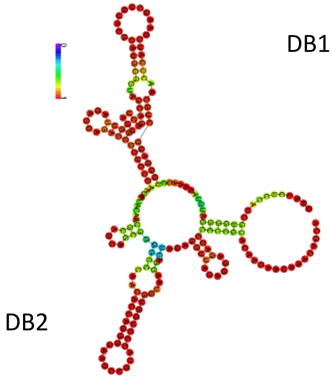	-64.40 kcal/mol	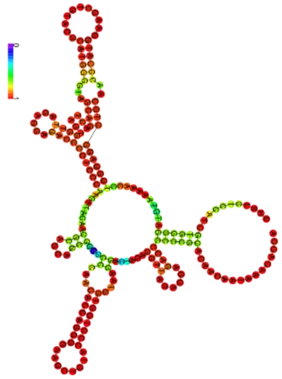

DENV1		Mfold predicted secondary structures		RNAfold predicted secondary structures		
		MFE structure		MFE structure		Centroid structure
Sri Lankan Isolates of DENV1	HQ891314	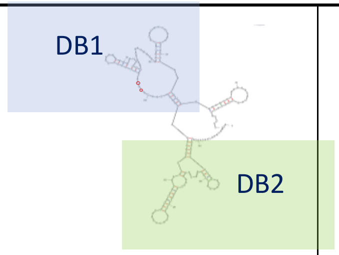	-65.10 kcal/mol	DB1 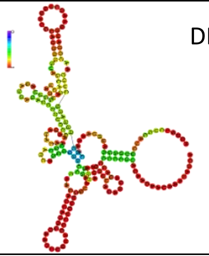 DB2	-66.62 kcal/mol	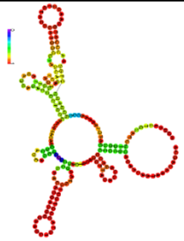
	KJ726664	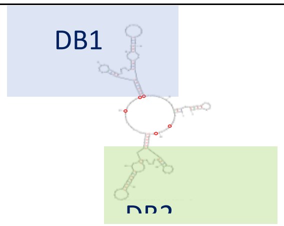	-64.80 kcal/mol	DB1 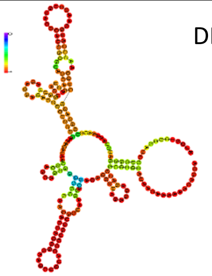 DB2	-66.62 kcal/mol	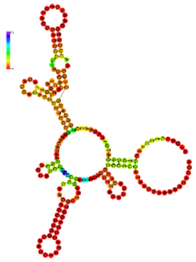
	KJ726665<br>KJ726663<br>HQ891315<br>JN054256<br>KJ468234<br>KJ726662<br>HQ891316<br>JN054255	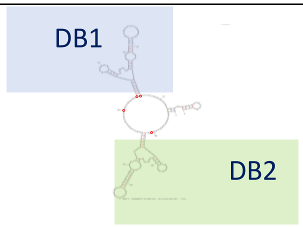	-64.80 kcal/mol	DB1 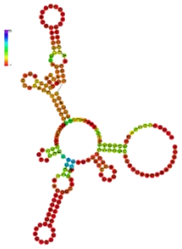 DB2	-66.63 kcal/mol	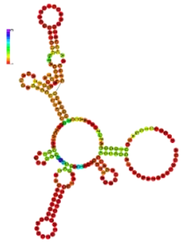
	KP398852	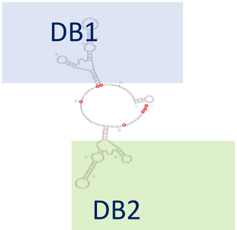	-64.30 kcal/mol	DB1 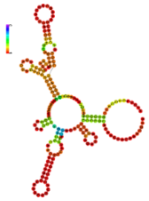 DB2	-66.61 kcal/mol	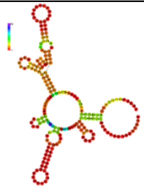

DENV1		Mfold predicted secondary structures		RNAfold predicted secondary structures		
		MFE structure		MFE structure		Centroid structure
DENV1 Genotype I	AF309641	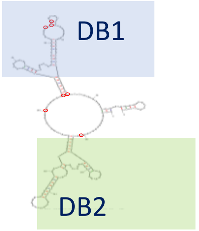	-61.29 kcal/mol	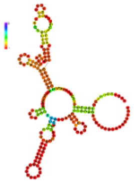	-65.12 kcal/mol	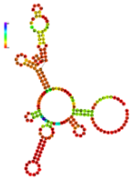
	AB074760	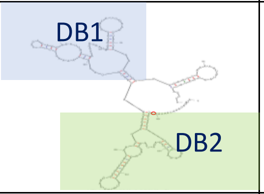	-66.60 kcal/mol	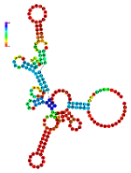	-67.06 kcal/mol	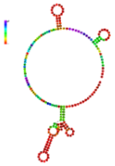
	AF350498<br>AY732477<br>JN638342	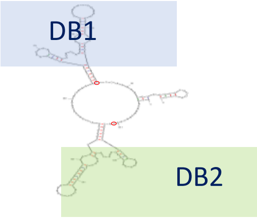	-61.59 kcal/mol	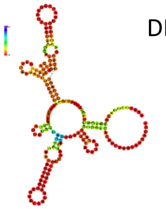	-67.11 kcal/mol	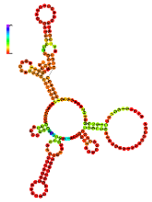
	AF298807	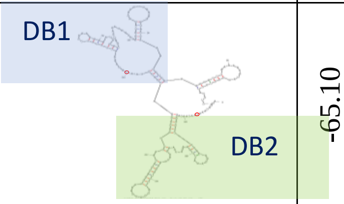	-65.10 kcal/mol	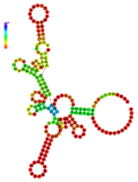	-66.63 kcal/mol	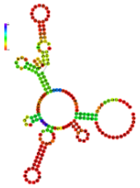
	AY726555<br>EU081226	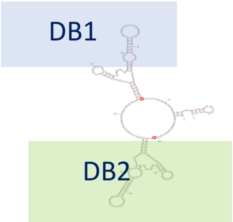	-61.59 kcal/mol	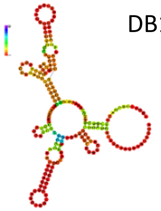	-67.11 kcal/mol	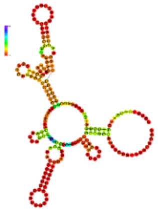

DENV1		Mfold predicted secondary structures		RNAfold predicted secondary structures		
		MFE structure		MFE structure		Centroid structure
DENV1 Genotype I	AY732479	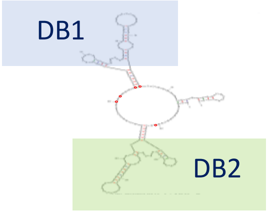	-64.80 kcal/mol	DB1 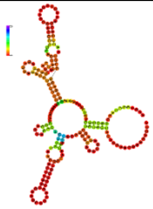 DB2	-60.49 kcal/mol	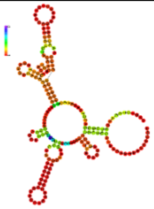
	AY732480	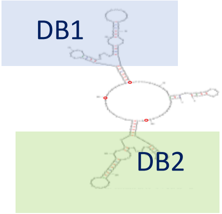	-60.49 kcal/mol	DB1 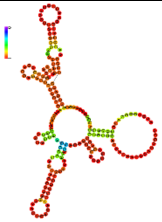 DB2	-63.92 kcal/mol	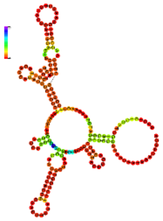
	AY732483	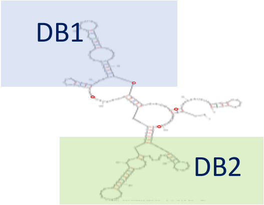	-63.80 kcal/mol	DB1 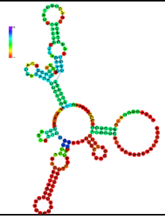 DB2	-64.15 kcal/mol	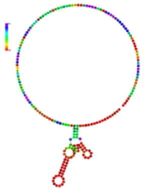
	AY835999<br>HQ891316<br>KJ726622	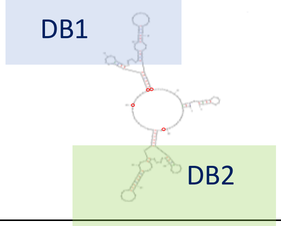	-64.80 kcal/mol	DB1 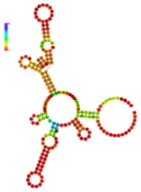 DB2	-66.63 kcal/mol	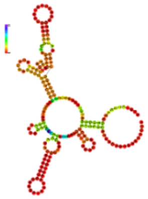
	DQ285561	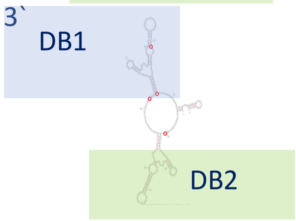	-60.80 kcal/mol	DB1 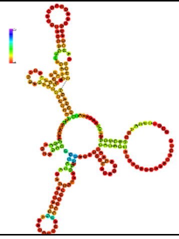 DB2	-60.88 kcal/mol	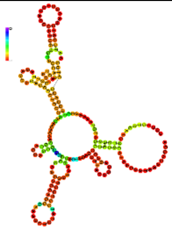

DENV1		Mfold predicted secondary structures		RNAfold predicted secondary structures		
		MFE structure		MFE structure		Centroid structure
DENV1 Genotype I	HG316481	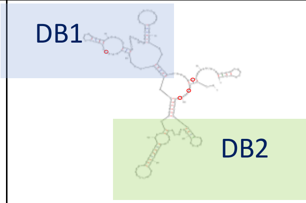	-65.00 kcal/mol	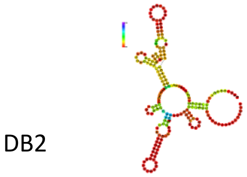	-66.65 kcal/mol	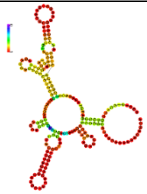
	HG316482	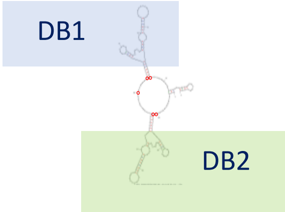	-64.90 kcal/mol	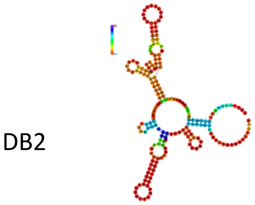	-66.98 kcal/mol	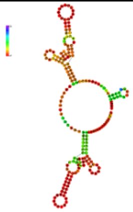
	JN638340	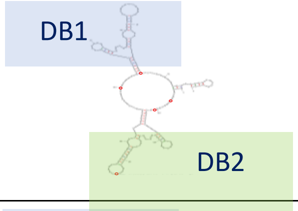	-64.40 kcal/mol	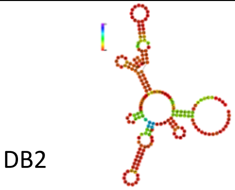	-63.96 kcal/mol	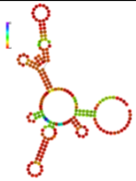
	JN638344	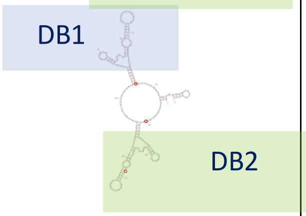	-61.40 kcal/mol	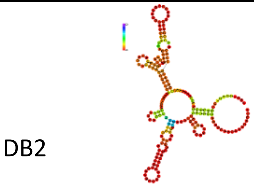	-63.57 kcal/mol	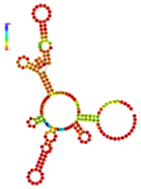
	U88537	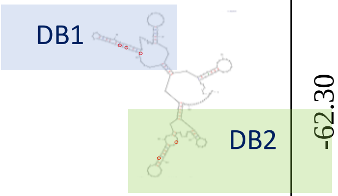	-62.30 kcal/mol	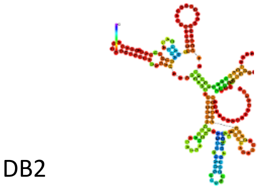	-64.38 kcal/mol	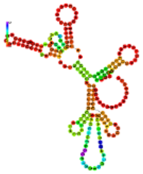

DENV1		Mfold predicted secondary structures		RNAfold predicted secondary structures		
		MFE structure		MFE structure		Centroid structure
DENV1 Genotype III	EF457905	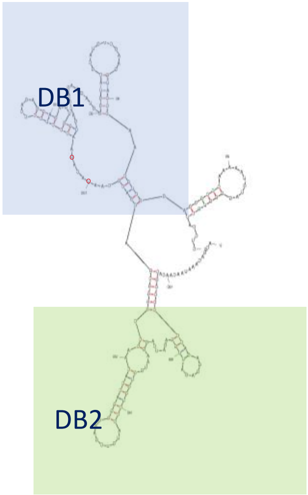	-65.10 kcal/mol	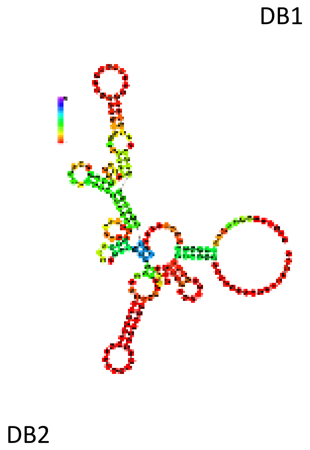	-66.68 kcal/mol	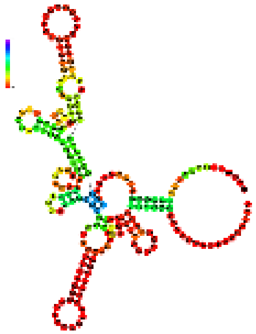

DENV1		Mfold predicted secondary structures		RNAfold predicted secondary structures		
		MFE structure		MFE structure		Centroid structure
DENV1 Genotype IV	DQ672564	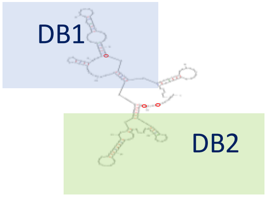	-65.40 kcal/mol	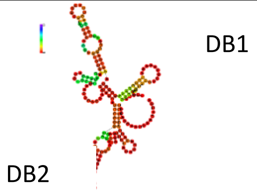	-65.98 kcal/mol	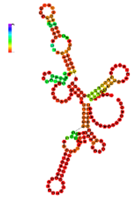
	EU863650	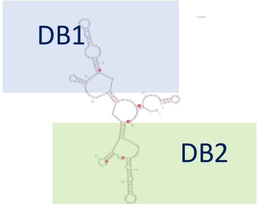	-58.50 kcal/mol	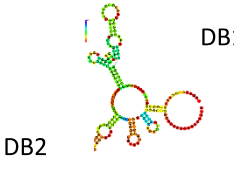	-59.61 kcal/mol	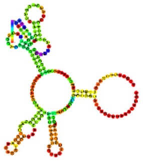
	FJ196842	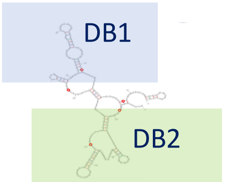	-62.80 kcal/mol	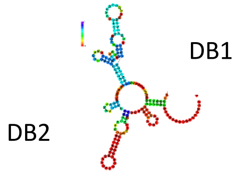	-64.39 kcal/mol	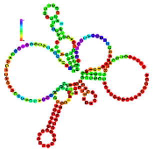
	FJ196845	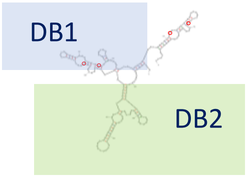	-63.60 kcal/mol	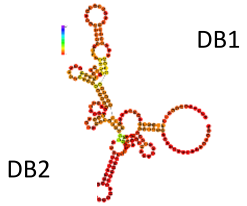	-65.31 kcal/mol	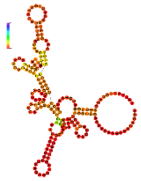
	U88535	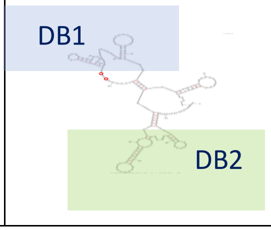	-65.10 kcal/mol	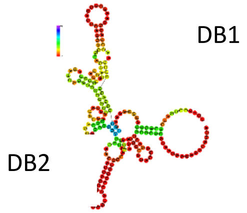	-66.62 kcal/mol	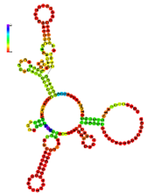

DENV1		Mfold predicted secondary structures		RNAfold predicted secondary structures		
		MFE structure		MFE structure		Centroid structure
DENV1 Genotype V	AF226687<br>EU596501<br>KC692512	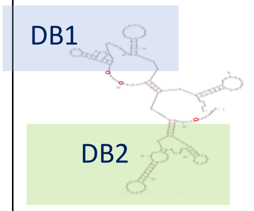	-62.40 kcal/mol	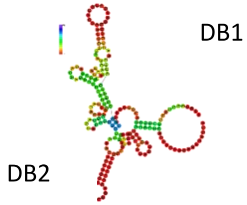	-63.99 kcal/mol	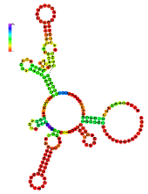
	AF514889	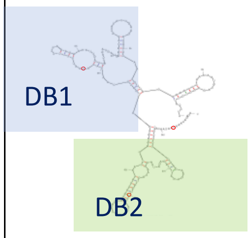	-60.45 kcal/mol	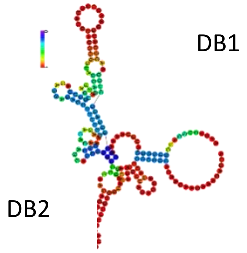	-64.42 kcal/mol	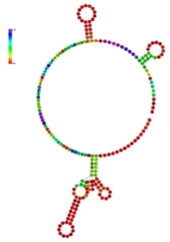
	AY732474<br>AY732476<br>AY762084<br>JN903581<br>KF289072	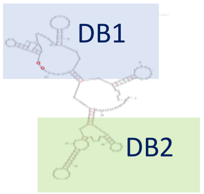	-65.10 kcal/mol	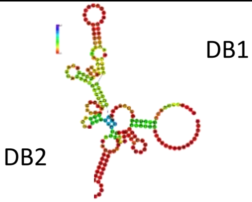	-66.42 kcal/mol	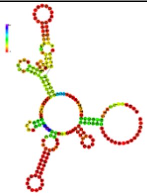
	AF514883	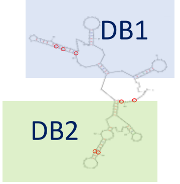	-62.30 kcal/mol	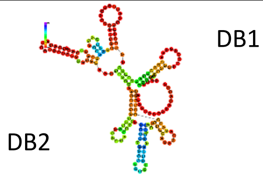	-64.38 kcal/mol	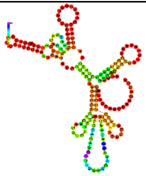
	AF298808	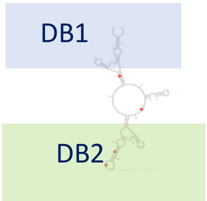	-62.80 kcal/mol	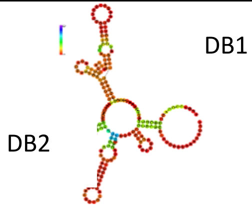	-65.10 kcal/mol	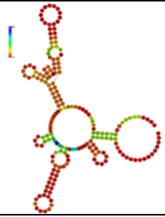

DENV1		Mfold predicted secondary structures		RNAfold predicted secondary structures		
		MFE structure		MFE structure		Centroid structure
DENV1 Genotype V	EU081258	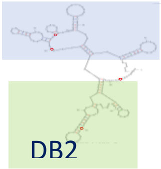	-62.80 kcal/mol	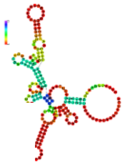 DB1 DB2	-64.47 kcal/mol	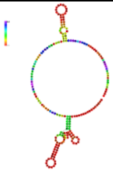
	GU13196	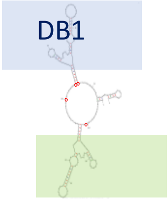	-64.80 kcal/mol	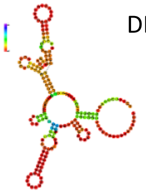 DB1 DB2	-66.70 kcal/mol	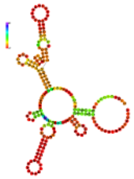
	HQ332182	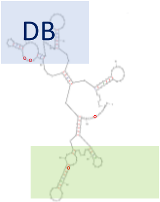		-60.45 kcal/mol 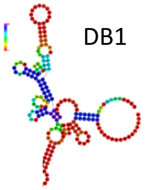 DB1 DB2	-64.41 kcal/mol	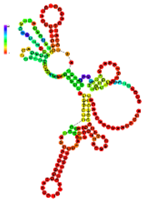
	JN903579	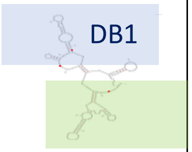	-65.40 kcal/mol	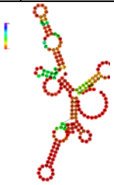 DB1 DB2	-65.98 kcal/mol	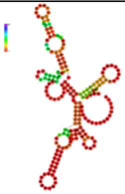
	JQ915080	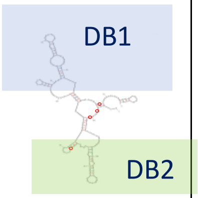	-58.50 kcal/mol	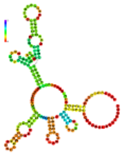 DB1 DB2	-59.61 kcal/mol	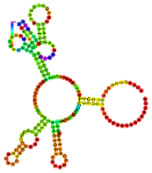

DENV1		Mfold predicted secondary structures		RNAfold predicted secondary structures		
		MFE structure		MFE structure		Centroid structure
DENV1 Genotype V	JQ922544	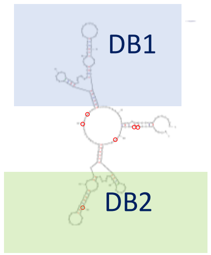	-63.70 kcal/mol	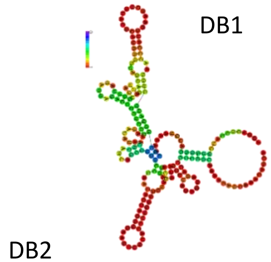	-64.04 kcal/mol	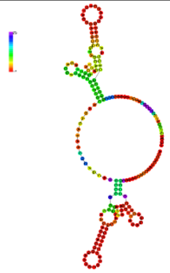
	JQ922546	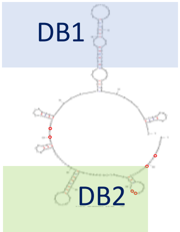	-28.64 kcal/mol	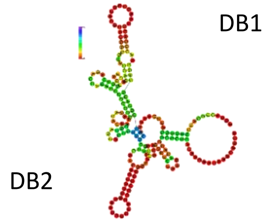	-67.09 kcal/mol	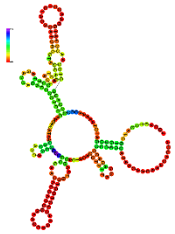
	JQ922548	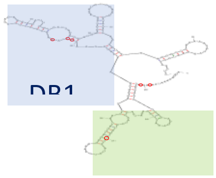	-63.51 kcal/mol	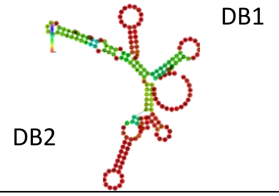	-66.34 kcal/mol	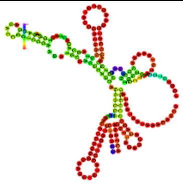
	M87512	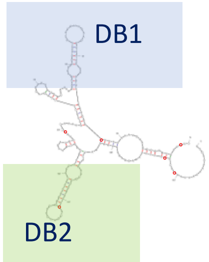	-60.80 kcal/mol	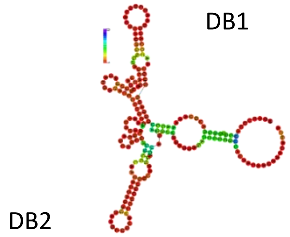	-61.14 kcal/mol	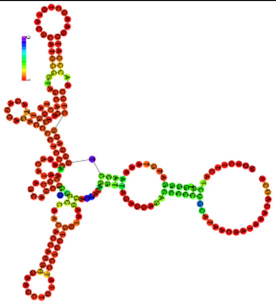

SNVs for each DENV1 isolate structure is highlighted in red on each Mfold Predicted structure. Base pairing probability in MEF and Centroid structures of RNA predicted secondary structures, is denoted by the colored nucleotides. Colours are rated from 1-0 to indicate strong to weak base pairing probabilities (Red - strongest probability, Green - medium probability, Blue - lowest probability) in the colour scale.
