# Supplementary figures and images for: Genetic Variation in the Domain II, 3′ Untranslated Region of Human and Mosquito Derived Dengue Virus Strains in Sri Lanka
Source: Viruses. 2021 Mar 5;13(3):421. doi: 10.3390/v13030421 (PMC8001906; doi:10.3390/v13030421)

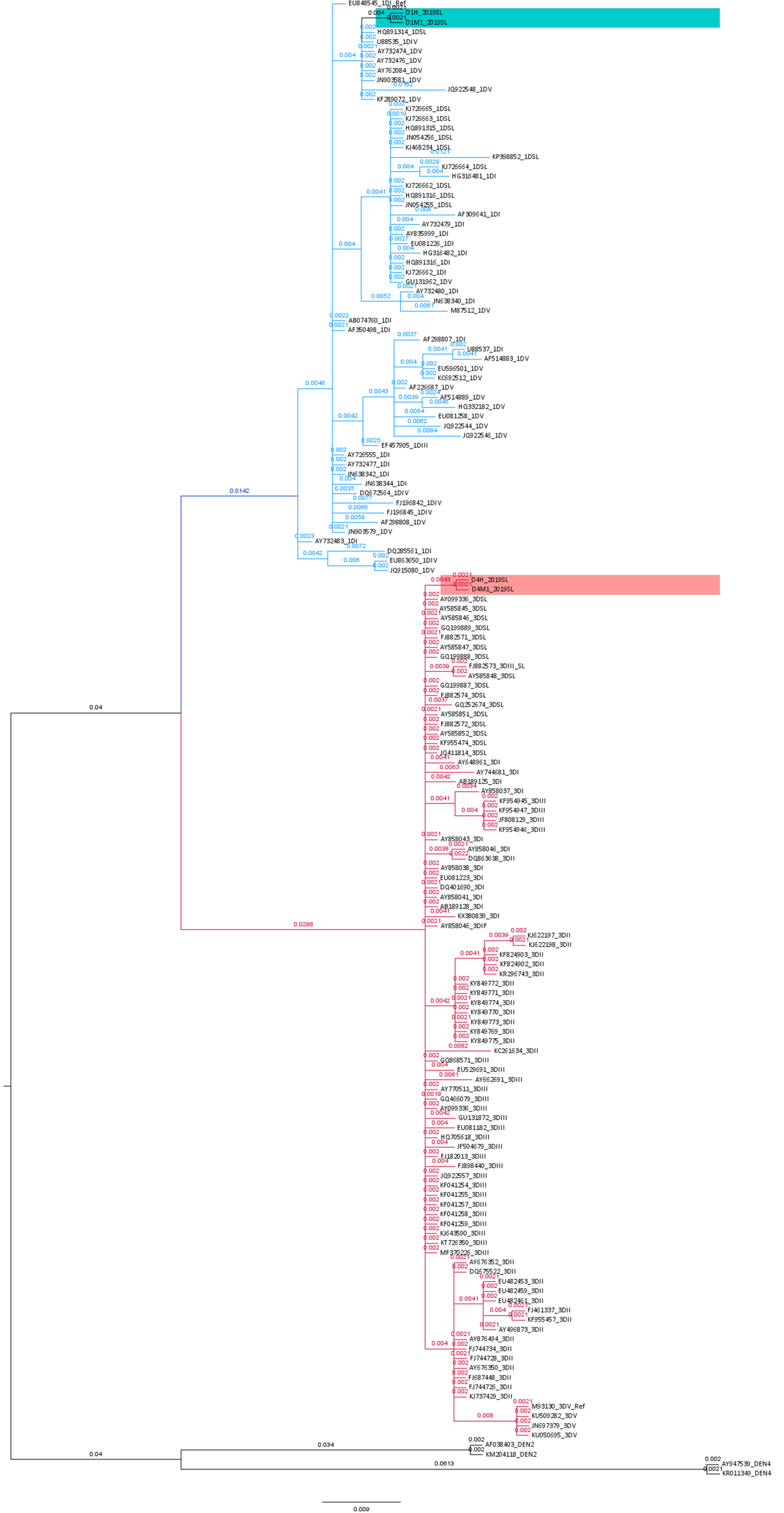

Supplement: Supplementary file 1 [file viruses-13-00421-s001.zip › Supplimentry files/Supplimentry figures/Figure S5.png]
